# Combining next‐generation sequencing and progeny testing for rapid identification of induced recessive and dominant mutations in maize M_2_ individuals

**DOI:** 10.1111/tpj.14431

**Published:** 2019-07-12

**Authors:** Marc C. Heuermann, Mario G. Rosso, Martin Mascher, Ronny Brandt, Henning Tschiersch, Lothar Altschmied, Thomas Altmann

**Affiliations:** ^1^ Leibniz Institute of Plant Genetics and Crop Plant Research (IPK) Gatersleben Corrensstrasse 3 06466 Seeland OT Gatersleben Germany; ^2^ Max Planck‐Genome‐Centre Cologne Max Planck Institute for Plant Breeding Research Carl‐von‐Linné‐Weg 10 50829 Köln Germany

**Keywords:** mutation identification, *dwarf*, EMS mutagenesis, *pale green*, *Zea mays*, zygosity filter, technical advance

## Abstract

Molecular identification of mutant alleles responsible for certain phenotypic alterations is a central goal of genetic analyses. In this study we describe a rapid procedure suitable for the identification of induced recessive and dominant mutations applied to two *Zea mays* mutants expressing a *dwarf* and a *pale green* phenotype, respectively, which were obtained through pollen ethyl methanesulfonate (EMS) mutagenesis. First, without prior backcrossing, induced mutations (single nucleotide polymorphisms, SNPs) segregating in a (M_2_) family derived from a heterozygous (M_1_) parent were identified using whole‐genome shotgun (WGS) sequencing of a small number of (M_2_) individuals with mutant and wild‐type phenotypes. Second, the state of zygosity of the mutation causing the phenotype was determined for each sequenced individual by phenotypic segregation analysis of the self‐pollinated (M_3_) offspring. Finally, we filtered for segregating EMS‐induced SNPs whose state of zygosity matched the determined state of zygosity of the mutant locus in each sequenced (M_2_) individuals. Through this procedure, combining sequencing of individuals and Mendelian inheritance, three and four SNPs in linkage passed our zygosity filter for the homozygous *dwarf* and heterozygous *pale green* mutation, respectively. The *dwarf* mutation was found to be allelic to the *an1* locus and caused by an insertion in the largest exon of the *AN1* gene. The *pale green* mutation affected the nuclear *W2* gene and was caused by a non‐synonymous amino acid exchange in encoded chloroplast DNA polymerase with a predicted deleterious effect. This coincided with lower cpDNA levels in pale green plants.

## Significance Statement

We demonstrate a rapid mapping approach by combining the power of next‐generation sequencing of M_2_ individuals with Mendelian inheritance analysis to identify a recessive and a semi‐dominant mutation in two independent EMS mutagenized maize families.

## Introduction

Maize (*Zea mays* L.) is among the top yielding crops (>1 gigaton) worldwide (FAO, 2017). Despite its agronomic importance and the availability of a high quality reference genome sequence, only a very restricted fraction of maize genes have been assigned to a biological role through direct experimental evidence, either through classical forward genetics like chemical mutagenesis (Gallavotti *et al*., 2010), radiation mutagenesis (Kynast and Riera‐Lizarazu, 2011), and transposon mutagenesis (Williams‐Carrier *et al*., 2010) or through reverse genetics approaches like TILLING (Till *et al*., 2004; Weil and Monde, 2009). More recently, the function of several genes has been studied with the genome editing tool CRISPR/Cas9 (Svitashev *et al*., 2015; Qi *et al*., 2016; Char *et al*., 2017). Conventional techniques like quantitative trait locus (QTL) mapping, genome‐wide association studies (GWAS) and nested association mapping (NAM) are routinely used to dissect complex traits like (seed) yield, plant size or architecture, pathogen resistance and control of metabolic pathways (Peiffer *et al*., 2014; Ding *et al*., 2015; Tang *et al*., 2015). However, maize barely profited from next‐generation mapping techniques (Nannas and Dawe, 2015), although forward genetics have regained momentum over the last years in many species. With the introduction of next‐generation sequencing (NGS) technologies, ethyl methanesulfonate (EMS)‐induced mutations in model organisms like *Caenorhabditis elegans* (Sarin *et al*., 2008), fission yeast (Irvine *et al*., 2009) and *Drosophila melanogaster* (Blumenstiel *et al*., 2009) were identified without the need to create classical mapping populations. Emerging technologies like bulk segregant analysis enabled combined mapping and mutant identification by sequencing pooled DNA of up to 500 F_2_ plants (Schneeberger *et al*., 2009). Zuryn *et al*. (2010) demonstrated that it is possible to identify EMS‐induced mutations by sequencing DNA from three *C. elegans* EMS mutant populations after 4–6 rounds of backcrossing. Hitherto, the success of these methods depends on a large number of individuals or several rounds of backcrossing. Austin *et al*. (2011) omitted backcrossing and detected mutations in EMS‐treated *Arabidopsis thaliana* plants by sequencing a small pooled F_2_ population and subsequent filtering for homozygous regions supporting the detection of recessive mutations only. The idea to directly compare corresponding plants with mutant and wild‐type phenotypes to reveal the causal mutation has been approached in several ways in Arabidopsis, rice, maize and barley (Abe *et al*., 2012; Hartwig *et al*., 2012; Lindner *et al*., 2012; Liu *et al*., 2012; Fekih *et al*., 2013; Nordström *et al*., 2013; Tabata *et al*., 2013; Mascher *et al*., 2014). However, each of these approaches involved preceding backcrossing programs or analysis of large numbers of F_2_ individuals for the assembly of bulks. Addo‐Quaye *et al*. (2017) omitted backcrossing and sequenced pools of *Sorghum bicolor* EMS mutant and wild‐type individuals to filter for homozygous SNPs causing non‐synonymous amino acid exchanges in the mutant population and could identify a recessive mutation. Although they were able to find a homozygous SNP in a protein‐coding region their approach potentially missed mutations not caused by SNPs like insertions or deletions and would furthermore not have enough discriminative power to detect mutations in a heterozygous state, which is relevant for homozygous lethal alleles.

In crops like maize, it is very time consuming to carry out several rounds of backcrossing and the production of large bulks of phenotypically well defined individuals may be limited by several constraints including appropriate cultivation area. Mutagenesis with EMS causes untargeted point mutations in the genome of the treated organism. Phenotypically selected individuals of an appropriately mutagenized population may suffer from low fecundity caused by a heavy genetic burden (high mutational load). Low numbers of mutant offspring, however, prevent immediate application of the aforementioned approaches, which rely on the assembly of larger phenotypically defined pools of plants.

To overcome these limitations, we mapped EMS‐induced mutations in populations of small sets of individually sequenced M_2_ maize mutants and their corresponding wild‐type siblings. Knowledge about the individual zygotic state of the mutation, which we gained by analyzing the segregation of the mutant phenotype in their M_3_ offspring, enabled us to directly filter for the causative SNPs without the need of backcrossing. Thereby, we successfully isolated a recessive mutation causing a dwarf structure and a semi‐dominant mutation responsible for a pale green leaf colour using only 16 individual plants for each mutation.

## Results

### The *dwarf* and *pale green* mutants

**Figure 1:**
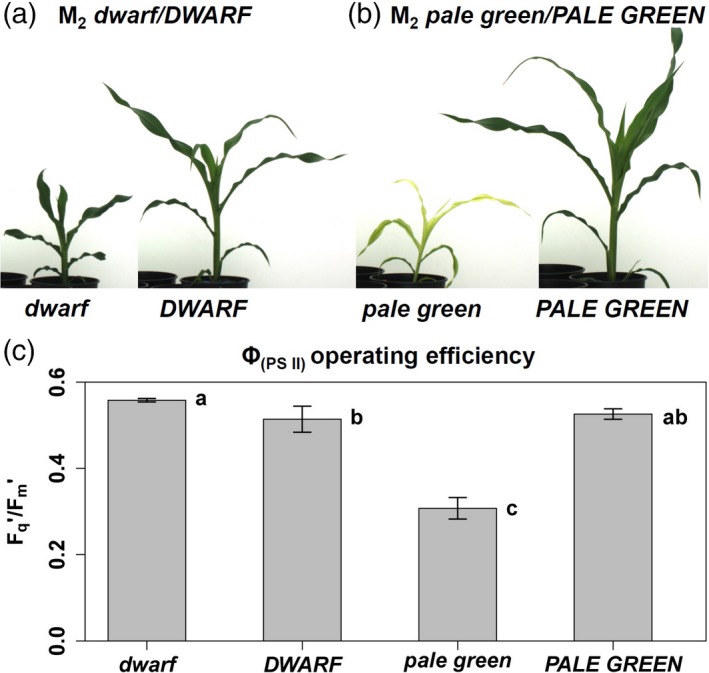
Phenotypes of the M_2_ (a) *dwarf* mutant plant and its corresponding sister plant without a mutant phenotype (*DWARF*) and of the (b) *pale green* mutant plant and its corresponding sister plant without a mutant phenotype (*PALE GREEN*). (c) Operating efficiency of PSII (Φ_(_ _PSII__)_) in M3 progeny in the V1 stage of *dwarf*,* pale green*,*DWARF* and *PALE GREEN*; Barplot of Fq′/Fm′ steady‐state data with standard error of the mean plotted; letters indicate significant differences (anova followed by a post‐hoc Tukey's test, *n* = 5)

We established a *Zea mays* mutant population based of the maize Iodent inbred line PH207 created by pollen EMS mutagenesis (modified protocol of Neuffer *et al*., 2009) as a resource for forward genetics with mutant phenotypes. In total, 7000 M_1_ seeds (unique mutation events) were planted, plants were self‐pollinated, and transferred into the segregating M_2_ population. For a proof of concept, we chose two mutant families – a *dwarf* (M_1_/M_2_ family #1744) and a *pale green* (M_1_/M_2_ family #1754) – to demonstrate direct sequencing‐based identification of EMS‐induced mutations in M_2_ maize mutant populations (Figure 1).

The *dwarf* phenotype was immediately expressed upon germination. During the early growth period (V1, V2; leaf collar method (Abendroth *et al*., 2011)), internodes were drastically shortened and leaves were reduced in length and increased in width. In later stages (V5, Figure 1a), symptoms were less severe but the plants did not catch up with siblings expressing a wild‐type (*DWARF*) phenotype. Furthermore, the *dwarf* plants appeared to have a darker green colouration and their photosystem II (PSII) operating efficiency (Φ_(PSII)_) was increased in comparison with *DWARF* plants (Figure 1c). The *dwarf* mutation also caused the ears to develop anthers next to the kernels and to abort the upper part of the ear (Figure S1).

The *pale green* phenotype was characterized by its pale green colouration which coincided with a decreased plant height. The overall architecture of the plant did not differ from siblings with a wild‐type (*PALE GREEN*) phenotype (V5, Figure 1b). The pale green colour was only observed in emerging and developing leaves. During maturation, leaves turned into darker green, which caused the plant to express a pale green (emerging tissue) to dark green colour (mature tissue) gradient (Figure 1b). This coincided with a strong decrease in Φ_(PSII)_ in *pale green* plants relative to *PALE GREEN* plants, suggesting impaired function of the photosynthesis apparatus (Figure 1c).

### Whole‐genome shotgun sequencing of 32 individuals of segregating M_2_ families

Genomic DNA of 32 individuals, composed of seven *dwarf*, nine *DWARF*, eight *pale green*, and eight *PALE GREEN* plants was sequenced to an average of 19‐fold coverage via WGS sequencing on an Illumina HiSeq 2000 platform (Table 1). About 99% of the reads were each successfully mapped to the B73 reference sequence and properly aligned, half of which were uniquely mapped (Table 1). SNPs, detected in regions with a minimal coverage of 10×, with a minor allele frequency (MAF) of at least 10% and which did not surpass a maximal threshold of 60% heterozygosity (population *dw*/*DW* against *pg*/*PG*) and 10% missing data in both sequenced populations combined, were called between the sequences of the EMS mutagenized PH207‐derived individuals and the B73 reference sequence resulting in 1 073 108 SNPs. Subsequently, SNPs segregating either among the 16 *dwarf*/*DWARF* (1357 SNPs) or among the *pale green*/*PALE GREEN* plants (3536 SNPs) were selected as induced mutations.

**Table 1:** Data alignment of paired‐end sequencing data from individual sequenced gDNAs for each of the mutant plants and their corresponding siblings without a mutant phenotype

Mutant plants *n* = 32	Total reads/plant	Mapped reads/plant	%	Properly paired/plant	%	Uniquely mapped/plant	%	Estimated coverage
*dwarf n* = 7	~432.9 m	~429.9 m	99.3	~428.6 m	99	~215.3 m	49.7	19×
*DWARF n* = 9	~437.6 m	~434.5 m	99.3	~433.2 m	98.9	~218.5 m	49.9	19×
*pale green n* = 8	~457.1 m	~453.9 m	99.3	~452.7 m	99	~229.7 m	50.3	20×
*PALE GREEN n* = 8	~448.3 m	~445.3 m	99.3	~444.1 m	99.1	~222.6 m	49.7	19×

### Dominance/recessiveness of *dwarf* and *pale green* alleles and state of zygosity in sequenced M_2_ individuals

**Figure 2:**
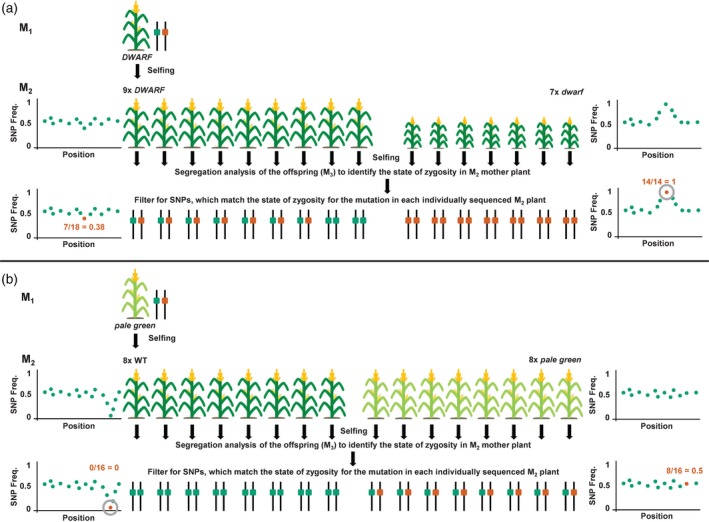
Schematic workflow of the SNP zygosity filter process for the *dwarf* and *pale* green populations. Individuals with and without a mutant phenotype of the M_2_ population were individually sequenced and self‐pollinated. The state of zygosity in M_2_ mother plants was determined by a segregation analysis of the offspring (M_3_). This information was used to filter for SNPs which match the state of zygosity in each individually sequenced M_2_ plant. (a) In the nine *DWARF* plants (seven heterozygous and two homozygous wild‐type) with 18 sequenced alleles, the frequency of the mutant SNP was 0.38 (7/18); and in the seven *dwarf* plants (all homozygous mutant), the expected frequency was 1 (14/14). (b) In the eight *PALE GREEN* plants (all homozygous wild‐type) with 16 sequenced alleles, the expected mutant frequency was 0 (0/16); and in the eight *pale green* mutant plants (all heterozygous, as the allele is homozygous lethal), the expected mutant frequency was 0.5 (8/16).

The individually sequenced mutant plants (M_2_) were, if possible, self‐pollinated or else fertilized with B73 wild‐type pollen and the segregation of the mutant phenotype was monitored in the offspring (M_3_). This allowed the precise determination of the dominance/ recessiveness of the mutations, *dwarf* and *pale green*, and the state of zygosity in each sequenced M_2_ individual (Figure 2).

The individuals expressing a *dwarf* phenotype were hard to self‐pollinate. However, when cross‐pollinated with B73, the resulting offspring expressed only wild‐type (*DWARF*) phenotypes thereby proving recessiveness of the *dwarf* mutation (#15, 17; Table 2). One *DWARF* M_2_ plant was pollinated with B73 and produced offspring with wild‐type phenotype only (#21; Table 2). Progeny of five of the self‐pollinated *DWARF* M_2_ plants segregated for *dwarf* and *DWARF* phenotypes in a 1:3 relation, demonstrating a heterozygous state of the parental M_2_ plant (#18, 19, 20, 23, and 24; Table 2). The offspring of one of the selfed *DWARF* M_2_ plants exhibited only the wild‐type phenotype, therefore originating from a homozygous *DWARF* M_2_ plant (#22; Table 2). From this, we concluded that the *dwarf* mutation is recessive and the sequenced *dwarf* M_2_ plants therefore were homozygous for the mutant (*dwarf*) allele (Figure 2a). *DWARF* M_2_ plants were either homozygous for the wild‐type (*DWARF*) allele or heterozygous (*dwarf*/*DWARF*) (Figures 2a and S4).

**Table 2:** Segregation analysis of the offspring (M_3_) of the individual sequenced M_2_ *dwarf*/ *pale green* (*dw*/*pg*) and *DWARF*/ *PALE GREEN* (*DW*/*PG*) plants. M_3_ offspring segregates into mutant (*mt*) and wild‐type (*WT*) phenotypes

#	M_2_ plant (*mt/WT*)	Pollinator	M_3_ phenotypes		Germinated	Total
			Mutant	*WT*		
1	*1754_5_pg*	Selfed	21	11	32	40
2	*1754_40_pg*	Selfed	3	3	6	35
3	*1754_46_pg*	Selfed	11	9	20	40
4	*1754_49_pg*	Selfed	4	1	5	21
5	*1754_50_pg*	Selfed	1	0	1	2
6	*1754_53_pg*	Selfed	2	2	4	16
7	*1754_63_pg*	Selfed	8	8	16	31
8	*1754_48_PG*	Selfed	0	33	33	40
9	*1754_52_PG*	Selfed	0	40	40	40
10	*1754_54_PG*	Selfed	0	40	40	40
11	*1754_55_PG*	Selfed	0	39	39	40
12	*1754_56_PG*	Selfed	0	38	38	40
13	*1754_58_PG*	Selfed	0	39	39	40
14	*1754_60_PG*	Selfed	0	39	39	40
15	*1744_10_dw*	B73	0	22	22	22
16	*1744_23_dw*	Selfed	1	0	1	1
17	*1744_28_dw*	B73	0	7	7	7
18	*1744_5_DW*	Selfed	12	28	42	42
19	*1744_11_DW*	Selfed	10	29	40	40
20	*1744_13_DW*	Selfed	9	31	40	40
21	*1744_16_DW*	B73	0	38	40	40
22	*1744_21_DW*	Selfed	0	39	40	40
23	*1744_25_DW*	Selfed	10	30	40	40
24	*1744_27_DW*	Selfed	12	28	42	42

*PALE GREEN* M_2_ plants were easily self‐pollinated and their offspring produced only wild‐type phenotypes (#8–14; Table 2), despite one case where we did not get any seeds. The offspring of the *pale green* M_2_ plants suffered from an overall low germination rate. Nonetheless, for six out of seven self‐pollinated *pale green* M_2_ plants, the offspring segregated for mutant (*pale green)* and wild‐type (*PALE GREEN*) phenotypes (#1–4, 6, and 7; Table 2). One *pale green* M_2_ plant produced only a single *pale green* offspring plant. Considering these data and the low germination rate, we concluded that the *pale green* mutation is semi‐dominant causing the *pale green* phenotype in heterozygous individuals, while homozygous *pale green* individuals are embryo lethal. From this we derived that all sequenced *pale green* M_2_ plants were heterozygous, and wild‐type M_2_ siblings were homozygous for the wild‐type (*PALE GREEN*) allele (Figure 2b).

The allele frequency of SNPs expected to be causal for or linked with the mutation was calculated in both individually sequenced populations. For the seven homozygous *dwarf* individuals we expected all 14/14 represented alleles to carry the mutation resulting in a corresponding SNP allele frequency of 1 (Figure 2a). Of the nine sequenced *DWARF* plants, seven were heterozygous and two were homozygous wild‐type. Thus, seven out of the 18 sequenced alleles have to carry the mutation resulting in an expected SNP allele frequency of 0.38 (Figure 2a). For the eight heterozygous *pale green* individuals, eight out of the 16 sequenced alleles have to carry the mutation resulting in an expected SNP frequency of 0.5 (Figure 2b). In the eight homozygous wild‐type *PALE GREEN* plants, all 16 represented alleles are wild‐type and the expected mutant SNP allele frequency therefore was 0 (Figure 2b). The measured allele frequencies of all segregating SNPs are plotted for each population together with the state of the zygosity of every SNP in each individually sequenced M_2_ plant in Figure S4.

### Filtering SNPs for matching allelic states

The precise knowledge of the allelic state of the investigated mutations in each sequenced M_2_ individual allowed filtering for SNPs whose state of zygosity exactly matched that of the mutation across all tested individuals (Figure S4).

In the *dwarf*/*DWARF* M_2_ family only three SNPs (1:241661229–1:243646893; AGPv3) matched the expected zygosity pattern: They were homozygous for the mutant allele in all seven *dwarf* plants (Table 4). In seven *DWARF* plants, the three SNPs were heterozygous and in two *DWARF* plants homozygous for the reference (wt) allele (Table 4). Although we were only able to filter for the precise state of zygosity of six *DWARF* plants (Table 2), calling the allelic state of these SNPs in every sequenced individual of the *dwarf*/*DWARF* population unveiled that at least two of the remaining *DWARF* individuals had to be heterozygous and one was homozygous wild‐type (Table 4).

In the *pale green*/*PALE GREEN* M_2_ family, only four SNPs (10:144036710–10:144996416; AGPv3) matched the expected zygosity pattern and were found to be heterozygous for the mutant allele in the *pale green* plants and homozygous for the reference allele in the *PALE GREEN* plants (Table 4). We had no information about the M_3_ phenotype for one sequenced *pale green* individual and one *PALE GREEN* plant as they failed to produce seeds. However, likewise to the situation in the dwarf population, the mutant allele count in the sequenced individuals allowed the deduction that the *pale green* plant was heterozygous and the *PALE GREEN* was homozygous wild‐type (Table 4).

### Identification of mutant genes

All three SNPs, which exactly matched the zygosity pattern of the *dwarf* mutation, are located on the long arm of chromosome 1 at 241.6, 243.6, and 243.6 Mbp. Here, 173 gene loci of any kind are annotated in this interval ±400 kb. Only five genes have previously been characterized experimentally, two transcription factors and three protein‐coding genes, including the *anther ear 1* gene according to the MaizeGDB (Andorf *et al*., 2016). The mutated recessive *anther ear 1* gene (Zm00001d032961) has been described to cause a similar semi‐dwarf phenotype as shown by the *dwarf* mutant in this study (Emerson and Emerson, 1922; Bensen *et al*., 1995). To test whether the *dwarf* mutation was allelic to the *an1* locus or to the neighbouring *D8* locus also responsible for a *dwarf* phenotype, 20 Mbp downstream of *an1*, we performed allelism tests between our *dwarf* mutant and two independent *an1* mutant lines (*116G* and *116GA*) and between three *D8* mutant lines (121C, 131E, and 131F) retrieved from the Maize Genetics Cooperation Stock Center (Scholl *et al*., 2003; Andorf *et al*., 2016). We crossed our *dwarf/DWARF* mutants with and without a phenotype to either of the publicly available mutants. Every cross between *dwarf* and either of the *116G*‐ or *116GA*‐*dwarf* plants produced only offspring (F_1_) with a dwarf phenotype (#2, 6, 7; Table 3), demonstrating that the tested mutations did not complement each other and thereby causing the recessive *dwarf* mutant phenotype of the F_1_. Crosses between heterozygotes of our *dwarf/DWARF* phenotypes with heterozygotes of both, *116G* and *116GA*, produced *dwarf/DWARF* offspring in a 1:3 ratio (#3, 4; Table 3). Homozygous *dwarf* crossed with heterozygous *DWARF* segregated into half *dwarf* and half *DWARF* (#1, 5, 8; Table 3). If the *dwarf* allele was absent in at least one of the crossing partner, their offspring only expressed wild‐type phenotypes (#9, 10; Table 3). From that we concluded that the *dwarf* mutation is fully allelic to both publicly available *an1* mutants.

**Table 3:** Allelism test between *dwarf* and public *an1* and *D8* mutants. Female and male parents exhibited either *dwarf* or *DWARF* phenotypes and the offspring of the crosses segregated into *dwarf* and *DWARF* phenotypes

#	Female plant (*dwarf/DWARF*)	Male plant (*dwarf*/*DWARF*)	*dwarf*	*DWARF*	Total
1	*dwarf*	*116G_DWARF*	25	23	48
2	*dwarf*	*116G_dwarf*	41	0	41
3	*DWARF*	*116G_DWARF*	15	33	48
4	*DWARF*	*116GA_DWARF*	11	39	50
5	*dwarf*	*116GA_DWARF*	24	26	50
6	*116GA_dwarf*	*dwarf*	35	0	35
7	*116G_dwarf*	*dwarf*	48	0	48
8	*116G_DWARF*	*dwarf*	20	24	44
9	*dwarf*	*116G_DWARF*	0	48	48
10	*DWARF*	*116G_DWARF*	0	50	50
11	*dwarf*	*121C_D8_DWARF*	0	40	40
12	*DWARF*	*131E_D8_dwarf*	18	22	40
13	*DWARF*	*131E_D8_DWARF*	0	40	40
14	*dwarf*	*131E_D8_dwarf*	17	23	40
15	*dwarf*	*131F_D8_dwarf*	18	12	30
16	*DWARF*	*131F_D8_DWARF*	0	30	30

As a negative control, we crossed *dwarf*/*DWARF* mutants with plants carrying a mutant dominant *D8* allele (#11–16; Table 3). *D8‐dwarfs* segregated into 1:1 mutant and wild‐type offspring regardless of the phenotype of their crossing partner. *D8‐DWARF* plants crossed to *dwarf*/*DWARF* only produced wild‐type phenotypes. Therefore, the *dwarf* mutation is not allelic to the *D8* locus also present on chromosome 1.

**Figure 3:**
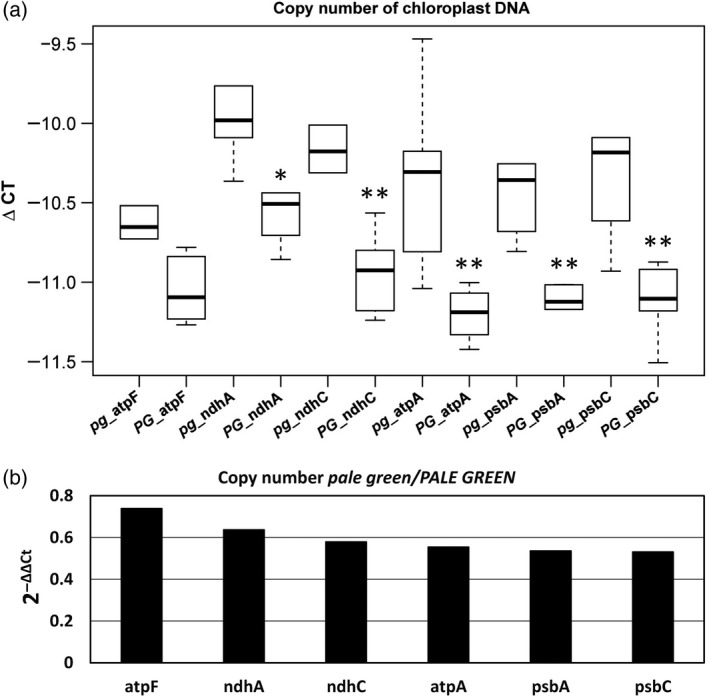
Chloroplast DNA copy number variation between *pale green* and *PALE GREEN* mutants. (a) ΔCt values (cpGOI – nuclear reference gene) of six chloroplast encoded genes; asterisks indicate significance (*n* = 6; unpaired *t‐*test *≤0.05 and **≤0.01 between ΔCt(cpGOI) *pale green* and ΔCt(cpGOI) *PALE GREEN*. (b) cpDNA levels of *pale green* relative to *PALE GREEN* plotted as 2^−ΔΔCt^ (mean ΔCt *pale green*−mean ΔCt *PALE GREEN*).

All four SNPs, which exactly matched the zygosity pattern of the *pale green* mutation, are located on the long arm of chromosome 10 at 144, 144.3, 145, and 145 Mbp. In this interval ±400 kb, 145 gene loci of any kind are predicted of which seven are transcription factors and three are protein‐coding genes previously characterized experimentally, including the *white seedling 2* gene, which expressed a similar mutant phenotype according to the MaizeGDB (Andorf *et al*., 2016). One of the *pg*/*PG* SNPs (10:144036710) causes a non‐synonymous amino acid exchange in the derived amino acid sequence of the Zm00001d026402 gene corresponding to the *white seedling 2* locus. This locus was published to condition shades of pale green mutant phenotypes, dependent on the homo‐ or heteroallelic state of the mutation, caused by a defective nuclear encoded chloroplast DNA polymerase, necessary for the replication of the chloroplast DNA (Udy *et al*., 2012). Therefore, the relative amount of chloroplast DNA in mutant (*pale green*) versus wild‐type (*PALE GREEN*) plants was analyzed by qPCR using primers for specific chloroplast encoded genes. The cycle threshold values (Ct) for each chloroplast gene were normalized to Ct values of a single copy nuclear gene, in other words, the chloroplast DNA levels were normalized to the amount of nuclear DNA to correct for differences in concentration, resulting in delta Ct (ΔCt) values (Figure 3a). Copy number of chloroplast DNA in *pale green* plants reached between 73.9% (atpF) and 53.2% (psbC) relative to the cpDNA levels of the *PALE GREEN* mutants (Figure 3a,b). The amino acid exchange of the 810^th^ amino acid (serine to phenylalanine) was predicted to be of deleterious nature (PROVEAN score −5.8), which can be interpreted as a negative effect on the function or even a loss of function for the mutated protein (Choi *et al*., 2012; Choi and Chan, 2015). Furthermore, the non‐synonymous amino acid exchange at the given position is located in the DNA polymerase A domain part of the protein (The UniProt Consortium, 2017).

### Full‐length sequence analysis of the affected genes

The SNP in the *white seedling 2* gene found in the *pale green* mutant caused an altered sequence of the encoded protein and causes a non‐synonymous amino acid exchange. However, it could not be excluded that additional EMS‐induced sequence alterations were present in this gene. Furthermore, the detected SNPs associated with the recessive *dwarf* mutation rather acted as markers than as the cause of the mutant phenotype, as the *an1* gene is found 300 kb upstream of the SNP at position 1:241 661 229. Therefore, we re‐sequenced the *dwarf* allele of the *anther ear 1* gene and the *pale green* allele of the *white seedling 2* gene and the corresponding wild‐type PH207 alleles.

An insertion was found to be present at position 259 of the large exon (Zm00001d032961_T003, AGPv4) (Andorf *et al*., 2016) of the *anther ear 1* gene in the *dwarf* mutant, which was absent in the *DWARF* and PH207 wild‐type sequence (Figure S2; Primer 612f, Alignment position 3111 ff). According to BLAST and the P‐MITE database the detected additional 256‐bp sequence had a 95% sequence identity with a DTH_Zem60 DNA transposon belonging to the PIF/Harbinger superfamily with a published length of 133 kb (Altschul *et al*., 1997; Chen *et al*., 2014).

Despite some sequencing gaps especially in the intron regions, the genomic sequence of the *pale green* gene was consistent with the PH207 sequence from the Genome Browser (Figure S3).

## Discussion

Here we devised and validated a strategy suitable for rapid detection of induced mutations closely linked to, and even causal to, phenotypically defined mutant loci. This strategy relies on the enormous capacity of the next‐generation sequence technology and the power of computational sequence analyses making high‐coverage WGS sequencing of multiple individuals affordable and comprehensive mutation detection and analysis feasible even for species with large genomes such as maize. The second decisive feature of the strategy is the outstanding discriminative power of Mendelian genetic segregation, which provides the means of filtering thousands of sequence variants for the very few (induced) mutation that are in the immediate vicinity of the phenotypically defined mutant locus and even within the responsible gene.

The success of this approach is dependent on covering a high fraction of the genome by reaching a sufficient sequencing depth for reliable SNP calling and genotyping of the mutants and their corresponding wild‐type siblings. EMS mutagenesis, a process which is strongly influenced by the genotype and the climate conditions of the day of mutagenesis (Neuffer *et al*., 2009), mutates base pairs in a random fashion, so that one can expect the mutations to occur evenly distributed over the chromosome. In the segregating *dwarf* and *pale green* M_2_ populations the mutational load (1357 and 3536 induced mutations) was slightly lower compared with previous EMS mutagenesis in maize (Till *et al*., 2004), which can be explained by lower concentrated EMS used in our mutagenesis. These few thousand sequence variants per mutated genome/per M_2_ family are distributed among the 10 maize chromosomes covering approximately 2.3 Gbp of DNA sequence, which corresponds to *c*. 2200 cm of genetic distance (Su *et al*., 2017). The maximal achievable genetic resolution for each mutant population – 16 individuals derived from 32 meiotic events – is *c*. (1/32) 3 cm, which corresponds to unresolved regions of *c*. 6 cm around the causal mutation assuming identical segregation for all sequence variants. In relation to the 2200 cm of the genome, one would expect four and 10 SNPs for the *dwarf* and *pale green* population to be in the same linkage block, respectively. The numbers of SNPs which passed our zygosity filter were slightly lower than expected which can be explained by the fact that both mutant loci are located on the long arms of their chromosomes, in regions of relatively high recombination frequency (Liu *et al*., 2015).

The low number/density of EMS‐induced mutations in comparison with the occurrence of sequence polymorphism in classical linkage mapping of phenotypically defined F_2_ populations, derived from a cross of a mutant with a genetically divergent wild‐type, has a strong advantage as in the latter approach one is generally confronted with a lot more polymorphisms (by at least two to three orders of magnitude dependent on the species), which will not be linked to the causal mutation. Furthermore, mapping carried out within the same genotypic background eliminates influences of QTL potentially present in mapping populations derived from genetically divergent lines, which may be particularly relevant for the analysis of complex traits.

A further advantage, which should not be overlooked, is saving time. The rapid mapping strategy does neither require crosses nor the creation of mapping populations, which can save years in species with longer generation times as is the case with most crop plants. Any segregating family derived from a selfed heterozygous individual, like an M_2_ family, is sufficient, and only the sequenced individuals need to be subjected to progeny analysis.

The time‐limiting step in this analysis is the phenotyping of the M_3_ generation. Early phenotypes, like those caused by the *dwarf* or *pale green* mutation, which were unambiguously discriminable 1 week after germination, have no additional impact on the time of the experiment. Late phenotypes like flowering time or yield would scale the time of the experiment with the generation time of a species.

Hitherto, we expected that most mutant phenotypes in our *dwarf* and *pale green* populations were caused by transition mutations as a direct result of the mutagen EMS. The *pale green* mutation was identified as a transition SNP in the *white seedling 2* (Zm00001d026402) gene leading to a non‐synonymous amino acid exchange. This gene encodes a chloroplast DNA polymerase, and, if mutated, the plant expresses a pale green phenotype (Udy *et al*., 2012). Although other alleles of the *w2* locus are described to segregate recessively (Andorf *et al*., 2016), the *pale green* mutation is inherited semi‐dominantly. The mutant allele is semi‐dominant over the wild‐type allele and therefore makes the wild‐type *w2* gene haploinsufficient to produce a wild‐type phenotype in a heterozygous state. The mutation is also affected by age‐related penetrance of the phenotype as in the later developmental stage the pale green colour diminishes, probably because the chloroplast biogenesis catches up, which could explain a slight discrepancy in the expected Mendelian segregation.

The mutation was observed in the M_1_ generation, where only dominant mutations would produce a phenotype. Despite the offspring from *pale green* mutants suffering from a low germination rate, every *pale green* M_2_ individual produced segregating mutant offspring with pale green mutant phenotypes, while *PALE GREEN* M_2_ individuals did not. In a diploid organism, the zygosity of an allele can only be homozygous or heterozygous, therefore the *pale green* mutation had to be inherited dominantly as supported by the four SNPs in chromosome 10 passing the stringent zygosity filter. The non‐synonymous amino acid exchange was predicted to have a deleterious effect on the protein function, as it changes the DNA polymerase A domain of the protein, which we could demonstrate by showing that the chloroplast DNA levels were strongly reduced in the *pale green* mutants (Figure 3). Furthermore, the pale green phenotype was visible with the naked eye and the reduced photosystem II operating efficiency was measured (Figure 1b,c), indicating a lower amount of chlorophylls likely because of an impaired chloroplast biogenesis. The other three SNPs, which also passed the required zygosity filter, were probably in linkage. The *pale green* mutant population supported our expectation and allowed the direct identification of an EMS‐induced mutation.

The *dwarf* mutation, conversely, was not caused by a point mutation. The mutation likely occurred spontaneously during, but not necessarily because of, the mutagenesis or during seed amplification immediately before. The dwarf phenotype caused by the *116GA‐an1‐93W1189* allele was also found to derive from a spontaneous mutation which arose in the Maize Genetics Cooperation Stock Center stock (Andorf *et al*., 2016). We identified publicly available mutants of the *anther ear 1* gene (Zm00001d032961) to be fully allelic to our *dwarf* mutation and during re‐sequencing identified an insertion of a DNA transposon in the largest exon, which likely disrupts the function of the gene and leads to the *dwarf* phenotype, which is in concordance with other studies (Bensen *et al*., 1995; Landoni *et al*., 2007). *Anther ear 1* encodes an *ent*‐kaurenoic acid synthase that acts as an entry point enzyme in the pathway of bioactive gibberellins and if mutated leads to a semi‐dwarf structure (Katsumi *et al*., 1964; Bensen *et al*., 1995; Landoni *et al*., 2007; Nelissen *et al*., 2012). Moreover, the anther ear phenotype has been known since the early 20^th^ century (Emerson and Emerson, 1922) and was also expressed in our *dwarf* mutants (Figure S1). We concluded that the SNPs in the dwarf population, which passed the zygosity filter, were in linkage with the insertion in the *an1* gene rather than being causal. Although this was not expected prior to the experiment, it demonstrates the power of the rapid mapping approach, which not only identifies causative SNPs, but also discovers linked mutations using the SNPs solely as markers. This is a strong advantage of sequencing individual plants, as knowledge of the exact allelic state in each sequenced individual allows unambiguous identification of SNPs in linkage with the causal mutation.

A classical approach to identify SNPs in linkage with the causal mutation is bulked segregant analysis (BSA). The power of discrimination of closely linked/causal SNPs from unlinked SNPs and the achieved mapping resolution in BSA depends on the size of the analyzed bulks and the sequencing depth. In a bulk of 16 homozygous mutant plants (equivalent to the number of plants analyzed in this study) an unlinked SNP will show an allele frequency of 1 (occurrence of only one allele) at a theoretical likelihood of (0.25^16^) 2.33e‐10 provided non‐limiting read coverage. Erroneous detection of unlinked SNPs will therefore be rare. The achievable mapping resolution, that is the range covered by linked SNPs, which cannot be resolved with a bulk size of 16 plants (32 meiotic events), will be around 9 cm at an (alpha) error threshold of 5% $\left(1-\sqrt[{32}]{0.05}=0.09\right)$. In experimental studies, the observed mapping resolution of sequenced bulks was close to the theoretical possible resolution. Abe *et al*. (2012) found mapping intervals of 2 Mb in pools of only 20 mutant rice plants, a resolution of 6.66 cm, which is very close to the expected 7 cm $\left(1-\sqrt[{40}]{0.05}=0.07\right)$. Similar high precision was found in a bulked maize RNA‐Seq analysis by Liu *et al*. (2012), who achieved a mapping resolution of 2 Mb (approx. 2 cm) matching the expected 2 cm $\left(1-\sqrt[{128}]{0.05}=0.02\right)$ resolution. In Arabidopsis, Lindner *et al*. (2012) realized a mapping resolution of 4.7 cm with pools of 53 plants compared with the expected 3 cm $\left(1-\sqrt[{106}]{0.05}=0.03\right)$. Even though increasing pool sizes tend to lead to higher mapping resolution, this parameter is strongly dependent on coverage of the NGS data. If the coverage is considerably lower than the pool size, allele frequencies cannot be resolved accurately enough and resolution drops, as seen by Hartwig *et al*. (2012) who achieved around 7 cm with pools of 318 plants despite a theoretically achievable resolution of 0.5 cm.

For zygosity mapping, the likelihood of unlinked SNP to be considered as causative is also (0.25^16^) 2.33e‐10. However, in contrast with the bulk segregant analysis, here the zygosity for each individual SNP position can be assessed. Therefore, only SNPs in perfect linkage exactly follow the same zygosity pattern as the causal SNP, whereas any SNP outside the strong linkage range will deviate from the exact pattern and therefore will not pass the zygosity filter (Table 4). The SNPs in full linkage are indistinguishable from each other and therefore the likelihood is the same for all SNPs sharing the same linkage block. Provided sufficient sequencing depth, the achievable resolution of the rapid mapping approach is therefore only determined by the number of meiotic events and the SNP density.

**Table 4:** SNP Position of the *dwarf*/*DWARF* (*dw*/*DW*) and *pale green*/*PALE GREEN* (*pg*/*PG*) population; allelic state (*mt*/WT) counts the fraction of mutant or wild‐type alleles in each population (*pg*/*PG* and *dw*/*DW*); base exchange from WT (identical to B73) at a certain SNP position; Total allele count in both populations (*pg*/*PG* and *dw*/*DW*); Reference allele count of *pg*/*PG* and *dw*/*DW* in the whole population, respectively; mutant allele count in the population with mutant and WT phenotype

	Population (*mt/WT*)	SNP position (Chr: position [nt])	Allelic state (*mt/WT*)		Base exchange (*WT*/*mt*)	Total allele count (*pg/PG/dw/DW*)	Ref allele count (*WT*)	Mutant allele count (*mt/WT*)
			*mt*	*WT*				
Chr. 10 144–144.9 Mb	*pg/PG*	10:144 036 710	0.5^a^	0^b^	C/T	64	56	8/0
	*pg/PG*	10:144 345 985	0.5^a^	0^b^	C/T	64	56	8/0
	*pg/PG*	10:144 991 851	0.5^a^	0^b^	C/T	64	56	8/0
	*pg/PG*	10:144 996 416	0.5^a^	0^b^	C/T	64	56	8/0
Chr. 1 241–243 Mb	*dw/DW*	1:241 661 229	1^c^	0.388^d^	G/A	64	43	14/7
	*dw/DW*	1:243 560 172	1^c^	0.388^d^	C/A	64	43	14/7
	*dw/DW*	1:243 646 893	1^c^	0.388^d^	G/A	64	43	14/7

a *Pale green* population is heterozygous (50% of alleles were mutant).

b *PALE GREEN* population is homozygous WT (0% of alleles were mutant).

c *dwarf* population is homozygous mutant (100% of alleles were mutant).

d *DWARF* population is either homozygous WT or heterozygous mutant (7/18 or 38.8% of alleles were mutant).

To reach a similar resolution by BSA a larger number of plants would be required and, subsequently, a sufficient sequencing depth needs to be achieved, which depends on the number of individuals in the pool, to unequivocally identify SNPs linked with the mutation. To achieve a mapping resolution of 3 cm with BSA in a diploid species and an alpha threshold of 5%, a pool of 50 individuals, 100 meiotic events $\left(1-\sqrt[{100}]{0.05}=0.03\right)$, and a minimal read coverage of 100× would be required, to sequence every allele in the pool once, assuming an ideal distribution of alleles in the pools. As an ideal distribution is experimentally challenging, read coverage of 2–3× per allele would be necessary to assess the real allele frequency in such pools. However, in most BSA experiments, the coverage is lower than the pool of individual alleles and therefore the assessment of the real allele frequency on whole‐genome sequencing data alone was rarely possible (Hartwig *et al*., 2012). In contrast, 16 individuals with 20× sequencing coverage each (16*20× = 320×) are on average needed for a similar resolution in the zygosity filter approach. In absolute numbers the zygosity filter procedure is two‐ to three‐fold more efficient than a classical BSA.

One of the strongest arguments for zygosity mapping is the fact that mutations can be mapped in a heterozygous state without the need of prior backcrossing, which is not possible with BSA. The *pale green* mutation mapped in this study would not be detectable in sequenced pools of M_2_ mutants as its SNP frequency of 0.5 would be indistinguishable from that of unlinked SNPs segregating in the population. Although it has been shown that heterozygous mutations can be generally mapped in pools of plants (Lindner *et al*., 2012), they require several rounds of prior backcrossing, which can be skipped performing a zygosity filter on sequenced individuals.

The only drawback of individually sequencing plants instead of performing bulk segregant analysis, is the increase in cost due to the necessity to reach the coverage 16 times per population instead of only once following the SHORE map approach (Schneeberger *et al*., 2009). However, working with maize rather than with Arabidopsis the coincidence of a drastically longer generation time, limited greenhouse space, and most importantly the gain in time, justifies our rapid mapping technique. Furthermore, the steady decrease in sequencing cost according to Wetterstrand (2017) will likely to continue in the coming years, making our rapid mapping approach much more feasible for a wide range of plant species and even with higher level of ploidy like wheat or oilseed rape. The only requirement for a successful adaptation for the rapid mapping approach is an available high quality reference sequence, which does not necessarily have to be from the same genotype. We compared sequence variation in 10 Mb regions around the two candidate mutations between B73_AGPv3 and PH207 and found no major structural rearrangements (Figure S5). Even though we performed SNPs calling of the individually sequenced plants in the PH207 background against the reference sequence of B73, as the genome of PH207 was not published when the work in this study was performed, which lead to a much larger number of SNPs, mainly not EMS specific, filtering for only segregating SNPs was enough to enable the zygosity filter to precisely identify the causative or linked SNPs.

## Experimental procedures

### Plant material

A *Zea mays* pollen mutagenized EMS population of the PH207 inbred line was generated with EMS concentrations (10–20 mm) according to (Neuffer and Coe, 1978). Among others, a *dwarf* (Figure 1a) and a *pale green* (Figure 1b) mutant were found during phenotypic screening using IPK's automated phenotyping platform for large plants (described in Junker *et al*., 2015). The mutant lines (internally numbered: 1744 and 1754) were named according to their phenotype, *dwarf* and *pale green*. *Zea mays* plants were grown in the scanalyzer system for large plants under standard conditions as described in (Junker *et al*., 2015). After EMS mutagenesis, M_1_ mutants were self‐pollinated and transferred into a segregating M_2_ population. M_2_ individuals of those populations, which were subjected to sequencing, were self‐pollinated and transferred into the M_3_ generation. For allelism tests, published mutants of the *an1* locus *116G an1* (Emerson and Emerson, 1922) and *116GA an1‐93W1189* and D8 locus *121C‐D8‐1* (Phinney, 1956) and *131D‐D8‐N1452*,* 131E‐D8‐N1591* and *131F‐D8‐N2023* (Neuffer, 1990) were ordered from the Maize Genetics Cooperation Stock Center (http://maizecoop.cropsci.uiuc.edu/).

### Chlorophyll fluorescence

Photosystem II (PSII) operating efficiency (Φ_PSII _= Fq′/Fm′) was measured via imaging with a custom FluorCam device (Photon Systems Instruments, http://www.psi.cz/) integrated in the IPK phenotyping platform for small plants (Tschiersch *et al*., 2017). Photochemical quenching of fluorescence by open PSII centres (Fq′) was computed by subtracting the maximal fluorescence (Fm′) in the light adapted leaf from the fluorescence emission (F′) from the light adapted leaf (Fq′ = Fm′ − F′; Baker, 2008). Young *dwarf*,* DWARF*,* pale green* and *PALE GREEN* plants (V1 stage), five replicates each, were light adapted to 500 μmol × m^−2 ^× sec^−1^ PAR for 15–20 min prior to the measurement. Imaging was performed after additional 20 sec of illumination with adaptation light and then photosystem II was saturated with an 800 msec light pulse (4100 μmol × m^−2^ × sec^−1^). Mean values of leaf area of top view images were taken as one measurement.

### Isolation of DNA

Shoot material from seven *dwarf*, nine *DWARF*, eight *pale green*, and eight *PALE GREEN* M_2_ plants, each exhibiting an identified phenotype, was quenched in liquid nitrogen and homogenized. DNA was extracted using the Qiagen DNeasy Plant mini Kit according to the manufacturer's protocol. In total, 32 DNA samples were used for Illumina sequencing. For qPCR, whole DNA (nuclear + plastidic) was extracted from *pale green* and *PALE GREEN* M_3_ mutants (six DAS [days after sowing]; whole plant) via a cetyltrimethyl ammonium bromide (CTAB) protocol developed in the GABI‐Kat project (Rosso *et al*., 2003).

### Whole‐genome shotgun sequencing

Thirty‐two Illumina uniquely barcoded paired‐end (2 × 100 bp) libraries were generated from sheared DNA (fragment size 400–500 bp) as described by Meyer and Kircher (2010). Prior to WGS, each sample was partially sequenced to determine its precise molarity. This information was used to optimize the loading of the flow cell for WGS. The 32 libraries were paired‐end sequenced in 32 lanes on four flow cells on the Illumina HiSeq 2000 instrument. All DNAs were individually barcoded and paired‐end sequenced together in one lane of the Illumina Flow Cells, 32 lanes altogether. The data were made publicly available under the accession number PRJEB31849 at the European Nucleotide Archive http://www.ebi.ac.uk/ena/data/view/PRJEB31849.

### SNP calling

Reads were mapped to the maize reference genome AGPv3 (Schnable *et al*., 2009) with BWA‐mem version 0.7.10 (Li, 2013). The resultant SAM files were converted to BAM format with SAMtools (Li *et al*., 2009) and sorted by position with Novosort (http://www.novocraft.com/products/novosort/). Multiple BAM files per individual were merged with Picard (https://broadinstitute.github.io/picard/). SNP calling was performed with SAMtools version 0.1.19 using the parameters ‘‐D ‐Q13 ‐q 10’ for the command ‘mpileup’. The SNPs were filtered using the AWK script ‘gen_call.awk’ (Mascher *et al*., 2013). Genotype calls with coverage (DP) below 10 or genotype quality (GQ) below 10 were set to missing. Only SNP with a MAF of at least 10%, with up to 10% missing data and no more than 60% heterozygous calls were retained and imported into the R statistical environment (R Core Team, 2018) for further analysis. Allele frequencies were computed for plants with mutant and wild‐type phenotypes separately and plotted along the genome using standard R plot functions, excluding SNPs with a mutant allele frequency >0.7 in both sets.

### Segregation analysis

The individual sequenced M_2_ plants were selfed and up to 40 kernels (M_3_) from the offspring were planted (Table 2). Two weeks after sowing, plants were screened for *dwarf* and *pale green* phenotype, respectively. Thereby, the precise state of zygosity of the parents could be determined, based on the specific segregation pattern of the offspring.

### Allelism test

Allelism tests tested whether the mapped *dwarf* allele is allelic to either the *an1* (Emerson and Emerson, 1922) or the D8 (Neuffer, 1990) locus on the long arm of chromosome 1. Therefore, M_3_ *dwarf* plants were crossed with *116G‐an1* and *116GA‐an1‐93W1189* and 121C‐D8‐1, *131D‐D8‐N1452*,* 131E‐D8‐N1591*, and *131F‐D8‐N2023* mutants with and without dwarf phenotype. Offspring was sown and segregation frequencies were analyzed.

### qPCR of chloroplast genes

Genomic DNAs of six *PALE GREEN* and six *pale green* M_3_ plants were diluted to a concentration of 50 ng μl^−1^. qPCR was run on a 7900HT Fast Real‐Time PCR System (Applied Biosystems, Darmstadt, Germany) with a standard protocol. Reaction volume was 10 μl (1× *Power* SYBR Green PCR Master Mix (ThermoFisher Scientific, Darmstadt, Germany); 500 nm primers; 50 ng DNA template). Primer sequences of the chloroplastic encoded genes (atpA, atpF, ndhA, ndhC, psbA, and psbC) and the nuclear encoded gene (HMG‐1) were designed according to the publication by Udy *et al*. (2012). qPCR was performed in six biological replicates, each consisting of three technical replicates for each primer tested. Delta Ct values (ΔCt) were computed by subtracting the arithmetic mean of the technical replicates of nuclear encoded HMG‐1 gene from the arithmetic mean of the gene of interest (GOI) *pale green* and GOI *PALE GREEN*, respectively (ΔCt = Ct GOI *pale green/PALE GREEN*−Ct HMG‐1). Subsequently, delta delta Ct values (ΔΔCt) were computed (mean ΔCt *pale green−*mean ΔCt *PALE GREEN*) and transformed to a linear scale (2^−ΔΔCt^). An unpaired *t‐*test decided whether differences between the ΔCt *PALE GREEN* and *pale green* levels for each tested gene were significant.

### Deep candidate re‐sequencing

*De novo* assembled NGS data of *DWARF* and a *PALE GREEN* plants was used as a template for primer design. Nested primers were designed to amplify the *an1* and *w2* locus (for primer sequences see Table S1). PCR fragment matching the expected size were purified using the QIAquick Gel Extraction Kit and sequenced by LGC Genomics GmbH. Sequences were aligned and analyzed with R statistical environment (R Core Team, 2018) and SeqMan Pro from DNASTAR Inc.

### Analysis of structural variation

DNA sequences 5 and 0.25 Mb up‐ and downstream of each candidate gene from B73_AGPv3 and PH207 were downloaded from the MaizeGDB (Andorf *et al*., 2016) and structural rearrangements between the two ecotypes were visualized by creating dotplots using the Gepard software version 1.40 (Krumsiek *et al*., 2007).

## Author contributions

MH and TA planned and designed the research. MH, MR, RB, HT, and LA performed the experiments. MH and MM analyzed data. MH, MM, and TA wrote the manuscript with contributions from MR and RB. Please correspond with MH and TA.

## Conflict of interest

The authors declare no conflict of interest.

## Supporting information


**Figure S1.** Phenotype of young dwarf and PH207 ears.

**Figure S2.** Alignment and strategy view of the re‐sequenced *an1* gene.

**Figure S3.** Alignment and strategy view of the re‐sequenced *w2* gene.

**Figure S4.** Allele frequencies of the segregating SNPs each in aggregated plots and individual plots.

**Figure S5.** Dot plots of the 10 MB and 500 kB regions surrounding the *an1* (Chr.1) and *w2* (Chr.10) gene.

**Table S1.** Primer sequences used for the re‐sequencing of the *an1* and *w2* gene loci in the mutant populations.

## Data Availability

The data were made publicly available under the accession number PRJEB31849 at the European Nucleotide Archive http://www.ebi.ac.uk/ena/data/view/PRJEB31849.
