# Supplementary figures and images for: Combining next‐generation sequencing and progeny testing for rapid identification of induced recessive and dominant mutations in maize M2 individuals
Source: Plant J. 2019 Jul 12;100(4):851–62. doi: 10.1111/tpj.14431 (PMC6899793; doi:10.1111/tpj.14431)

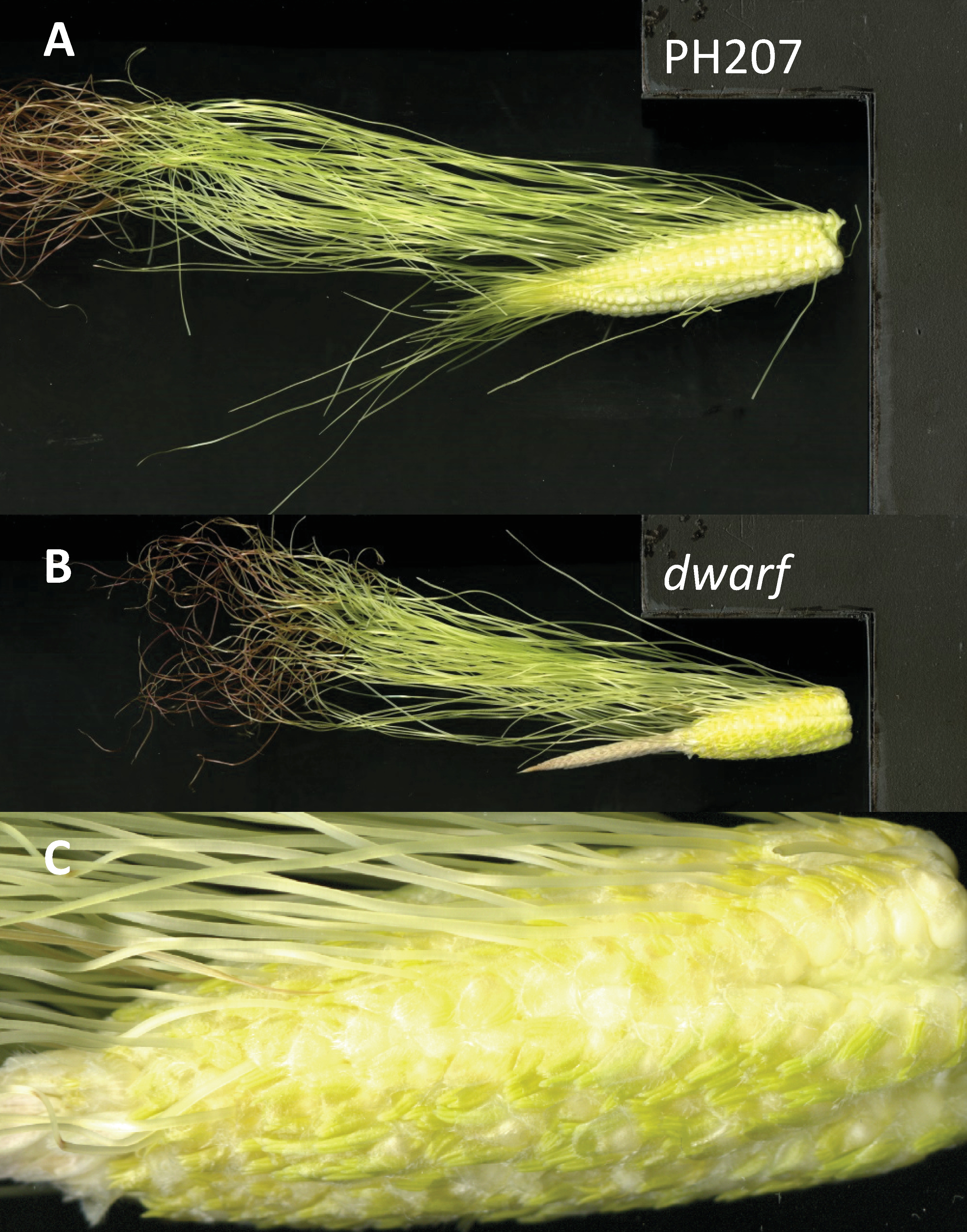

Supplement: Supplementary file 1 — Figure S1. Phenotype of young dwarf and PH207 ears. [file TPJ-100-851-s001.jpg]
