# Supplementary material for: Combining next‐generation sequencing and progeny testing for rapid identification of induced recessive and dominant mutations in maize M2 individuals
Source: Plant J. 2019 Jul 12;100(4):851–62. doi: 10.1111/tpj.14431 (PMC6899793; doi:10.1111/tpj.14431)
Supplement: Supplementary file 2 — Figure S2. Alignment and strategy view of the re‐sequenced an1 gene. [file TPJ-100-851-s002.pdf]

Figure S2: Alignment and strategy view of the re-sequenced *an1* gene in the *dwarf* (1744) and *DWARF* (WT) mutants, B73 (AGPv4; Chr1:244856531-244869541) and PH207 (Chr1:244828512-244841534)

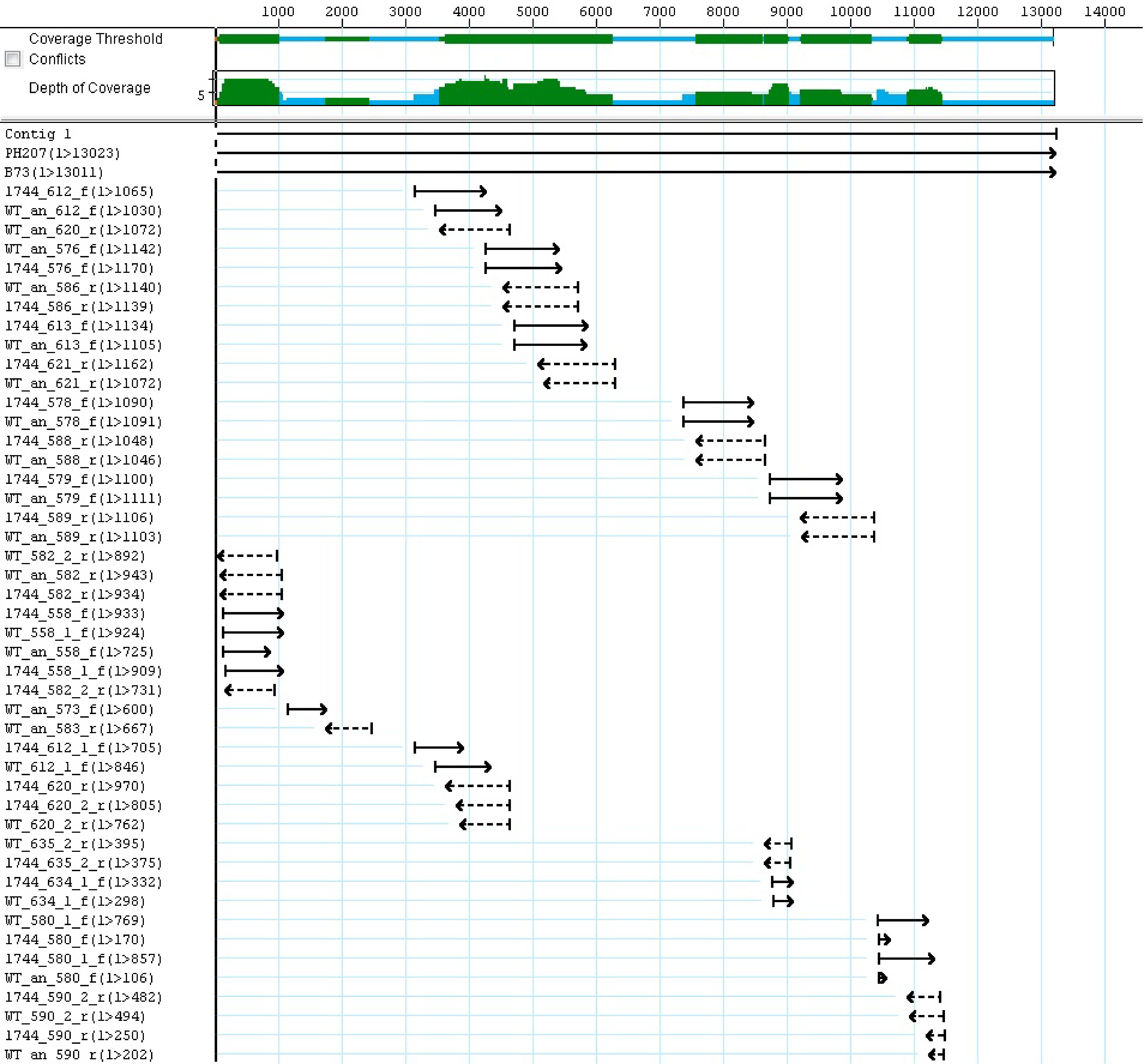

1000
2000
3000
4000
5000
6000
7000
8000
9000
10000
11000
12000
13000
14000
Coverage Threshold
Conflicts
Depth of Coverage
5
Contig 1
PH207(1>13023)
B73(1>13011)
1744_612_f(1>1065)
WT_an_612_f(1>1030)
WT_an_620_r(1>1072)
WT_an_576_f(1>1142)
1744_576_f(1>1170)
WT_an_586_r(1>1140)
1744_586_r(1>1139)
1744_613_f(1>1134)
WT_an_613_f(1>1105)
1744_621_r(1>1162)
WT_an_621_r(1>1072)
1744_578_f(1>1090)
WT_an_578_f(1>1091)
1744_588_r(1>1048)
WT_an_588_r(1>1046)
1744_579_f(1>1100)
WT_an_579_f(1>1111)
1744_589_r(1>1106)
WT_an_589_r(1>1103)
WT_582_2_r(1>892)
WT_an_582_r(1>943)
1744_582_r(1>934)
1744_558_f(1>933)
WT_558_1_f(1>924)
WT_an_558_f(1>725)
1744_558_1_f(1>909)
1744_582_2_r(1>731)
WT_an_573_f(1>600)
WT_an_583_r(1>667)
1744_612_1_f(1>705)
WT_612_1_f(1>846)
1744_620_r(1>970)
1744_620_2_r(1>805)
WT_620_2_r(1>762)
WT_635_2_r(1>395)
1744_635_2_r(1>375)
1744_634_1_f(1>332)
WT_634_1_f(1>298)
WT_580_1_f(1>769)
1744_580_f(1>170)
1744_580_1_f(1>857)
WT_an_580_f(1>106)
1744_590_2_r(1>482)
WT_590_2_r(1>494)
1744_590_r(1>250)
WT_an_590_r(1>202)

```
                     10        20        30        40        50        60        70        80
                   gTGGACGACCCcAAGWACGTTTGTATCACCGGCTCTTTGTGGGCAAAATTAAGGGATTGCCTTGACCTDTGTGGTCGgCATTGAGG
PH207(1>13023)   → gtggacgaccc-aagtacgtttgtatcaccggctctttgtgggcaaaattaagggattgccttgacctgtgtggtcggcattgagg
B73(1>13011)     →  tggacgaccccaagaacgtttgtatcaccggctctttgtgggcaaaattaagggattgccttgacctatgtggtcggcattgagg
WT_582_2_r(1>892)   ←                                                           TTTTTTTTTTTTTTTTTTTTTTTGTCC-CTTATATG
WT_an_582_r(1>943)  ←                                                                                           GA-G

                   90        100       110       120       130       140       150       160       170
                   AACGGGGTTGAGAAAGATCCGGCCCTTTGTGGGCACCTCAAAGAGGAGTAGGACACCTTGGTGGTGTGACCGAACCTCGGTAAAAA
PH207(1>13023)   → aacggggttgagaaagatccggccctttgtgggcacctcaaagaggagtaggacaccttggtggtgtgaccgaacctcggtaaaaa
B73(1>13011)     → aacggggttgagaaagatccagccctttgtgggcacctcaacgaggagtacaacaccttggtggtgtgaccgaacctcggtaaaaa
WT_582_2_r(1>892)   ← AA--GGGTTGAGAAAGATCCGGCCCTTTGTGGGCACCTCAAAGAGGAGTAGGACACCTTGGTGGTGTGACCGAACCTCGGTAAAAA
WT_an_582_r(1>943)  ← AACGGGGTTGAGAAAGATCCGGCCCTTTGTGGGCACCTCAAAGAGGAGTAGGACACCTTGGTGGTGTGACCGAACCTCGGTAAAAA
1744_582_r(1>934)   ←     GGTTGAGAAAGATCCGGCCCTTTGTGGGCACCTCAAAGAGGAGTAGGACACCTTGGTGGTGTGACCGAACCTCGGTAAAAA
1744_558_f(1>933)   →                                       GAGGAGTAGGACACCTTGGTGGTGTGACCGAACCTCGGTAAAAA
WT_558_1_f(1>924)   →                                       GAGGAGTAGGACACCTTGGTGGTGTGACCGAACCTCGGTAAAAA
WT_an_558_f(1>725)  →                                       GAGGAGTAGGACACCTTGGTGGTGTGACCGAACCTCGGTAAAAA
1744_558_1_f(1>909) →                                                            TGTGACCGAACCTCGGTAAAAA
1744_582_2_r(1>731) ←                                                                                 AAAAA

                   180       190       200       210       220       230       240       250
                   TCGTGTGTTTTGTGTGCTTCATTGTGGTTGTGACTTGGTCATTATCATCTCAGATCTGTGATCTATCTAGTTAACTTTGGTTCAAT
PH207(1>13023)   → tcgtgtgttttgtgtgcttcattgtggttgtgacttggtcattatcatctcagatctgtgatctatctagttaactttggttcaat
B73(1>13011)     → ttgtgtgtcttgtgtgcttcattgtggttgtgacttggtcattatcatctcagatctgtgatctatctagttaactttagttcaat
WT_582_2_r(1>892)   ← TCGTGTGTTTTGTGTGCTTCATTGTGGTTGTGACTTGGTCATTATCATCTCAGATCTGTGATCTATCTAGTTAACTTTGGTTCAAT
WT_an_582_r(1>943)  ← TCGTGTGTTTTGTGTGCTTCATTGTGGTTGTGACTTGGTCATTATCATCTCAGATCTGTGATCTATCTAGTTAACTTTGGTTCAAT
1744_582_r(1>934)   ← TCGTGTGTTTTGTGTGCTTCATTGTGGTTGTGACTTGGTCATTATCATCTCAGATCTGTGATCTATCTAGTTAACTTTGGTTCAAT
1744_558_f(1>933)   → TCGTGTGTTTTGTGTGCTTCATTGTGGTTGTGACTTGGTCATTATCATCTCAGATCTGTGATCTATCTAGTTAACTTTGGTTCAAT
WT_558_1_f(1>924)   → TCGTGTGTTTTGTGTGCTTCATTGTGGTTGTGACTTGGTCATTATCATCTCAGATCTGTGATCTATCTAGTTAACTTTGGTTCAAT
WT_an_558_f(1>725)  → TCGTGTGTTTTGTGTGCTTCATTGTGGTTGTGACTTGGTCATTATCATCTCAGATCTGTGATCTATCTAGTTAACTTTGGTTCAAT
1744_558_1_f(1>909) → TCGTGTGTTTTGTGTGCTTCATTGTGGTTGTGACTTGGTCATTATCATCTCAGATCTGTGATCTATCTAGTTAACTTTGGTTCAAT
1744_582_2_r(1>731) ← TCGTGTGTTTTGTGTGCTTCATTGTGGTTGTGACTTGGTCATTATCATCTCAGATCTGTGATCTATCTAGTTAACTTTGGTTCAAT

                   260       270       280       290       300       310       320       330       340
                   TATTCCGCTACATTTATTCATTGTAGATCTGAGCAGTTTCTATTAAGTTCGCAGTTGAACAAGTGATTTGCGATTAAACCGACCTC
PH207(1>13023)   → tattccgctacatttattcattgtagatctgagcagtttctattaagttcgcagttgaacaagtgatttgcgattaaaccgacctc
B73(1>13011)     → tattccgctacatt-attcattgtagatctgagtagtttctattaagttcacagttgaacaagtgatttgcgattaaaccggcctc
WT_582_2_r(1>892)   ← TATTCCGCTACATTTATTCATTGTAGATCTGAGCAGTTTCTATTAAGTTCGCAGTTGAACAAGTGATTTGCGATTAAACCGACCTC
WT_an_582_r(1>943)  ← TATTCCGCTACATTTATTCATTGTAGATCTGAGCAGTTTCTATTAAGTTCGCAGTTGAACAAGTGATTTGCGATTAAACCGACCTC
1744_582_r(1>934)   ← TATTCCGCTACATTTATTCATTGTAGATCTGAGCAGTTTCTATTAAGTTCGCAGTTGAACAAGTGATTTGCGATTAAACCGACCTC
1744_558_f(1>933)   → TATTCCGCTACATTTATTCATTGTAGATCTGAGCAGTTTCTATTAAGTTCGCAGTTGAACAAGTGATTTGCGATTAAACCGACCTC
WT_558_1_f(1>924)   → TATTCCGCTACATTTATTCATTGTAGATCTGAGCAGTTTCTATTAAGTTCGCAGTTGAACAAGTGATTTGCGATTAAACCGACCTC
WT_an_558_f(1>725)  → TATTCCGCTACATTTATTCATTGTAGATCTGAGCAGTTTCTATTAAGTTCGCAGTTGAACAAGTGATTTGCGATTAAACCGACCTC
1744_558_1_f(1>909) → TATTCCGCTACATTTATTCATTGTAGATCTGAGCAGTTTCTATTAAGTTCGCAGTTGAACAAGTGATTTGCGATTAAACCGACCTC
1744_582_2_r(1>731) ← TATTCCGCTACATTTATTCATTGTAGATCTGAGCAGTTTCTATTAAGTTCGCAGTTGAACAAGTGATTTGCGATTAAACCGACCTC

                   350       360       370       380       390       400       410       420       430
                   AAGACCGGTTGAACATGCCTCACTTGTTAAACCGTCTTTTTCTCATAAAAGTTCGAAGTTTATTGAGAATTGCAGGTGTAGCCTAT
PH207(1>13023)   → aagaccggttgaacatgcctcacttgttaaaccgtctttttctcataaaagttcgaagtttattgagaattgcaggtgtagcctat
B73(1>13011)     → aagaccggttgaacatgcctcacttgttaaaccgtctttttctcataaaagttcgaagtttattgagaattgcaggtgttgcctat
WT_582_2_r(1>892)   ← AAGACCGGTTGAACATGCCTCACTTGTTAAACCGTCTTTTTCTCATAAAAGTTCGAAGTTTATTGAGAATTGCAGGTGTAGCCTAT
WT_an_582_r(1>943)  ← AAGACCGGTTGAACATGCCTCACTTGTTAAACCGTCTTTTTCTCATAAAAGTTCGAAGTTTATTGAGAATTGCAGGTGTAGCCTAT
1744_582_r(1>934)   ← AAGACCGGTTGAACATGCCTCACTTGTTAAACCGTCTTTTTCTCATAAAAGTTCGAAGTTTATTGAGAATTGCAGGTGTAGCCTAT
1744_558_f(1>933)   → AAGACCGGTTGAACATGCCTCACTTGTTAAACCGTCTTTTTCTCATAAAAGTTCGAAGTTTATTGAGAATTGCAGGTGTAGCCTAT
WT_558_1_f(1>924)   → AAGACCGGTTGAACATGCCTCACTTGTTAAACCGTCTTTTTCTCATAAAAGTTCGAAGTTTATTGAGAATTGCAGGTGTAGCCTAT
WT_an_558_f(1>725)  → AAGACCGGTTGAACATGCCTCACTTGTTAAACCGTCTTTTTCTCATAAAAGTTCGAAGTTTATTGAGAATTGCAGGTGTAGCCTAT
1744_558_1_f(1>909) → AAGACCGGTTGAACATGCCTCACTTGTTAAACCGTCTTTTTCTCATAAAAGTTCGAAGTTTATTGAGAATTGCAGGTGTAGCCTAT
1744_582_2_r(1>731) ← AAGACCGGTTGAACATGCCTCACTTGTTAAACCGTCTTTTTCTCATAAAAGTTCGAAGTTTATTGAGAATTGCAGGTGTAGCCTAT

                   440       450       460       470       480       490       500       510
                   TCACCCCCCTCTAGGCTACTTTCAATGGATCAGTTCCCACCCCCATGGTCTTGGCCTTTGCCTTGGTGAGCATGGTCGTGCGCCAT
PH207(1>13023)   → tcacccccctctaggctactttcaatggatcagttcccacccccatggtcttggcctttgccttggtgagcatggtcgtgcgccat
B73(1>13011)     → tcacccccctctaggctactttcaatggatcagttcccacccacatggtcttggcctttgccttggtgagcatggtcgtgcgccat
WT_582_2_r(1>892)   ← TCACCCCCCTCTAGGCTACTTTCAATGGATCAGTTCCCACCCCCATGGTCTTGGCCTTTGCCTTGGTGAGCATGGTCGTGCGCCAT
WT_an_582_r(1>943)  ← TCACCCCCCTCTAGGCTACTTTCAATGGATCAGTTCCCACCCCCATGGTCTTGGCCTTTGCCTCGGTGAGCATGGTCGTGCGCCAT
1744_582_r(1>934)   ← TCACCCCCCTCTAGGCTACTTTCAATGGATCAGTTCCCACCCCCATGGTCTTGGCCTTTGCCTTGGTGAGCATGGTCGTGCGCCAT
1744_558_f(1>933)   → TCACCCCCCTCTAGGCTACTTTCAATGGATCAGTTCCCACCCCCATGGTCTTGGCCTTTGCCTTGGTGAGCATGGTCGTGCGCCAT
WT_558_1_f(1>924)   → TCACCCCCCTCTAGGCTACTTTCAATGGATCAGTTCCCACCCCCATGGTCTTGGCCTTTGCCTTGGTGAGCATGGTCGTGCGCCAT
WT_an_558_f(1>725)  → TCACCCCCCTCTAGGCTACTTTCAATGGATCAGTTCCCACCCCCATGGTCTTGGCCTTTGCCTTGGTGAGCATGGTCGTGCGCCAT
```

```
                                440       450       460       470       480       490       500       510
                         TCACCCCCCTCTAGGCTACTTTCAATGGATCAGTTCCCACCCCCATGGTCTTGGCCTTTGCCTTGGTGAGCATGGTCGTGCGCCAT
1744_558_1_f(1>909)  →   TCACCCCCCTCTAGGCTACTTTCAATGGATCAGTTCCCACCCCCATGGTCTTGGCCTTTGCCTTGGTGAGCATGGTCGTGCGCCAT
1744_582_2_r(1>731)  ←   TCACCCCCCTCTAGGCTACTTTCAATGGATCAGTTCCCACCCCCATGGTCTTGGCCTTTGCCTTGGTGAGCATGGTCGTGCGCCAT

                           520       530       540       550       560       570       580       590       600
                         GTACCCAGGAAGCTCGCAGGGGGATGTGCGGGTGAAGGGGTAGTGCTTGGTGGGGTCGGCATAGAAGGCAGCACACTCGAGCGATG
PH207(1>13023)       →   gtacccaggaagctcgcagggggatgtgcgggtgaaggggtagtgcttggtggggtcggcatagaaggcagcacactcgagcgatg
B73(1>13011)         →   gtacccaggaagctcgta-ggggatgtgcgggtgaaggggcagtgcttggtggggtcggcatagaaggcggcacactcgagcggtg
WT_582_2_r(1>892)    ←   GTACCCAGGAAGCTCGCAGGGGGATGTGCGGGTGAAGGGGTAGTGCTTGGTGGGGTCGGCATAGAAGGCAGCACACTCGAGCGATG
WT_an_582_r(1>943)   ←   GTACCCAGGAAGCTCGCAGGGGGATGTGCGGGTGAAGGGGTAGTGCTTGGTGGGGTCGGCATAGAAGGCAGCACACTCGAGCGATG
1744_582_r(1>934)    ←   GTACCCAGGAAGCTCGCAGGGGGATGTGCGGGTGAAGGGGTAGTGCTTGGTGGGGTCGGCATAGAAGGCAGCACACTCGAGCGATG
1744_558_f(1>933)    →   GTACCCAGGAAGCTCGCAGGGGGATGTGCGGGTGAAGGGGTAGTGCTTGGTGGGGTCGGCATAGAAGGCAGCACACTCGAGCGATG
WT_558_1_f(1>924)    →   GTACCCAGGAAGCTCGCAGGGGGATGTGCGGGTGAAGGGGTAGTGCTTGGTGGGGTCGGCATAGAAGGCAGCACACTCGAGCGATG
WT_an_558_f(1>725)   →   GTACCCAGGAAGCTCGCAGGGGGATGTGCGGGTGAAGGGGTAGTGCTTGGTGGGGTCGGCATAGAAGGCAGCACACTCGAGCGATG
1744_558_1_f(1>909)  →   GTACCCAGGAAGCTCGCAGGGGGATGTGCGGGTGAAGGGGTAGTGCTTGGTGGGGTCGGCATAGAAGGCAGCACACTCGAGCGATG
1744_582_2_r(1>731)  ←   GTACCCAGGAAGCTCGCAGGGGGATGTGCGGGTGAAGGGGTAGTGCTTGGTGGGGTCGGCATAGAAGGCAGCACACTCGAGCGATG

                              610       620       630       640       650       660       670       680
                         GCCGCTAGTCGGGGAACCTAACGCCGAGGTCGCGCTCGTCCGATTCTCCAACTGCAGCCGACGAGGGAGTGGAACAGGCGATGGAG
PH207(1>13023)       →   gccgctagtcggggaacctaacgccgaggtcgcgctcgtccgattctccaactgcagccgacgagggagtggaacaggcgatggag
B73(1>13011)         →   accgctagtcgggaaacctaacaccgaggtcgcggttgtctgattctccaactgcagccgacgagggagtggaacaggcggtggag
WT_582_2_r(1>892)    ←   GCCGCTAGTCGGGGAACCTAACGCCGAGGTCGCGCTCGTCCGATTCTCCAACTGCAGCCGACGAGGGAGTGGAACAGGCGATGGAG
WT_an_582_r(1>943)   ←   GCCGCTAGTCGGGGAACCTAACGCCGAGGTCGCGCTCGTCCGATTCTCCAACTGCAGCCGACGAGGGAGTGGAACAGGCGATGGAG
1744_582_r(1>934)    ←   GCCGCTAGTCGGGGAACCTAACGCCGAGGTCGCGCTCGTCCGATTCTCCAACTGCAGCCGACGAGGGAGTGGAACAGGCGATGGAG
1744_558_f(1>933)    →   GCCGCTAGTCGGGGAACCTAACGCCGAGGTCGCGCTCGTCCGATTCTCCAACTGCAGCCGACGAGGGAGTGGAACAGGCGATGGAG
WT_558_1_f(1>924)    →   GCCGCTAGTCGGGGAACCTAACGCCGAGGTCGCGCTCGTCCGATTCTCCAACTGCAGCCGACGAGGGAGTGGAACAGGCGATGGAG
WT_an_558_f(1>725)   →   GCCGCTAGTCGGGGAACCTAACGCCGAGGTCGCGCTCGTCCGATTCTCCAACTGCAGCCGACGAGGGAGTGGAACAGGCGATGGAG
1744_558_1_f(1>909)  →   GCCGCTAGTCGGGGAACCTAACGCCGAGGTCGCGCTCGTCCGATTCTCCAACTGCAGCCGACGAGGGAGTGGAACAGGCGATGGAG
1744_582_2_r(1>731)  ←   GCCGCTAGTCGGGGAACCTAACGCCGAGGTCGCGCTCGTCCGATTCTCCAACTGCAGCCGACGAGGGAGTGGAACAGGCGATGGAG

                         690       700       710       720       730       740       750       760       770
                         AAAGGGGAGAGAGGCGCACAGACATGGGAGAGAAGGAAAGATATGGAAGGATGACTCACAGGAGAAAAGGTGGCTGACGAAGATGG
PH207(1>13023)       →   aaaggggagagaggcgcacagacatgggagagaaggaaagatatggaaggatgactcacaggagaaaaggtggctgacgaagatgg
B73(1>13011)         →   aaaggggagagaggcgcacagacatgggagagaaggaaagatagggaaggatgactcacaggagaaaaggtggctgacgaagatgg
WT_582_2_r(1>892)    ←   AAAGGGGAGAGAGGCGCACAGACATGGGAGAGAAGGAAAGATATGGAAGGATGACTCACAGGAGAAAAGGTGGCTGACGAAGATGG
WT_an_582_r(1>943)   ←   AAAGGGGAGAGAGGCGCACAGACATGGGAGAGAAGGAAAGATATGGAAGGATGACTCACAGGAGAAAAGGTGGCTGACGAAGATGG
1744_582_r(1>934)    ←   AAAGGGGAGAGAGGCGCACAGACATGGGAGAGAAGGAAAGATATGGAAGGATGACTCACAGGAGAAAAGGTGGCTGACGAAGATGG
1744_558_f(1>933)    →   AAAGGGGAGAGAGGCGCACAGACATGGGAGAGAAGGAAAGATATGGAAGGATGACTCACAGGAGAAAAGGTGGCTGACGAAGATGG
WT_558_1_f(1>924)    →   AAAGGGGAGAGAGGCGCACAGACATGGGAGAGAAGGAAAGATATGGAAGGATGACTCACAGGAGAAAAGGTGGCTGACGAAGATGG
WT_an_558_f(1>725)   →   AAAGGGGAGAGAGGCGCACAGACATGGGAGAGAAGGAAAGATATGGAAGGATGACTCACAGGAGAAAAGGTGGCTGACGAAGATGG
1744_558_1_f(1>909)  →   AAAGGGGAGAGAGGCGCACAGACATGGGAGAGAAGGAAAGATATGGAAGGATGACTCACAGGAGAAAAGGTGGCTGACGAAGATGG
1744_582_2_r(1>731)  ←   AAAGGGGAGAGAGGCGCACAGACATGGGAGAGAAGGAAAGATATGGAAGGATGACTCACAGGAGAAAAGGTGGCTGACGAAGATGG

                             780       790       800       810       820       830       840       850       860
                         CGGCGATCGGTGGTGGAAGGGACGCATTGCCGTTGTGAATCGCGCTGCCGTCGTCGCTTCCACACCCATGCGTGTCTAGGGTGAAG
PH207(1>13023)       →   cggcgatcggtggtggaagggacgcattgccgttgtgaatcgcgctgccgtcgtcgcttccacacccatgcgtgtctagggtgaag
B73(1>13011)         →   cggcgatcggtggtggaagggacgcattgccgttgtgaatcgcgctgccgtcgtcgctttcacacccatgcgtgtctagggtgaag
WT_582_2_r(1>892)    ←   CGGCGATCGGTGGTGGAAGGGACGCATTGCCGTTGTGAATCGCGCTGCCGTCGTCGCTTCCACACCCATGCGTGTCTAGGGTGAAG
WT_an_582_r(1>943)   ←   CGGCGATCGGTGGTGGAAGGGACGCATTGCCGTTGTGAATCGCGCTGCCGTCGTCGCTTCCACACCCATGCGTGTCTAGGGTGAAG
1744_582_r(1>934)    ←   CGGCGATCGGTGGTGGAAGGGACGCATTGCCGTTGTGAATCGCGCTGCCGTCGTCGCTTCCACACCCATGCGTGTCTAGGGTGAAG
1744_558_f(1>933)    →   CGGCGATCGGTGGTGGAAGGGACGCATTGCCGTTGTGAATCGCGCTGCCGTCGTCGCTTCCACACCCATGCGTGTCTAGGGTGAAG
WT_558_1_f(1>924)    →   CGGCGATCGGTGGTGGAAGGGACGCATTGCCGTTGTGAATCGCGCTGCCGTCGTCGCTTCCACACCCATGCGTGTCTAGGGTGAAG
WT_an_558_f(1>725)   →   CGTCGATCGGTGGTGGAAGGGACGCATTGCCGTTGTGAATCGCGCTGCCGTCGTCCCTTCCACACCCATGCGTGTCTAG
1744_558_1_f(1>909)  →   CGGCGATCGGTGGTGGAAGGGACGCATTGCCGTTGTGAATCGCGCTGCCGTCGTCGCTTCCACACCCATGCGTGTCTAGGGTGAAG
1744_582_2_r(1>731)  ←   CGGCGATCGGTGGTGGAAGGGACGCATTGCCGTTGTGAATCGCGCTGCCGTCGTCGCTTCCACACCCATGCGTGTCTAGGGTGAAG

                                870       880       890       900       910       920       930       940
                         TTCCACAACTATTTTTTTTTCGTGGATCGATTCTAGGCCGGGATTGGTGGTTTTCACTTGGATTGGGCCTGGTTTGCAAACACATGT
PH207(1>13023)       →   ttccacaactatttttttttcgtggatcgattctaggccgggattggtggttttcacttggattgggcctggtttgcaaacacatgt
B73(1>13011)         →   ttccacagctattttttt-c-tggatcgattctaggccgggattggtggttttcgtttggattgagcgtagtttgcaaacac----
WT_582_2_r(1>892)    ←   TTCCACAACTATTTTTTTTTCGTGGATCGATTCTAGGCCGGGATTGGTGGTTTTCACTTGGATTGGGCCTGGTTTGCAAACACATGT
WT_an_582_r(1>943)   ←   TTCCACAACTATTTTTTTTTCGTGGATCGATTCTAGGCCGGGATTGGTGGTTTTCACTTGGATTGGGCCTGGTTTGCAAACACATGT
1744_582_r(1>934)    ←   TTCCACAACTATTTTTTTTTCGTGGATCGATTCTAGGCCGGGATTGGTGGTTTTCACTTGGATTGGGCCTGGTTTGCAAACACATGT
1744_558_f(1>933)    →   TTCCACAACTATTTTTTTTTCGTGGATCGATTCTAGGCCGGGATTGGTGGTTTTCACTTGGATTGGGCCTGGTTTGCAAACACATGT
WT_558_1_f(1>924)    →   TTCCACAACTATTTTTTTTTCGTGGATCGATTCTAGGCCGGGATTGGTGGTTTTCACTTGGATTGGGCCTGGTTTGCAAACACATGT
1744_558_1_f(1>909)  →   TTCCACAACTATTTTTTTTTCGTGGATCGATTCTAGGCCGGGATTGGTGGTTTTCACTTGGATTGGGCCTGGTTTGCAAACACATGT
1744_582_2_r(1>731)  ←   TTCCACAACTATTTTTTTTTCGTGGATCGATTCTAGGCC
```

```
                         950        960        970        980        990        1000       1010       1020       1030
                         ATGTGGGCTAAATGAACAACCATTTAATCACGGGCTGGTATTAGATTCACAGCGGCA-TGATA-CCTATGGATCATACAACGATCC
PH207(1>13023)      →    atgtgggctaaatgaacaaccatttaatcacgggctggtattagattcacagcggca-tgata-cctatggatcatacaacgatcc
B73(1>13011)        →    atgtgggctaaatgaacaaccatttgatcacgggctggtattagattcacagcggcaatgata-cctatggatcatacaacgatcc
WT_582_2_r(1>892)   ←    A
WT_an_582_r(1>943)  ←    ATGTGGGCTAAATGAACAACCATTTAATCACGGGCTGGTATTAGATTCACAGCGGCA-TGATATCCTATGGATCATACAAC
1744_582_r(1>934)   ←    ATGTGGGCTAAATGAACAACCATTTAATCACGGGCTGGTATTAGATTCACAGCGGCA-TGATA-CCTATGGATCATACAAC
1744_558_f(1>933)   →    ATGTGGGCTAAATGAACAACCATTTAATCACGGGCTGGTATTAGATTCACAGCGGCA-TGATA-CCTATGGATCATACAACGATCC
WT_558_1_f(1>924)   →    ATGTGGGCTAAATGAACAACCATTTAATCACGGGCTGGTATTAGATTCACAGCGGCA-TGATA-CCTATGGATCATACAACGATCC
1744_558_1_f(1>909) →    ATGTGGGCTAAATGAACAACCATTTAATCACGGGCTGGTATTAGATTCACAGCGGCA-TGATA-CCTATGGATCATACAACGATCC

                         1040       1050       1060       1070       1080       1090       1100       1110
                         CCGCCGTTTCAGACCCAACCGAACATGGCCTAAGGAGTATAGCTTGAATGTGTTTGGGGTTGAGACTGTTTATATTTTGCGGGTGA
PH207(1>13023)      →    ccgccgtttcagacccaaccgaacatggcctaaggagtatagcttgaatgtgtttggggttgagactgtttatattttgcgggtga
B73(1>13011)        →    ccgtcgttt-agacccaaccgaacatggcctaaggagtatagcttgaatgtgtttggggttgagactgtttatattttgcgggtga
1744_558_f(1>933)   →    CCGCCGTTTCAGACCCAACCGAACATGGCCT
WT_558_1_f(1>924)   →    CCGCCGTTTCAGACCCAACCGA
1744_558_1_f(1>909) →    CCGCCGTTTCAGACCCAACCGAACATGGC

                         1120       1130       1140       1150       1160       1170       1180       1190       1200
                         GCCCTAGAATTCTTTTAAAAAAATAACGTCTGTGCCTCACAGTAGCTGTAGGAGTAGGGTGTTGTATCTCGCGCTTCAATTCGGTC
PH207(1>13023)      →    gccctagaattcttttaaaaaaataacgtctgtgcctcacagtagctgtaggagtagggtgttgtatctcgcgcttcaattcggtc
B73(1>13011)        →    gccctagaattcttttaaaaaaataacgtctgtgcctcacagtagctgtaggagtagggtgttgcatctcgcgcttcaattcggtc
WT_an_573_f(1>600)  →                 TTCTTTTAAAAAAATAACGTCTGTGCCTCACAGTAGCTGTAGGAGTAGGGTGTTGTATCTCGCGCTTCAATTCGGTC

                         1210       1220       1230       1240       1250       1260       1270       1280       1290
                         GACAGCGGCTCCTGTGGTCTCCAGCCTTTACCGCTTTGGCAACTGGTTCTGTTTCAATCAGGTCCATGCCGTATCTTGCTAAAGCG
PH207(1>13023)      →    gacagcggctcctgtggtctccagcctttaccgctttggcaactggttctgtttcaatcaggtccatgccgtatcttgctaaagcg
B73(1>13011)        →    gacagcggctcctgtggtctccagcctttaccgctttggcaactggttctgtttcaatcaggtccatgccgtatcttgctaaagct
WT_an_573_f(1>600)  →    GACAGCGGCTCCTGTGGTCTCCAGCCTTTACCGCTTTGGCAACTGGTTCTGTTTCAATCAGGTCCATGCCGTATCTTGCTAAAGCG

                         1300       1310       1320       1330       1340       1350       1360       1370
                         AAAAATAAATGCGTTCACGGGAACGGCGTGTGTCCGTGTTTCTGCTCTGCTGCATGCAGCTCTCGCCTTTAtTTTTTTCTTTCCAT
PH207(1>13023)      →    aaaaataaatgcgttcacgggaacggcgtgtgtccgtgtttctgctctgctgcatgcagctctcgcctttatttttttctttccat
B73(1>13011)        →    aaaaataaatgcgttcacgggaacggcgtgtgtccgtgtttctgctctgctgcatgcagctctcgcctttta-ttttttctttccat
WT_an_573_f(1>600)  →    AAAAATAAATGCGTTCACGGGAACGGCGTGTGTCCGTGTTTCTGCTCTGCTGCATGCAGCTCTCGCCTTTATTTTTTTCTTTCCAT

                         1380       1390       1400       1410       1420       1430       1440       1450       1460
                         CAAAAGCAACCGATGACCAACGGCCTTACACAGTCTGTCGAGAACTCGAGATTCTCCATCCCCCCAAATGAAAACGAGGTCGTAAA
PH207(1>13023)      →    caaaagcaaccgatgaccaacggccttacacagtctgtcgagaactcgagattctccatccccccaaatgaaaacgaggtcgtaaa
B73(1>13011)        →    caaaagcaaccgatgaccaacggccttacacagtctgtcgagaactcgagattctccatccccccaaatgaaaacgaggtcgtaaa
WT_an_573_f(1>600)  →    CAAAAGCAACCGATGACCAACGGCCTTACACAGTCTGTCGAGAACTCGAGATTCTCCATCCCCCCAAATGAAAACGAGGTCGTAAA

                         1470       1480       1490       1500       1510       1520       1530       1540
                         TCTCGCTTCACGTCGGTAAGTCTAAAAAATCTTAAATTTAACTGAACTTGTTAAAGATATTGTCAACATTTAGATGTTTAGTTAAA
PH207(1>13023)      →    tctcgcttcacgtcggtaagtctaaaaaatcttaaatttaactgaacttgttaaagatattgtcaacatttagatgtttagttaaa
B73(1>13011)        →    tctcgcttcacgtcggtaagtctaaaaaatcttaaatttaactgaacttgttaaagatattgtcaacatttagatgtttagttaaa
WT_an_573_f(1>600)  →    TCTCGCTTCACGTCGGTAAGTCTAAAAAATCTTAAATTTAACTGAACTTGTTAAAGATATTGTCAACATTTAGATGTTTAGTTAAA

                         1550       1560       1570       1580       1590       1600       1610       1620       1630
                         TTTACTATAAAAAAAGTATCTTAAGATACGTCCATTCAGTcaAAAAAAAAACAAAAAGAAAAACTTTAACTATTCGACAAACGAGA
PH207(1>13023)      →    tttactataaaaaaagtatcttaagatacgtccattcagtcaaaaaaaaaacaaaaagaaaaactttaactattcgacaaacgaga
B73(1>13011)        →    tttactataaaaaaagtatcttaagatacgtccattcagt--aaaaaaaaacaaaaagaaaaactttaactattcgacaaacgaga
WT_an_573_f(1>600)  →    TTTACTATAAAAAAAGTATCTTAAGATACGTCCATTCAGTCAAAAAAAAAACTAAAAGAAAAACTTTAACTATTCGACAAACGAGA

                         1640       1650       1660       1670       1680       1690       1700       1710       1720
                         TTTTTTTTTCCCGGGTTGCCGTCCCACTGCACGGACTAGTTGCCAGGCATCTTCCCTGAAACAAAAAGGAAAACACCAGTTCTAGA
PH207(1>13023)      →    tttttttttcccgggttgccgtcccactgcacggactagttgccaggcatcttccctgaaacaaaaaggaaaacaccagttctaga
B73(1>13011)        →    tttttttttcccgggttgccgtcccactgcacggactagttgccaggcatcttccctgaaacaaaaaggaaaacaccagttctagg
WT_an_573_f(1>600)  →    TTTTTTTTTCCCGGGTTGCCGTCCCACTGCACGGACTAGTTGCCAGGCATCTTCCCTGAAACAAAAAGGAAAACACCAGTTCTAGA
```

```
                      1730      1740      1750      1760      1770      1780      1790      1800
                  CACGTACGTACTACGCTTACGTGTATATAAATATTTATCGGCTGTGGACAGATTATAGAGGGCATCTTGTTGCGACGGGGCGACGA
PH207(1>13023)  → cacgtacgtactacgcttacgtgtatataaatatttatcggctgtggacagattatagagggcatcttgttgcgacggggcgacga
B73(1>13011)    → cacgtacgtactacgcttacgtgtatataaatatttatcggctgtggacagattatagagggcatcttgttgcgacggggcgacga
WT_an_573_f(1>600) → CACGTAC
WT_an_583_r(1>667) ←        TACTACGCTTAAGTGTATATAAATATTTATCGGCTGTGGACAGATTATAGAGGGCATCTTGTTGCGACGGGGCGACGA
```

```
                  1810      1820      1830      1840      1850      1860      1870      1880      1890
                  ATGTCCGTCTCCAGCCACCCGTCCGTTCACAAATCACGTACAAAGCTACTTTGTGTTATCAG---------AAAAAACAAAATAGA
PH207(1>13023)  → atgtccgtctccagccacccgtccgttcacaaatcacgtacaaagctactttgtgttatcag---------aaaaaacaaaataga
B73(1>13011)    → atgtccgtctccagccacccgtccgttcgcaaatcacgtacaaagctactttgtgttagcagaaacagcataaaaaacaaaataga
WT_an_583_r(1>667) ← ATGTCCGTCTCCAGCCACCCGTCCGTTCACAAATCACGTACAAAGCTACTTTGTGTTATCAG---------AAAAAACAAAATAGA
```

```
                      1900      1910      1920      1930      1940      1950      1960      1970
                  TTAACTAACAAATTATAAAGCACGACACATAAATTTACTTgAGAAACCCTTCAACGCGAGGAGAAAAATTACGAGCATCAGCCAGa
PH207(1>13023)  → ttaactaacaaattataaagcacgacacataaatttacttgagaaacccttcaacgcgaggagaaaaattacgagcatcagccaga
B73(1>13011)    → tcaactaacaaatctggaagcacgacacataaatttactt-aaaaaccttttcaatgagaaaggaaaaattatgagcaccagccag-
WT_an_583_r(1>667) ← TTAACTAACAAATTATAAAGCACGACACATAAATTTACTTGAGAAACCCTTCAACGCGAGGAGAAAAATTACGAGCATCAGCCAGA
```

```
                 1980      1990      2000      2010      2020      2030      2040      2050      2060
                  cgaaaccTTCATTatataTGAAGTGTTTATGTAACACCGTCCGTAGACGGCGGCTTACCGGAGAAGTAAAAAGACGACGTGCAAGA
PH207(1>13023)  → cgaaaccttcattatatatgaagtgtttatgtaacaccgtccgtagacggcggcttaccggagaagtaaaaagacgacgtgcaaga
B73(1>13011)    → -------ttgatg-----tgaagtgtttatgtaacgtcgttcgtagacggcggcttacaagagaagtaaaaagacgacgtgcgata
WT_an_583_r(1>667) ← CGAAACCTTCATTATATATGAAGTGTTTATGTAACACCGTCCGTAGACGGCGGCTTACCGGAGAAGTAAAAAGACGACGTGCAAGA
```

```
                     2070      2080      2090      2100      2110      2120      2130      2140      2150
                  AACCCTAATTAAGTTCATcacTAATATATACCCAGGTCTTTCGACTCTGAGCCTTCGCTC--CACTC-ACCAGTGCCTCCATATAT
PH207(1>13023)  → aaccctaattaagttcatcactaatatatacccaggtctttcgactctgagccttcgctc--cactc-accagtgcctccatatat
B73(1>13011)    → aattctaattaggttcat---taatatatacctaagttttggacgatgggtatccactctgctcgctaatattgtctctatattc
WT_an_583_r(1>667) ← AACCCTAATTAAGTTCATCACTAATATATACCCAGGTCTTTCGACTCTGAGCCTTCGCTC--CACTC-ACCAGTGCCTCCATATAT
```

```
                      2160      2170      2180      2190      2200      2210      2220      2230
                  AGAATTTAGGTCACAAAATCAACGGTTTGATTGGTTA-G------GTATACTCACGCACGAACGAACGCACGCCGCGAGAAGGATT
PH207(1>13023)  → agaatttaggtcacaaaatcaacggtttgattggtta-g------gtatactcacgcacgaacgaacgcacgccgcgagaaggatt
B73(1>13011)    → agaatttggatcacaaacttaatagtttggttggttatgtatacagtatactcacgcaccaacgcacgcacgccgcgagaaggatt
WT_an_583_r(1>667) ← AGAATTTAGGTCACAAAATCAACGGTTTGATTGGTTA-G------GTATACTCACGCACGAACGAACGCACGCCGCGAGAAGGATT
```

```
                  2240      2250      2260      2270      2280      2290      2300      2310      2320
                  AGCGAAACGCTGGTGGTTTTTTGTCCACTAGCCGGTGCGTCCCCGCGCGGGAATCATTCGGGCCTGCCTCTCTACTCTGCTCCCAG
PH207(1>13023)  → agcgaaacgctggtggttttttgtccactagccggtgcgtccccgcgcgggaatcattcgggcctgcctctctactctgctcccag
B73(1>13011)    → agcgaaacgctggtggttttttgtccactagccggtgcgtccccgcgcgggaatcattcgggcctgcctctctactctgctcccag
WT_an_583_r(1>667) ← AGCGAAACGCTGGTGGTTTTTTGTCCACTAGCCGGTGCGTCCCCGCGCGGGAATCATTCGGGCCTGCCTCTCTACTCTGCTCCCAG
```

```
                      2330      2340      2350      2360      2370      2380      2390      2400
                  CTACTAGTCCCTCACTCTCTTCTCTCAGACTTGTGTGTCTCGTCC--TATATATATACACACGCTACGCTATAGCTGCTCACACAC
PH207(1>13023)  → ctactagtccctcactctcttctctcagacttgtgtgtctcgtcc--tatatatatacacacgctacgctatagctgctcacacac
B73(1>13011)    → ctactagtccctcactcacttctctcagacttgtgtgtctcgtcctatatatatacacacgctacgctatagctgctcacacac
WT_an_583_r(1>667) ← CTACTAGTCCCTCACTCTCTTCTCTCAGACTTGTGTGTCTCGTCC--TATATATATACACACGCTACGCTATAGCTGCTCACACAC
```

```
                 2410      2420      2430      2440      2450      2460      2470      2480      2490
                  ATAGTATCGTCGTCTCTCTCTCCTTCCCACCAACAACGCACGCGTCGAAGAAGGAAATGATTAGYAGCAGCCATACTTGCTCCGCC
PH207(1>13023)  → atagtatcgtcgtctctctctccttcccaccaacaacgcacgcgtcgaagaaggaaatgattagcagcagccatacttgctccgcc
B73(1>13011)    → atagtatcgtcgtctctctctccttcccaccaacaacgcacgcgtcgaagaaggaaatgattagtagcagccatacttgctccgcc
WT_an_583_r(1>667) ← ATAGTATC
```

```
                     2500      2510      2520      2530      2540      2550      2560      2570      2580
                  TATTAATAGCCAGCGCGCGCGTCTTGTTTTGCTCTCTTCTGTTCTGTTTTGCCCTAGATTAGCGGCGGCGTTTTGGCCTCGCGCGA
PH207(1>13023)  → tattaatagccagcgcgcgcgtcttgttttgctctcttctgttctgttttgccctagattagcggcggcgttttggcctcgcgcga
B73(1>13011)    → tattaatagccagcgcgcgcgtcttgttttgctctcttctgttctgttttgccctagattagcggcggcgttttggcctcgcgcga
```

```
                       2590      2600      2610      2620      2630      2640      2650      2660
                      ACGTCTCGTCCTTGCCGTCTCGCGCGCGTGCGTACGTGCCTGCATTGCGATTTGCAATTATTACGATCGCGCGGCGGCGGCGG
PH207(1>13023)   →    acgtctcgtccttgccgtctcgcgcgcgtgcgtacgtgcctgcattgcgatttgcaattattacgatcgcgcggcggcggcgg
B73(1>13011)     →    acgtctcgtccttgccgtctcgcgcgcgtgcgtacgtgcctgcattgcgatttgcaattattacgatcgcgcggcggcggcgg

                   2670      2680      2690      2700      2710      2720      2730      2740      2750
                      CGGCATGAAGCTCCTCTCGCCGGCGGCCGCACCGTCGTCCTCGCCGTTGTTCCCTCCTCGCATCGTCGAAGGTACGTGTACACCGT
PH207(1>13023)   →    cggcatgaagctcctctcgccggcggccgcaccgtcgtcctcgccgttgttccctcctcgcatcgtcgaaggtacgtgtacaccgt
B73(1>13011)     →    cggcatgaagctcctctcgccggcggccgcaccgtcgtcctcgccgttgttccctcctcgcatcgtcgaaggtacgtgtacaccgt

                          2760      2770      2780      2790      2800      2810      2820      2830
                      CGTCAGCAGCTGCTACCTCCGCGGCGCCGGCCAGCCGAGGTTCCATGATGCctatCTATCTATCTATGTATAGTACGTATATGGCG
PH207(1>13023)   →    cgtcagcagctgctacctccgcggcgccggccagccgaggttccatgatgcctatctatctatctatgtatagtacgtatatggcg
B73(1>13011)     →    cgtcagcagctgctacctccgcggcgccggccagccgaggttccatgatgc----ctatctatctatgtatagtacgtatatggcg

                 2840      2850      2860      2870      2880      2890      2900      2910      2920
                      CCGCGCCAGGCCCTTGCCCTTGTCGTCTGCCTGCATGCCTACTACTACAAGCTACTTCCAAATTTCGCATTGTCCTCGGCGCTACA
PH207(1>13023)   →    ccgcgccaggcccttgcccttgtcgtctgcctgcatgcctactactacaagctacttccaaatttcgcattgtcctcggcgctaca
B73(1>13011)     →    ccgcgccaggcccttgcccttgtcgtctgcctgcatgcctactactacaagctacttccaaatttcgcattgtcctcggcgctaca

                     2930      2940      2950      2960      2970      2980      2990      3000      3010
                      CGGCCGGTGGGCAATCAGACAAAGAAACAAACGTGTAAGCAAGATGAAAAATTGTATTTTTGGGTTCGGACAAGCAAGTCGTCGTC
PH207(1>13023)   →    cggccggtgggcaatcagacaaagaaacaaacgtgtaagcaagatgaaaaattgtatttttgggttcggacaagcaagtcgtcgtc
B73(1>13011)     →    cggccggtgggcaatcagacaaagaaacaaacgtgtaagcaagatgaaaaattgtatttttgggttcggacaagcaagtcgtcgtc

                       3020      3030      3040      3050      3060      3070      3080      3090
                      GTCGTCTTAGGGTAGCCACACACACAGGCAGATGGGCAATCAGACAAAGAAACAAACATAAGCAAGATGGAGAGAGGCAGGCAGGC
PH207(1>13023)   →    gtcgtcttagggtagccacacacacaggcagatgggcaatcagacaaagaaacaaacataagcaagatggagagaggcaggcaggc
B73(1>13011)     →    gtcgtcttagggtagccacacacacaggcagatgggcaatcagacaaagaaacaaacataagcaagatggagagaggcaggcaggc

                   3100      3110      3120      3130      3140      3150      3160      3170      3180
                      AGTCAGGCGCTGCTGcTGCTAGTKSTaGcTCTTaGMgTKTGTYKWGKGKSSTGAWKGTMRARWWYCTCAMcGMYRCKTWTgCTTKW
PH207(1>13023)   →    agtcaggcgctgctgctgctagtgctagctctt-gc-tttgttgtgtgtcctgatggtcgagttcctcaccgctgcttttgctttt
B73(1>13011)     →    agtcaggcgctgctgctgctagtgctagctctt-gc-tttgttgtgtgtcctgatggtcgagttcctcaccgctgcttttgctttt
1744_612_f(1>1065)   →                                  GTTGT-G-TCTTAGAGTGTGTCTAGGGGGGTGAATGTAAAAAATCTCAA-GACACGTAT-CTTGA
1744_612_1_f(1>705)  →                        G-TCTTAGTTGT-G-TCTTAGAGTGTGTCTAGGGGGGTGAATGTAAAAAATCTCAA-GACACGTAT-CTTGA

                          3190      3200      3210      3220      3230      3240      3250      3260
                      CtGMWKWCACWgTGYCTKcAGCTSYAKMTtCGWSAcAKSAGRtCYATgCYGtATYSGtCAtCYKTAaTYYRTKGcAAWGYWgCWGc
PH207(1>13023)   →    ctgctttcact-tgcctgcagctgcagct-cgtca-atcaggtccatgccgtatccg-catccgta-tccgtggcaaagcagcagc
B73(1>13011)     →    ctgctttcact-tgcctgcagctgcagct-cgtca-atcaggtccatgccgtatccg-catccgta-tccgtggcaaagcagcagc
1744_612_f(1>1065)   →    C-GAAGACACAGTGTCTT-AGCTCTATATTCGAGACAGGAGA-CTAT-CTG-ATTGGTCA-CTTTAATTTATTG-AATGTT-CTG-
1744_612_1_f(1>705)  →    C-GAAGACACAGTGTCTT-AGCTCTATATTCGAGACAGGAGA-CTAT-CTG-ATTGGTCA-CTTTAATTTATTG-AATGTT-CTG-

                 3270      3280      3290      3300      3310      3320      3330      3340      3350
                      AKYRGKASRAKGARKAKGcGYRRGMgCgACRKGKYYYMGMggCAtGMCtCAggCTcGYMKKRTGgTKGWGWKYKcAGCWGYaGKCY
PH207(1>13023)   →    agcaggaggaggaggaggcgcgggcgcgacggggccccgcggca-gcctcaggctcgccgggtggtggagagcgcagcagcaggcc
B73(1>13011)     →    agcaggaggaggaggaggcgcgggcgcgacggggccccgcggca-gcctcaggctcgccgggtggtggagagcgcagcagcaggcc
1744_612_f(1>1065)   →    ATTGGTACAATGAATATG-GTAAGA-C-ACATGTTTTAGA--CATGAC-CA--CT-GTATTATG-TTGTGTTTT-AGCTGT-GTCT
1744_612_1_f(1>705)  →    ATTGGTACAATGAATATG-GTAAGA-C-ACATGTTTTAGA--CATGAC-CA--CT-GTATTATG-TTGTGTTTT-AGCTGT-GTCT

                     3360      3370      3380      3390      3400      3410      3420      3430      3440
                      YMKgccACKKSGAcGWcAMCGYaGCAGCcTGtAtCWASGTYgTMCAtGYSCTAAAGGTGCTAGCTTGCTCGTTATATTTGATTTGA
PH207(1>13023)   →    ccggccacggcgacgacaacgcagcagcctg-a-caacgtc-tcca-gtgctaaaggtgctagcttgctcgttatatttgatttga
B73(1>13011)     →    ccggccacggcgacgacaacgcagcagcctg-a-caacgtc-tcca-gtgctaaaggtgctagcttgctcgttatatttgatttga
1744_612_f(1>1065)   →    TAT---ACTTGGA-GT-ACCGT-GCAGC-TGTATCTAGGTTGTACATGCCCTAAAGGTGCTAGCTTGCTCGTTATATTTGATTTGA
WT_an_612_f(1>1030)  →                                                                                  TTTGATTTGA
1744_612_1_f(1>705)  →    TAT---ACTTGGA-GT-ACCGT-GCAGC-TGTATCTAGGTTGTACATGCCCTAAAGGTGCTAGCTTGCTCGTTATATTTGATTTGA
WT_612_1_f(1>846)    →                                                                                  TTTGATTTGA
```

```
                 3450      3460      3470      3480      3490      3500      3510      3520
                 CTAGTCTCATCATCCA-CCCCCAGTCACGTACACAGATGCTCTCTCTCTCTCTCTCTCTTGAATTGATGAGCGAACGAAACAC-
PH207(1>13023)       ctagtctcatcatccacccccc-agtcacgtacacagatgctctctctctctctctctcttgaattgatgagcgaacgaaacac-
B73(1>13011)         ctagtctcatcatccaccccccagtcacgtacacagatg--ctctctctctctctctctcttgaattgatgagcgaacgaaacac-
1744_612_f(1>1065)   CTAGTCTCATCATCCA-CCCCCAGTCACGTACACAGATGCTCTCTCTCTCTCTCTCTCTTGAATTGATGAGCGAACGAAACAC-
WT_an_612_f(1>1030)  CTAGTCTCATCATCCA-CCCCCAGTCACGTACACAGATGCTCTCTCTCTCTCTCTCTCTTGAATTGATGAGCGAACGAAACAC-
WT_an_620_r(1>1072)                                                               TGAATTGATGAGCGAACGAAACACT
1744_612_1_f(1>705)  CTAGTCTCATCATCCA-CCCCCAGTCACGTACACAGATGCTCTCTCTCTCTCTCTCTCTTGAATTGATGAGCGAACGAAACAC-
WT_612_1_f(1>846)    CTAGTCTCATCATCCA-CCCCCAGTCACGTACACAGATGCTCTCTCTCTCTCTCTCTCTTGAATTGATGAGCGAACGAAACAC-

                 3530      3540      3550      3560      3570      3580      3590      3600      3610
                 TCAGACAGATGCTGTGCCGTGCTGCAGTGCGCCCGTAGCAGCACAGACACTCTGCCGCACGCACCTGCGCTTGTCGCTTCCCC-TC
PH207(1>13023)       tcagacagatgctgtgccgtgctgcagtgcgcccgtagcagcacagacactctgccgcacgcacctgcgcttgtcgcttcccc-tc
B73(1>13011)         tcagacagatgctgtgccgtgctgcagtgcgcccgtagcagcacagacactctgccgcacgcacctgcgcttgtcgcttcccc-tc
1744_612_f(1>1065)   TCAGACAGATGCTGTGCCGTGCTGCAGTGCGCCCGTAGCAGCACAGACACTCTGCCGCACGCACCTGCGCTTGTCGCTTCCCC-TC
WT_an_612_f(1>1030)  TCAGACAGATGCTGTGCCGTGCTGCAGTGCGCCCGTAGCAGCACAGACACTCTGCCGCACGCACCTGCGCTTGTCGCTTCCCC-TC
WT_an_620_r(1>1072)  TCAGACAGATGCTGTGCCGTGCTGCAGTGCGCCCGTAGCAGCACAGACATTCTGCCGCACGCACCTGCGCTTGTCGCTTCCCC-TC
1744_612_1_f(1>705)  TCAGACAGATGCTGTGCCGTGCTGCAGTGCGCCCGTAGCAGCACAGACACTCTGCCGCACGCACCTGCGCTTGTCGCTTCCCC-TC
WT_612_1_f(1>846)    TCAGACAGATGCTGTGCCGTGCTGCAGTGCGCCCGTAGCAGCACAGACACTCTGCCGCACGCACCTGCGCTTGTCGCTTCCCC-TC
1744_620_r(1>970)                                                                           TGTCGCTTCCCCTTC

                 3620      3630      3640      3650      3660      3670      3680      3690
                 -TTGCTATATCTCCTGCTGCTTTTGCTAAAGCCGGAAACCAAAAAG-AAAGTTGAGCTTTTCGTCACAATTTTGCTTTACTTATTA
PH207(1>13023)       -ttgctatatctcctgctgcttttgctaaagccggaaaccaaaaag-aaagttgagcttttcgtcacaattttgctttacttatta
B73(1>13011)         -ttgctatatctcctgctgcttttgctaaagccggaaaccaaaaag-aaagttgagcttttcgtcacaattttgctttacttatta
1744_612_f(1>1065)   -TTGCTATATCTCCTGCTGCTTTTGCTAAAGCCGGAAACCAAAAAG-AAAGTTGAGCTTTTCGTCACAATTTTGCTTTACTTATTA
WT_an_612_f(1>1030)  -TTGCTATATCTCCTGCTGCTTTTGCTAAAGCCGGAAACCAAAAAG-AAAGTTGAGCTTTTCGTCACAATTTTGCTTTACTTATTA
WT_an_620_r(1>1072)  TTTGCTATATCTCCTGCTGCTTTTGCTAAAGCCGGAAACCAAAAAG-AAAGTTGAGCTTTTCGTCACAATTTTGCTTTACTTATTA
1744_612_1_f(1>705)  -TTGCTATATCTCCTGCTGCTTTTGCTAAAGCCGGAAACCAAAAAG-AAAGTTGAGCTTTTCGTCACAATTTTGCTTTACTTATTA
WT_612_1_f(1>846)    -TTGCTATATCTCCTGCTGCTTTTGCTAAAGCCGGAAACCAAAAAG-AAAGTTGAGCTTTTCGTCACAATTTTGCTTTACTTATTA
1744_620_r(1>970)    -TTGCTATATCTCCTGCTGCTTTTGCTAAAGCCGGAAACCAAAAAGAAAAGTTGAGCTTTTCGTCACAATTTTGCTTTACTTATTA

                 3700      3710      3720      3730      3740      3750      3760      3770      3780
                 AGTGCTAGTCCGTTTTGTTTATACGACCTTACTATTAGCTTCTAGCCCGGATTCAAAGTGCTACAGTGCGTTTTCACTAACAACAT
PH207(1>13023)       agtgctagtccgttttgtttatacgaccttactattagcttctagcccggattcaaagtgctacagtgcgttttcactaacaacat
B73(1>13011)         agtgctagtccgttttgtttatacgaccttactattagcttctagcccggattcaaagtgctacagtgcgttttcactaacaacat
1744_612_f(1>1065)   AGTGCTAGTCCGTTTTGTTTATACGACCTTACTATTAGCTTCTAGCCCGGATTCAAAGTGCTACAGTGCGTTTTCACTAACAACAT
WT_an_612_f(1>1030)  AGTGCTAGTCCGTTTTGTTTATACGACCTTACTATTAGCTTCTAGCCCGGATTCAAAGTGCTACAGTGCGTTTTCACTAACAACAT
WT_an_620_r(1>1072)  AGTGCTAGTCCGTTTTGTTTATACGACCTTACTATTAGCTTCTAGCCCGGATTCAAAGTGCTACAGTGCGTTTTCACTAACAACAT
1744_612_1_f(1>705)  AGTGCTAGTCCGTTTTGTTTATACGACCTTACTATTAGCTTCTAGCCCGGATTCAAAGTGCTACAGTGCGTTTTCACTAACAACAT
WT_612_1_f(1>846)    AGTGCTAGTCCGTTTTGTTTATACGACCTTACTATTAGCTTCTAGCCCGGATTCAAAGTGCTACAGTGCGTTTTCACTAACAACAT
1744_620_r(1>970)    AGTGCTAGTCCGTTTTGTTTATACGACCTTACTATTAGCTTCTAGCCCGGATTCAAAGTGCTACAGTGCGTTTTCACTAACAACAT
1744_620_2_r(1>805)                                                                           TCACTAACAACAT

                 3790      3800      3810      3820      3830      3840      3850      3860      3870
                 TTAGGATTATAGCACATCAAATTTGACTAGTGTTACAAAAATATATATTAGCATCATACATATTTAGTTAAAGTTAAAAATATTTA
PH207(1>13023)       ttaggattatagcacatcaaatttgactagtgttacaaaaatatatattagcatcatacatatttagttaaagttaaaaatattta
B73(1>13011)         ttaggattatagcacatcaaatttgactagtgttacaaaaatatatattagcatcatacatatttagttaaagttaaaaatattta
1744_612_f(1>1065)   TTAGGATTATAGCACATCAAATTTGACTAGTGTTACAAAAATATATATTAGCATCATACATATTTAGTTAAAGTTAAAAATATTTA
WT_an_612_f(1>1030)  TTAGGATTATAGCACATCAAATTTGACTAGTGTTACAAAAATATATATTAGCATCATACATATTTAGTTAAAGTTAAAAATATTTA
WT_an_620_r(1>1072)  TTAGGATTATAGCACATCAAATTTGACTAGTGTTACAAAAATATATATTAGCATCATACATATTTAGTTAAAGTTAAAAATATTTA
1744_612_1_f(1>705)  TTAGGATTATAGCACATCAAATTTGACTAGTGTTACAAAAATATATATTAGCATCATACATATTTAGTT
WT_612_1_f(1>846)    TTAGGATTATAGCACATCAAATTTGACTAGTGTTACAAAAATATATATTAGCATCATACATATTTAGTTAAAGTTAAAAATATTTA
1744_620_r(1>970)    TTAGGATTATAGCACATCAAATTTGACTAGTGTTACAAAAATATATATTAGCATCATACATATTTAGTTAAAGTTAAAAATATTTA
1744_620_2_r(1>805)  TTAGGATTATAGCACATCAAATCTGACTAGTGTTACAAAAATATATATTAGCATCATACATATTTAGTTAAAGTTAAAAATATTTA
WT_620_2_r(1>762)                                  TGTTACAAAAATATATATTAGCATCATACATACCTAGTTAAAGTTAAAAATATTTA

                 3880      3890      3900      3910      3920      3930      3940      3950
                 ATTTGTATGAAAAACAAGAGTGACACTCTCTATGGGATAGGCCGAGTACGCACCATGCATGATTACTACTATTAGGCTATCTCCAG
PH207(1>13023)       atttgtatgaaaaacaagagtgacactctctatgggataggccgagtacgcaccatgcatgattactactattaggctatctccag
B73(1>13011)         atttgtatgaaaaacaagagtgacactctctatgggataggccgagtacgcaccatgcatgattactactattaggctatctccag
1744_612_f(1>1065)   ATTTGTATGAAAAACAAGAGTGACACTCTCTATGGGATAGGCCGAGTACGCACCATGCATGATTACTACTATTAGGCTATCTCCAG
WT_an_612_f(1>1030)  ATTTGTATGAAAAACAAGAGTGACACTCTCTATGGGATAGGCCGAGTACGCACCATGCATGATTACTACTATTAGGCTATCTCCAG
WT_an_620_r(1>1072)  ATTTGTATGAAAAACAAGAGTGACACTCTCTATGGGATAGGCCGAGTACGCACCATGCATGATTACTACTATTAGGCTATCTCCAG
WT_612_1_f(1>846)    ATTTGTATGAAAAACAAGAGTGACACTCTCTATGGGATAGGCCGAGTACGCACCATGCATGATTACTACTATTAGGCTATCTCCAG
1744_620_r(1>970)    ATTTGTATGAAAAACAAGAGTGACACTCTCTATGGGATAGGCCGAGTACGCACCATGCATGATTACTACTATTAGGCTATCTCCAG
1744_620_2_r(1>805)  ATTTGTATGAAAAACAAGAGTGACACTCTCTATGGGATAGGCCGAGTACGCACCATGCATGATTACTACTATTAGGCTATCTCCAG
WT_620_2_r(1>762)    ATTTGTATGAAAAACAAGAGTGACACTCTCTATGGGATAGGCCGAGTACGCACCATGCATGATTACTACTATTAGGCTATCTCCAG
```

```
                   3960      3970      3980      3990      4000      4010      4020      4030      4040
                   CAGCGTTACCTATTCTACCTTCTATTTCAAACTTTACTTCGTAAACAGTATAATTTGTAATGCAAAACAATGTTTTATACGGTCAT

PH207(1>13023)     cagcgttacctattctaccttctatttcaaactttacttcgtaaacagtataatttgtaatgcaaaacaatgttttatacggtcat
B73(1>13011)       cagcgttacctattctaccttctatttcaaactttacttcgtaaacagtataatttgtaatgcaaaacaatgttttatacggtcat
1744_612_f(1>1065) CAGCGTTACCTATTCTACCTTCTATTTCAAACTTTACTTCGTAAACAGTATAATTTGTAATGCAAAACAATGTTTTATACGGTCAT
WT_an_612_f(1>1030) CAGCGTTACCTATTCTACCTTCTATTTCAAACTTTACTTCGTAAACAGTATAATTTGTAATGCAAAACAATGTTTTATACGGTCAT
WT_an_620_r(1>1072) CAGCGTTACCTATTCTACCTTCTATTTCAAACTTTACTTCGTAAACAGTATAATTTGTAATGCAAAACAATGTTTTATACGGTCAT
WT_612_1_f(1>846)  CAGCGTTACCTATTCTACCTTCTATTTCAAACTTTACTTCGTAAACAGTATAATTTGTAATGCAAAACAATGTTTTATACGGTCAT
1744_620_r(1>970)  CAGCGTTACCTATTCTACCTTCTATTTCAAACTTTACTTCGTAAACAGTATAATTTGTAATGCAAAACAATGTTTTATACGGTCAT
1744_620_2_r(1>805) CAGCGTTACCTATTCTACCTTCTATTTCAAACTTTACTTCGTAAACAGTATAATTTGTAATGCAAAACAATGTTTTATACGGTCAT
WT_620_2_r(1>762)  CAGCGTTACCTATTCTACCTTCTATTTCAAACTTTACTTCGTAAACAGTATAATTTGTAATGCAAAACAATGTTTTATACGGTCAT

                       4050      4060      4070      4080      4090      4100      4110      4120
                   ATACACGGTTTGCTAGCCACAGCCTAAGGCCAACTCCAATAGACTGTTCTGTACTGCAGACGACAATGTTTACATAGTAAAGTTTG

PH207(1>13023)     atacacggtttgctagccacagcctaaggccaactccaatagactgttctgtactgcagacgacaatgtttacatagtaaagtttg
B73(1>13011)       atacacggttcgctagccacagcctaaggccaactccaatagactgttctgtactgcagacgacaatgtttacatagtaaagtttg
1744_612_f(1>1065) ATACACGGTTTGCTAGCCACAGCCTAAGGCCAACTCCAATAGACTGTTCTGTACTGCAGACGACAATGTTTACATAGTAAAGTTTG
WT_an_612_f(1>1030) ATACACGGTTTGCTAGCCACAGCCTAAGGCCAACTCCAATAGACTGTTCTGTACTGCAGACGACAATGTTTACATAGTAAAGTTTG
WT_an_620_r(1>1072) ATACACGGTTTGCTAGCCACAGCCTAAGGCCAACTCCAATAGACTGTTCTGTACTGCAGACGACAATGTTTACATAGTAAAGTTTG
WT_612_1_f(1>846)  ATACACGGTTTGCTAGCCACAGCCTAAGGCCAACTCCAATAGACTGTTCTGTACTGCAGACGACAATGTTTACATAGTAAAGTTTG
1744_620_r(1>970)  ATACACGGTTTGCTAGCCACAGCCTAAGGCCAACTCCAATAGACTGTTCTGTACTGCAGACGACAATGTTTACATAGTAAAGTTTG
1744_620_2_r(1>805) ATACACGGTTTGCTAGCCACAGCCTAAGGCCAACTCCAATAGACTGTTCTGTACTGCAGACGACAATGTTTACATAGTAAAGTTTG
WT_620_2_r(1>762)  ATACACGGTTTGCTAGCCACAGCCTAAGGCCAACTCCAATAGACTGTTCTGTACTGCAGACGACAATGTTTACATAGTAAAGTTTG

                   4130      4140      4150      4160      4170      4180      4190      4200      4210
                   AAATACTGATTGAAGTACGTAGTTTGCTGGAGATAGCATAAGCCTCCTTGATAAGGCTCTTTCAAAGGCTCCTCCAACAACTCCTT

PH207(1>13023)     aaatactgattgaagtacgtagtttgctggagatagcataagcctccttgataaggctctttcaaaggctcctccaacaactcctt
B73(1>13011)       aaatactgattgaagtacgtagtttgctggagatagcataagcctccttgataaggctctttcaaaggctcctccaacaactcctt
1744_612_f(1>1065) AAATACTGATTGAAGTACGTAGTTTGCTGGAAATAGCATAAGCCTCCTTGAAAAGGCTCTTTCAAAGGCTCCTCCAACAACTCCTT
WT_an_612_f(1>1030) AAATACTGATTGAAGTACGTAGTTTGCTGGAGATAGCATAAGCCTCCTTGATAAGGCTCTTTCAAAGGCTCCTCCAACAACTCCTT
WT_an_620_r(1>1072) AAATACTGATTGAAGTACGTAGTTTGCTGGAGATAGCATAAGCCTCCTTGATAAGGCTCTTTCAAAGGCTCCTCCAACAACTCCTT
WT_an_576_f(1>1142)                                                                                 CTCCTT
1744_576_f(1>1170)                                                                                   TCCTT
WT_612_1_f(1>846)  AAATACTGATTGAAGTACGTAGTTTGCTGGAGATAGCATAAGCCTCCTTGATAAGGCTCTTTCAAAGGCTCCTCCAACAACTCCTT
1744_620_r(1>970)  AAATACTGATTGAAGTACGTAGTTTGCTGGAGATAGCATAAGCCTCCTTGATAAGGCTCTTTCAAAGGCTCCTCCAACAACTCCTT
1744_620_2_r(1>805) AAATACTGATTGAAGTACGTAGTTTGCTGGAGATAGCATAAGCCTCCTTGATAAGGCTCTTTCAAAGGCTCCTCCAACAACTCCTT
WT_620_2_r(1>762)  AAATACTGATTGAAGTACGTAGTTTGCTGGAGATAGCATAAGCCTCCTTGATAAGGCTCTTTCAAAGGCTCCTCCAACAACTCCTT

                     4220      4230      4240      4250      4260      4270      4280      4290      4300
                   AAAAAATCTATGAAACGGGGGCTATTCCGCTAGCTTCTCGTTGAAAATAGCAAGGAGCTGCTTCCAAGAAACGAGCAAAGTCGGGG

PH207(1>13023)     aaaaaatctatgaaacgggggctattccgctagcttctcgttgaaaatagcaaggagctgcttccaagaaacgagcaaagtcgggg
B73(1>13011)       aaaaaatctatgaaacgggggctattccgctagcttctcgttgaaaatagcaaggagctgcttccaagaaacgagcaaagtcgggg
1744_612_f(1>1065) AAAAA
WT_an_612_f(1>1030) AAAAAATCTATGAAACGGGGGCTATTCCGCTAGCTTCTCGTTGAAAATAGCAAGGAGCTGCTTCCAAGAAACGAGCAAAGTCGGGG
WT_an_620_r(1>1072) AAAAAATCTATGAAACGGGGGCTATTCCGCTAGCTTCTCGTTGAAAATAGCAAGGAGCTGCTTCCAAGAAACGAGCAAAGTCGGGG
WT_an_576_f(1>1142) -AAAAATCTATGAAACGGGGGCTATTCCGCTAGCTTCTCGTTGAAAATAGCAAGGAGCTGCTTCCAAGAAACGAGCAAAGTCGGGG
1744_576_f(1>1170) -AAAAATCTATGAAACGGGGGCTATTCCGCTAGCTTCTCGTTGAAAATAGCAAGGAGCTGCTTCCAAGAAACGAGCAAAGTCGGGG
WT_612_1_f(1>846)  AAAAAATCTATGAAACGGGGGCTATTCCGCTAGCTTCTCGTTGAAAATAGCAAGGAGCTGCTTCCAA
1744_620_r(1>970)  AAAAAATCTATGAAACGGGGGCTATTCCGCTAGCTTCTCGTTGAAAATAGCAAGGAGCTGCTTCCAAGAAACGAGCAAAGTCGGGG
1744_620_2_r(1>805) AAAAAATCTATGAAACGGGGGCTATTCCGCTAGCTTCTCGTTGAAAATAGCAAGGAGCTGCTTCCAAGAAACGAGCAAAGTCGGGG
WT_620_2_r(1>762)  AAAAAATCTATGAAACGGGGGCTATTCCGCTAGCTTCTCGTTGAAAATAGCAAGGAGCTGCTTCCAAGAAACGAGCAAAGTCGGGG

                         4310      4320      4330      4340      4350      4360      4370      4380
                   GCTCGAAAAGAGGCTTCTAAGGCTCCCTCCCATCCTCCATTCCTCCCCTCTCTCATGGGTTCCATGTTGGCTGAAGGTGCTGCATT

PH207(1>13023)     gctcgaaaagaggcttctaaggctccctcccatcctccattcctcccctctctcatgggttccatgttggctgaaggtgctgcatt
B73(1>13011)       gctcgaaaagaggcttctaaggctccctcccatcctccattcctcccctctctcatgggttccatgttggctgaaggtgctgcatt
WT_an_612_f(1>1030) GCTCGAAAAGAGGCTTCTAAGGCTCCCTCCCATCCTCCATTCCTCCCCTCTCTCATGGGTTCCATGTTGGCTGAAGGTGCTGCATT
WT_an_620_r(1>1072) GCTCGAAAAGAGGCTTCTAAGGCTCCCTCCCATCCTCCATTCCTCCCCTCTCTCATGGGTTCCATGTTGGCTGAAGGTGCTGCATT
WT_an_576_f(1>1142) GCTCGAAAAGAGGCTTCTAAGGCTCCCTCCCATCCTCCATTCCTCCCCTCTCTCATGGGTTCCATGTTGGCTGAAGGTGCTGCATT
1744_576_f(1>1170) GCTCGAAAAGAGGCTTCTAAGGCTCCCTCCCATCCTCCATTCCTCCCCTCTCTCATGGGTTCCATGTTGGCTGAAGGTGCTGCATT
1744_620_r(1>970)  GCTCGAAAAGAGGCTTCTAAGGCTCCCTCCCATCCTCCATTCCTCCCCTCTCTCATGGGTTCCATGTTGGCTGAAGGTGCTGCATT
1744_620_2_r(1>805) GCTCGAAAAGAGGCTTCTAAGGCTCCCTCCCATCCTCCATTCCTCCCCTCTCTCATGGGTTCCATGTTGGCTGAAGGTGCTGCATT
WT_620_2_r(1>762)  GCTCGAAAAGAGGCTTCTAAGGCTCCCTCCCATCCTCCATTCCTCCCCTCTCTCATGGGTTCCATGTTGGCTGAAGGTGCTGCATT

                   4390      4400      4410      4420      4430      4440      4450      4460      4470
                   TCGTACTAAATAGGGTGCTCATCCCGAAACTTTTTGTGAAAACAAGAACATAGCTATTTTCACACAAGAGCCAAAACCAGAGTTGG

PH207(1>13023)     tcgtactaaatagggtgctcatcccgaaactttttgtgaaaacaagaacatagctattttcacacaagagccaaaaccagagttgg
B73(1>13011)       tcgtactaaatagggtgctcatcccgaaactttttgtgaaaacaagaacatagctattttcacacaagagccaaaaccagagttgg
WT_an_612_f(1>1030) TCGTACTAAATAGGGTGCTCATCCCGAAACTTTTTGTGAAAACAAGAACATAGCTATTTTCACACAAGAGCCAAAACCA
```

```
                          4390      4400      4410      4420      4430      4440      4450      4460      4470
                          TCGTACTAAATAGGGTGCTCATCCCGAAACTTTTTGTGAAAACAAGAACATAGCTATTTTCACACAAGAGCCAAAACCAGAGTTGG
WT_an_620_r(1>1072)   ←   TCGTACTAAATAGGGTGCTCATCCCGAAACTTTTTGTGAAAACAAGAACATAGCTATTTTCACACAAGAGCCAAAACCAGAGTTGG
WT_an_576_f(1>1142)   →   TCGTACTAAATAGGGTGCTCATCCCGAAACTTTTTGTGAAAACAAGAACATAGCTATTTTCACACAAGAGCCAAAACCAGAGTTGG
1744_576_f(1>1170)    →   TCGTACTAAATAGGGTGCTCATCCCGAAACTTTTTGTGAAAACAAGAACATAGCTATTTTCACACAAGAGCCAAAACCAGAGTTGG
1744_620_r(1>970)     ←   TCGTACTAAATAGGGTGCTCATCCCGAAACTTTTTGTGAAAACAAGAACATAGCTATTTTCACACAAGAGCCAAAACCAGAGTTGG
1744_620_2_r(1>805)   ←   TCGTACTAAATAGGGTGCTCATCCCGAAACTTTTTGTGAAAACAAGAACATAGCTATTTTCACACAAGAGCCAAAACCAGAGTTGG
WT_620_2_r(1>762)     ←   TCGTACTAAATAGGGTGCTCATCCCGAAACTTTTTGTGAAAACAAGAACATAGCTATTTTCACACAAGAGCCAAAACCAGAGTTGG
```

```
                              4480      4490      4500      4510      4520      4530      4540      4550
                          AGTTGTTGACACAAAAGTCTAACTAAAGGCGGGCAAACATGCATACCTGGTATTCCGCATTGTGTTGGTCTTGCATATATTCAATT
PH207(1>13023)        →   agttgttgacacaaaagtctaactaaaggcgggcaaacatgcatacctggtattccgcattgtgttggtcttgcatatattcaatt
B73(1>13011)          →   agttgttgacacaaaagtctaactaaaggcgggcaaacatgcatacctggtattccgcggtgtgttggtcttgcatatattcaatt
WT_an_620_r(1>1072)   ←   AGTTGTTGACACAAAAGTCTAACTAAAGGCGGGCAAACATGCATACCTGGTATTCCGCATTGTGTTGGTCTTGCATATATTCAATT
WT_an_576_f(1>1142)   →   AGTTGTTGACACAAAAGTCTAACTAAAGGCGGGCAAACATGCATACCTGGTATTCCGCATTGTGTTGGTCTTGCATATATTCAATT
1744_576_f(1>1170)    →   AGTTGTTGACACAAAAGTCTAACTAAAGGCGGGCAAACATGCATACCTGGTATTCCGCATTGTGTTGGTCTTGCATATATTCAATT
WT_an_586_r(1>1140)   ←                       AACTAAAGGCGGGCAAACATGCATACCTGGTATTCCGCATTGTGTTGGTCTTGCATATATTCAATT
1744_586_r(1>1139)    ←                       AACTAAAGGCGGGCAAACATGCATACCTGGTATTCCGCATTG-GTTGGTCTTGCATATATTCAATT
1744_620_r(1>970)     ←   AGTTGTTGACACAAAAGTCTAACTAAAGGCGGGCAAACATGCATACCTGGTATTCCGCATTGTGTTGGTCTTGCATATATTCAATT
1744_620_2_r(1>805)   ←   AGTTGTTGACACAAAAGTCTAACTAAAGGCGGGCAAACATGCATACCTGGTATTCCGCATTGTGTTGGTCTTGCATATATTCAATT
WT_620_2_r(1>762)     ←   AGTTGTTGACACAAAAGTCTAACTAAAGGCGGGCAAACATGCATACCTGGTATTCCGCATTGTGTTGGTCTTGCATATATTCAATT
```

```
                        4560      4570      4580      4590      4600      4610      4620      4630      4640
                          TTCCTATTAAGTTCACAATGTTTACATTTTATTATTTACTAGGTAGGTGCTCGTGTATGGTTACCGTTTCTATATATCATATAGGT
PH207(1>13023)        →   ttcctattaagttcacaatgtttacattttattatttactaggtaggtgctcgtgtatggttaccgtttctatatatcatataggt
B73(1>13011)          →   ttcctattaagttcacaatgtttacattttattatttactaggtaggtgctcgtgtatggttaccgtttctatatatcatataggt
WT_an_620_r(1>1072)   ←   TTCCTATTAAGT-CACAA
WT_an_576_f(1>1142)   →   TTCCTATTAAGTTCACAATGTTTACATTTTATTATTTACTAGGTAGGTGCTCGTGTATGGTTACCGTTTCTATATATCATATAGGT
1744_576_f(1>1170)    →   TTCCTATTAAGTTCACAATGTTTACATTTTATTATTTACTAGGTAGGTGCTCGTGTATGGTTACCGTTTCTATATATCATATAGGT
WT_an_586_r(1>1140)   ←   TTCCTATTAAGTTCACAATGTTTACATTTTATTATTTACTAGGTAGGTGCTCGTGTATGGTTACCGTTTCTATATATCATATAGGT
1744_586_r(1>1139)    ←   TTCCTATTAAGTTCACAATGTTTACATTTTATTATTTACTAGGTAGGTGCTCGTGTATGGTTACCGTTTCTATATATCATATAGGT
1744_620_r(1>970)     ←   TTCCTATTAA
1744_620_2_r(1>805)   ←   TTCCTATTAAGTTCACAA
WT_620_2_r(1>762)     ←   TTCCTATTAAGTTCACAA
```

```
                            4650      4660      4670      4680      4690      4700      4710      4720      4730
                          AGACCTTGATTAAATGTGATAGTTATTTAGACACAGCTGATCCATTTAATATTGTTACGACCTGATACGACACTATTTGAAATAGG
PH207(1>13023)        →   agaccttgattaaatgtgatagttatttagacacagctgatccatttaatattgttacgacctgatacgacactatttgaaatagg
B73(1>13011)          →   agaccttgattaaatgtgatagttatttagacacagctgatccatttaatattgttacgacctgatacgacactatttgaaatagg
WT_an_576_f(1>1142)   →   AGACCTTGATTAAATGTGATAGTTATTTAGACACAGCTGATCCATTTAATATTGTTACGACCTGATACGACACTATTTGAAATAGG
1744_576_f(1>1170)    →   AGACCTTGATTAAATGTGATAGTTATTTAGACACAGCTGATCCATTTAATATTGTTACGACCTGATACGACACTATTTGAAATAGG
WT_an_586_r(1>1140)   ←   AGACCTTGATTAAATGTGATAGTTATTTAGACACAGCTGATCCATTTAATATTGTTACGACCTGATACGACACTATTTGAAATAGG
1744_586_r(1>1139)    ←   AGACCTTGATTAAATGTGATAGTTATTTAGACACAGCTGATCCATTTAATATTGTTACGACCTGATACGACACTATTTGAAATAGG
1744_613_f(1>1134)    →                 TGTGATAGTTATTTAGACACAGCTGATCCATTTAATATTGTTACGACCTGATACGACACTATTTGAAATAGG
WT_an_613_f(1>1105)   →                       GTTATTTAGACACAGCTGATCCATTTAATATTGTTACGACCTGATACGACACTATTTGAAATAGG
```

```
                                4740      4750      4760      4770      4780      4790      4800      4810
                          CCCATGTTGGACTAGCCCGCAAGTGCCGATCCAGCCTGATACGATTATATTAAGATATATATATTTACAATGTATATCCTTGGTAT
PH207(1>13023)        →   cccatgttggactagcccgcaagtgccgatccagcctgatacgattatattaagatatatatatttacaatgtatatccttggtat
B73(1>13011)          →   cccatgttggactagcccgcaagtgccgatccagcctgatacgattatattaagatatatatatttacaatgtatatccttggtat
WT_an_576_f(1>1142)   →   CCCATGTTGGACTAGCCCGCAAGTGCCGATCCAGCCTGATACGATTATATTAAGATATATATATTTACAATGTATATCCTTGGTAT
1744_576_f(1>1170)    →   CCCATGTTGGACTAGCCCGCAAGTGCCGATCCAGCCTGATACGATTATATTAAGATATATATATTTACAATGTATATCCTTGGTAT
WT_an_586_r(1>1140)   ←   CCCATGTTGGACTAGCCCGCAAGTGCCGATCCAGCCTGATACGATTATATTAAGATATATATATTTACAATGTATATCCTTGGTAT
1744_586_r(1>1139)    ←   CCCATGTTGGACTAGCCCGCAAGTGCCGATCCAGCCTGATACGATTATATTAAGATATATATATTTACAATGTATATCCTTGGTAT
1744_613_f(1>1134)    →   CCCATGTTGGACTAGCCCGCAAGTGCCGATCCAGCCTGATACGATTATATTAAGATATATATATTTACAATGTATATCCTTGGTAT
WT_an_613_f(1>1105)   →   CCCATGTTGGACTAGCCCGCAAGTGCCGATCCAGCCTGATACGATTATATTAAGATATATATATTTACAATGTATATCCTTGGTAT
```

```
                          4820      4830      4840      4850      4860      4870      4880      4890      4900
                          ATAAGAAATGTCTTTCCTATAACACAAAAGCAGATGAGTTGTATTGGCCAGCACCTTCCCTCTATAAGGCGCATGGTCGAGGCGCT
PH207(1>13023)        →   ataagaaatgtctttcctataacacaaaagcagatgagttgtattggccagcaccttccctctataaggcgcatggtcgaggcgct
B73(1>13011)          →   ataagaaatgtctttcctataacacaaaagcagatgagttgtattggccagcaccttccctctataaggcgcatggtcgaggcgct
WT_an_576_f(1>1142)   →   ATAAGAAATGTCTTTCCTATAACACAAAAGCAGATGAGTTGTATTGGCCAGCACCTTCCCTCTATAAGGCGCATGGTCGAGGCGCT
1744_576_f(1>1170)    →   ATAAGAAATGTCTTTCCTATAACACAAAAGCAGATGAGTTGTATTGGCCAGCACCTTCCCTCTATAAGGCGCATGGTCGAGGCGCT
WT_an_586_r(1>1140)   ←   ATAAGAAATGTCTTTCCTATAACACAAAAGCAGATGAGTTGTATTGGCCAGCACCTTCCCTCTATAAGGCGCATGGTCGAGGCGCT
1744_586_r(1>1139)    ←   ATAAGAAATGTCTTTCCTATAACACAAAAGCAGATGAGTTGTATTGGCCAGCACCTTCCCTCTATAAGGCGCATGGTCGAGGCGCT
1744_613_f(1>1134)    →   ATAAGAAATGTCTTTCCTATAACACAAAAGCAGATGAGTTGTATTGGCCAGCACCTTCCCTCTATAAGGCGCATGGTCGAGGCGCT
WT_an_613_f(1>1105)   →   ATAAGAAATGTCTTTCCTATAACACAAAAGCAGATGAGTTGTATTGGCCAGCACCTTCCCTCTATAAGGCGCATGGTCGAGGCGCT
```

```
                      4910      4920      4930      4940      4950      4960      4970      4980
                      GCAGAGGAGGCACATAGGGAGGGAGGATGCACAACGATTTTGACACTTAGGGCTAGTTTGACAACTCAATTTTTCTAACACCCCGT
PH207(1>13023)    →   gcagaggaggcacatagggagggaggatgcacaacgattttgacacttagggctagtttgacaactcaatttttctaacaccccgt
B73(1>13011)      →   gcagaggaggcacatagggagggaggatgcacaacgattttgacacttagggctagtttgacaactcaatttttctaacaccccgt
WT_an_576_f(1>1142) → GCAGAGGAGGCACATAGGGAGGGAGGATGCACAACGATTTTGACACTTAGGGCTAGTTTGACAACTCAATTTTTCTAACACCCCGT
1744_576_f(1>1170)  → GCAGAGGAGGCACATAGGGAGGGAGGATGCACAACGATTTTGACACTTAGGGCTAGTTTGACAACTCAATTTTTCTAACACCCCGT
WT_an_586_r(1>1140) ← GCAGAGGAGGCACATAGGGAGGGAGGATGCACAACGATTTTGACACTTAGGGCTAGTTTGACAACTCAATTTTTCTAACACCCCGT
1744_586_r(1>1139)  ← GCAGAGGAGGCACATAGGGAGGGAGGATGCACAACGATTTTGACACTTAGGGCTAGTTTGACAACTCAATTTTTCTAACACCCCGT
1744_613_f(1>1134)  → GCAGAGGAGGCACATAGGGAGGGAGGATGCACAACGATTTTGACACTTAGGGCTAGTTTGACAACTCAATTTTTCTAACACCCCGT
WT_an_613_f(1>1105) → GCAGAGGAGGCACATAGGGAGGGAGGATGCACAACGATTTTGACACTTAGGGCTAGTTTGACAACTCAATTTTTCTAACACCCCGT
```

```
                      4990      5000      5010      5020      5030      5040      5050      5060      5070
                      TTGGATCATTGGAATTGAATTCCATTCTAATAACAGTAATTTAGGTATATATCAATTAAGCTAATTCGGTTTTATGTAAAATATA-
PH207(1>13023)    →   ttggatcattggaattgaattccattctaataacagtaatttaggtatatatcaattaagctaattcggttttatgtaaaatata-
B73(1>13011)      →   ttggatcattggaattgaattccattctaataacagtaatttaggtatatatcaattaagctaattcggttttatgtaaaatata-
WT_an_576_f(1>1142) → TTGGATCATTGGAATTGAATTCCATTCTAATAACAGTAATTTAGGTATATATCAATTAAGCTAATTCGGTTTTATGTAAAATATA-
1744_576_f(1>1170)  → TTGGATCATTGGAATTGAATTCCATTCTAATAACAGTAATTTAGGTATATATCAATTAAGCTAATTCGGTTTTATGTAAAATATA-
WT_an_586_r(1>1140) ← TTGGATCATTGGAATTGAATTCCATTCTAATAACAGTAATTTAGGTATATATCAATTAAGCTAATTCGGTTTTATGTAAAATATA-
1744_586_r(1>1139)  ← TTGGATCATTGGAATTGAATTCCATTCTAATAACAGTAATTTAGGTATATATCAATTAAGCTAATTCGGTTTTATGTAAAATATA-
1744_613_f(1>1134)  → TTGGATCATTGGAATTGAATTCCATTCTAATAACAGTAATTTAGGTATATATCAATTAAGCTAATTCGGTTTTATGTAAAATATA-
WT_an_613_f(1>1105) → TTGGATCATTGGAATTGAATTCCATTCTAATAACAGTAATTTAGGTATATATCAATTAAGCTAATTCGGTTTTATGTAAAATATA-
1744_621_r(1>1162)  ←                                                       ATTAAGCTAATTCGGTTTTAAGTAAAA-ATAA
```

```
                      5080      5090      5100      5110      5120      5130      5140      5150      5160
                      TTTGTATACTATTATTATTAAGATGTCGGAGATATTTATGTGCTATATTTTTAGTATAGA-GGAGTGAGACGAATAGGGT-CATGT
PH207(1>13023)    →   tttgtatactattattattaagatgtcggagatatttatgtgctatatttttagtataga-ggagtgagacgaatagggt-catgt
B73(1>13011)      →   tttgtatactattattattaagatgtcggagatatttatgtgctatatttttagtataga-ggagtgagacgaatagggt-catgt
WT_an_576_f(1>1142) → TTTGTATACTATTATTATTAAGATGTCGGAGATATTTATGTGCTATATTTTTAGTATAGAAGGAGTGAGACGAATAGGGT-CATGT
1744_576_f(1>1170)  → TTTGTATACTATTATTATTAAGATGTCGGAGATATTTATGTGCTATATTTTTAGTATAGA-GGAGTGAGACGAATAGGGT-CATGT
WT_an_586_r(1>1140) ← TTTGTATACTATTATTATTAAGATGTCGGAGATATTTATGTGCTATATTTTTAGTATAGA-GGAGTGAGACGAATAGGGT-CATGT
1744_586_r(1>1139)  ← TTTGTATACTATTATTATTAAGATGTCGGAGATATTTATGTGCTATATTTTTAGTATAGA-GGAGTGAGACGAATAGGGT-CATGT
1744_613_f(1>1134)  → TTTGTATACTATTATTATTAAGATGTCGGAGATATTTATGTGCTATATTTTTAGTATAGA-GGAGTGAGACGAATAGGGT-CATGT
WT_an_613_f(1>1105) → TTTGTATACTATTATTATTAAGATGTCGGAGATATTTATGTGCTATATTTTTAGTATAGA-GGAGTGAGACGAATAGGGT-CATGT
1744_621_r(1>1162)  ← TTGGTAAACTATTATTATTAAGATGTCGGAGATATTTATGTGCTATATTTTTAGTATAGA-GGAGTGAGACGAATAGGGT-CATGT
WT_an_621_r(1>1072) ←                                                              GGAGTGAGACGAATAGGGTTCATGT
```

```
                      5170      5180      5190      5200      5210      5220      5230      5240
                      AATTTACAGAGTACAAACAAATTC-TACTAATACATAAAATCATTTTCAATCCT-CCACCCCATGAATTCGAGGTAACCTTATATC
PH207(1>13023)    →   aatttacagagtacaaacaaattc-tactaatacataaaatcattttcaatcct-ccaccccatgaattcgaggtaaccttatatc
B73(1>13011)      →   aatttacagagtacaaacaaattc-tactaatacataaaatcattttcaatcct-ccaccccatgaattcgaggtaaccttatatc
WT_an_576_f(1>1142) → AATTTACAGAGTACAAACAAATTC-TACTAATACATAAAATCATTTTCAATCCT-CCACCCCATGAATTCGAGGTAACCTTATATC
1744_576_f(1>1170)  → AATTTACAGAGTACAAACAAATTC-TACTAATACATAAAATCATTTTCAATCCT-CCACCCCATGAATTCGAGGTAACCTTATATC
WT_an_586_r(1>1140) ← AATTTACAGAGTACAAACAAATTC-TACTAATACATAAAATCATTTTCAATCCT-CCACCCCATGAATTCGAGGTAACCTTATATC
1744_586_r(1>1139)  ← AATTTACAGAGTACAAACAAATTC-TACTAATACATAAAATCATTTTCAATCCT-CCACCCCATGAATTCGAGGTAACCTTATATC
1744_613_f(1>1134)  → AATTTACAGAGTACAAACAAATTC-TACTAATACATAAAATCATTTTCAATCCT-CCACCCCATGAATTCGAGGTAACCTTATATC
WT_an_613_f(1>1105) → AATTTACAGAGTACAAACAAATTC-TACTAATACATAAAATCATTTTCAATCCT-CCACCCCATGAATTCGAGGTAACCTTATATC
1744_621_r(1>1162)  ← AATTTACAGAGTACAAACAAATTCTTACTAATACATAAAATCATTTTCAATCCT-CCACCCCATGAATTCGAGGTAACCTTATATC
WT_an_621_r(1>1072) ← AATTTACCGAGTACAAACAAATTC-TACTAATACATAAAATCATTTTCAATCCTCCCACCCCATGAATTCGAGGTAACCTTATATC
```

```
                      5250      5260      5270      5280      5290      5300      5310      5320      5330
                      TGGACTTTAGAAAATGGTGGAATATCAAATTTCGAGCTAAATATGTTACTTTATTGAATGAATTTCAATTCCTTTAAAATGAAGGG
PH207(1>13023)    →   tggactttagaaaatggtggaatatcaaatttcgagctaaatatgttactttattgaatgaatttcaattcctttaaaatgaaggg
B73(1>13011)      →   tggactttagaaaatggtggaatatcaaatttcgagctaaatatgttactttattgaatgaatttcaattcctttaaaatgaaggg
WT_an_576_f(1>1142) → TGGACTTTAGAAAATGGTGGAATATCAAATTTCGAGCTAAATATGTTACTTTATTGAATGAATTTCAATTCCTTTAAAATGAAGGG
1744_576_f(1>1170)  → TGGACTTTAGAAAATGGTGGAATATCAAATTTCGAGCTAAATATGTTACTTTATTGAATGAATTTCAATTCCTTTAAAATGAAGGG
WT_an_586_r(1>1140) ← TGGACTTTAGAAAATGGTGGAATATCAAATTTCGAGCTAAATATGTTACTTTATTGAATGAATTTCAATTCCTTTAAAATGAAGGG
1744_586_r(1>1139)  ← TGGACTTTAGAAAATGGTGGAATATCAAATTTCGAGCTAAATATGTTACTTTATTGAATGAATTTCAATTCCTTTAAAATGAAGGG
1744_613_f(1>1134)  → TGGACTTTAGAAAATGGTGGAATATCAAATTTCGAGCTAAATATGTTACTTTATTGAATGAATTTCAATTCCTTTAAAATGAAGGG
WT_an_613_f(1>1105) → TGGACTTTAGAAAATGGTGGAATATCAAATTTCGAGCTAAATATGTTACTTTATTGAATGAATTTCAATTCCTTTAAAATGAAGGG
1744_621_r(1>1162)  ← TGGACTTTAGAAAATGGTGGAATATCAAATTTCGAGCTAAATATGTTACTTTATTGAATGAATTTCAATTCCTTTAAAATGAAGGG
WT_an_621_r(1>1072) ← TGGACTTTAGAAAATGGTGGAATATCAAATTTCGAGCTAAATATGTTACTTTATTGAATGAATTTCAATTCCTTTAAAATGAAGGG
```

```
                      5340      5350      5360      5370      5380      5390      5400      5410
                      ATTCAAACGGCCCGCAAGGGAAAATAAACTGGTTTCATTGGAAAATGGAAATTTCTTCAAAGAATGGGATTGCCAAACTAGCCTTT
PH207(1>13023)    →   attcaaacggcccgcaagggaaaataaactggtttcattggaaaatggaaatttcttcaaagaatgggattgccaaactagccttt
B73(1>13011)      →   attcaaacggcccgcaagggaaaataaactggtttcattggaaaatggaaatttcttcaaagaatgggattgccaaactagccttt
WT_an_576_f(1>1142) → ATTCAAACGGCCCGCAAGGGAAA
1744_576_f(1>1170)  → ATTCAAACGGCCCGCAAGGGAAAATAAACTGGTTTCATTGGAAAATGGAAATT
WT_an_586_r(1>1140) ← ATTCAAACGGCCCGCAAGGGAAAATAAACTGGTTTCATTGGAAAATGGAAATTTCTTCAAAGAATGGGATTGCCAAACTAGCCTTT
```

```
                       5340      5350      5360      5370      5380      5390      5400      5410
                       ATTCAAACGGCCCGCAAGGGAAAATAAACTGGTTTCATTGGAAAATGGAAATTTCTTCAAAGAATGGGATTGCCAAACTAGCCTTT
1744_586_r(1>1139)     ATTCAAACGGCCCGCAAGGGAAAATAAACTGGTTTCATTGGAAAATGGAAATTTCTTCAAAGAATGGGATTGCCAAACTAGCCTTT
1744_613_f(1>1134)     ATTCAAACGGCCCGCAAGGGAAAATAAACTGGTTTCATTGGAAAATGGAAATTTCTTCAAAGAATGGGATTGCCAAACTAGCCTTT
WT_an_613_f(1>1105)    ATTCAAACGGCCCGCAAGGGAAAATAAACTGGTTTCATTGGAAAATGGAAATTTCTTCAAAGAATGGGATTGCCAAACTAGCCTTT
1744_621_r(1>1162)     ATTCAAACGGCCCGCAAGGGAAAATAAACTGGTTTCATTGGAAAATGGAAATTTCTTCAAAGAATGGGATTGCCAAACTAGCCTTT
WT_an_621_r(1>1072)    ATTCAAACGGCCCGCAAGGGAAAATAAACTGGTTTCATTGGAAAATGGAAATTTCTTCAAAGAATGGGATTGCCAAACTAGCCTTT

                       5420      5430      5440      5450      5460      5470      5480      5490      5500
                       AAAGTAGTAGGAGATCTATTTATTTATTTTGACAAAATATATGAAAAAAAGGTTTTAGCTCCTTCACGATCATCGATCGATGCCTT
PH207(1>13023)         aaagtagtaggagatctatttatttattttgacaaaatatatgaaaaaaaggttttagctccttcacgatcatcgatcgatgcctt
B73(1>13011)           aaagtagtaggagatctatttatttattttgacaaaatatatgaaaaaaaggttttagctccttcacgatcatcgatcgatgcctt
WT_an_586_r(1>1140)    AAAGTAGTAGGAGATCTATTTATTTATTTTGACAAAATATATGAAAAAAAGGTTTTAGCTCCTTCACGATCATCGATCGATGCCTT
1744_586_r(1>1139)     AAAGTAGTAGGAGATCTATTTATTTATTTTGACAAAATATATGAAAAAAAGGTTTTAGCTCCTTCACGATCATCGATCGATGCCTT
1744_613_f(1>1134)     AAAGTAGTAGGAGATCTATTTATTTATTTTGACAAAATATATGAAAAAAAGGTTTTAGCTCCTTCACGATCATCGATCGATGCCTT
WT_an_613_f(1>1105)    AAAGTAGTAGGAGATCTATTTATTTATTTTGACAAAATATATGAAAAAAAGGTTTTAGCTCCTTCACGATCATCGATCGATGCCTT
1744_621_r(1>1162)     AAAGTAGTAGGAGATCTATTTATTTATTTTGACAAAATATATGAAAAAAAGGTTTTAGCTCCTTCACGATCATCGATCGATGCCTT
WT_an_621_r(1>1072)    AAAGTAGTAGGAGATCTATTTATTTATTTTGACAAAATATATGAAAAAAAGGTTTTAGCTCCTTCACGATCATCGATCGATGCCTT

                       5510      5520      5530      5540      5550      5560      5570      5580      5590
                       ATCGGTGACGGAAGGTGGATCGCTATCAACCGTACTGCTGCAAACAGTAGTTGACTGCGAAATTAAGATTCTTGATGTGTGATTGA
PH207(1>13023)         atcggtgacggaaggtggatcgctatcaaccgtactgctgcaaacagtagttgactgcgaaattaagattcttgatgtgtgattga
B73(1>13011)           atcggtgacggaaggtggatcgctatcaaccgtactgctgcaaacagtagttgactgcgaaattaagattcttgatgtgtgattga
WT_an_586_r(1>1140)    ATCGGTGACGGAAGGTGGATCGCTATCAACCGTACTGCTGCAAACAGTAGTTGACTGCGAAATTAAGATTCTTGATGTGTGATTGA
1744_586_r(1>1139)     ATCGGTGACGGAAGGTGGATCGCTATCAACCGTACTGCTGCAAACAGTAGTTGACTGCGAAATTAAGATTCTTGATGTGTGATTGA
1744_613_f(1>1134)     ATCGGTGACGGAAGGTGGATCGCTATCAACCGTACTGCTGCAAACAGTAGTTGACTGCGAAATTAAGATTCTTGATGTGTGATTGA
WT_an_613_f(1>1105)    ATCGGTGACGGAAGGTGGATCGCTATCAACCGTACTGCTGCAAACAGTAGTTGACTGCGAAATTAAGATTCTTGATGTGTGATTGA
1744_621_r(1>1162)     ATCGGTGACGGAAGGTGGATCGCTATCAACCGTACTGCTGCAAACAGTAGTTGACTGCGAAATTAAGATTCTTGATGTGTGATTGA
WT_an_621_r(1>1072)    ATCGGTGACGGAAGGTGGATCGCTATCAACCGTACTGCTGCAAACAGTAGTTGACTGCGAAATTAAGATTCTTGATGTGTGATTGA

                       5600      5610      5620      5630      5640      5650      5660      5670
                       TTATGAGGTAACTGTCCACATTGTCCTCTTTGTTTCTTGACCCCAAATCCTTGGCGAACTTTACTCATCAATCACAAAGGTATCTC
PH207(1>13023)         ttatgaggtaactgtccacattgtcctctttgtttcttgaccccaaatccttggcgaactttactcatcaatcacaaaggtatctc
B73(1>13011)           ttatgaggtaactgtccacattgtcctctttgtttcttgaccccaaatccttggcgaactttactcatcaatcacaaaggtatctc
WT_an_586_r(1>1140)    TTATGAGGTAACTGTCCACATTGTCCTCTTTGTTTCTTGACCCCAAA
1744_586_r(1>1139)     TTATGAGGTAACTGTCCACATTGTCCTCTTTGTTTCTTGACCCCAAA
1744_613_f(1>1134)     TTATGAGGTAACTGTCCACATTGTCCTCTTTGTTTCTTGACCCCAAATCCTTGGCGAACTTTACTCATCAATCACAAAGGTATCTC
WT_an_613_f(1>1105)    TTATGAGGTAACTGTCCACATTGTCCTCTTTGTTTCTTGACCCCAAATCCTTGGCGAACTTTACTCATCAATCACAAAGGTATCTC
1744_621_r(1>1162)     TTATGAGGTAACTGTCCACATTGTCCTCTTTGTTTCTTGACCCCAAATCCTTGGCGAACTTTACTCATCAATCACAAAGGTATCTC
WT_an_621_r(1>1072)    TTATGAGGTAACTGTCCACATTGTCCTCTTTGTTTCTTGACCCCAAATCCTTGGCGAACTTTACTCATCAATCACAAAGGTATCTC

                       5680      5690      5700      5710      5720      5730      5740      5750      5760
                       T-G-TATGTGTGTGGTCAGAGGGATCCTGTCCTCCTTGTACCCCACTGTTCTTTTTAGCTAATGCAAATTGCCATTGGATCAACTT
PH207(1>13023)         t-g-tatgtgtgtggtcagagggatcctgtcctccttgtaccccactgttctttttagctaatgcaaattgccattggatcaactt
B73(1>13011)           t-g-tatgtgtgtggtcagagggatcctgtcctccttgtaccccactgttctttttagctaatgcaaattgccattggatcaactt
1744_613_f(1>1134)     T-GGTATGTGTGTGGTCAGAGGGATCCTGTCCTCCTTGTACCCCACTGTTCTTTTTAGCTAATGCAAATTGCCATTGGATCAACTT
WT_an_613_f(1>1105)    TTG-TATGTGTGTGGTCAGAGGGATCCTGTCCTCCTTGTACCCCACTGTTCTTTTTAGCTAATGCAAATTGCCATTGGATCAACTT
1744_621_r(1>1162)     T-G-TATGTGTGTGGTCAGAGGGATCCTGTCCTCCTTGTACCCCACTGTTCTTTTTAGCTAATGCAAATTGCCATTGGATCAACTT
WT_an_621_r(1>1072)    T-G-TATGTGTGTGGTCAGAGGGATCCTGTCCTCCTTGTACCCCACTGTTCTTTTTAGCTAATGCAAATTGCCATTGGATCAACTT

                       5770      5780      5790      5800      5810      5820      5830      5840
                       GG-TGATTTTGATGGATACAGAGATCAAATATCCGATCAGGAATATAGTAGATATGGCACTCCAAGGTAGATAGATGATTAGACTG
PH207(1>13023)         gg-tgattttgatggatacagagatcaaatatccgatcaggaatatagtagatatggcactccaaggtagatagatgattagactg
B73(1>13011)           gg-tgattttgatggatacagagatcaaatatccgatcaggaatatagtagatatggcactccaaggtagatagatgattagactg
1744_613_f(1>1134)     GG-TGATTTTGATGGATACGGAGATCAAATTTCCGAT
WT_an_613_f(1>1105)    GGGTGATTTTGATG
1744_621_r(1>1162)     GG-TGATTTTGATGGATACAGAGATCAAATATCCGATCAGGAATATAGTAGATATGGCACTCCAAGGTAGATAGATGATTAGACTG
WT_an_621_r(1>1072)    GG-TGATTTTGATGGATACAGAGATCAAATATCCGATCAGGAATATAGTAGATATGGCACTCCAAGGTAGATAGATGATTAGACTG

                       5850      5860      5870      5880      5890      5900      5910      5920      5930
                       GACCGCAGAGGTATCTTCAGTTTTTTTTTGCCATGTAACTCGCGCGCACAGCAGGGAATCTGATTGTGTGTGTAAGGGAGATTTTA
PH207(1>13023)         gaccgcagaggtatcttcagtttttttttgccatgtaactcgcgcgcacagcagggaatctgattgtgtgtgtaagggagatttta
B73(1>13011)           gaccgcagaggtatcttcagtttttttttgccatgtaactcgcgcgcacagcagggaatctgattgtgtgtgtaagggagatttta
1744_621_r(1>1162)     GACCGCAGAGGTATCTTCAGTTTTTTTTTGCCATGTAACTCGCGCGCACAGCAGGGAATCTGATTGTGTGTGTAAGGGAGATTTTA
WT_an_621_r(1>1072)    GACCGCAGAGGTATCTTCAGTTTTTTTTTGCCATGTAACTCGCGCGCACAGCAGGGAATCTGATTGTGTGTGTAAGGGAGATTTTA
```

```
                    5940      5950      5960      5970      5980      5990      6000      6010      6020
                    AAGAACTGGCCATCTATCTTCTGTTGGGAGAAATGAAGTCAAAATGGGTAATGCAGTGCTGAATCTGACGCTCTAGTTGACGTTAC
PH207(1>13023)    → aagaactggccatctatcttctgttgggagaaatgaagtcaaaatgggtaatgcagtgctgaatctgacgctctagttgacgttac
B73(1>13011)      → aagaactggccatctatcttctgttgggagaaatgaagtcaaaatgggtaatgcagtgctgaatctgacgctctagttgacgttac
1744_621_r(1>1162) ← AAGAACTGGCCATCTATCTTCTGTTGGGAGAAATGAAGTCAAAATGGGTAATGCAGTGCTGAATCTGACGCTCTAGTTGACGTTAC
WT_an_621_r(1>1072) ← AAGAACTGGCCATCTATCTTCTGTTGGGAGAAATGAAGTCAAAATGGGTAATGCAGTGCTGAATCTGACGCTCTAGTTGACGTTAC

                    6030      6040      6050      6060      6070      6080      6090      6100
                    GCTGCTCTGCTCAGGCCAGCAGGTTCATGGTCCTTTGTTGCATGCTGAACACTTGCTAGAGATGCAATTGGGTGCCGAAAAATAAA
PH207(1>13023)    → gctgctctgctcaggccagcaggttcatggtcctttgttgcatgctgaacacttgctagagatgcaattgggtgccgaaaaataaa
B73(1>13011)      → gctgctctgctcaggccagcaggttcatggtcctttgttgcatgctgaacacttgctagagatgcaattgggtgccgaaaaataaa
1744_621_r(1>1162) ← GCTGCTCTGCTCAGGCCAGCAGGTTCATGGTCCTTTGTTGCATGCTGAACACTTGCTAGAGATGCAATTGGGTGCCGAAAAATAAA
WT_an_621_r(1>1072) ← GCTGCTCTGCTCAGGCCAGCAGGTTCATGGTCCTTTGTTGCATGCTGAACACTTGCTAGAGATGCAATTGGGTGCCGAAAAATAAA

                    6110      6120      6130      6140      6150      6160      6170      6180      6190
                    CCAACAGTAGCTCAATCTGACCAGTCACATAATTTTCTTCAGTTTTCCTCCTTTGAGAAATGTTTTGTTGGAGCTGGGTTTTCCTG
PH207(1>13023)    → ccaacagtagctcaatctgaccagtcacataattttcttcagttttcctcctttgagaaatgttttgttggagctgggttttcctg
B73(1>13011)      → ccaacagtagctcaatctgaccagtcacataattttcttcagttttcctcctttgagaaatgttttgttggagctgggttttcctg
1744_621_r(1>1162) ← CCAACAGTAGCTCAATCTGACCAGTCACATAATTTTCTTCAGTTTTCCTCCTTTGAGAAATGTTTTGTTGGAGCTGGGTTTTCCTG
WT_an_621_r(1>1072) ← CCAACAGTAGCTCAATCTGACCAGTCACATAATTTTCTTCAGTTTTCCTCCTTTGAGAAATGTTTTGTTGGAGCTGGGTTTTCCTG

                    6200      6210      6220      6230      6240      6250      6260      6270
                    CAGTGTTCCAGACCAGCCGTGTGGAAACCGAGTCCGAAATTGCGAAATGGCCAGGGAAACCACAAGACCTTGAGGATGAGCACCAG
PH207(1>13023)    → cagtgttccagaccagccgtgtggaaaccgagtccgaaattgcgaaatggccagggaaaccacaagaccttgaggatgagcaccag
B73(1>13011)      → cagtgttccagaccagccgtgtggaaaccgagtccgaaattgcgaaatggccagggaaaccacaagaccttgaggatgagcaccag
1744_621_r(1>1162) ← CAGTGTTCCAGACCAGCCG
WT_an_621_r(1>1072) ← CAGTGTTCCAGACCAGCCG

                    6280      6290      6300      6310      6320      6330      6340      6350      6360
                    GTGGTCCGTCTTAGGTTGTTTTCAGTTCACAGATGCTTGTTGCTGAAATTAATAAATCTTCGTCTGTAGGCTGAGGAGGCAGAGCT
PH207(1>13023)    → gtggtccgtcttaggttgttttcagttcacagatgcttgttgctgaaattaataaatcttcgtctgtaggctgaggaggcagagct
B73(1>13011)      → gtggtccgtcttaggttgttttcagttcacagatgcttgttgctgaaattaataaatcttcgtctgtaggctgaggaggcagagct

                    6370      6380      6390      6400      6410      6420      6430      6440      6450
                    GCAGCCACTTATCGACCAGGTGAGGGCGATGCTACGGTCGATGAACGACGGGGATACCAGCGCCTCGGCGTACGACACGGCGTGGG
PH207(1>13023)    → gcagccacttatcgaccaggtgagggcgatgctacggtcgatgaacgacggggataccagcgcctcggcgtacgacacggcgtggg
B73(1>13011)      → gcagccacttatcgaccaggtgagggcgatgctacggtcgatgaacgacggggataccagcgcctcggcgtacgacacggcgtggg

                    6460      6470      6480      6490      6500      6510      6520      6530
                    TGGCGATGGTGCCGAAGSYGGRCGGCGRCGGSGGCGCCCAGCCCCAGTTCCCGGCCACCGTGCGCTGGATCGTGGACCACCAGCTG
PH207(1>13023)    → tggcgatggtgccgaagccggacggcggcggggcgcccagccccagttcccggccaccgtgcgctggatcgtggaccaccagctg
B73(1>13011)      → tggcgatggtgccgaaggtgggcggcgacggcggcgcccagccccagttcccggccaccgtgcgctggatcgtggaccaccagctg

                    6540      6550      6560      6570      6580      6590      6600      6610      6620
                    CCCGACGGCTCCTGGGGCGACTCGGCCCTGTTCTCCGCCTACGACCGCATGATCAACACCCTCGCCTGCGTCGTCGCGCTGACCAA
PH207(1>13023)    → cccgacggctcctggggcgactcggccctgttctccgcctacgaccgcatgatcaacaccctcgcctgcgtcgtcgcgctgaccaa
B73(1>13011)      → cccgacggctcctggggcgactcggccctgttctccgcctacgaccgcatgatcaacaccctcgcctgcgtcgtcgcgctgaccaa

                    6630      6640      6650      6660      6670      6680      6690      6700
                    GTGGTCGCTGGAGCCCGCGAGGTGCGAGGCGGGGCTCTCGTTCCTGCACGAGAACATGTGGAGGCTAGCGGAGGAGGAGGCGGAGT
PH207(1>13023)    → gtggtcgctggagcccgcgaggtgcgaggcggggctctcgttcctgcacgagaacatgtggaggctagcggaggaggaggcggagt
B73(1>13011)      → gtggtcgctggagcccgcgaggtgcgaggcggggctctcgttcctgcacgagaacatgtggaggctagcggaggaggaggcggagt

                    6710      6720      6730      6740      6750      6760      6770      6780      6790
                    CGATGCCCATCGGCTTCGAGATCGCCTTCCCTTCTCTCATCCAGACGGCTAGGGACCTGGGCGTCGTCGACTTCCCGTACGGACAC
PH207(1>13023)    → cgatgcccatcggcttcgagatcgccttcccttctctcatccagacggctagggacctgggcgtcgtcgacttcccgtacggacac
B73(1>13011)      → cgatgcccatcggcttcgagatcgccttcccttctctcatccagacggctagggacctgggcgtcgtcgacttcccgtacggacac
```

```
                  6800      6810      6820      6830      6840      6850      6860      6870      6880
                  CCGGCGCTGCAGAGCATATACGCCAACAGGGAAGTCAAGCTGAAGCGGATCCCAAGGGACATGATGCACAGGGTCCCGACGTCCAT
PH207(1>13023)    ccggcgctgcagagcatatacgccaacagggaagtcaagctgaagcggatcccaagggacatgatgcacagggtcccgacgtccat
B73(1>13011)      ccggcgctgcagagcatatacgccaacagggaagtcaagctgaagcggatcccaagggacatgatgcacagggtcccgacgtccat

                  6890      6900      6910      6920      6930      6940      6950      6960
                  CCTGCACAGCCTTGAAGGGATGCCTGACCTGGACTGGCCGAGGCTTCTGAACCTCCAGTCCTGCGACGGCTCCTTCTTGTTCTCTC
PH207(1>13023)    cctgcacagccttgaagggatgcctgacctggactggccgaggcttctgaacctccagtcctgcgacggctccttcttgttctctc
B73(1>13011)      cctgcacagccttgaagggatgcctgacctggactggccgaggcttctgaacctccagtcctgcgacggctccttcttgttctctc

                  6970      6980      6990      7000      7010      7020      7030      7040      7050
                  CTTCGGCTACCGCTTACGCGCTGATGCAAACCGGTGACAAGAAGTGCTTCGAATACATCGACAGGATTGTCAAAAAATTCAACGGG
PH207(1>13023)    cttcggctaccgcttacgcgctgatgcaaaccggtgacaagaagtgcttcgaatacatcgacaggattgtcaaaaaattcaacggg
B73(1>13011)      cttcggctaccgcttacgcgctgatgcaaaccggtgacaagaagtgcttcgaatacatcgacaggattgtcaaaaaattcaacggg

                  7060      7070      7080      7090      7100      7110      7120      7130
                  GGAGGTAAGCCGATCGTCCATGCATGGAGGATTAATTAAGACGATCGATGATGTTTAATCCGTGTCTCGTCTCATCAGACTGTTTG
PH207(1>13023)    ggaggtaagccgatcgtccatgcatggaggattaattaagacgatcgatgatgtttaatccgtgtctcgtctcatcagactgtttg
B73(1>13011)      ggaggtaagccgatcgtccatgcatggaggattaattaagacgatcgatgatgtttaatccgtgtctcgtctcatcagactgtttg

                  7140      7150      7160      7170      7180      7190      7200      7210      7220
                  CCATCACCGTTTCAGTCCCCAATGTTTATCCGGTCGATCTTTTCGAGCACATCTGGGTTGTGGATCGGTTGGAGCGACTCGGGATC
PH207(1>13023)    ccatcaccgtttcagtccccaatgtttatccggtcgatcttttcgagcacatctgggttgtggatcggttggagcgactcgggatc
B73(1>13011)      ccatcaccgtttcagtccccaatgtttatccggtcgatcttttcgagcacatctgggttgtggatcggttggagcgactcgggatc

                  7230      7240      7250      7260      7270      7280      7290      7300      7310
                  TCCCGCTACTTCCAACGAGAGATTGAGCAGTGCATGGACTATGTGAACAGGTTTTTGCTTCTGCGATCGATCACTCTTTATGTGAA
PH207(1>13023)    tcccgctacttccaacgagagattgagcagtgcatggactatgtgaacaggtttttgcttctgcgatcgatcactctttatgtgaa
B73(1>13011)      tcccgctacttccaacgagagattgagcagtgcatggactatgtgaacaggtttttgcttctgcgatcgatcactctttatgtgaa
1744_578_f(1>1090)                                                                          TCTTTATGTGAA
WT_an_578_f(1>1091)                                                                         TCTTTATGTG-A

                  7320      7330      7340      7350      7360      7370      7380      7390
                  CAGGTTTTTTTATGACAGATTGAGTAGATGAATTTCTTTGACTTGTCTTGTCATTTCGCGTAGGCACTGGACTGAAGATGGGATTT
PH207(1>13023)    caggtttttttatgacagattgagtagatgaatttctttgacttgtcttgtcatttcgcgtaggcactggactgaagatgggattt
B73(1>13011)      caggtttttttatgacagattgagtagatgaatttctttgacttgtcttgtcatttcgcgtaggcactggactgaagatgggattt
1744_578_f(1>1090) CAGGTTTTTTTATGACAGATTGAGTAGATGAATTTCTTTGACTTGTCTTGTCATTTCGCGTAGGCACTGGACTGAAGATGGGATTT
WT_an_578_f(1>1091) CAGGTTTTTTTATGACAGATTGAGTAGATGAATTTCTTTGACTTGTCTTGTCATTTCGCGTAGGCACTGGACTGAAGATGGGATTT

                  7400      7410      7420      7430      7440      7450      7460      7470      7480
                  GCTGGGCTAGGAAATCCAATGTGAAGGATGTGGATGACACAGCTATGGCTTTCCGACTACTAAGGCTACATGGATACAATGTCTCT
PH207(1>13023)    gctgggctaggaaatccaatgtgaaggatgtggatgacacagctatggctttccgactactaaggctacatggatacaatgtctct
B73(1>13011)      gctgggctaggaaatccaatgtgaaggatgtggatgacacagctatggctttccgactactaaggctacatggatacaatgtctct
1744_578_f(1>1090) GCTGGGCTAGGAAATCCAATGTGAAGGATGTGGATGACACAGCTATGGCTTTCCGACTACTAAGGCTACATGGATACAATGTCTCT
WT_an_578_f(1>1091) GCTGGGCTAGGAAATCCAATGTGAAGGATGTGGATGACACAGCTATGGCTTTCCGACTACTAAGGCTACATGGATACAATGTCTCT

                  7490      7500      7510      7520      7530      7540      7550      7560
                  CCAAGTATATATAAACACCATTCCCTTTTTAGCTTAAACATCTCATT-AACTTGTTATTATATCTTAATGACATAAGCCAGCCGTG
PH207(1>13023)    ccaagtatatataaacaccattccctttttagcttaaacatctcatt-aacttgttattatatcttaatgacataagccagccgtg
B73(1>13011)      ccaagtatatataaacaccattccctttttagcttaaacatctcatt-aacttgttattatatcttaatgacataagccagccgtg
1744_578_f(1>1090) CCAAGTATATATAAACACCATTCCCTTTTTAGCTTAAACATCTCATT-AACTTGTTATTATATCTTAATGACATAAGCCAGCCGTG
WT_an_578_f(1>1091) CCAAGTATATATAAACACCATTCCCTTTTTAGCTTAAACATCTCATT-AACTTGTTATTATATCTTAATGACATAAGCCAGCCGTG
1744_588_r(1>1048)                       TCCCTTTTTAGCTTAAACATCTCATT-AACTTGTTATTATATCTTAATGACATAAGCCAGCCGTG
WT_an_588_r(1>1046)                          CTTTTTAGCTTAAACATCTCATTAAACTTGTTATTATATCTTAATGACATAAGCCAGCCGTG

                  7570      7580      7590      7600      7610      7620      7630      7640      7650
                  TTCTGTAGGTGTGTTTAAGAACTTTGAGAAAGATGGAGAGTTCTTTTGTTTTGTGGGCCAATCGACTCAAGCCGTCACTGGGATGT
PH207(1>13023)    ttctgtaggtgtgtttaagaactttgagaaagatggagagttcttttgttttgtgggccaatcgactcaagccgtcactgggatgt
B73(1>13011)      ttctgtaggtgtgtttaagaactttgagaaagatggagagttcttttgttttgtgggccaatcgactcaagccgtcactgggatgt
1744_578_f(1>1090) TTCTGTAGGTGTGTTTAAGAACTTTGAGAAAGATGGAGAGTTCTTTTGTTTTGTGGGCCAATCGACTCAAGCCGTCACTGGGATGT
WT_an_578_f(1>1091) TTCTGTAGGTGTGTTTAAGAACTTTGAGAAAGATGGAGAGTTCTTTTGTTTTGTGGGCCAATCGACTCAAGCCGTCACTGGGATGT
```

7570 7580 7590 7600 7610 7620 7630 7640 7650

TTCTGTAGGTGTGTTTAAGAACTTTGAGAAAGATGGAGAGTTCTTTTGTTTTGTGGGCCAATCGACTCAAGCCGTCACTGGGATGT

1744_588_r(1>1048) ← TTCTGTAGGTGTGTTTAAGAACTTTGAGAAAGATGGAGAGTTCTTTTGTTTTGTGGGCCAATCGACTCAAGCCGTCACTGGGATGT
WT_an_588_r(1>1046) ← TTCTGTAGGTGTGTTTAAGAACTTTGAGAAAGATGGAGAGTTCTTTTGTTTTGTGGGCCAATCGACTCAAGCCGTCACTGGGATGT

7660 7670 7680 7690 7700 7710 7720 7730 7740

ATAACCTCAACAGAGCCTCTCAGATAAGTTTTCAAGGAGAGGATGTATTGCATCGTGCTAGGGTTTTCTCGTATGAGTTTCTGAGA

PH207(1>13023) → ataacctcaacagagcctctcagataagttttcaaggagaggatgtattgcatcgtgctagggttttctcgtatgagtttctgaga
B73(1>13011) → ataacctcaacagagcctctcagataagttttcaaggagaggatgtattgcatcgtgctagggttttctcgtatgagtttctgaga
1744_578_f(1>1090) → ATAACCTCAACAGAGCCTCTCAGATAAGTTTTCAAGGAGAGGATGTATTGCATCGTGCTAGGGTTTTCTCGTATGAGTTTCTGAGA
WT_an_578_f(1>1091) → ATAACCTCAACAGAGCCTCTCAGATAAGTTTTCAAGGAGAGGATGTATTGCATCGTGCTAGGGTTTTCTCGTATGAGTTTCTGAGA
1744_588_r(1>1048) ← ATAACCTCAACAGAGCCTCTCAGATAAGTTTTCAAGGAGAGGATGTATTGCATCGTGCTAGGGTTTTCTCGTATGAGTTTCTGAGA
WT_an_588_r(1>1046) ← ATAACCTCAACAGAGCCTCTCAGATAAGTTTTCAAGGAGAGGATGTATTGCATCGTGCTAGGGTTTTCTCGTATGAGTTTCTGAGA

7750 7760 7770 7780 7790 7800 7810 7820

CAGAGAGAAGAACAAGGCATGATCCGTGATAAATGGATCGTTGCCAAGGATCTACCTGGCGAGGTAATCCAAACCATTCTACCATT

PH207(1>13023) → cagagagaagaacaaggcatgatccgtgataaatggatcgttgccaaggatctacctggcgaggtaatccaaaccattctaccatt
B73(1>13011) → cagagagaagaacaaggcatgatccgtgataaatggatcgttgccaaggatctacctggcgaggtaatccaaaccattctaccatt
1744_578_f(1>1090) → CAGAGAGAAGAACAAGGCATGATCCGTGATAAATGGATCGTTGCCAAGGATCTACCTGGCGAGGTAATCCAAACCATTCTACCATT
WT_an_578_f(1>1091) → CAGAGAGAAGAACAAGGCATGATCCGTGATAAATGGATCGTTGCCAAGGATCTACCTGGCGAGGTAATCCAAACCATTCTACCATT
1744_588_r(1>1048) ← CAGAGAGAAGAACAAGGCATGATCCGTGATAAATGGATCGTTGCCAAGGATCTACCTGGCGAGGTAATCCAAACCATTCTACCATT
WT_an_588_r(1>1046) ← CAGAGAGAAGAACAAGGCATGATCCGTGATAAATGGATCGTTGCCAAGGATCTACCTGGCGAGGTAATCCAAACCATTCTACCATT

7830 7840 7850 7860 7870 7880 7890 7900 7910

TGATGATCTTAGATCCATTGAAACATGCATGAATAGAGGCGAAAATTAGACGGTGTTATTTTTTTGGCTTTCATTGTGTTGATCGA

PH207(1>13023) → tgatgatcttagatccattgaaacatgcatgaatagaggcgaaaattagacggtgttatttttttggctttcattgtgttgatcga
B73(1>13011) → tgatgatcttagatccattgaaacatgcatgaatagaggcgaaaattagacggtgttatttttttggctttcattgtgttgatcga
1744_578_f(1>1090) → TGATGATCTTAGATCCATTGAAACATGCATGAATAGAGGCGAAAATTAGACGGTGTTATTTTTTTGGCTTTCATTGTGTTGATCGA
WT_an_578_f(1>1091) → TGATGATCTTAGATCCATTGAAACATGCATGAATAGAGGCGAAAATTAGACGGTGTTATTTTTTTGGCTTTCATTGTGTTGATCGA
1744_588_r(1>1048) ← TGATGATCTTAGATCCATTGAAACATGCATGAATAGAGGCGAAAATTAGACGGTGTTATTTTTTTGGCTTTCATTGTGTTGATCGA
WT_an_588_r(1>1046) ← TGATGATCTTAGATCCATTGAAACATGCATGAATAGAGGCGAAAATTAGACGGTGTTATTTTTTTGGCTTTCATTGTGTTGATCGA

7920 7930 7940 7950 7960 7970 7980 7990

TAGGTGCAATATACACTAGACTTCCCTTGGTATGCAAGCTTGCCTCGTGTAGAGGCAAGAACCTATCTAGATCAATATGGTGGTAA

PH207(1>13023) → taggtgcaatatacactagacttcccttggtatgcaagcttgcctcgtgtagaggcaagaacctatctagatcaatatggtggtaa
B73(1>13011) → taggtgcaatatacactagacttcccttggtatgcaagcttgcctcgtgtagaggcaagaacctatctagatcaatatggtggtaa
1744_578_f(1>1090) → TAGGTGCAATATACACTAGACTTCCCTTGGTATGCAAGCTTGCCTCGTGTAGAGGCAAGAACCTATCTAGATCAATATGGTGGTAA
WT_an_578_f(1>1091) → TAGGTGCAATATACACTAGACTTCCCTTGGTATGCAAGCTTGCCTCGTGTAGAGGCAAGAACCTATCTAGATCAATATGGTGGTAA
1744_588_r(1>1048) ← TAGGTGCAATATACACTAGACTTCCCTTGGTATGCAAGCTTGCCTCGTGTAGAGGCAAGAACCTATCTAGATCAATATGGTGGTAA
WT_an_588_r(1>1046) ← TAGGTGCAATATACACTAGACTTCCCTTGGTATGCAAGCTTGCCTCGTGTAGAGGCAAGAACCTATCTAGATCAATATGGTGGTAA

8000 8010 8020 8030 8040 8050 8060 8070 8080

AGATGACGTTTGGATTGGAAAGACACTCTACAGGTGCACACTTGTACTCCAAAAAAAAACGTTGATATACTTCGTTGCATACTTAT

PH207(1>13023) → agatgacgtttggattggaaagacactctacaggtgcacacttgtactccaaaaaaaaacgttgatatacttcgttgcatacttat
B73(1>13011) → agatgacgtttggattggaaagacactctacaggtgcacacttgtactccaaaaaaaaacgttgatatacttcgttgcatacttat
1744_578_f(1>1090) → AGATGACGTTTGGATTGGAAAGACACTCTACAGGTGCACACTTGTACTCCAAAAAAAAACGTTGATATACTTCGTTGCATACTTAT
WT_an_578_f(1>1091) → AGATGACGTTTGGATTGGAAAGACACTCTACAGGTGCACACTTGTACTCCAAAAAAAAACGTTGATATACTTCGTTGCATACTTAT
1744_588_r(1>1048) ← AGATGACGTTTGGATTGGAAAGACACTCTACAGGTGCACACTTGTACTCCAAAAAAAAACGTTGATATACTTCGTTGCATACTTAT
WT_an_588_r(1>1046) ← AGATGACGTTTGGATTGGAAAGACACTCTACAGGTGCACACTTGTACTCCAAAAAAAAACGTTGATATACTTCGTTGCATACTTAT

8090 8100 8110 8120 8130 8140 8150 8160 8170

TATTAGTGTTTTTGGCAAGAACCTATCTAGATCAATATGTAAGAGAAATGTTCTACTTACATGTGCGGCGTTTTTGGCAGGATGCC

PH207(1>13023) → tattagtgtttttggcaagaacctatctagatcaatatgtaagagaaatgttctacttacatgtgcggcgtttttggcaggatgcc
B73(1>13011) → tattagtgtttttggcaagaacctatctagatcaatatgtaagagaaatgttctacttacatgtgcggcgtttttggcaggatgcc
1744_578_f(1>1090) → TATTAGTGTTTTTGGCAAGAACCTATCTAGATCAATATGTAAGAGAAATGTTCTACTTACATGTGCGGCGTTTTTGGCAGGATGCC
WT_an_578_f(1>1091) → TATTAGTGTTTTTGGCAAGAACCTATCTAGATCAATATGTAAGAGAAATGTTCTACTTACATGTGCGGCGTTTTTGGCAGGATGCC
1744_588_r(1>1048) ← TATTAGTGTTTTTGGCAAGAACCTATCTAGATCAATATGTAAGAGAAATGTTCTACTTACATGTGCGGCGTTTTTGGCAGGATGCC
WT_an_588_r(1>1046) ← TATTAGTGTTTTTGGCAAGAACCTATCTAGATCAATATGTAAGAGAAATGTTCTACTTACATGTGCGGCGTTTTTGGCAGGATGCC

8180 8190 8200 8210 8220 8230 8240 8250

TCTTGTGAATAACGACACATATCTAGAGTTGGCAATAAGGGATTTCAACCATTGCCAAGCTCTGCATCAGCTTGAGTGTAATGGGC

PH207(1>13023) → tcttgtgaataacgacacatatctagagttggcaataagggatttcaaccattgccaagctctgcatcagcttgagtgtaatgggc
B73(1>13011) → tcttgtgaataacgacacatatctagagttggcaataagggatttcaaccattgccaagctctgcatcagcttgagtgtaatgggc
1744_578_f(1>1090) → TCTTGTGAATAACGACACATATCTAGAGTTGGCAATAAGGGATTTCAACCATTGCCAAGCTCTGCATCAGCTTGAGTGTAATGGGC
WT_an_578_f(1>1091) → TCTTGTGAATAACGACACATATCTAGAGTTGGCAATAAGGGATTTCAACCATTGCCAAGCTCTGCATCAGCTTGAGTGTAATGGGC

```
                           8180      8190      8200      8210      8220      8230      8240      8250
                           TCTTGTGAATAACGACACATATCTAGAGTTGGCAATAAGGGATTTCAACCATTGCCAAGCTCTGCATCAGCTTGAGTGTAATGGGC
1744_588_r(1>1048)     ←   TCTTGTGAATAACGACACATATCTAGAGTTGGCAATAAGGGATTTCAACCATTGCCAAGCTCTGCATCAGCTTGAGTGTAATGGGC
WT_an_588_r(1>1046)    ←   TCTTGTGAATAACGACACATATCTAGAGTTGGCAATAAGGGATTTCAACCATTGCCAAGCTCTGCATCAGCTTGAGTGTAATGGGC
```

```
                           8260      8270      8280      8290      8300      8310      8320      8330      8340
                           TGCAAACGTGAGCACCATCATTTCATTCTCCACTTCATGAATTTTATGCTGGAAGGTTAACTTTGATTTAATTTACAATATTTTCT
PH207(1>13023)         →   tgcaaacgtgagcaccatcatttcattctccacttcatgaattttatgctggaaggttaactttgatttaatttacaatattttct
B73(1>13011)           →   tgcaaacgtgagcaccatcatttcattctccacttcatgaattttatgctggaaggttaactttgatttaatttacaatattttct
1744_578_f(1>1090)     →   TGCAAACGTGAGCACCATCATTTCATTCTCCACTTCATGAATTTTATGCTGGAAGGTTAACTTTGATTTAATTTACAATATTTTCT
WT_an_578_f(1>1091)    →   TGCAAACGTGAGCACCATCATTTCATTCTCCACTTCATGAATTTTATGCTGGAAGGTTAACTTTGATTTAATTTACAATATTTTCT
1744_588_r(1>1048)     ←   TGCAAACGTGAGCACCATCATTTCATTCTCCACTTCATGAATTTTATGCTGGAAGGTTAACTTTGATTTAATTTACAATATTTTCT
WT_an_588_r(1>1046)    ←   TGCAAACGTGAGCACCATCATTTCATTCTCCACTTCATGAATTTTATGCTGGAAGGTTAACTTTGATTTAATTTACAATATTTTCT
```

```
                           8350      8360      8370      8380      8390      8400      8410      8420
                           AAACTATATTTT-AGGTGGTA-CAAGGATAATTGCCTTGACGCTTTTGGAGTAGAACCACAAGATGTTTTAAGATCTTACTTTTTA
PH207(1>13023)         →   aaactatatttt-aggtggta-caaggataattgccttgacgcttttggagtagaaccacaagatgttttaagatcttacttttta
B73(1>13011)           →   aaactatatttt-aggtggta-caaggataattgccttgacgcttttggagtagaaccacaagatgttttaagatcttacttttta
1744_578_f(1>1090)     →   AAACTATATTTT-AGGTGGTA-CAAGGATAATTGCCTTGACGCTTTTGG
WT_an_578_f(1>1091)    →   AAACTATATTTTTAGGTGGTACCAAGGATAATTGCCTTGACGCTTTTGG
1744_588_r(1>1048)     ←   AAACTATATTTT-AGGTGGTA-CAAGGATAATTGCCTTGACGCTTTTGGAGTAGAACCACAAGATGTTTTAAGATCTTACTTTTTA
WT_an_588_r(1>1046)    ←   AAACTATATTTT-AGGTGGTA-CAAGGATAATTGCCTTGACGCTTTTGGAGTAGAACCACAAGATGTTTTAAGATCTTACTTTTTA
```

```
                          8430      8440      8450      8460      8470      8480      8490      8500      8510
                           GCTGCTGCTTGCATTTTTGAACCTAGCCGTGCTGCTGAGCGGCTTGCATGGGCTAGAACGTCAATGATTGCCAATGCCATTTCTAC
PH207(1>13023)         →   gctgctgcttgcatttttgaacctagccgtgctgctgagcggcttgcatgggctagaacgtcaatgattgccaatgccatttctac
B73(1>13011)           →   gctgctgcttgcatttttgaacctagccgtgctgctgagcggcttgcatgggctagaacgtcaatgattgccaatgccatttctac
1744_588_r(1>1048)     ←   GCTGCTGCTTGCATTTTTGAACCTAGCCGTGCTGCTGAGCGGCTTGCATGGGCTAGAACGTCAATGATTGCCAATGCCATTTCTAC
WT_an_588_r(1>1046)    ←   GCTGCTGCTTGCATTTTTGAACCTAGCCGTGCTGCTGAGCGGCTTGCATGGGCTAGAACGTCAATGATTGCCAATGCCATTTCTAC
```

```
                             8520      8530      8540      8550      8560      8570      8580      8590      8600
                           ACATCTTCGTGACATTTCGGAAGACAAGAAGAGATTGGAATGTTTCGTGCACTGTCTCTATGAAGAAAACGATGTATCATGGTGAG
PH207(1>13023)         →   acatcttcgtgacatttcggaagacaagaagagattggaatgtttcgtgcactgtctctatgaagaaaacgatgtatcatggtgag
B73(1>13011)           →   acatcttcgtgacatttcggaagacaagaagagattggaatgtttcgtgcactgtctctatgaagaaaacgatgtatcatggtgag
1744_588_r(1>1048)     ←   ACATCTTCGTGACATTTCGGAAGACAAGAAGAGATTGGAA
WT_an_588_r(1>1046)    ←   ACATCTTCGTGACATTTCGGAAGACAAGAAGAGATTGGAA
WT_635_2_r(1>395)      ←                                                    CACTGTCTCTATGAAGAAAACGATGTATCATGGTGAG
1744_635_2_r(1>375)    ←                                                    CACTGTCTCTATGAAGAAAACGATGTATCATGGTGAG
```

```
                               8610      8620      8630      8640      8650      8660      8670      8680
                           TACTGTACCTTAATTATATCAGTTAACCATTATATATCCATTTAAGAATCATCTTGAAGCACTTGTCATTTACCAAGTAGTAGTAA
PH207(1>13023)         →   tactgtaccttaattatatcagttaaccattatatatccatttaagaatcatcttgaagcacttgtcatttaccaagtagtagtaa
B73(1>13011)           →   tactgtaccttaattatatcagttaaccattatatatccatttaagaatcatcttgaagcacttgtcatttaccaagtagtagtaa
1744_579_f(1>1100)     →                                                     TCTTGAAGCACTTGTCATTTACCAAGTAGTAGTAA
WT_an_579_f(1>1111)    →                                                     TCTTGAAGCACTTGTCATTTACCAAGTAGTAGTAA
WT_635_2_r(1>395)      ←   TACTGTACCTTAATTATATCAGTTAACCATTATATATCCATTTAAGAATCATCTTGAAGCACTTGTCATTTACCAAGTAGTAGTAA
1744_635_2_r(1>375)    ←   TACTGTACCTTAATTATATCAGTTAACCATTATATATCCATTTAAGAATCATCTTGAAGCACTTGTCATTTACCAAGTAGTAGTAA
1744_634_1_f(1>332)    →                                                                           AAGTAGTAGTAA
```

```
                           8690      8700      8710      8720      8730      8740      8750      8760      8770
                           CATTGGCATTTGATTTCTTAAACAAACAAATAAATAAATTGGGTAATAGAACGAATGAATTTCATAATTATATATATAGGAAGATCAA
PH207(1>13023)         →   cattggcatttgatttcttaaacaaacaaataaataaattgggtaatagaacgaatgaatttcataattatatatataggaagatcaa
B73(1>13011)           →   cattggcatttgatttcttaaacaaacaaataaataaattgggtaatagaacgaatgaatttcataattatatatataggaagatcaa
1744_579_f(1>1100)     →   CATTGGCATTTGATTTCTTAAACAAACAAATAAATAAATTGGGTAATAGAACGAATGAATTTCATAATTATATATATAGGAAGATCAA
WT_an_579_f(1>1111)    →   CATTGGCATTTGATTTCTTAAACAAACAAATAAATAAATTGGGTAATAGAACGAATGAATTTCATAATTATATATATAGGAAGATCAA
WT_635_2_r(1>395)      ←   CATTGGCATTTGATTTCTTAAACAAACAAATAAATAAATTGGGTAATAGAACGAATGAATTTCATAATTATATATATAGGAAGATCAA
1744_635_2_r(1>375)    ←   CATTGGCATTTGATTTCTTAAACAAACAAATAAATAAATTGGGTAATAGAACGAATGAATTTCATAATTATATATATAGGAAGATCAA
1744_634_1_f(1>332)    →   CATTGGCATTTGATTTCTTAAACAAACAAATAAATAAATTGGGTAATAGAACGAATGAATTTCATAATTATATATATAGGAAGATCAA
WT_634_1_f(1>298)      →                          CAAACAAATAAATAAATTGGGTAATAGAACGAATGAATCTCATAATTATATATATAGGAAGATCAA
```

```
                             8780      8790      8800      8810      8820      8830      8840      8850
                           TAATGATATTTTAACAATAAGGATTTATGTAGTTACGTAATCTTTGTTATATATACTCATCGCAGTGTACATATAAAAGTAAATTG
PH207(1>13023)         →   taatgatattttaacaataaggatttatgtagttacgtaatctttgttatatatactcatcgcagtgtacatataaaagtaaattg
B73(1>13011)           →   taatgatattttaacaataaggatttatgtagttacgtaatctttgttatatatactcatcgcagtgtacatataaaagtaaattg
1744_579_f(1>1100)     →   TAATGATATTTTAACAATAAGGATTTATGTAGTTACGTAATCTTTGTTATATATACTCATCGCAGTGTACATATAAAAGTAAATTG
```

```
                                    8780      8790      8800      8810      8820      8830      8840      8850
                                    TAATGATATTTTAACAATAAGGATTTATGTAGTTACGTAATCTTTGTTATATATACTCATCGCAGTGTACATATAAAAGTAAATTG
WT_an_579_f(1>1111)        →        TAATGATATTTTAACAATAAGGATTTATGTAGTTACGTAATCTTTGTTATATATACTCATCGCAGTGTACATATAAAAGTAAATTG
WT_635_2_r(1>395)          ←        TAATGATATTTTAACAATAAGGATTTATGTAGTTACGTAATCTTTGTTATATATACTCATCGCAGTGTACATATAAAAGTAAATTG
1744_635_2_r(1>375)        ←        TAATGATATTTTAACAATAAGGATTTATGTAGTTACGTAATCTTTGTTATATATACTCATCGCAGTGTACATATAAAAGTAAATTG
1744_634_1_f(1>332)        →        TAATGATATTTTAACAATAAGGATTTATGTAGTTACGTAATCTTTGTTATATATACTCATCGCAGTGTACATATAAAAGTAAATTG
WT_634_1_f(1>298)          →        TAATGATATTTTAACAATAAGGATTTATGTAGTTACGTAATCTTTGTTATATATACTCATCGCAGTGTACATATAAAAGTAAATTG
```

```
                                  8860      8870      8880      8890      8900      8910      8920      8930      8940
                                    TACCAAATTGATCTTTGATATTCAGAATATGCATTAGCATTTATTGCATGAGTCAATATTGTGCATTAGTTACATATATTACAAAA
PH207(1>13023)             →        taccaaattgatctttgatattcagaatatgcattagcatttattgcatgagtcaatattgtgcattagttacatatattacaaaa
B73(1>13011)               →        taccaaattgatctttgatattcagaatatgcattagcatttattgcatgagtcaatattgtgcattagttacatatattacaaaa
1744_579_f(1>1100)         →        TACCAAATTGATCTTTGATATTCAGAATATGCATTAGCATTTATTGCATGAGTCAATATTGTGCATTAGTTACATATATTACAAAA
WT_an_579_f(1>1111)        →        TACCAAATTGATCTTTGATATTCAGAATATGCATTAGCATTTATTGCATGAGTCAATATTGTGCATTAGTTACATATATTACAAAA
WT_635_2_r(1>395)          ←        TACCAAATTGATCTTTGATATTCAGAATATGCATTAGCATTTATTGCATGAGTCAATATTGTGCATTAGTTACATATATTACAAAA
1744_635_2_r(1>375)        ←        TACCAAATTGATCTTTGATATTCAGAATATGCATTAGCATTTATTGCATGAGTCAATATTGTGCATTAGTTACATATATT
1744_634_1_f(1>332)        →        TACCAAATTGATCTTTGATATTCAGAATATGCATTAGCATTTATTGCATGAGTCAATATTGTGCATTAGTTACATATATTACAAAA
WT_634_1_f(1>298)          →        TACCAAATTGATCTTTGATATTCAGAATATGCATTAGCATTTATTGCATGAGTCAATATTGTGCATTAGTTACATATATTACAAAA
```

```
                                     8950      8960      8970      8980      8990      9000      9010      9020      9030
                                    GAGGTAAAGATGACGTCATATTCTTTTTTAGCTTCCTTTTTGTCAACTCGTGTGGACTGATTGTGCTTTATTCTGGTGGCCATTAT
PH207(1>13023)             →        gaggtaaagatgacgtcatattcttttttagcttcctttttgtcaactcgtgtggactgattgtgctttattctggtggccattat
B73(1>13011)               →        gaggtaaagatgacgtcatattcttttttagcttcctttttgtcaactcgtgtggactgattgtgctttattctggtggccattat
1744_579_f(1>1100)         →        GAGGTAAAGATGACGTCATATTCTTTTTTAGCTTCCTTTTTGTCAACTCGTGTGGACTGATTGTGCTTTATTCTGGTGGCCATTAT
WT_an_579_f(1>1111)        →        GAGGTAAAGATGACGTCATATTCTTTTTTAGCTTCCTTTTTGTCAACTCGTGTGGACTGATTGTGCTTTATTCTGGTGGCCATTAT
WT_635_2_r(1>395)          ←        GAGGTAAAGATGAC
1744_634_1_f(1>332)        →        GAGGTAAAGATGACGTCATATTCTTTTTTAGCTTCCTTTTTGTCAACTCGTGTGGACTGATT
WT_634_1_f(1>298)          →        GAGGTAAAGATGACGTCATATTCTTTTTTAGCTTCCTTTTTGTCAACTCGTGTGGACTGATT
```

```
                                         9040      9050      9060      9070      9080      9090      9100      9110
                                    GTCATGCCACTTAATTTTTTTGATGTATTATTATCGCCTCTTAAGTTATGACCTCAATGAAAACTTGCATAAGTTCATTGATATAT
PH207(1>13023)             →        gtcatgccacttaatttttttgatgtattattatcgcctcttaagttatgacctcaatgaaaacttgcataagttcattgatatat
B73(1>13011)               →        gtcatgccacttaatttttttgatgtattattatcgcctcttaagttatgacctcaatgaaaacttgcataagttcattgatatat
1744_579_f(1>1100)         →        GTCATGCCACTTAATTTTTTTGATGTATTATTATCGCCTCTTAAGTTATGACCTCAATGAAAACTTGCATAAGTTCATTGATATAT
WT_an_579_f(1>1111)        →        GTCATGCCACTTAATTTTTTTGATGTATTATTATCGCCTCTTAAGTTATGACCTCAATGAAAACTTGCATAAGTTCATTGATATAT
```

```
                                      9120      9130      9140      9150      9160      9170      9180      9190      9200
                                    ACTTTAGATATTTTATATCGATGTTGTAATTTTTAACTCGCATTTTACACCGACCTC-TTTTATTTTAGCTGTTTTATTTAACATG
PH207(1>13023)             →        actttagatattttatatcgatgttgtaatttttaactcgcattttacaccgacctc-ttttattttagctgttttatttaacatg
B73(1>13011)               →        actttagatattttatatcgatgttgtaatttttaactcgcattttacaccgacctc-ttttattttagctgttttatttaacatg
1744_579_f(1>1100)         →        ACTTTAGATATTTTATATCGATGTTGTAATTTTTAACTCGCATTTTACACCGACCTC-TTTTATTTTAGCTGTTTTATTTAACATG
WT_an_579_f(1>1111)        →        ACTTTAGATATTTTATATCGATGTTGTAATTTTTAACTCGCATTTTACACCGACCTC-TTTTATTTTAGCTGTTTTATTTAACATG
1744_589_r(1>1106)         ←                                    TTGTAATTTTTAACTCGCATTTTACACCGACCTTTTTTTATTTTAGCTGTTTTATTTAACATG
WT_an_589_r(1>1103)        ←                                           ATTTTTAACTCGCATTTTACACCGACCTTTTTTTATTTTAGCTGTTTTATTTAACATG
```

```
                                    9210      9220      9230      9240      9250      9260      9270      9280
                                    TGTATTCTATTTTCATCGTGTCATATATTATGTAATATATGAAATTGAGAGAATATTATTTTCCTGACATAAATGATAAATTCAGG
PH207(1>13023)             →        tgtattctattttcatcgtgtcatatattatgtaatatatgaaattgagagaatattattttcctgacataaatgataaattcagg
B73(1>13011)               →        tgtattctattttcatcgtgtcatatattatgtaatatatgaaattgagagaatattattttcctgacataaatgataaattcagg
1744_579_f(1>1100)         →        TGTATTCTATTTTCATCGTGTCATATATTATGTAATATATGAAATTGAGAGAATATTATTTTCCTGACATAAATGATAAATTCAGG
WT_an_579_f(1>1111)        →        TGTATTCTATTTTCATCGTGTCATATATTATGTAATATATGAAATTGAGAGAATATTATTTTCCTGACATAAATGATAAATTCAGG
1744_589_r(1>1106)         ←        TGTATTCTATTTTCATCGTGTCATATATTATGTAATATATGAAATTGAGAGAATATTATTTTCCTGACATAAATGATAAATTCAGG
WT_an_589_r(1>1103)        ←        TGTATTCTATTTTCATCGTGTCATATATTATGTAATATATGAAATTGAGAGAATATTATTTTCCTGACATAAATGATAAATTCAGG
```

```
                                  9290      9300      9310      9320      9330      9340      9350      9360      9370
                                    CTTAAACGAAATCCTAAT-GATGTTATTCTTGAGAGGGCACTTCGAAGATTAATTAACTTATTAGCACAAGAAGCATTGCCAATTC
PH207(1>13023)             →        cttaaacgaaatcctaat-gatgttattcttgagagggcacttcgaagattaattaacttattagcacaagaagcattgccaattc
B73(1>13011)               →        cttaaacgaaatcctaat-gatgttattcttgagagggcacttcgaagattaattaacttattagcacaagaagcattgccaattc
1744_579_f(1>1100)         →        CTTAAACGAAATCCTAAT-GATGTTATTCTTGAGAGGGCACTTCGAAGATTAATTAACTTATTAGCACAAGAAGCATTGCCAATTC
WT_an_579_f(1>1111)        →        CTTAAACGAAATCCTAAT-GATGTTATTCTTGAGAGGGCACTTCGAAGATTAATTAACTTATTAGCACAAGAAGCATTGCCAATTC
1744_589_r(1>1106)         ←        CTTAAACGAAATCCTAATGGATGTTATTCTTGAGAGGGCACTTCGAAGATTAATTAACTTATTAGCACAAGAAGCATTGCCAATTC
WT_an_589_r(1>1103)        ←        CTTAAACGAAATCCTAAT-GATGTTA-TCTTGAGAGGGCACTTCGAAGATTAATTAACTTATTAGCACAAGAAGCATTGCCAATTC
```

```
                      9380      9390      9400      9410      9420      9430      9440      9450      9460
                      ATGAAGGACAAAGATTCATACACAGTCTATTGAGTCTTGCAGTAAGTTATCCCCACTTGTACTTAATTAGATTATAACGACTTCTT
PH207(1>13023)    →   atgaaggacaaagattcatacacagtctattgagtcttgcagtaagttatccccacttgtacttaattagattataacgacttctt
B73(1>13011)      →   atgaaggacaaagattcatacacagtctattgagtcttgcagtaagttatccccacttgtacttaattagattataacgacttctt
1744_579_f(1>1100)  → ATGAAGGACAAAGATTCATACACAGTCTATTGAGTCTTGCAGTAAGTTATCCCCACTTGTACTTAATTAGATTATAACGACTTCTT
WT_an_579_f(1>1111) → ATGAAGGACAAAGATTCATACACAGTCTATTGAGTCTTGCAGTAAGTTATCCCCACTTGTACTTAATTAGATTATAACGACTTCTT
1744_589_r(1>1106)  ← ATGAAGGACAAAGATTCATACACAGTCTATTGAGTCTTGCAGTAAGTTATCCCCACTTGTACTTAATTAGATTATAACGACTTCTT
WT_an_589_r(1>1103) ← ATGAAGGACAAAGATTCATACACAGTCTATTGAGTCTTGCAGTAAGTTATCCCCACTTGTACTTAATTAGATTATAACGACTTCTT

                          9470      9480      9490      9500      9510      9520      9530      9540
                      CTCAACAATCATTCTAA-TATGTTTGTCTATACTTCTAGTGGACCGAATGGATGTTGCAAAAGGCAAATAAAGAAGAAAACAAATA
PH207(1>13023)    →   ctcaacaatcattctaa-tatgtttgtctatacttctagtggaccgaatggatgttgcaaaaggcaaataaagaagaaaacaaata
B73(1>13011)      →   ctcaacaatcattctaa-tatgtttgtctatacttctagtggaccgaatggatgttgcaaaaggcaaataaagaagaaaacaaata
1744_579_f(1>1100)  → CTCAACAATCATTCTAA-TATGTTTGTCTATACTTCTAGTGGACCGAATGGATGTTGCAAAAGGCAAATAAAGAAGAAAACAAATA
WT_an_579_f(1>1111) → CTCAACAATCATTCTAAATATGTTTGTCTATACTTCTAGTGGACCGAATGGATGTTGCAAAAGGCAAATAAAGAAGAAAACAAATA
1744_589_r(1>1106)  ← CTCAACAATCATTCTAA-TATGTTTGTCTATACTTCTAGTGGACCGAATGGATGTTGCAAAAGGCAAATAAAGAAGAAAACAAATA
WT_an_589_r(1>1103) ← CTCAACAATCATTCTAA-TATGTTTGTCTATACTTCTAGTGGACCGAATGGATGTTGCAAAAGGCAAATAAAGAAGAAAACAAATA

                    9550      9560      9570      9580      9590      9600      9610      9620      9630
                      TCACAAATGCAGTGGTATAGAACCACAATACATGGTTCATGATAGGCAAACATACTTACTTTTAGTTCAGGTTATTGA-GATTTGT
PH207(1>13023)    →   tcacaaatgcagtggtatagaaccacaatacatggttcatgataggcaaacatacttacttttagttcaggttattga-gatttgt
B73(1>13011)      →   tcacaaatgcagtggtatagaaccacaatacatggttcatgataggcaaacatacttacttttagttcaggttattga-gatttgt
1744_579_f(1>1100)  → TCACAAATGCAGTGGTATAGAACCACAATACATGGTTCATGATAGGCAAACATACTTACTTTTAGTTCAGGTTATTGA-GATTTGT
WT_an_579_f(1>1111) → TCACAAATGCAGTGGTATAGAACCACAATACATGGTTCATGATAGGCAAACATACTTACTTTTAGTTCAGGTTATTGAAGATTTGT
1744_589_r(1>1106)  ← TCACAAATGCAGTGGTATAGAACCACAATACATGGTTCATGATAGGCAAACATACTTACTTTTAGTTCAGGTTATTGA-GATTTGT
WT_an_589_r(1>1103) ← TCACAAATGCAGTGGTATAGAACCACAATACATGGTTCATGATAGGCAAACATACTTACTTTTAGTTCAGGTTATTGA-GATTTGT

                        9640      9650      9660      9670      9680      9690      9700      9710
                      GCTGGACGAATTGGTGAGGCTGTGTCAATGATAAACAACAAGGATAA-TGATT-GGTTTATTCAACTCACATGTGCTACTTGTGAC
PH207(1>13023)    →   gctggacgaattggtgaggctgtgtcaatgataaacaacaaggataa-tgatt-ggtttattcaactcacatgtgctacttgtgac
B73(1>13011)      →   gctggacgaattggtgaggctgtgtcaatgataaacaacaaggataa-tgatt-ggtttattcaactcacatgtgctacttgtgac
1744_579_f(1>1100)  → GCTGGACGAATTGGTGAGGCTGTGTCAATGATAAACAACAGGGATAA-TGATT-GGTTTATTCAACTCACATGTGCTACTTGTGAC
WT_an_579_f(1>1111) → GCTGGACGAATTGGTGAGGCTGTGTCAATGATAAACAACAAGGATAAATGATTGGGTTTATTCAACTCACATGTGCTACTTGTGAC
1744_589_r(1>1106)  ← GCTGGACGAATTGGTGAGGCTGTGTCAATGATAAACAACAAGGATAA-TGATT-GGTTTATTCAACTCACATGTGCTACTTGTGAC
WT_an_589_r(1>1103) ← GCTGGACGAATTGGTGAGGCTGTGTCAATGATAAACAACAAGGATAA-TGATT-GGTTTATTCAACTCACATGTGCTACTTGTGAC

                  9720      9730      9740      9750      9760      9770      9780      9790      9800
                      AGTCTTAACCATA-GGATGTTACTGTCCCAGGTAAACCTTGTATCATAATTAAATTATTTTTACTCTTTATGTATAGCTAGATACA
PH207(1>13023)    →   agtcttaaccata-ggatgttactgtcccaggtaaaccttgtatcataattaaattatttttactctttatgtatagctagataca
B73(1>13011)      →   agtcttaaccata-ggatgttactgtcccaggtaaaccttgtatcataattaaattatttttactctttatgtatagctagataca
1744_579_f(1>1100)  → AGTCTTAACCATA-GGATGTTACTGTCCCAGGTAAACCTT
WT_an_579_f(1>1111) → AGTCTTAACCATAAGGATGTTACTGTCCCAGGTAAACCTTGTATCA
1744_589_r(1>1106)  ← AGTCTTAACCATA-GGATGTTACTGTCCCAGGTAAACCTTGTATCATAATTAAATTATTTTTACTCTTTATGTATAGCTAGATACA
WT_an_589_r(1>1103) ← AGTCTTAACCATA-GGATGTTACTGTCCCAGGTAAACCTTGTATCATAATTAAATTATTTTTACTCTTTATGTATAGCTAGATACA

                      9810      9820      9830      9840      9850      9860      9870      9880      9890
                      TGTGTGGTCTAATGATTGTTCCATTAGGGGGTAATAATAATTTTAGGATCAAACCATCACATATATAACTATTTTTAGCACCTTAA
PH207(1>13023)    →   tgtgtggtctaatgattgttccattagggggtaataataattttaggatcaaaccatcacatatataactatttttagcaccttaa
B73(1>13011)      →   tgtgtggtctaatgattgttccattagggggtaataataattttaggatcaaaccatcacatatataactatttttagcaccttaa
1744_589_r(1>1106)  ← TGTGTGGTCTAATGATTGTTCCATTAGGGGGTAATAATAATTTTAGGATCAAACCATCACATATATAACTATTTTTAGCACCTTAA
WT_an_589_r(1>1103) ← TGTGTGGTCTAATGATTGTTCCATTAGGGGGTAATAATAATTTTAGGATCAAACCATCACATATATAACTATTTTTAGCACCTTAA

                          9900      9910      9920      9930      9940      9950      9960      9970
                      TTCTCCACCAAAGACTAGAATTTGCTCCTAGATCCTTACAACCTCAAGTTTTGGATTAGGTCATTGTGTAGGTTTCTTTGTCTTTC
PH207(1>13023)    →   ttctccaccaaagactagaatttgctcctagatccttacaacctcaagttttggattaggtcattgtgtaggtttctttgtctttc
B73(1>13011)      →   ttctccaccaaagactagaatttgctcctagatccttacaacctcaagttttggattaggtcattgtgtaggtttctttgtctttc
1744_589_r(1>1106)  ← TTCTCCACCAAAGACTAGAATTTGCTCCTAGATCCTTACAACCTCAAGTTTTGGATTAGGTCATTGTGTAGGTTTCTTTGTCTTTC
WT_an_589_r(1>1103) ← TTCTCCACCAAAGACTAGAATTTGCTCCTAGATCCTTACAACCTCAAGTTTTGGATTAGGTCATTGTGTAGGTTTCTTTGTCTTTC

                    9980      9990     10000     10010     10020     10030     10040     10050     10060
                      CTTGGCTTAGTGAATTCAAAACATTAAGTTTATGACTATGTTGGGCAAGAATAACTTCAATGTTTTGTGAGTCCGGATCTTCAAAA
PH207(1>13023)    →   cttggcttagtgaattcaaaacattaagtttatgactatgttgggcaagaataacttcaatgttttgtgagtccggatcttcaaaa
B73(1>13011)      →   cttggcttagtgaattcaaaacattaagtttatgactatgttgggcaagaataacttcaatgttttgtgagtccggatcttcaaaa
1744_589_r(1>1106)  ← CTTGGCTTAGTGAATTCAAAACATTAAGTTTATGACTATGTTGGGCAAGAATAACTTCAATGTTTTGTGAGTCCGGATCTTCAAAA
WT_an_589_r(1>1103) ← CTTGGCTTAGTGAATTCAAAACATTAAGTTTATGACTATGTTGGGCAAGAATAACTTCAATGTTTTGTGAGTCCGGATCTTCAAAA
```

```
                          10070     10080     10090     10100     10110     10120     10130     10140
                         TGTATACACTAGCAAATTACCCGAACCTTATTCAACGCAAAATCATATAACAAACTTTATTTCATGCAATTTAGAAGCTAAATAAA
PH207(1>13023)      →    tgtatacactagcaaattacccgaaccttattcaacgcaaaatcatataacaaactttatttcatgcaatttagaagctaaataaa
B73(1>13011)        →    tgtatacactagcaaattacccgaaccttattcaacgcaaaatcatataacaaactttatttcatgcaatttagaagctaaataaa
1744_589_r(1>1106)  ←    TGTATACACTAGCAAATTACCCGAACCTTATTCAACGCAAAATCATATAACAAACTTTATTTCATGCAATTTAGAAGCTAAATAAA
WT_an_589_r(1>1103) ←    TGTATACACTAGCAAATTACCCGAACCTTATTCAACGCAAAATCATATAACAAACTTTATTTCATGCAATTTAGAAGCTAAATAAA
```

```
                       10150     10160     10170     10180     10190     10200     10210     10220     10230
                         TTTCCATATATTATTCTAGTTTCATCATCAATATTCCTTACATGTATAAGTAATCCTCCCTTAAGCCATATTTACAACTAAGGACA
PH207(1>13023)      →    tttccatatattattctagtttcatcatcaatattccttacatgtataagtaatcctcccttaagccatatttacaactaaggaca
B73(1>13011)        →    tttccatatattattctagtttcatcatcaatattccttacatgtataagtaatcctcccttaagccatatttacaactaaggaca
1744_589_r(1>1106)  ←    TTTCCATATATTATTCTAGTTTCATCATCAATATTCCTTACATGTATAAGTAATCCTCCCTTAAGCCATATTTACAACTAAGGACA
WT_an_589_r(1>1103) ←    TTTCCATATATTATTCTAGTTTCATCATCAATATTCCTTACATGTATAAGTAATCCTCCCTTAAGCCATATTTACAACTAAGGACA
```

```
                           10240     10250     10260     10270     10280     10290     10300     10310     10320
                         ATGAGTAAATTTAAAATTAAGAATGCTATTAAAACAATTTATTTAATTTTCATTTTCTCTATGGTACATGCAAATGGAATACCAAA
PH207(1>13023)      →    atgagtaaatttaaaattaagaatgctattaaaacaatttatttaattttcattttctctatggtacatgcaaatggaataccaaa
B73(1>13011)        →    atgagtaaatttaaaattaagaatgctattaaaacaatttatttaattttcattttctctatggtacatgcaaatggaataccaaa
1744_589_r(1>1106)  ←    ATGAGTAAATTTAAAA
WT_an_589_r(1>1103) ←    ATGAGTAAATTTAAAATTAA
```

```
                             10330     10340     10350     10360     10370     10380     10390     10400
                         TGTTTCTTAAGAAAAGTATAGATACTATCTTTCTAGGCACTTGTAGCAATCATAGACTCTACTTTGTTTTATGACCATTAATTTAT
PH207(1>13023)      →    tgtttcttaagaaaagtatagatactatctttctaggcacttgtagcaatcatagactctactttgttttatgaccattaatttat
B73(1>13011)        →    tgtttcttaagaaaagtatagatactatctttctaggcacttgtagcaatcatagactctactttgttttatgaccattaatttat
WT_580_1_f(1>769)   →                    TATAGATACTATCTTTCTAGGCACTTGTAGCAATCATAGACTCTACTTTGTTTTATGACCATTAATTTAT
1744_580_f(1>170)   →                        GATACTATCTTTCTAGGCACTTGTAGCAATCATAGACTCTACTTTGTTTTATGACCATTAATTTAT
1744_580_1_f(1>857) →                           ACTATCTTTCTAGGCACTTGTAGCAATCATAGACTCTACTTTGTTTTATGACCATTAATTTAT
WT_an_580_f(1>106)  →                            CTATCTTTCTAGGCACTTGTAGCAATCATAGACTCTACTTTGTTTTATGACCATTAATTTAT
```

```
                         10410     10420     10430     10440     10450     10460     10470     10480     10490
                         AATAAGTTAAATTTGTGATTGTACATATAATCACACATTCCATAAAATTTATTTATTTTTACATGCCAACATGTTGAACTAATGAC
PH207(1>13023)      →    aataagttaaatttgtgattgtacatataatcacacattccataaaatttatttatttttacatgccaacatgttgaactaatgac
B73(1>13011)        →    aataagttaaatttgtgattgtacatataatcacacattccataaaatttatttatttttacatgccaacatgttgaactaatgac
WT_580_1_f(1>769)   →    AATAAGTTAAATTTGTGATTGTACATATAATCACACATTCCATAAAATTTATTTATTTTTACATGCCAACATGTTGAACTAATGAC
1744_580_f(1>170)   →    AATAAGTTAAATTTGTGATTGTACATATAATCACACATTCCATAAAATTTATTTATTTTTACATGCCAACATGTTGAACTAATGAC
1744_580_1_f(1>857) →    AATAAGTTAAATTTGTGATTGTACATATAATCACACATTCCATAAAATTTATTTATTTTTACATGCCAACATGTTGAACTAATGAC
WT_an_580_f(1>106)  →    AATAAGTTAAATTTGTGATTGTACATATAATCACACATTCCATA
```

```
                          10500     10510     10520     10530     10540     10550     10560     10570
                         ACCATGTGCAGGATACTATGAAGAATGAAGCAAGAATAAATTGGATTGAGAAGGAAATCGAGTTGAATATGCAAGAGCTTGCTCAA
PH207(1>13023)      →    accatgtgcaggatactatgaagaatgaagcaagaataaattggattgagaaggaaatcgagttgaatatgcaagagcttgctcaa
B73(1>13011)        →    accatgtgcaggatactatgaagaatgaagcaagaataaattggattgagaaggaaatcgagttgaatatgcaagagcttgctcaa
WT_580_1_f(1>769)   →    ACCATGTGCAGGATACTATGAAGAATGAAGCAAGAATAAATTGGATTGAGAAGGAAATCGAGTTGAATATGCAAGAGCTTGCTCAA
1744_580_f(1>170)   →    ACCATGTGCAGGATACTA
1744_580_1_f(1>857) →    ACCATGTGCAGGATACTATGAAGAATGAAGCAAGAATAAATTGGATTGAGAAGGAAATCGAGTTGAATATGCAAGAGCTTGCTCAA
```

```
                       10580     10590     10600     10610     10620     10630     10640     10650     10660
                         TCTCTCCTTTTGAGATGTGATGAGAAAACTAGCAATAAGAAGACCAAGAAAACCTTATGGGATGTCCTAAGAAGTTTATACTATGC
PH207(1>13023)      →    tctctccttttgagatgtgatgagaaaactagcaataagaagaccaagaaaaccttatgggatgtcctaagaagtttatactatgc
B73(1>13011)        →    tctctccttttgagatgtgatgagaaaactagcaataagaagaccaagaaaaccttatgggatgtcctaagaagtttatactatgc
WT_580_1_f(1>769)   →    TCTCTCCTTTTGAGATGTGATGAGAAAACTAGCAATAAGAAGACCAAGAAAACCTTATGGGATGTCCTAAGAAGTTTATACTATGC
1744_580_1_f(1>857) →    TCTCTCCTTTTGAGATGTGATGAGAAAACTAGCAATAAGAAGACCAAGAAAACCTTATGGGATGTCCTAAGAAGTTTATACTATGC
```

```
                           10670     10680     10690     10700     10710     10720     10730     10740     10750
                         TACTCATTCCCCACAACATATGATCGATAGACATGTTTCCAGAGTTATCTTTGAGCCTGTTTAAAAATGTTTAAGTGGTAGACCAT
PH207(1>13023)      →    tactcattccccacaacatatgatcgatagacatgtttccagagttatctttgagcctgtttaaaaatgtttaagtggtagaccat
B73(1>13011)        →    tactcattccccacaacatatgatcgatagacatgtttccagagttatctttgagcctgtttaaaaatgtttaagtggtagaccat
WT_580_1_f(1>769)   →    TACTCATTCCCCACAACATATGATCGATAGACATGTTTCCAGAGTTATCTTTGAGCCTGTTTAAAAATGTTTAAGTGGTAGACCAT
1744_580_1_f(1>857) →    TACTCATTCCCCACAACATATGATCGATAGACATGTTTCCAGAGTTATCTTTGAGCCTGTTTAAAAATGTTTAAGTGGTAGACCAT
```

```
                     10760     10770     10780     10790     10800     10810     10820     10830
                     TATGTTAGGTGTAAATGTGTACATAAAAGTTATCATAAGGAGTAATGGTAGCAGAAGCATGCAGTTGTAAGTTTATTTGTTGCTTA
PH207(1>13023)       tatgttaggtgtaaatgtgtacataaaagttatcataaggagtaatggtagcagaagcatgcagttgtaagtttatttgttgctta
B73(1>13011)         tatgttaggtgtaaatgtgtacataaaagttatcataaggagtaatggtagcagaagcatgcagttgtaagtttatttgttgctta
WT_580_1_f(1>769)    TATGTTAGGTGTAAATGTGTACATAAAAGTTATCATAAGGAGTAATGGTAGCAGAAGCATGCAGTTGTAAGTTTATTTGTTGCTTA
1744_580_1_f(1>857)  TATGTTAGGTGTAAATGTGTACATAAAAGTTATCATAAGGAGTAATGGTAGCAGAAGCATGCAGTTGTAAGTTTATTTGTTGCTTA
1744_590_2_r(1>482)                                                    GCAGAAGCATGCAGTTGTAAGTTTATTTGTTGCTTA
```

```
                     10840     10850     10860     10870     10880     10890     10900     10910     10920
                     GAATAGAAATTAGTGTAGCTATAATATCAAGAATGTTCCTATATAAGTAATCATATTATGGATAGAGGTGTTCATATGAATAATAA
PH207(1>13023)       gaatagaaattagtgtagctataatatcaagaatgttcctatataagtaatcatattatggatagaggtgttcatatgaataataa
B73(1>13011)         gaatagaaattagtgtagctataatatcaagaatgttcctatataagtaatcatattatggatagaggtgttcatatgaataataa
WT_580_1_f(1>769)    GAATAGAAATTAGTGTAGCTATAATATCAAGAATGTTCCTATATAAGTAATCATATTATGGATAGAGGTGTTCATATGAATAATAA
1744_580_1_f(1>857)  GAATAGAAATTAGTGTAGCTATAATATCAAGAATGTTCCTATATAAGTAATCATATTATGGATAGAGGTGTTCATATGAATAATAA
1744_590_2_r(1>482)  GAATAGAAATTAGTGTAGCTATAATATCAAGAATGTTCCTATATAAGTAATCATATTATGGATAGAGGTGTTCATATGAATAATAA
WT_590_2_r(1>494)        AGAAATTAGTGTAGCTATA-TATCAAGAATGTTGATATATAAGTAATCATATTATGGATAGAGGTGTTCATATGAATAATAA
```

```
                     10930     10940     10950     10960     10970     10980     10990     11000
                     TTTTATATGTTAAGTGTTATCTTACCTAATTTTAGTAGCATCACCAATTGTCTAGAGAACTTATCTATTTTCTTGGCATCAAAATA
PH207(1>13023)       ttttatatgttaagtgttatcttacctaattttagtagcatcaccaattgtctagagaacttatctattttcttggcatcaaaata
B73(1>13011)         ttttatatgttaagtgttatcttacctaattttagtagcatcaccaattgtctagagaacttatctattttcttggcatcaaaata
WT_580_1_f(1>769)    TTTTATATGTTAAGTGTTATCTTACCTAATTTTAGTAGCATCACCAATTGTCTAGAGAACTTATCTATTTTCTTGGCATCAAAATA
1744_580_1_f(1>857)  TTTTATATGTTAAGTGTTATCTTACCTAATTTTAGTAGCATCACCAATTGTCTAGAGAACTTATCTATTTTCTTGGCATCAAAATA
1744_590_2_r(1>482)  TTTTATATGTTAAGTGTTATCTTACCTAATTTTAGTAGCATCACCAATTGTCTAGAGAACTTATCTATTTTCTTGGCATCAAAATA
WT_590_2_r(1>494)    TTTTATATGTTAAGTGTTATCTTACCTAATTTTAGTAGCATCACCAATTGTCTAGAGAACTTATCTATTTTCTTGGCATCAAAATA
```

```
                     11010     11020     11030     11040     11050     11060     11070     11080     11090
                     CATGTACTAGTTATCAAAGAACAAATAAGGTATCTTATTAATAATAACATACTTATTAAATGTTGAGAATAAATTT-CGGG-ATTT
PH207(1>13023)       catgtactagttatcaaagaacaaataaggtatcttattaataataacatacttattaaatgttgagaataaattt-cggg-attt
B73(1>13011)         catgtactagttatcaaagaacaaataaggtatcttattaataataacatacttattaaatgttgagaataaattt-cggg-attt
WT_580_1_f(1>769)    CATGTACTAGTTATCAAAGAACAAATAAGGTATCTTATTAATAATAACATACTTATTAAATGTTGAGAATAAATTTTCGGG-ATTT
1744_580_1_f(1>857)  CATGTACTAGTTATCAAAGAACAAATAAGGTATCTTATTAATAATAACATACTTATTAAATGTTGAGAATAAATTT-CGGGGATTT
1744_590_2_r(1>482)  CATGTACTAGTTATCAAAGAACAAATAAGGTATCTTATTAATAATAACATACTTATTAAATGTTGAGAATAAATTT-CGGG-ATTT
WT_590_2_r(1>494)    CATGTACTAGTTATCAAAGAACAAATAAGGTATCTTATTAATAATAACATACTTATTAAATGTTGAGAATAAATTT-CGGG-ATTT
```

```
                     11100     11110     11120     11130     11140     11150     11160     11170     11180
                     TTTAGTATTTCATTATATTGG-CACCTTTAAACTACAA-GGAAACTAACAAAACATTTGTACAAAACTGTTGCAACACAGAGAATG
PH207(1>13023)       tttagtatttcattatattgg-cacctttaaactacaa-ggaaactaacaaaacatttgtacaaaactgttgcaacacagagaatg
B73(1>13011)         tttagtatttcattatattgg-cacctttaaactacaa-ggaaactaacaaaacatttgtacaaaactgttgcaacacagagaatg
WT_580_1_f(1>769)    TTTAGTATTTCA
1744_580_1_f(1>857)  TTTAGTATTTCATTATATTGGGCACCTTTAAACTACAAGGGAAACTAACAAAACATTTGTACAAAACTGTTGCAACACAGAGAATG
1744_590_2_r(1>482)  TTTAGTATTTCATTATATTGG-CACCTTTAAACTACAA-GGAAACTAACAAAACATTTGTACAAAACTGTTGCAACACAGAGAATG
WT_590_2_r(1>494)    TTTAGTATTTCATTATATTGG-CACCTTTAAACTACAA-GGAAACTAACAAAACATTTGTACAAAACTGTTGCAACACAGAGAATG
1744_590_r(1>250)      TAGTATTTCATTATATTGG-CACCTTTAAACTACAA-GGAAACTAACAAAACATTTGTACAAAACTGTTGCAACACAGAGAATG
WT_an_590_r(1>202)                                             AACTAACAAAACAATTGTACAAAACTGTTGCAACACAGAGAATG
```

```
                     11190     11200     11210     11220     11230     11240     11250     11260
                     GAAAACCCTAGCTACAGTAACGGGTACAAAATATAGAACGCTAATGGGCCAGCACACATACGGCCCATAATACTCTAACACCCCTC
PH207(1>13023)       gaaaaccctagctacagtaacgggtacaaaatatagaacgctaatgggccagcacacatacggcccataatactctaacacccctc
B73(1>13011)         gaaaaccctagctacagtaacgggtacaaaatatagaacgctaatgggccagcacacatacggcccataatactctaacacccctc
1744_580_1_f(1>857)  GAAAACCCTAGCTACAGTAAC
1744_590_2_r(1>482)  GAAAACCCTAGCTACAGTAACGGGTACAAAATATAGAACGCTAATGGGCCAGCACACATACGGCCCATAATACTCTAACACCCCTC
WT_590_2_r(1>494)    GAAAACCCTAGCTACAGTAACGGGTACAAAATATAGAACGCTAATGGGCCAGCACACATACGGCCCATAATACTCTAACACCCCTC
1744_590_r(1>250)    GAAAACCCTAGCTACAGTAACGGGTACAAAATATAGAACGCTAATGGGCCAGCACACATACGGCCCATAATACTCTAACACCCCTC
WT_an_590_r(1>202)   GAAAACCCTAGCTACAGTAACGGGTACAAAATATAGAACGCTAATGGGCCAGCACACATACGGCCCATAATACTCTAACACCCCTT
```

```
                     11270     11280     11290     11300     11310     11320     11330     11340     11350
                     GTAGCTGAAACTCTAGCCACTGCAGATGTTGAGATTGGACCGAAACTCCGTAAATACGGTCGTCGAAAGCCCCTtGGTGAAGACGT
PH207(1>13023)       gtagctgaaactctagccactgcagatgttgagattggaccgaaactccgtaaatacggtcgtcgaaagccccttggtgaagacgt
B73(1>13011)         gtagctgaaactctagccactgcagatgttgagattggaccgaaactccgtaaatacggtcgtcgaaagccccttggtgaagacgt
1744_590_2_r(1>482)  GTAGCTGAAACTCTAGCCAC
WT_590_2_r(1>494)    GTAGCTGAAACTCTAGCCACTGCAGATGTTGAGATTGGACCGAAACTCCGTAAATACGGTCGTCGAAAGCCCC
1744_590_r(1>250)    GTAGCTGAAACTCTAGCCACTGCAGATGTTGAGATTGGACCGAAACTCCGTAAATACGGTCGTCGAAAGCCCCT-GGTGAAGA
WT_an_590_r(1>202)   GTAGCTGAAACTCTAGCCACTGCAGATGTTGAGATTGGACCGAAACTCCGTAAATACGGTCGTCGAAAGCCC
```

11360 11370 11380 11390 11400 11410 11420 11430

CGGCGAACTGGGAGGCGGTCGGGACGTGGAGGACGCGAACAGCTCCACAGGTGACTCGCTCGCGGACAAAGTGAAGATCGATCTCG

PH207(1>13023) → cggcgaactgggaggcggtcgggacgtggaggacgcgaacagctccacaggtgactcgctcgcggacaaagtgaagatcgatctcg
B73(1>13011) → cggcgaactgggaggcggtcgggacgtggaggacgcgaacagctccacaggtgactcgctcgcggacaaagtgaagatcgatctcg

11440 11450 11460 11470 11480 11490 11500 11510 11520

ACATGCTTCATGCGCTGGTGTTGGACGGGGTTGGTGGAGAGGTAGACGGCACTGACGTTGTCGCAGAAGATGAGGGTACTCCGTGC

PH207(1>13023) → acatgcttcatgcgctggtgttggacggggttggtggagaggtagacggcactgacgttgtcgcagaagatgagggtactccgtgc
B73(1>13011) → acatgcttcatgcgctggtgttggacggggttggtggagaggtagacggcactgacgttgtcgcagaagatgagggtactccgtgc

11530 11540 11550 11560 11570 11580 11590 11600 11610

TAGCGGACTGTGGAGCTCCTAGAGCAGCTGATGGAGCCAGGTCGCCTCAGCCACGTCGTTTGCCACGGCGCGGTACTCGGCCTCGG

PH207(1>13023) → tagcggactgtggagctcctagagcagctgatggagccaggtcgcctcagccacgtcgtttgccacggcgcggtactcggcctcgg
B73(1>13011) → tagcggactgtggagctcctagagcagctgatggagccaggtcgcctcagccacgtcgtttgccacggcgcggtactcggcctcgg

11620 11630 11640 11650 11660 11670 11680 11690

CGCTGGAGCGGGAGACGACTGGTTGGCGCTTCAATGACCAGGAGACGAGGTTGTCCTCTAGGAAGACGGCATAGCCCGAGGTGGAC

PH207(1>13023) → cgctggagcgggagacgactggttggcgcttcaatgaccaggagacgaggttgtcctctaggaagacggcatagcccgaggtggac
B73(1>13011) → cgctggagcgggagacgactggttggcgcttcaatgaccaggagacgaggttgtcctctaggaagacggcatagcccgaggtggac

11700 11710 11720 11730 11740 11750 11760 11770 11780

TGGCACGTGTCTGGACAGCCCGCCTAGTCGACATCGGTGTAGACACGAAGCTCCGTAGTCGAGGAGCGGCGGAGAAGGAGCCCGAA

PH207(1>13023) → tggcacgtgtctggacagcccgcctagtcgacatcggtgtagacacgaagctccgtagtcgaggagcggcggagaaggagcccgaa
B73(1>13011) → tggcacgtgtctggacagcccgcctagtcgacatcggtgtagacacgaagctccgtagtcgaggagcggcggagaaggagcccgaa

11790 11800 11810 11820 11830 11840 11850 11860

GTCGATGGTGCCACACAAGTACCATAGGATGCGCTTCATCGCGGTGAGGTGCGGCTCCCGAGGGTCGTGCATGTGAAGGCACACTT

PH207(1>13023) → gtcgatggtgccacacaagtaccataggatgcgcttcatcgcggtgaggtgcggctcccgagggtcgtgcatgtgaaggcacactt
B73(1>13011) → gtcgatggtgccacacaagtaccataggatgcgcttcatcgcggtgaggtgcggctcccgagggtcgtgcatgtgaaggcacactt

11870 11880 11890 11900 11910 11920 11930 11940 11950

GCTGGACGGCGTAAGCGATGTCGGGCCGGGTGAAGATGAGGTACTGCAGGGCCCCAGCAAGGCTGCGGTAGGTGGTCGGATCAGCG

PH207(1>13023) → gctggacggcgtaagcgatgtcgggccgggtgaagatgaggtactgcagggccccagcaaggctgcggtaggtggtcggatcagcg
B73(1>13011) → gctggacggcgtaagcgatgtcgggccgggtgaagatgaggtactgcagggccccagcaaggctgcggtaggtggtcggatcagcg

11960 11970 11980 11990 12000 12010 12020 12030 12040

ACGGGGGGCCTGCGGCAGAGACCTTGCCCTGCGTGTCCACTGGGGTCACGCAGGGCTTGCACTCGGCCATGCCAGYGAGCTCGAGG

PH207(1>13023) → acggggggcctgcggcagagaccttgccctgcgtgtccactggggtcacgcagggcttgcactcggccatgccagtgagctcgagg
B73(1>13011) → acggggggcctgcggcagagaccttgccctgcgtgtccactggggtcacgcagggcttgcactcggccatgccagcgagctcgagg

12050 12060 12070 12080 12090 12100 12110 12120

AGGTCGACAGTGTACTACCGTTGGTGAAGGAACATGCCCTGGGGCCTGCGCTCGACGGTGACACCTAGGAAGTGGTGGAGGGCCCC

PH207(1>13023) → aggtcgacagtgtactaccgttggtgaaggaacatgccctggggcctgcgctcgacggtgacacctaggaagtggtggagggcccc
B73(1>13011) → aggtcgacagtgtactaccgttggtgaaggaacatgccctggggcctgcgctcgacggtgacacctaggaagtggtggagggcccc

12130 12140 12150 12160 12170 12180 12190 12200 12210

GAGGTCCTTCATCGCAAACTCCTGCTGGAGGCAGGAGATGATGTGACGCAGGAGCCCAGGGGAGGAGGCGGTGAGCACGATATCAT

PH207(1>13023) → gaggtccttcatcgcaaactcctgctggaggcaggagatgatgtgacgcaggagcccaggggaggaggcggtgagcacgatatcat
B73(1>13011) → gaggtccttcatcgcaaactcctgctggaggcaggagatgatgtgacgcaggagcccaggggaggaggcggtgagcacgatatcat

12220 12230 12240 12250 12260 12270 12280 12290

CGACGTAGAGGAGGAGGTACGCAGTATCTGGACCGCGACGGAGGATGAACAGGGACATGTCCGACTTGGCCTCGATGAAACCCTGC

PH207(1>13023) → cgacgtagaggaggaggtacgcagtatctggaccgcgacggaggatgaacagggacatgtccgacttggcctcgatgaaaccctgc
B73(1>13011) → cgacgtagaggaggaggtacgcagtatctggaccgcgacggaggatgaacagggacatgtccgacttggcctcgatgaaaccctgc

12300 12310 12320 12330 12340 12350 12360 12370 12380

GAAAACAAGAAAGTGGCGAAGCGACTGTACCAAGCCCGGGGGGCCTGCTTGAGGCTGTAGAGGGACTTGTTGAGCTTGCAGACTAT

PH207(1>13023) gaaaacaagaaagtggcgaagcgactgtaccaagcccggggggcctgcttgaggctgtagagggacttgttgagcttgcagactat
B73(1>13011) gaaaacaagaaagtggcgaagcgactgtaccaagcccggggggcctgcttgaggctgtagagggacttgttgagcttgcagactat

12390 12400 12410 12420 12430 12440 12450 12460 12470

GTCGGGGTGAGCAGGGTCGACAAACCCAACAGGCTGAGTGCAGTAGACCGTCTTCGTCAGGGTGCCATGGAGGAAGGCGTTCATCA

PH207(1>13023) gtcggggtgagcagggtcgacaaacccaacaggctgagtgcagtagaccgtcttcgtcagggtgccatggaggaaggcgttcatca
B73(1>13011) gtcggggtgagcagggtcgacaaacccaacaggctgagtgcagtagaccgtcttcgtcagggtgccatggaggaaggcgttcatca

12480 12490 12500 12510 12520 12530 12540 12550

CGTCGAGTTGGTGGACTGACCAGTCCCTGGATAGAGCCAGAGCCAGAACAGTCCAGACGGTGGCAGGCTTGACCACCGGACTGAAG

PH207(1>13023) cgtcgagttggtggactgaccagtccctggatagagccagagccagaacagtccagacggtggcaggcttgaccaccggactgaag
B73(1>13011) cgtcgagttggtggactgaccagtccctggatagagccagagccagaacagtccagacggtggcaggcttgaccaccggactgaag

12560 12570 12580 12590 12600 12610 12620 12630 12640

GTCTCATCGTAGTCGACTCYGGGGCGTTGGGTGAAGCCCCAGAGCACATAGCGGGCCTGTTACCGCTCTAGGGACCCGTCGTCGTT

PH207(1>13023) gtctcatcgtagtcgactctggggcgttgggtgaagccccagagcacatagcgggcctgttaccgctctagggacccgtcgtcgtt
B73(1>13011) gtctcatcgtagtcgactccggggcgttgggtgaagccccagagcacatagcgggcctgttaccgctctagggacccgtcgtcgtt

12650 12660 12670 12680 12690 12700 12710 12720

CAACTTCAGCTTGAAGATCCACTTCCTGGTGACCACGCTGGCGCCGAGGGGAGGGGCACCAGGTCCCAGGTGTGGTTAGCCTGGAG

PH207(1>13023) caacttcagcttgaagatccacttcctggtgaccacgctggcgccgaggggaggggcaccaggtcccaggtgtggttagcctggag
B73(1>13011) caacttcagcttgaagatccacttcctggtgaccacgctggcgccgaggggaggggcaccaggtcccaggtgtggttagcctggag

12730 12740 12750 12760 12770 12780 12790 12800 12810

AGTCGTGTACTCTACTTCCATGGCACGTCGCCACTGTGGGTTGGCAAGCGCACCACGGACAGTCGTGGGCATCGGCGAAAGAGCCG

PH207(1>13023) agtcgtgtactctacttccatggcacgtcgccactgtgggttggcaagcgcaccacggacagtcgtgggcatcggcgaaagagccg
B73(1>13011) agtcgtgtactctacttccatggcacgtcgccactgtgggttggcaagcgcaccacggacagtcgtgggcatcggcgaaagagccg

12820 12830 12840 12850 12860 12870 12880 12890 12900

GAGAAGAGGTTGCGGAGAGAATCAGGCGATCAGGTGCCCGGAGGACACCGACCACGCACCGGGTGACCATCGGGTGGATGTGTCGA

PH207(1>13023) gagaagaggttgcggagagaatcaggcgatcaggtgcccggaggacaccgaccacgcaccgggtgaccatcgggtggatgtgtcga
B73(1>13011) gagaagaggttgcggagagaatcaggcgatcaggtgcccggaggacaccgaccacgcaccgggtgaccatcgggtggatgtgtcga

12910 12920 12930 12940 12950 12960 12970 12980

GGATCCCGGTGGACGACGACAGGGTGATAGGCCGGGGCTCAGCGCGAGAGCGAGCTGGGGCACCACGTCGACCACGACACTGGTAC

PH207(1>13023) ggatcccggtggacgacgacagggtgataggccggggctcagcgcgagagcgagctggggcaccacgtcgaccacgacactggtac
B73(1>13011) ggatcccggtggacgacgacagggtgataggccggggctcagcgcgagagcgagctggggcaccacgtcgaccacgacactggtac

12990 13000 13010 13020 13030 13040 13050 13060 13070

GTCTGGATGGGGTCGGTGTAGCGAGACGACGGGGCAGTGTTCGTTGGTGCGGGAGGCACCGGAGCCGCACGGGGCTATGCGGGAGA

PH207(1>13023) gtctggatggggtcggtgtagcgagacgacggggcagtgttcgttggtgcgggaggcaccggagccgcacggggctatgcgggaga
B73(1>13011) gtctggatggggtcggtgtagcgagacgacggggcagtgttcgttggtgcgggaggcaccggagccgcacggggctatgcgggaga

13080

CGGCGGGGCCGCGC

PH207(1>13023) cggcggggccgcgc
B73(1>13011) cggcggggccgcgc
