# Supplementary material for: Combining next‐generation sequencing and progeny testing for rapid identification of induced recessive and dominant mutations in maize M2 individuals
Source: Plant J. 2019 Jul 12;100(4):851–62. doi: 10.1111/tpj.14431 (PMC6899793; doi:10.1111/tpj.14431)
Supplement: Supplementary file 3 — Figure S3. Alignment and strategy view of the re‐sequenced w2 gene. [file TPJ-100-851-s003.pdf]

Figure S3: Alignment and strategy view of the re-sequenced *w2* gene in the *pale green* (1754) and *PALE GREEN* (WT) mutants, PH207_w2_flanking & PH207_ws (Chr10:140702976-140735202 & 140707976-140724202)

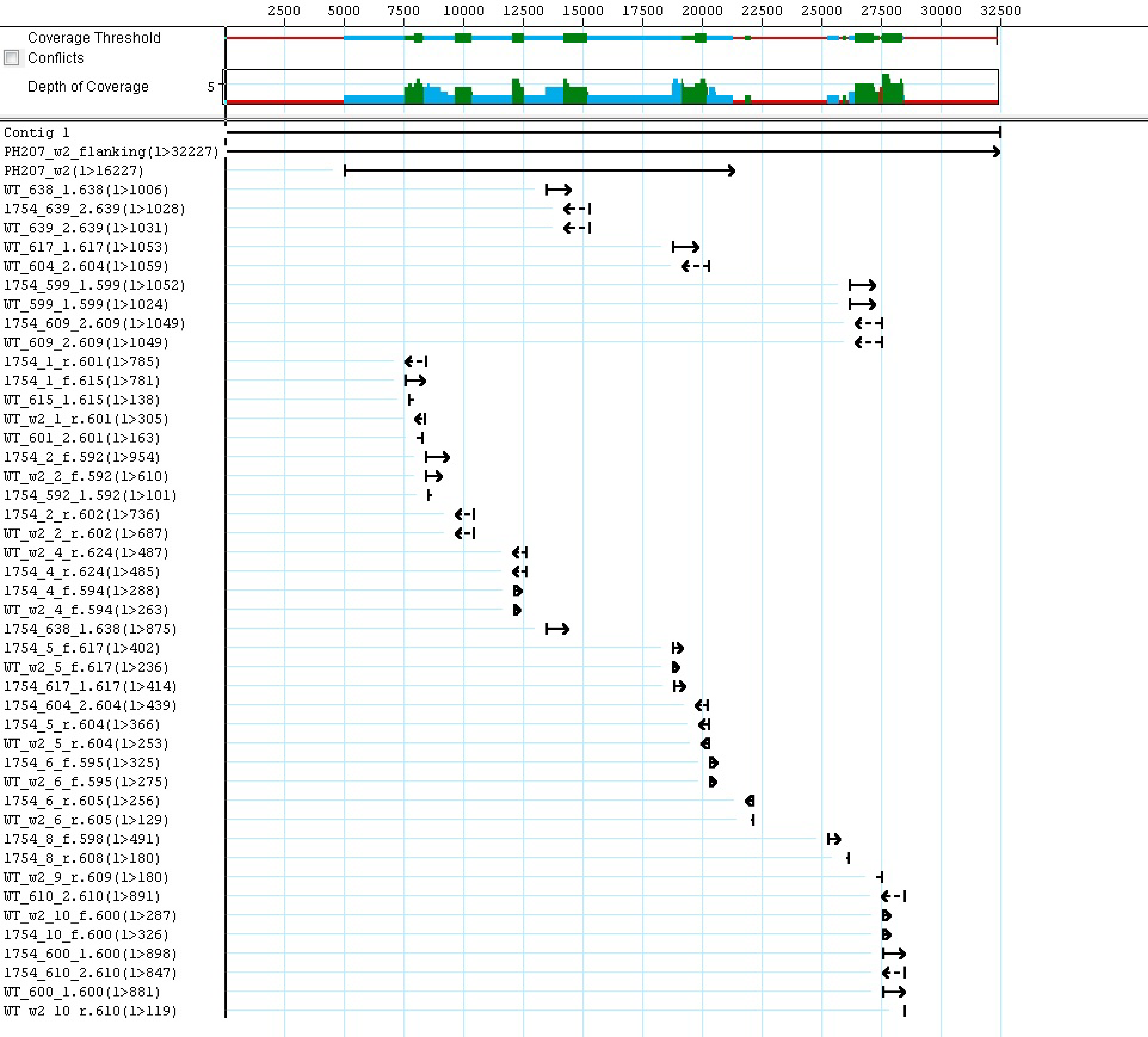

2500
5000
7500
10000
12500
15000
17500
20000
22500
25000
27500
30000
32500
Coverage Threshold
Conflicts
Depth of Coverage
5
Contig 1
PH207_w2_flanking(1>32227)
PH207_w2(1>16227)
WT_638_1.638(1>1006)
1754_639_2.639(1>1028)
WT_639_2.639(1>1031)
WT_617_1.617(1>1053)
WT_604_2.604(1>1059)
1754_599_1.599(1>1052)
WT_599_1.599(1>1024)
1754_609_2.609(1>1049)
WT_609_2.609(1>1049)
1754_1_r.601(1>785)
1754_1_f.615(1>781)
WT_615_1.615(1>138)
WT_w2_1_r.601(1>305)
WT_601_2.601(1>163)
1754_2_f.592(1>954)
WT_w2_2_f.592(1>610)
1754_592_1.592(1>101)
1754_2_r.602(1>736)
WT_w2_2_r.602(1>687)
WT_w2_4_r.624(1>487)
1754_4_r.624(1>485)
1754_4_f.594(1>288)
WT_w2_4_f.594(1>263)
1754_638_1.638(1>875)
1754_5_f.617(1>402)
WT_w2_5_f.617(1>236)
1754_617_1.617(1>414)
1754_604_2.604(1>439)
1754_5_r.604(1>366)
WT_w2_5_r.604(1>253)
1754_6_f.595(1>325)
WT_w2_6_f.595(1>275)
1754_6_r.605(1>256)
WT_w2_6_r.605(1>129)
1754_8_f.598(1>491)
1754_8_r.608(1>180)
WT_w2_9_r.609(1>180)
WT_610_2.610(1>891)
WT_w2_10_f.600(1>287)
1754_10_f.600(1>326)
1754_600_1.600(1>898)
1754_610_2.610(1>847)
WT_600_1.600(1>881)
WT_w2_10_r.610(1>119)

10 20 30 40 50 60 70
gactgctcgcacgcgtgcgggNctcgcacgcgtgcggggcgtgctcgccgtgcagccgcgtcatggtcagcttcaagt
PH207_w2_flanking(1>32227) → gactgctcgcacgcgtgcgggnctcgcacgcgtgcggggcgtgctcgccgtgcagccgcgtcatggtcagcttcaagt

80 90 100 110 120 130 140 150
gctccgccgccgagccgctgccgtgccccatggtgtaccgctgcatgtgcaggggcaagtgctacccggtgccgtcca
PH207_w2_flanking(1>32227) → gctccgccgccgagccgctgccgtgccccatggtgtaccgctgcatgtgcaggggcaagtgctacccggtgccgtcca

160 170 180 190 200 210 220 230
gctgacggtgatgcatgcttgccgatcgagttgccgccgcgcgcgcgggatgtggaaatgacgatcgattaccctgct
PH207_w2_flanking(1>32227) → gctgacggtgatgcatgcttgccgatcgagttgccgccgcgcgcgcgggatgtggaaatgacgatcgattaccctgct

240 250 260 270 280 290 300 310
aataaagctagctagctatagcacttacttagggcctgtttggtagagctccggctcctttaaaaatagctccaactc
PH207_w2_flanking(1>32227) → aataaagctagctagctatagcacttacttagggcctgtttggtagagctccggctcctttaaaaatagctccaactc

320 330 340 350 360 370 380 390
cggctcgtgtgtaagagcagctctttcacgggagccactattttgtttacaaaacatttggcaaaatgactctttcaa
PH207_w2_flanking(1>32227) → cggctcgtgtgtaagagcagctctttcacgggagccactattttgtttacaaaacatttggcaaaatgactctttcaa

400 410 420 430 440 450 460
tacgttttatttatgagtgtgttagacatttgcacaaataccttgcaggcgtgctgtgtgaatcgaaacagaaatcaa
PH207_w2_flanking(1>32227) → tacgttttatttatgagtgtgttagacatttgcacaaataccttgcaggcgtgctgtgtgaatcgaaacagaaatcaa

470 480 490 500 510 520 530 540
atagttgggaaacgagatgagcgaggggattgggaggacctggcaagtgaggagatgagtgaagcagctagtaaaatc
PH207_w2_flanking(1>32227) → atagttgggaaacgagatgagcgaggggattgggaggacctggcaagtgaggagatgagtgaagcagctagtaaaatc

550 560 570 580 590 600 610 620
acgcaagcagatagatgattgctgctccccgccagcgtgctctcgcctgggtgcctccgtccttgccgtccgacgagc
PH207_w2_flanking(1>32227) → acgcaagcagatagatgattgctgctccccgccagcgtgctctcgcctgggtgcctccgtccttgccgtccgacgagc

630 640 650 660 670 680 690 700
gccaggctagagaatgcgtcacggagccgcttggagccacttttctgtgctctggctcctgtccccgaattggctcct
PH207_w2_flanking(1>32227) → gccaggctagagaatgcgtcacggagccgcttggagccacttttctgtgctctggctcctgtccccgaattggctcct

710 720 730 740 750 760 770 780
gctcctgagatggagctgtttttggcaccttgtttgggagggctccggcaaggagccagagctggagctgctcgggag
PH207_w2_flanking(1>32227) → gctcctgagatggagctgtttttggcaccttgtttgggagggctccggcaaggagccagagctggagctgctcgggag

790 800 810 820 830 840 850
ccctcccaaacagccccttaattggaccttaatttatgcatgtggagagttggagacttcttgatccgtttgggctaa
PH207_w2_flanking(1>32227) → ccctcccaaacagccccttaattggaccttaatttatgcatgtggagagttggagacttcttgatccgtttgggctaa

860 870 880 890 900 910 920 930
tcacggtgttacggggcacccccttttttttttgtaaatgacgatgggtgatgagtcgttacctagcgctagctagtgtg
PH207_w2_flanking(1>32227) → tcacggtgttacggggcacccccttttttttttgtaaatgacgatgggtgatgagtcgttacctagcgctagctagtgtg

940 950 960 970 980 990 1000 1010
tgtgttgtggagaagatcgagatcctgatgacgtttaatttggtagatgtacgaggcttcttttggtaatttaagtac
PH207_w2_flanking(1>32227) → tgtgttgtggagaagatcgagatcctgatgacgtttaatttggtagatgtacgaggcttcttttggtaatttaagtac

1020 1030 1040 1050 1060 1070 1080 1090

tctctccatttcaatttataattgttaacgtttttttatcaaatttgaccgactcgtcttatttaaaaagtttcataa

PH207_w2_flanking(1>32227) → tctctccatttcaatttataattgttaacgtttttttatcaaatttgaccgactcgtcttatttaaaaagtttcataa

1100 1110 1120 1130 1140 1150 1160 1170

ttattattaatttttaccgtgatagcgtgtagcatataatgtgctttaattacaatgtttattttttattttttcat

PH207_w2_flanking(1>32227) → ttattattaatttttaccgtgatagcgtgtagcatataatgtgctttaattacaatgtttattttttattttttcat

1180 1190 1200 1210 1220 1230 1240

aaaaaatgaataagacaattcagtcaaacttaataaaaaatcaaatgaattataaattataacagaatgagtcaaaaa

PH207_w2_flanking(1>32227) → aaaaaatgaataagacaattcagtcaaacttaataaaaaatcaaatgaattataaattataacagaatgagtcaaaaa

1250 1260 1270 1280 1290 1300 1310 1320

ctttgatgtataggcatattgggtgacactacttggggtggggtgggtgctggcaggagtggaggtatggcttcagaa

PH207_w2_flanking(1>32227) → ctttgatgtataggcatattgggtgacactacttggggtggggtgggtgctggcaggagtggaggtatggcttcagaa

1330 1340 1350 1360 1370 1380 1390 1400

agtagaccgaaaacaccttatggttacacacttttcaatggctatgactaacagactatatataggctttgtttggga

PH207_w2_flanking(1>32227) → agtagaccgaaaacaccttatggttacacacttttcaatggctatgactaacagactatatataggctttgtttggga

1410 1420 1430 1440 1450 1460 1470 1480

tattccagcttcaaaaaaaaagtagaattagttaagcagatcattagatgatatagggaattgtggagttgatgttgt

PH207_w2_flanking(1>32227) → tattccagcttcaaaaaaaaagtagaattagttaagcagatcattagatgatatagggaattgtggagttgatgttgt

1490 1500 1510 1520 1530 1540 1550 1560

aagcctttaaaatatatttagattagtcattttgtttattatttgtattattttttttaaaactatttaaattggtatc

PH207_w2_flanking(1>32227) → aagcctttaaaatatatttagattagtcattttgtttattatttgtattattttttttaaaactatttaaattggtatc

1570 1580 1590 1600 1610 1620 1630

ataaactaaactacaacgcatattggagctgaaatcttgagccatcttaaccacctctatagttctgtaaaacactat

PH207_w2_flanking(1>32227) → ataaactaaactacaacgcatattggagctgaaatcttgagccatcttaaccacctctatagttctgtaaaacactat

1640 1650 1660 1670 1680 1690 1700 1710

tttacactatagacgacattgtttagacggtgaagtgtgaagttttaaatagaagataaataattaagacttgtagat

PH207_w2_flanking(1>32227) → tttacactatagacgacattgtttagacggtgaagtgtgaagttttaaatagaagataaataattaagacttgtagat

1720 1730 1740 1750 1760 1770 1780 1790

agccttaaccttccttacatgaaataagagacgttactagtgctaataaatcaacaatcatgtacctaaaaaataatg

PH207_w2_flanking(1>32227) → agccttaaccttccttacatgaaataagagacgttactagtgctaataaatcaacaatcatgtacctaaaaaataatg

1800 1810 1820 1830 1840 1850 1860 1870

agaactaacctttcaactaacgcatgtgcccaccatcttacacgattaaaactttaggaggtccaccaacctaattaa

PH207_w2_flanking(1>32227) → agaactaacctttcaactaacgcatgtgcccaccatcttacacgattaaaactttaggaggtccaccaacctaattaa

1880 1890 1900 1910 1920 1930 1940 1950

acaaaactctcaaaattattcactgaggctctgtttagaatccctaaatctaatagttagccaactaatatattatta

PH207_w2_flanking(1>32227) → acaaaactctcaaaattattcactgaggctctgtttagaatccctaaatctaatagttagccaactaatatattatta

1960 1970 1980 1990 2000 2010 2020

gctaggtttaaccaactaataaactaattattagcagtgagatagttaataattacttgcgatattaactgagagttt

PH207_w2_flanking(1>32227) → gctaggtttaaccaactaataaactaattattagcagtgagatagttaataattacttgcgatattaactgagagttt

2030 2040 2050 2060 2070 2080 2090 2100
gtagaactaaataactaaattttagtagctaattattaactattagttctatactagttaggtacccgtgcgttgcga
PH207_w2_flanking(1>32227) → gtagaactaaataactaaattttagtagctaattattaactattagttctatactagttaggtacccgtgcgttgcga

2110 2120 2130 2140 2150 2160 2170 2180
cagctcataaattctcacaaattacaaatctaaatgtcatagcccaatacatacatgttaggtgtgtttatatgccat
PH207_w2_flanking(1>32227) → cagctcataaattctcacaaattacaaatctaaatgtcatagcccaatacatacatgttaggtgtgtttatatgccat

2190 2200 2210 2220 2230 2240 2250 2260
ggaaaaatcatttctatttctgcacattgtctcaaaactctaaaaagctataacaaaagaacatatcatagtgttcat
PH207_w2_flanking(1>32227) → ggaaaaatcatttctatttctgcacattgtctcaaaactctaaaaagctataacaaaagaacatatcatagtgttcat

2270 2280 2290 2300 2310 2320 2330 2340
gaaaaaaggacacgttgcggacggtaaataaatttgttcattttgaagctatagaaagatgcatttatttatcatttt
PH207_w2_flanking(1>32227) → gaaaaaaggacacgttgcggacggtaaataaatttgttcattttgaagctatagaaagatgcatttatttatcatttt

2350 2360 2370 2380 2390 2400 2410
gaagctatagaaagatgcaaagtacatggtaaaagccattctatgtgaagaatgtgaaggttttgtgtacttcgatct
PH207_w2_flanking(1>32227) → gaagctatagaaagatgcaaagtacatggtaaaagccattctatgtgaagaatgtgaaggttttgtgtacttcgatct

2420 2430 2440 2450 2460 2470 2480 2490
gcatttggagcaatgtatttgccaccatctctatccagatcaaattacagcgtgaactcagtgcagtcattaatctgg
PH207_w2_flanking(1>32227) → gcatttggagcaatgtatttgccaccatctctatccagatcaaattacagcgtgaactcagtgcagtcattaatctgg

2500 2510 2520 2530 2540 2550 2560 2570
caagcaagtattaataaacatgacaatatcaacaaaaaagtaagtgttaatttttttatttagactgattgtaatta
PH207_w2_flanking(1>32227) → caagcaagtattaataaacatgacaatatcaacaaaaaagtaagtgttaatttttttatttagactgattgtaatta

2580 2590 2600 2610 2620 2630 2640 2650
ctgcacacatcaagcgtcccctaaagtacatgaacaatacaactaattgaacaaagtgaaagttataagaacaaatct
PH207_w2_flanking(1>32227) → ctgcacacatcaagcgtcccctaaagtacatgaacaatacaactaattgaacaaagtgaaagttataagaacaaatct

2660 2670 2680 2690 2700 2710 2720 2730
taatcgtggtatctctcatataatcgtattcagagcgcttcagctggagttgcaaaagatgggggaatcaaggaaaag
PH207_w2_flanking(1>32227) → taatcgtggtatctctcatataatcgtattcagagcgcttcagctggagttgcaaaagatgggggaatcaaggaaaag

2740 2750 2760 2770 2780 2790 2800
aatgtccttcctcgcatcctgcgatatcaagattgaatccagtcatgagataccgtcaaaaataaactatagaaaaag
PH207_w2_flanking(1>32227) → aatgtccttcctcgcatcctgcgatatcaagattgaatccagtcatgagataccgtcaaaaataaactatagaaaaag

2810 2820 2830 2840 2850 2860 2870 2880
gttatcataacttacaaacttccttcatcagtctgtaaaaaaagaacaggaagtaaaggaatatcacatgcaccctac
PH207_w2_flanking(1>32227) → gttatcataacttacaaacttccttcatcagtctgtaaaaaaagaacaggaagtaaaggaatatcacatgcaccctac

2890 2900 2910 2920 2930 2940 2950 2960
atgttcaccatatggctataccgtgcaataatttagttgtttcctatcttagtaaatgaatacattgaagctcacaaa
PH207_w2_flanking(1>32227) → atgttcaccatatggctataccgtgcaataatttagttgtttcctatcttagtaaatgaatacattgaagctcacaaa

2970 2980 2990 3000 3010 3020 3030 3040
taacataaatatcttgatcaatagaccaactctatttggttttcatcttaacaaacaacaagatggtttgttacctgc
PH207_w2_flanking(1>32227) → taacataaatatcttgatcaatagaccaactctatttggttttcatcttaacaaacaacaagatggtttgttacctgc

```
                                    3050      3060      3070      3080      3090      3100      3110      3120
                                    agaccatgattctttgtgtgatccttaccacccctaattgcaagacctctagctcaaaaatagcaaaacactccccac
PH207_w2_flanking(1>32227)    →     agaccatgattctttgtgtgatccttaccacccctaattgcaagacctctagctcaaaaatagcaaaacactccccac

                                    3130      3140      3150      3160      3170      3180      3190
                                    tccatgcaagcagcaaacacagtacaacaagtaaacaaagcaagtggacaacgacacttcatgggcagatttaggaat
PH207_w2_flanking(1>32227)    →     tccatgcaagcagcaaacacagtacaacaagtaaacaaagcaagtggacaacgacacttcatgggcagatttaggaat

                                    3200      3210      3220      3230      3240      3250      3260      3270
                                    cttcccttagcttaggtcgacccctgacaatctctagatgtccctaatcccaatcgagcatcgtagttatgaaccaac
PH207_w2_flanking(1>32227)    →     cttcccttagcttaggtcgacccctgacaatctctagatgtccctaatcccaatcgagcatcgtagttatgaaccaac

                                    3280      3290      3300      3310      3320      3330      3340      3350
                                    acttttcttcccttctagagtcaatgtggcccagctcaactgaagttaaaggagggtttggaccctgcatagagccca
PH207_w2_flanking(1>32227)    →     acttttcttcccttctagagtcaatgtggcccagctcaactgaagttaaaggagggtttggaccctgcatagagccca

                                    3360      3370      3380      3390      3400      3410      3420      3430
                                    aacatgaaacaattcattcagctataacaaaaaaatcggatgcaactggcaataatcaaaatttatgaataattacaa
PH207_w2_flanking(1>32227)    →     aacatgaaacaattcattcagctataacaaaaaaatcggatgcaactggcaataatcaaaatttatgaataattacaa

                                    3440      3450      3460      3470      3480      3490      3500      3510
                                    accttgtatattctattgattcgaaaatgcatgcaaacaaaattattcttgtcatgagtacactacgcaggttcatgt
PH207_w2_flanking(1>32227)    →     accttgtatattctattgattcgaaaatgcatgcaaacaaaattattcttgtcatgagtacactacgcaggttcatgt

                                    3520      3530      3540      3550      3560      3570      3580
                                    ttgctcttgtgttggcagtttacctaccctagtatagttttgaaatatgaatataatggtaagatataagtggaccag
PH207_w2_flanking(1>32227)    →     ttgctcttgtgttggcagtttacctaccctagtatagttttgaaatatgaatataatggtaagatataagtggaccag

                                    3590      3600      3610      3620      3630      3640      3650      3660
                                    tcatgtcaattttattgctagtttaaaaagtgtttcttttcaaggaatctctctactcaacaacatttggttaggaca
PH207_w2_flanking(1>32227)    →     tcatgtcaattttattgctagtttaaaaagtgtttcttttcaaggaatctctctactcaacaacatttggttaggaca

                                    3670      3680      3690      3700      3710      3720      3730      3740
                                    acagacttgcaaataatatttttgtttgatgttgaacaaaggttagggtacgaatgccaatatgcaaggatctgtaaa
PH207_w2_flanking(1>32227)    →     acagacttgcaaataatatttttgtttgatgttgaacaaaggttagggtacgaatgccaatatgcaaggatctgtaaa

                                    3750      3760      3770      3780      3790      3800      3810      3820
                                    taaatacatctgacgttgctttctaattttgacaggttggtgtcatacactacttttcgttcttgaaagctagagagg
PH207_w2_flanking(1>32227)    →     taaatacatctgacgttgctttctaattttgacaggttggtgtcatacactacttttcgttcttgaaagctagagagg

                                    3830      3840      3850      3860      3870      3880      3890      3900
                                    aactcggctgtgtacctataagctgtggtgaggcctcacaaaggattagctgcaacggtttccttatgtttctatgtt
PH207_w2_flanking(1>32227)    →     aactcggctgtgtacctataagctgtggtgaggcctcacaaaggattagctgcaacggtttccttatgtttctatgtt

                                    3910      3920      3930      3940      3950      3960      3970
                                    tccagattgccatgaactgaaccattcagttatatcaccgattagatgaagaaacaattgcaagcagcccaaaggctc
PH207_w2_flanking(1>32227)    →     tccagattgccatgaactgaaccattcagttatatcaccgattagatgaagaaacaattgcaagcagcccaaaggctc

                                    3980      3990      4000      4010      4020      4030      4040      4050
                                    ttccgcaaacaaatcaggcaaatggttaatagtaagacagctataaacatactccactcaataacataaaaacatact
PH207_w2_flanking(1>32227)    →     ttccgcaaacaaatcaggcaaatggttaatagtaagacagctataaacatactccactcaataacataaaaacatact
```

```
                               4060      4070      4080      4090      4100      4110      4120      4130
                               cgacagcaggcaaccttgagcacagttagggtagaataaaggtctagaacacacttctccacatcaagtagaagcaca
PH207_w2_flanking(1>32227) →   cgacagcaggcaaccttgagcacagttagggtagaataaaggtctagaacacacttctccacatcaagtagaagcaca

                               4140      4150      4160      4170      4180      4190      4200      4210
                               cggccaaaattagaagaaaaaaacaagagagagagtcgtaccacagctccccgtcgtaccacacggccaaaataaatt
PH207_w2_flanking(1>32227) →   cggccaaaattagaagaaaaaaacaagagagagagtcgtaccacagctccccgtcgtaccacacggccaaaataaatt

                               4220      4230      4240      4250      4260      4270      4280      4290
                               tcatgttacatccaatgatatcagaaaaaaaatattatcactttagaagcactcggtgccatgtttttgtctttggaa
PH207_w2_flanking(1>32227) →   tcatgttacatccaatgatatcagaaaaaaaatattatcactttagaagcactcggtgccatgtttttgtctttggaa

                               4300      4310      4320      4330      4340      4350      4360
                               tagaaggagtgctgaaacgatgtcagcaactaactgggcaaaagaatactggtaccatgcattgtggcctctgcacct
PH207_w2_flanking(1>32227) →   tagaaggagtgctgaaacgatgtcagcaactaactgggcaaaagaatactggtaccatgcattgtggcctctgcacct

                               4370      4380      4390      4400      4410      4420      4430      4440
                               caaccatgcctgcatcgtgcatccatccaccggagacctcagcccatcatccttcccttccctgatcccctcccctttt
PH207_w2_flanking(1>32227) →   caaccatgcctgcatcgtgcatccatccaccggagacctcagcccatcatccttcccttccctgatcccctcccctttt

                               4450      4460      4470      4480      4490      4500      4510      4520
                               cttcttgtatataaatcatccaaactgctccttgtccgcgcaagagcaagcagtgatgactaatccgactccctagct
PH207_w2_flanking(1>32227) →   cttcttgtatataaatcatccaaactgctccttgtccgcgcaagagcaagcagtgatgactaatccgactccctagct

                               4530      4540      4550      4560      4570      4580      4590      4600
                               cccacactaccaccatcccattcccatccaaccagctttaaaaagcccccaccaggccaccactacatcctgcgctgc
PH207_w2_flanking(1>32227) →   cccacactaccaccatcccattcccatccaaccagctttaaaaagcccccaccaggccaccactacatcctgcgctgc

                               4610      4620      4630      4640      4650      4660      4670      4680
                               acgccaagctcgcctcgcctctgttcagctcacggtggaagaggaggcgacatgccgacagcacacgaaaatctgttc
PH207_w2_flanking(1>32227) →   acgccaagctcgcctcgcctctgttcagctcacggtggaagaggaggcgacatgccgacagcacacgaaaatctgttc

                               4690      4700      4710      4720      4730      4740      4750
                               gccagtcgagggtcggattgaggatgaagacagcgccaacgaacagcacgtgcattaggagtaccatgaatctgagtc
PH207_w2_flanking(1>32227) →   gccagtcgagggtcggattgaggatgaagacagcgccaacgaacagcacgtgcattaggagtaccatgaatctgagtc

                               4760      4770      4780      4790      4800      4810      4820      4830
                               ctagcgtcgtcctcctctatgcgcaaaagcctccagaggcccactcaaacccccatggacacaacaaagcaccgaa
PH207_w2_flanking(1>32227) →   ctagcgtcgtcctcctctatgcgcaaaagcctccagaggcccactcaaacccccatggacacaacaaagcaccgaa

                               4840      4850      4860      4870      4880      4890      4900      4910
                               tcgagcaaaatatcaggtacagagaaagatctgcttttgacaggcgttgcgacgttaggtaccctatcaggatgaaga
PH207_w2_flanking(1>32227) →   tcgagcaaaatatcaggtacagagaaagatctgcttttgacaggcgttgcgacgttaggtaccctatcaggatgaaga

                               4920      4930      4940      4950      4960      4970      4980      4990
                               caggcacaagagcaaaatccaatccagtttatctgcaaaagcctctgttcgNctcgccgagtaggagaccgcgacccc
PH207_w2_flanking(1>32227) →   caggcacaagagcaaaatccaatccagtttatctgcaaaagcctctgttcgnctcgccgagtaggagaccgcgacccc

                               5000      5010      5020      5030      5040      5050      5060      5070
                               ggtgttccATGGAGAGCAGCTCGTTGGGGGAGTGGACTGCGGGGGAGAGGAGATCTGCTGGGACCGCTATTCCATGGA
PH207_w2_flanking(1>32227) →   ggtgttccatggagagcagctcgttgggggagtggactgcgggggagaggagatctgctgggaccgctattccatgga
PH207_w2(1>16227)          →           atggagagcagctcgttgggggagtggactgcgggggagaggagatctgctgggaccgctattccatgga
```

```
                          5080      5090      5100      5110      5120      5130      5140
                          GAGCAGCGTGGGCAGGATGGGCTTGCCCGGTCGTGGATCTCGCCGAGGAGGAGACCGCGACCCCGGCCTTCCGTGGAG
PH207_w2_flanking(1>32227)  gagcagcgtgggcaggatgggcttgcccggtcgtggatctcgccgaggaggagaccgcgaccccggccttccgtggag
PH207_w2(1>16227)           gagcagcgtgggcaggatgggcttgcccggtcgtggatctcgccgaggaggagaccgcgaccccggccttccgtggag

                         5150      5160      5170      5180      5190      5200      5210      5220
                          AGCAGCGTGGGCGGGATGGGCGGGCTCAGCCGTGGATCGCGCGGAGGAGTGGATCGCGGTGAGGGATTGGGTCGCCTT
PH207_w2_flanking(1>32227)  agcagcgtgggcgggatgggcgggctcagccgtggatcgcgcggaggagtggatcgcggtgagggattgggtcgcctt
PH207_w2(1>16227)           agcagcgtgggcgggatgggcgggctcagccgtggatcgcgcggaggagtggatcgcggtgagggattgggtcgcctt

                           5230      5240      5250      5260      5270      5280      5290      5300
                          CCAGGATTTGCAGTCTGTTCAAAGCCGCGCGTAGAAGGAAGCCCACGCACCATGGATTGCGGCATCGAGCAGAGTGGA
PH207_w2_flanking(1>32227)  ccaggatttgcagtctgttcaaagccgcgcgtagaaggaagcccacgcaccatggattgcggcatcgagcagagtgga
PH207_w2(1>16227)           ccaggatttgcagtctgttcaaagccgcgcgtagaaggaagcccacgcaccatggattgcggcatcgagcagagtgga

                             5310      5320      5330      5340      5350      5360      5370      5380
                          TCCGCGTGGTGGTCGGGTCGGGGTCGATTCGGAGTGGACCGCGCGCTTCCTCCCATGGTGGTCGGGTCGAGGTCGGCT
PH207_w2_flanking(1>32227)  tccgcgtggtggtcgggtcggggtcgattcggagtggaccgcgcgcttcctcccatggtggtcgggtcgaggtcggct
PH207_w2(1>16227)           tccgcgtggtggtcgggtcggggtcgattcggagtggaccgcgcgcttcctcccatggtggtcgggtcgaggtcggct

                               5390      5400      5410      5420      5430      5440      5450      5460
                          TGGATTGCGGCGCCGTTGGTGGGGGAGGAAGCCACGCGTAGAAGGAAGGAAGCCGCGTAGAAGGAAGTTGGCCGCGCG
PH207_w2_flanking(1>32227)  tggattgcggcgccgttggtgggggaggaagccacgcgtagaaggaaggaagccgcgtagaaggaagttggccgcgcg
PH207_w2(1>16227)           tggattgcggcgccgttggtgggggaggaagccacgcgtagaaggaaggaagccgcgtagaaggaagttggccgcgcg

                                 5470      5480      5490      5500      5510      5520      5530
                          TCCGCGCGCGTGTTGGGTGGTCGGCCGCGCGTAGAAGGAACGAGCGGGGGCACGGTGATGCCTCGGTGTGGACGCCCT
PH207_w2_flanking(1>32227)  tccgcgcgcgtgttgggtggtcggccgcgcgtagaaggaacgagcgggggcacggtgatgcctcggtgtggacgccct
PH207_w2(1>16227)           tccgcgcgcgtgttgggtggtcggccgcgcgtagaaggaacgagcgggggcacggtgatgcctcggtgtggacgccct

                         5540      5550      5560      5570      5580      5590      5600      5610
                          CGTTCCGCTCTTAAGGAGTAGTGGGAGGAAGTGGATGCTGCCGGATGGGAAAGGGCGCGGTCTCCGGCAGGGAAGTGG
PH207_w2_flanking(1>32227)  cgttccgctcttaaggagtagtgggaggaagtggatgctgccggatgggaaagggcgcggtctccggcagggaagtgg
PH207_w2(1>16227)           cgttccgctcttaaggagtagtgggaggaagtggatgctgccggatgggaaagggcgcggtctccggcagggaagtgg

                           5620      5630      5640      5650      5660      5670      5680      5690
                          GGAAGGGCGGGAATGGCGTCGGTGGTGGGGAATGGCGTGGAGGGCGGAGCAAGGGAGGAAGGCGACCGGGAAGAGGGC
PH207_w2_flanking(1>32227)  ggaagggcgggaatggcgtcggtggtggggaatggcgtggagggcggagcaagggaggaaggcgaccgggaagagggc
PH207_w2(1>16227)           ggaagggcgggaatggcgtcggtggtggggaatggcgtggagggcggagcaagggaggaaggcgaccgggaagagggc

                             5700      5710      5720      5730      5740      5750      5760      5770
                          GGGGATGGGGGCCGGCCCTCGCGGTGATGGGGGCGATGACATTTTTGGGAAGGTGTGGCGGAGAACGCGCTGCCGCGG
PH207_w2_flanking(1>32227)  ggggatgggggccggccctcgcggtgatgggggcgatgacatttttgggaaggtgtggcggagaacgcgctgccgcgg
PH207_w2(1>16227)           ggggatgggggccggccctcgcggtgatgggggcgatgacatttttgggaaggtgtggcggagaacgcgctgccgcgg

                               5780      5790      5800      5810      5820      5830      5840      5850
                          CGAGATTAGCGAGGGATGAAGACAGGGGCGTGCGTGATGTGCTGGCAGCGAGATTAGCGGGGAGAGAGCAGAGCGGAG
PH207_w2_flanking(1>32227)  cgagattagcgagggatgaagacaggggcgtgcgtgatgtgctggcagcgagattagcggggagagagcagagcggag
PH207_w2(1>16227)           cgagattagcgagggatgaagacaggggcgtgcgtgatgtgctggcagcgagattagcggggagagagcagagcggag

                                 5860      5870      5880      5890      5900      5910      5920
                          CCNCCACAAACAATATCAGGCGTAGGTAGCGTACCTCGATCAGAGAGGCGGGAGCTGGGCTCAGCAACAGAGGAGGAC
PH207_w2_flanking(1>32227)  ccnccacaaacaatatcaggcgtaggtagcgtacctcgatcagagaggcgggagctgggctcagcaacagaggaggac
PH207_w2(1>16227)           ccnccacaaacaatatcaggcgtaggtagcgtacctcgatcagagaggcgggagctgggctcagcaacagaggaggac
```

```
                              5930      5940      5950      5960      5970      5980      5990      6000
                              GCCAGCCATGGATCTCGCCGAGTAGGAGACCGCGACCCCGGTGTTCCATGGAGAGCAGCTCGTTGGGGGAGTGGACTG
PH207_w2_flanking(1>32227)  → gccagccatggatctcgccgagtaggagaccgcgaccccggtgttccatggagagcagctcgttgggggagtggactg
PH207_w2(1>16227)           → gccagccatggatctcgccgagtaggagaccgcgaccccggtgttccatggagagcagctcgttgggggagtggactg

                              6010      6020      6030      6040      6050      6060      6070      6080
                              CGGGGGAGAGGAGATCTGGTGGGACCGCTATTCCATGGAGAGCAGCGTGGGCAGGATGGGCTTGCCCGGCCGTGGATC
PH207_w2_flanking(1>32227)  → cgggggagaggagatctggtgggaccgctattccatggagagcagcgtgggcaggatgggcttgcccggccgtggatc
PH207_w2(1>16227)           → cgggggagaggagatctggtgggaccgctattccatggagagcagcgtgggcaggatgggcttgcccggccgtggatc

                              6090      6100      6110      6120      6130      6140      6150      6160
                              TCGCCGAGGAGGAGACCCGGCCGTGGATCTCGCCGAGGAGGAGACCGCGACCCCGGCCTTCCGTGGAGAGCAGCGTGG
PH207_w2_flanking(1>32227)  → tcgccgaggaggagacccggccgtggatctcgccgaggaggagaccgcgaccccggccttccgtggagagcagcgtgg
PH207_w2(1>16227)           → tcgccgaggaggagacccggccgtggatctcgccgaggaggagaccgcgaccccggccttccgtggagagcagcgtgg

                              6170      6180      6190      6200      6210      6220      6230      6240
                              GCGGGANAGCCGGGCGGGGGCACGCTAGGTGGTGGACGCCCTCGCTCTGCTCATATGGGATAGTAGAGATGTTCCCAA
PH207_w2_flanking(1>32227)  → gcggganagccgggcgggggcacgctaggtggtggacgccctcgctctgctcatatgggatagtagagatgttcccaa
PH207_w2(1>16227)           → gcggganagccgggcgggggcacgctaggtggtggacgccctcgctctgctcatatgggatagtagagatgttcccaa

                              6250      6260      6270      6280      6290      6300      6310
                              CAAGATTTAGACGTCTAAACTCGGACTAACAAACAACCGTAACCCCCACAGGAACCGATGTGTATTTGTATACTCGCA
PH207_w2_flanking(1>32227)  → caagatttagacgtctaaactcggactaacaaacaaccgtaacccccacaggaaccgatgtgtatttgtatactcgca
PH207_w2(1>16227)           → caagatttagacgtctaaactcggactaacaaacaaccgtaacccccacaggaaccgatgtgtatttgtatactcgca

                              6320      6330      6340      6350      6360      6370      6380      6390
                              CAAAAGAAACAATACACAAAGAGAATATATATAGTGCACATGCGTCTTTGGTACTAGGCTGGTAACGGTACAACCATT
PH207_w2_flanking(1>32227)  → caaaagaaacaatacacaaagagaatatatatagtgcacatgcgtctttggtactaggctggtaacggtacaaccatt
PH207_w2(1>16227)           → caaaagaaacaatacacaaagagaatatatatagtgcacatgcgtctttggtactaggctggtaacggtacaaccatt

                              6400      6410      6420      6430      6440      6450      6460      6470
                              TCATACATGTATGTGTATCATAAAAAAAAATCTACCCACCGGGTTACCCATATATGTTTGCGGGTATAAAATCTTACC
PH207_w2_flanking(1>32227)  → tcatacatgtatgtgtatcataaaaaaaaatctacccaccgggttacccatatatgtttgcgggtataaaatcttacc
PH207_w2(1>16227)           → tcatacatgtatgtgtatcataaaaaaaaatctacccaccgggttacccatatatgtttgcgggtataaaatcttacc

                              6480      6490      6500      6510      6520      6530      6540      6550
                              CATATTCATACTCGCATGAGTAATTTTACCTGTCGGTTAACCTGTACCCGCTAGAAAAGTCTTAAATTTTAATATATA
PH207_w2_flanking(1>32227)  → catattcatactcgcatgagtaattttacctgtcggttaacctgtacccgctagaaaagtcttaaattttaatatata
PH207_w2(1>16227)           → catattcatactcgcatgagtaattttacctgtcggttaacctgtacccgctagaaaagtcttaaattttaatatata

                              6560      6570      6580      6590      6600      6610      6620      6630
                              CCAATGACTCAAAATATTAAATAAACATACACATATGATTACATAACTTGTTTGCTAGACAAATGATTGCTAGAGAAG
PH207_w2_flanking(1>32227)  → ccaatgactcaaaatattaaataaacatacacatatgattacataacttgtttgctagacaaatgattgctagagaag
PH207_w2(1>16227)           → ccaatgactcaaaatattaaataaacatacacatatgattacataacttgtttgctagacaaatgattgctagagaag

                              6640      6650      6660      6670      6680      6690      6700
                              GGTTTAATTTTAGCTAATATAGGCTCCATTAGATTGTTATAGTGGTATTGATGCGAGTATATTTTACCTATGGGTAGA
PH207_w2_flanking(1>32227)  → ggtttaattttagctaatataggctccattagattgttatagtggtattgatgcgagtatattttacctatgggtaga
PH207_w2(1>16227)           → ggtttaattttagctaatataggctccattagattgttatagtggtattgatgcgagtatattttacctatgggtaga

                              6710      6720      6730      6740      6750      6760      6770      6780
                              CGGGTCGGTAATACCACCTCGTACCTATACTCGTCTACCCAACGGATAGGTGTTCTTACATGTTAATATACCCGCGGG
PH207_w2_flanking(1>32227)  → cgggtcggtaataccacctcgtacctatactcgtctacccaacggataggtgttcttacatgttaatatacccgcggg
PH207_w2(1>16227)           → cgggtcggtaataccacctcgtacctatactcgtctacccaacggataggtgttcttacatgttaatatacccgcggg
```

```
                                    6790      6800      6810      6820      6830      6840      6850      6860
                                 TAGAGAAATCATCATGTACCTGCTTTATATCAGGTAAAACCCGTCGAGTAGTTGGGTTTTGATTACCCATTGCACCTC
PH207_w2_flanking(1>32227)   →   tagagaaatcatcatgtacctgctttatatcaggtaaaacccgtcgagtagttgggttttgattacccattgcacctc
PH207_w2(1>16227)            →   tagagaaatcatcatgtacctgctttatatcaggtaaaacccgtcgagtagttgggttttgattacccattgcacctc

                                      6870      6880      6890      6900      6910      6920      6930      6940
                                 TATTTGGTACATATATGATACACATGAGTTAACAAAATCCATAAAAAATATAGAAAATCATGCATATTCTTGATATGT
PH207_w2_flanking(1>32227)   →   tatttggtacatatatgatacacatgagttaacaaaatccataaaaaatatagaaaatcatgcatattcttgatatgt
PH207_w2(1>16227)            →   tatttggtacatatatgatacacatgagttaacaaaatccataaaaaatatagaaaatcatgcatattcttgatatgt

                                        6950      6960      6970      6980      6990      7000      7010      7020
                                 TACTCAAATTTATTATACATATGTGCACAAGATATATTCTCACACATAAAAGGTCAATATTGCCGGACACGCTGGTGG
PH207_w2_flanking(1>32227)   →   tactcaaatttattatacatatgtgcacaagatatattctcacacataaaaggtcaatattgccggacacgctggtgg
PH207_w2(1>16227)            →   tactcaaatttattatacatatgtgcacaagatatattctcacacataaaaggtcaatattgccggacacgctggtgg

                                          7030      7040      7050      7060      7070      7080      7090
                                 CGGCCACGGTGTGCATGTAGCAGAAAAGTCCTGGTCCTGGGACACCACCAACGCGTACGCTGCCCCCGTGTTCCTGGT
PH207_w2_flanking(1>32227)   →   cggccacggtgtgcatgtagcagaaaagtcctggtcctgggacaccaccaacgcgtacgctgcccccgtgttcctggt
PH207_w2(1>16227)            →   cggccacggtgtgcatgtagcagaaaagtcctggtcctgggacaccaccaacgcgtacgctgcccccgtgttcctggt

                                 7100      7110      7120      7130      7140      7150      7160      7170
                                 CCCTGTCTGTCTCCCTTTGGCGGCCACGGCACGAGAGGCGGAGGAGGCACAAAAATACGTAATACGTTTCAGGATTCC
PH207_w2_flanking(1>32227)   →   ccctgtctgtctccctttggcggccacggcacgagaggcggaggaggcacaaaaatacgtaatacgtttcaggattcc
PH207_w2(1>16227)            →   ccctgtctgtctccctttggcggccacggcacgagaggcggaggaggcacaaaaatacgtaatacgtttcaggattcc

                                   7180      7190      7200      7210      7220      7230      7240      7250
                                 AGTCCACCGTTGGCGCTGGCGTAAACCCTGCCTCTCCCTATCCCCCAAGTCCGCGGCGCCCAGCTGCCCCTGCCTCCT
PH207_w2_flanking(1>32227)   →   agtccaccgttggcgctggcgtaaaccctgcctctccctatcccccaagtccgcggcgcccagctgcccctgcctcct
PH207_w2(1>16227)            →   agtccaccgttggcgctggcgtaaaccctgcctctccctatcccccaagtccgcggcgcccagctgcccctgcctcct

                                      7260      7270      7280      7290      7300      7310      7320      7330
                                 CCTACCGCNNNNNNNNNNNNNNNNNNNNNNNNNNNNNNNNNNNNNNNNNNNNNNNNNNNNNNNNNNNNNNNNNNNNNNN
PH207_w2_flanking(1>32227)   →   cctaccgcnnnnnnnnnnnnnnnnnnnnnnnnnnnnnnnnnnnnnnnnnnnnnnnnnnnnnnnnnnnnnnnnnnnnnn
PH207_w2(1>16227)            →   cctaccgcnnnnnnnnnnnnnnnnnnnnnnnnnnnnnnnnnnnnnnnnnnnnnnnnnnnnnnnnnnnnnnnnnnnnnn

                                        7340      7350      7360      7370      7380      7390      7400      7410
                                 NNNNNNNNNNNNNNNNNNNNNNNNNNNNNNNNNNNNNNNNNNNNNNNNNNNNNNNNNNNNNNNNNNNNNNNNNNNNNN
PH207_w2_flanking(1>32227)   →   nnnnnnnnnnnnnnnnnnnnnnnnnnnnnnnnnnnnnnnnnnnnnnnnnnnnnnnnnnnnnnnnnnnnnnnnnnnnnn
PH207_w2(1>16227)            →   nnnnnnnnnnnnnnnnnnnnnnnnnnnnnnnnnnnnnnnnnnnnnnnnnnnnnnnnnnnnnnnnnnnnnnnnnnnnnn

                                          7420      7430      7440      7450      7460      7470      7480
                                 NNNNNNNNNNNNNNNNNNNNNNNNNNNNNNNNNNNNNTGCTCTGCGCCTCCCTGGGCGCCCTCGCCCTTCCGCCCCCG
PH207_w2_flanking(1>32227)   →   nnnnnnnnnnnnnnnnnnnnnnnnnnnnnnnnnnnnntgctctgcgcctccctgggcgccctcgcccttccgcccccg
PH207_w2(1>16227)            →   nnnnnnnnnnnnnnnnnnnnnnnnnnnnnnnnnnnnntgctctgcgcctccctgggcgccctcgcccttccgcccccg

                                 7490      7500      7510      7520      7530      7540      7550      7560
                                 CCGCCCCCGCCGCGGCCGCTCCATGATGTCGCCGTTCACTGGCGCCAGAAGGTAGTGCCCCCTACTGTTTAGCGGTTC
PH207_w2_flanking(1>32227)   →   ccgcccccgccgcggccgctccatgatgtcgccgttcactggcgccagaaggtagtgccccctactgtttagcggttc
PH207_w2(1>16227)            →   ccgcccccgccgcggccgctccatgatgtcgccgttcactggcgccagaaggtagtgccccctactgtttagcggttc
1754_1_r.601(1>785)          ←                                      TCACTGGCGCCAGAAGGTAGTGCCCCCTACTGTTTAGCGGTTC
1754_1_f.615(1>781)          →                                                                          GCGGTTC

                                   7570      7580      7590      7600      7610      7620      7630      7640
                                 AATGCTCCTTCCGGTGACCTTAGAGCTTGTGGTGCCGCGTGATTTGGTCGTAGCGGCGATGATGATAAAACATTTGAC
PH207_w2_flanking(1>32227)   →   aatgctccttccggtgaccttagagcttgtggtgccgcgtgatttggtcgtagcggcgatgatgataaaacatttgac
PH207_w2(1>16227)            →   aatgctccttccggtgaccttagagcttgtggtgccgcgtgatttggtcgtagcggcgatgatgataaaacatttgac
1754_1_r.601(1>785)          ←   AATGCTCCTTCCGGTGACCTTAGAGCTTGTGGTGCCGCGTGATTTGGTCGTAGCGGCGATGATGATAAAACATTTGAC
1754_1_f.615(1>781)          →   -ATGCTCCTTCCGGTGACCTTAGAGCTTGTGGTGCCGCGTGATTTGGTCGTAGCGGCGATGATGATAAAACATTTGAC
```

```
                              7650      7660      7670      7680      7690      7700      7710      7720
                              TAGATAGAGGTTTGCACTTTGGTTTGCTCGATGGTGTGTTGATATTCTGGTGATTACTGTCGTGTCGTGGGAACCTTG
PH207_w2_flanking(1>32227) →  tagatagaggtttgcactttggtttgctcgatggtgtgttgatattctggtgattactgtcgtgtcgtgggaaccttg
PH207_w2(1>16227)          →  tagatagaggtttgcactttggtttgctcgatggtgtgttgatattctggtgattactgtcgtgtcgtgggaaccttg
1754_1_r.601(1>785)        ←  TAGATAGAGGTTTGCACTTTGGTTTGCTCGATGGTGTGTTGATATTCTGGTGATTACTGTCGTGTCGTGGGAACCTTG
1754_1_f.615(1>781)        →  TAGATAGAGGTTTGCACTTTGGTTTGCTCGATGGTGTGTTGATATTCTGGTGATTACTGTCGTGTCGTGGGAACCTTG
WT_615_1.615(1>138)        →                              GATGGTGTGTTGATATTCTGGTGATTACTGTCGTGTCGTGGGAACCTTG

                                7730      7740      7750      7760      7770      7780      7790      7800
                              CGTGTCGTGTTCTGACTTGATGCTAGTAATGTAATCTCTATATATGAATCAATCAGGGGGATGAGTGAGCCAAATATT
PH207_w2_flanking(1>32227) →  cgtgtcgtgttctgacttgatgctagtaatgtaatctctatatatgaatcaatcagggggatgagtgagccaaatatt
PH207_w2(1>16227)          →  cgtgtcgtgttctgacttgatgctagtaatgtaatctctatatatgaatcaatcagggggatgagtgagccaaatatt
1754_1_r.601(1>785)        ←  CGTGTCGTGTTCTGACTTGATGCTAGTAATGTAATCTCTATATATGAATCAATCAGGGGGATGAGTGAGCCAAATATT
1754_1_f.615(1>781)        →  CGTGTCGTGTTCTGACTTGATGCTAGTAATGTAATCTCTATATATGAATCAATCAGGGGGATGAGTGAGCCAAATATT
WT_615_1.615(1>138)        →  CGTGTCGTGTTCTGACTTGATGCTAGTAATGTAATCTCTATATATGAATCAATCAGGGGGATGAGTGAGCCAAATATT

                                  7810      7820      7830      7840      7850      7860      7870
                              GTCTGTTCTGTAATTTGCCTCTGCACCCATAGCCCGCAGGATAAAGCTATTGCTTTTTGATGGTGGTGTTTTTGCTGA
PH207_w2_flanking(1>32227) →  gtctgttctgtaatttgcctctgcacccatagcccgcaggataaagctattgctttttgatggtggtgtttttgctga
PH207_w2(1>16227)          →  gtctgttctgtaatttgcctctgcacccatagcccgcaggataaagctattgctttttgatggtggtgtttttgctga
1754_1_r.601(1>785)        ←  GTCTGTTCTGTAATTTGCCTCTGCACCCATAGCCCGCAGGATAAAGCTATTGCTTTTTGATGGTGGTGTTTTTGCTGA
1754_1_f.615(1>781)        →  GTCTGTTCTGTAATTTGCCTCTGCACCCATAGCCCGCAGGATAAAGCTATTGCTTTTTGATGGTGGTGTTTTTGCTGA
WT_615_1.615(1>138)        →  GTCTGTTCTGT

                              7880      7890      7900      7910      7920      7930      7940      7950
                              ACTTCGTGAGCGAACAACATGTGATGATGTTTCTCCAGAGTATCCTACCTATTGGCCTAATATCAGTCATTAAAAAAT
PH207_w2_flanking(1>32227) →  acttcgtgagcgaacaacatgtgatgatgtttctccagagtatcctacctattggcctaatatcagtcattaaaaaat
PH207_w2(1>16227)          →  acttcgtgagcgaacaacatgtgatgatgtttctccagagtatcctacctattggcctaatatcagtcattaaaaaat
1754_1_r.601(1>785)        ←  ACTTCGTGAGCGAACAACATGTGATGATGTTTCTCCAGAGTATCCTACCTATTGGCCTAATATCAGTCATTAAAAAAT
1754_1_f.615(1>781)        →  ACTTCGTGAGCGAACAACATGTGATGATGTTTCTCCAGAGTATCCTACCTATTGGCCTAATATCAGTCATTAAAAAAT
WT_w2_1_r.601(1>305)       ←                                                                          AAAAAT

                                7960      7970      7980      7990      8000      8010      8020      8030
                              GTTCATCAGTTTGATGCTTCGCCTCTGACTTTTAGAATGGCTGATTGCCAGGCAGGAGTACTCCCATAGCTCAGTCCT
PH207_w2_flanking(1>32227) →  gttcatcagtttgatgcttcgcctctgacttttagaatggctgattgccaggcaggagtactcccatagctcagtcct
PH207_w2(1>16227)          →  gttcatcagtttgatgcttcgcctctgacttttagaatggctgattgccaggcaggagtactcccatagctcagtcct
1754_1_r.601(1>785)        ←  GTTCATCAGTTTGATGCTTCGCCTCTGACTTTTAGAATGGCTGATTGCCAGGCAGGAGTACTCCCATAGCTCAGTCCT
1754_1_f.615(1>781)        →  GTTCATCAGTTTGATGCTTCGCCTCTGACTTTTAGAATGGCTGATTGCCAGGCAGGAGTACTCCCATAGCTCAGTCCT
WT_w2_1_r.601(1>305)       ←  GTTCATCAGTTTGATGTTTCGCCTCTGACTTTTAGAATGGCTGATTGCCAGGCAGGAGTACTCCCATAGCTCAGTCCT

                                8040      8050      8060      8070      8080      8090      8100      8110
                              TGGAATTCAAGACAACCGAGCTCTTAAGCTTCCAGTATGTGTCAACTTTAATCTGCAAAGTGGCGGCGCACAAGAATG
PH207_w2_flanking(1>32227) →  tggaattcaagacaaccgagctcttaagcttccagtatgtgtcaactttaatctgcaaagtggcggcgcacaagaatg
PH207_w2(1>16227)          →  tggaattcaagacaaccgagctcttaagcttccagtatgtgtcaactttaatctgcaaagtggcggcgcacaagaatg
1754_1_r.601(1>785)        ←  TGGAATTCAAGACAACCGAGCTCTTAAGCTTCCAGTATGTGTCAACTTTAATCTGCAAAGTGGCGGCGCACAAGAATG
1754_1_f.615(1>781)        →  TGGAATTCAAGACAACCGAGCTCTTAAGCTTCCAGTATGTGTCAACTTTAATCTGCAAAGTGGCGGCGCACAAGAATG
WT_w2_1_r.601(1>305)       ←  TGGAATTCAAGACAACCGAGCTCTTAAGCTTCCAGTATGTGTCAACTTTAATCTGCAAAGTGGCGGCGCACAAGAATG
WT_601_2.601(1>163)        ←    GAATTCAAGACAACCGAGCTCTTAAGCTTCCAGTATGTGTCAACTTTAATCTGCAAAGTGGCGGCGCACAAGAATG

                                8120      8130      8140      8150      8160      8170      8180      8190
                              GGCTGACGAGAGCAGGAGGTTATCATTAAGCAAAGCCAGCAATAGTAATGGTTCCACTCATCTAGGGTCTGGTATTTT
PH207_w2_flanking(1>32227) →  ggctgacgagagcaggaggttatcattaagcaaagccagcaatagtaatggttccactcatctagggtctggtatttt
PH207_w2(1>16227)          →  ggctgacgagagcaggaggttatcattaagcaaagccagcaatagtaatggttccactcatctagggtctggtatttt
1754_1_r.601(1>785)        ←  GGCTGACGAGAGCAGGAGGTTATCATTAAGCAAAGCCAGCAATAGTAATGGTTCCACTCATCTAGGGTCTGGTATTTT
1754_1_f.615(1>781)        →  GGCTGACGAGAGCAGGAGGTTATCATTAAGCAAAGCCAGCAATAGTAATGGTTCCACTCATCTAGGGTCTGGTATTTT
WT_w2_1_r.601(1>305)       ←  GGCTGACGAGAGCAGGAGGTTATCATTAAGCAAAGCCAGCAATAGTAATGGTTCCACTCATCTAGGGTCTGGTATTTT
WT_601_2.601(1>163)        ←  GGCTGACGAGAGCAGGAGGTTATCATTAAGCAAAGCCAGCAATAGTAATGGTTCCACTCATCTAGGGTGTGGTATTTT

                                  8200      8210      8220      8230      8240      8250      8260
                              CCATCATGAACCCTTGGAAGATTTCAAGAGCTCCAACCAATCTCTATTGCACAGTGTAAGACAAAGAATGTCACCGAA
PH207_w2_flanking(1>32227) →  ccatcatgaacccttggaagatttcaagagctccaaccaatctctattgcacagtgtaagacaaagaatgtcaccgaa
PH207_w2(1>16227)          →  ccatcatgaacccttggaagatttcaagagctccaaccaatctctattgcacagtgtaagacaaagaatgtcaccgaa
1754_1_r.601(1>785)        ←  CCATCATGAACCCTTGGAAGATTTCAAGAGCTCCAACCAATCTCTATTGCACAGTGTAAGACAAAGAATGTCACCGAA
1754_1_f.615(1>781)        →  CCATCATGAACCCTTGGAAGATTTCAAGAGCTCCAACCAATCTCTATTGCACAGTGTAAGACAAAGAATGTCACCGAA
WT_w2_1_r.601(1>305)       ←  CCATCATTATGATGTCGCCG-TT-CACTGGCGCTGATGAAAGGCT-TT-CGCGATGCC-G-CAGGTAATG-C
```

```
                              8200      8210      8220      8230      8240      8250      8260
                              CCATCATGAACCCTTGGAAGATTTCAAGAGCTCCAACCAATCTCTATTGCACAGTGTAAGACAAAGAATGTCACCGAA
WT_601_2.601(1>163)           CCATCATCA

                              8270      8280      8290      8300      8310      8320      8330      8340
                              TTCTCTTGCGAATAGGCATGTCAACACGGAATTGGCAAAGCATCATGCGATTAAGCATGCCGCAGTTGCTGTGTCAGC
PH207_w2_flanking(1>32227)    ttctcttgcgaataggcatgtcaacacggaattggcaaagcatcatgcgattaagcatgccgcagttgctgtgtcagc
PH207_w2(1>16227)             ttctcttgcgaataggcatgtcaacacggaattggcaaagcatcatgcgattaagcatgccgcagttgctgtgtcagc
1754_1_r.601(1>785)           TTCTCTTGCGAATAGGCATGTCAACACGGAAT-GGCAAAGC
1754_1_f.615(1>781)           TTCTCTTGCGAATAGGCATGTCAACACGGAATTGGCAAAGCATCATGCGATTAAGCATGCCGCAGTTGCTGTG

                              8350      8360      8370      8380      8390      8400      8410      8420
                              ACTGACCAGTGTTGTTAACGATGACCTTAAATCAGTGAAGAGGCCTAAGGAATCTGAAGTGGAGGTTCATTGGTCTAA
PH207_w2_flanking(1>32227)    actgaccagtgttgttaacgatgaccttaaatcagtgaagaggcctaaggaatctgaagtggaggttcattggtctaa
PH207_w2(1>16227)             actgaccagtgttgttaacgatgaccttaaatcagtgaagaggcctaaggaatctgaagtggaggttcattggtctaa
1754_2_f.592(1>954)           GAGGCCTAAGGAATCTGAAGTGGAGGTTCATTGGTCTAA
WT_w2_2_f.592(1>610)          G-AGTGGAGGTTCATTGGTCTAA

                              8430      8440      8450      8460      8470      8480      8490      8500
                              TGGTCCCAAGTTTCATTCTCCTATTCCGAAAATCTCTGAAGCTGAAACAAGCCTGCGATTCGATGAAAATGCTACAGA
PH207_w2_flanking(1>32227)    tggtcccaagtttcattctcctattccgaaaatctctgaagctgaaacaagcctgcgattcgatgaaaatgctacaga
PH207_w2(1>16227)             tggtcccaagtttcattctcctattccgaaaatctctgaagctgaaacaagcctgcgattcgatgaaaatgctacaga
1754_2_f.592(1>954)           TGGTCCCAAGTTTCATTCTCCTATTCCGAAAATCTCTGAAGCTGAAACAAGCCTGCGATTCGATGAAAATGCTACAGA
WT_w2_2_f.592(1>610)          TGGTCCCAAGTTTCATTCTCCTATTCCGAAAATCTCTGAAGCTGAAACAAGCCTGCGATTCGATGAAAATGCTACAGA
1754_592_1.592(1>101)         TGAGAAGCTGAAACAAGCCTGCGATTCGATGAAAATGCTACAGA

                              8510      8520      8530      8540      8550      8560      8570      8580
                              TGGCTATGCTAAAGATGTGAATGAATGCTCACCTGAGGAGACAGTACAACCCTCTCCAACTAGAGCGCCCTCATCCCA
PH207_w2_flanking(1>32227)    tggctatgctaaagatgtgaatgaatgctcacctgaggagacagtacaaccctctccaactagagcgccctcatccca
PH207_w2(1>16227)             tggctatgctaaagatgtgaatgaatgctcacctgaggagacagtacaaccctctccaactagagcgccctcatccca
1754_2_f.592(1>954)           TGGCTATGCTAAAGATGTGAATGAATGCTCACCTGAGGAGACAGTACAACCCTCTCCAACTAGAGCGCCCTCATCCCA
WT_w2_2_f.592(1>610)          TGGCTATGCTAAAGATGTGAATGAATGCTCACCTGAGGAGACAGTACAACCCTCTCCAACTAGAGCGCCCTCATCCCA
1754_592_1.592(1>101)         TGGCTATGCTAAAGATGTGAATGAATGCTCACCTGAGGAGACAGTACAACCCTCTCC

                              8590      8600      8610      8620      8630      8640      8650
                              GGAGTCTCTAGATGCACGGAAGGCACTCAGTACCATATATGGCAAAGTTCTGGTGGTTGACAATGTCATGTCAGCTAG
PH207_w2_flanking(1>32227)    ggagtctctagatgcacggaaggcactcagtaccatatatggcaaagttctggtggttgacaatgtcatgtcagctag
PH207_w2(1>16227)             ggagtctctagatgcacggaaggcactcagtaccatatatggcaaagttctggtggttgacaatgtcatgtcagctag
1754_2_f.592(1>954)           GGAGTCTCTAGATGCACGGAAGGCACTCAGTACCATATATGGCAAAGTTCTGGTGGTTGACAATGTCATGTCAGCTAG
WT_w2_2_f.592(1>610)          GGAGTCTCTAGATGCACGGAAGGCACTCAGTACCATATATGGCAAAGTTCTGGTGGTTGACAATGTCATGTCAGCTAG

                              8660      8670      8680      8690      8700      8710      8720      8730
                              GAGTGTTGTTCAACTGCTTACTACGAAGTACAGAAATTATATTCATGCATGTGACACAGAGGTAAGGCATTAAAGCCT
PH207_w2_flanking(1>32227)    gagtgttgttcaactgcttactacgaagtacagaaattatattcatgcatgtgacacagaggtaaggcattaaagcct
PH207_w2(1>16227)             gagtgttgttcaactgcttactacgaagtacagaaattatattcatgcatgtgacacagaggtaaggcattaaagcct
1754_2_f.592(1>954)           GAGTGTTGTTCAACTGCTTACTACGAAGTACAGAAATTATATTCATGCATGTGACACAGAGGTAAGGCATTAAAGCCT
WT_w2_2_f.592(1>610)          GAGTGTTGTTCAACTGCTTACTACGAAGTACAGAAATTATATTCATGCATGTGACACAGAGGTAAGGCATTAAAGCCT

                              8740      8750      8760      8770      8780      8790      8800      8810
                              TTTACATCTACGTAAAATGCTTTATGTATTTATATTTATTTACATCGGTCATGGCTTGTTACCTAAATCTAATGTTGA
PH207_w2_flanking(1>32227)    tttacatctacgtaaaatgctttatgtatttatatttatttacatcggtcatggcttgttacctaaatctaatgttga
PH207_w2(1>16227)             tttacatctacgtaaaatgctttatgtatttatatttatttacatcggtcatggcttgttacctaaatctaatgttga
1754_2_f.592(1>954)           TTTACATCTACGTAAAATGCTTTATGTATTTATATTTATTTACATCGGTCATGGCTTGTTACCTAAATCTAATGTTGA
WT_w2_2_f.592(1>610)          TTTACATCTACGTAAAATGCTTTATGTATTTATATTTATTTACATCGGTCATGGCTTGTTACCTAAATCTAATGTTGA

                              8820      8830      8840      8850      8860      8870      8880      8890
                              CATATTCAATCAGGTAGTATACACTCCCCTCTTATCATTCATTATACACTCCCCTCTTATCATTCATTATTCAGGGTT
PH207_w2_flanking(1>32227)    catattcaatcaggtagtatacactcccctcttatcattcattatacactcccctcttatcattcattattcagggtt
PH207_w2(1>16227)             catattcaatcaggtagtatacactcccctcttatcattcattatacactcccctcttatcattcattattcagggtt
1754_2_f.592(1>954)           CATATTCAATCAGGTAGTATACACTCCCCTCTTATCATTCATTATACACTCCCCTCTTATCATTCATTATTCAGGGTT
WT_w2_2_f.592(1>610)          CATATTCAATCAGGTAGTATACACTCCCCTCTTATCATTCATTATACACTCCCCTCTTATCATTCATTATTCAGGGTT
```

```
                              8900      8910      8920      8930      8940      8950      8960      8970
                          CAACATATGACTACATACCCACTCATTTTTATTCCAGTGTCTGCTTCTTTCTGCAACTTCTGTTCATTATGCATCATT
PH207_w2_flanking(1>32227)  caacatatgactacatacccactcatttttattccagtgtctgcttctttctgcaacttctgttcattatgcatcatt
PH207_w2(1>16227)           caacatatgactacatacccactcatttttattccagtgtctgcttctttctgcaacttctgttcattatgcatcatt
1754_2_f.592(1>954)         CAACATATGACTACATACCCACTCATTTTTATTCCAGTGTCTGCTTCTTTCTGCAACTTCTGTTCATTATGCATCATT
WT_w2_2_f.592(1>610)        CAACATATGACTACATACCCACTCATTTTTATTCCAGTGTCTGCTTCTTTCTGCAACTTCTGTTCATTATGCATCATT

                                8980      8990      9000      9010      9020      9030      9040
                          TATGCCTACATGTTTCCTTTTTGTTCACTATGAAATCCATGAAAAATGAGGGTATCACTGTTGTTATGTATTTTGAAC
PH207_w2_flanking(1>32227)  tatgcctacatgtttcctttttgttcactatgaaatccatgaaaaatgagggtatcactgttgttatgtattttgaac
PH207_w2(1>16227)           tatgcctacatgtttcctttttgttcactatgaaatccatgaaaaatgagggtatcactgttgttatgtattttgaac
1754_2_f.592(1>954)         TATGCCTACATGTTTCCTTTTTGTTCACTATGAAATCCATGAAAAATGAGGGTATCACTGTTGTTATGTATTTTGAAC
WT_w2_2_f.592(1>610)        TATGCCTACATGTTTCCTTTTTGTTCACTATGAAATCCATGA

                          9050      9060      9070      9080      9090      9100      9110      9120
                          AGCGAGTAGAATTGTAGATACACATCATCATCTTGTTGGCTCAGTGGTATGTAATGGG-TAGGGATGCAATCGGGTAC
PH207_w2_flanking(1>32227)  agcgagtagaattgtagatacacatcatcatcttgttggctcagtggtatgtaatggg-tagggatgcaatcgggtac
PH207_w2(1>16227)           agcgagtagaattgtagatacacatcatcatcttgttggctcagtggtatgtaatggg-tagggatgcaatcgggtac
1754_2_f.592(1>954)         AGCGAGTAGAATTGTAGATACACATCATCATCTTGTTGGCTCAGTGGTATGTAATGGGGTAGGGATGCAATCGGGTAC

                            9130      9140      9150      9160      9170      9180      9190      9200
                          ACAATGGTGTTTTTTGGGTCAACTAATTAGTTACGTAGCATGCTGTTTTGATTCATTTCTTTCTCTAGATTAATGAAG
PH207_w2_flanking(1>32227)  acaatggtgttttttgggtcaactaattagttacgtagcatgctgttttgattcatttctttctctagattaatgaag
PH207_w2(1>16227)           acaatggtgttttttgggtcaactaattagttacgtagcatgctgttttgattcatttctttctctagattaatgaag
1754_2_f.592(1>954)         ACAATGGTGTTTTTTGGGTCAACTAATTAGTTACGTAGCATGCTGTTTTGATTCATTTCTTTCTCTAGATTAATGAAG

                              9210      9220      9230      9240      9250      9260      9270      9280
                          TGACATGTCACTTTAGTAAGTTTTGGGTCGGTACAATGACCAAAAGTGGGTCTTGCATTCCTATTACTGGGCAACTGA
PH207_w2_flanking(1>32227)  tgacatgtcactttagtaagttttgggtcggtacaatgaccaaaagtgggtcttgcattcctattactgggcaactga
PH207_w2(1>16227)           tgacatgtcactttagtaagttttgggtcggtacaatgaccaaaagtgggtcttgcattcctattactgggcaactga
1754_2_f.592(1>954)         TGACATGTCACTTTAGTAAGTTTTGGGTCGGTACAATGACCAAAAGTGGGTCTTGCATTCCTATTACTGGGCAACTGA

                              9290      9300      9310      9320      9330      9340      9350      9360
                          GTTATATGTTGGG-AAACTTTCTTCCCTTTTGAATGCATATTGACTCTTTGGTATTATAATTCAGGAGAATTCAATGT
PH207_w2_flanking(1>32227)  gttatatgttggg-aaactttcttcccttttgaatgcatattgactctttggtattataattcaggagaattcaatgt
PH207_w2(1>16227)           gttatatgttggg-aaactttcttcccttttgaatgcatattgactctttggtattataattcaggagaattcaatgt
1754_2_f.592(1>954)         GTTATATGTTGGGGAAACTTTCTTCCCTTTTGAATGCATATTGACTCTTTGGTATTA

                                9370      9380      9390      9400      9410      9420      9430
                          TATGCAGGTAGCTAATATTAATGTTAAACAAGAGACACCAGTTGGCCATGGAAAGGTCACATGCTTTAGCATTTACTC
PH207_w2_flanking(1>32227)  tatgcaggtagctaatattaatgttaaacaagagacaccagttggccatggaaaggtcacatgctttagcatttactc
PH207_w2(1>16227)           tatgcaggtagctaatattaatgttaaacaagagacaccagttggccatggaaaggtcacatgctttagcatttactc

                          9440      9450      9460      9470      9480      9490      9500      9510
                          TGGCACTAAAGGTGCCGAGGCTGATTTTGGAAATGGCAAGACATGCATTTGGGTGGATGTGCTAGATGGTGGACCAGA
PH207_w2_flanking(1>32227)  tggcactaaaggtgccgaggctgattttggaaatggcaagacatgcatttgggtggatgtgctagatggtggaccaga
PH207_w2(1>16227)           tggcactaaaggtgccgaggctgattttggaaatggcaagacatgcatttgggtggatgtgctagatggtggaccaga

                            9520      9530      9540      9550      9560      9570      9580      9590
                          TGTACTCATGGAGTTTGTCCCATTTTTTGAGGATTCATCAATCAGGAAGGTGAAATGCAGCTTACTTCTTTGTTCGTT
PH207_w2_flanking(1>32227)  tgtactcatggagtttgtcccattttttgaggattcatcaatcaggaaggtgaaatgcagcttacttctttgttcgtt
PH207_w2(1>16227)           tgtactcatggagtttgtcccattttttgaggattcatcaatcaggaaggtgaaatgcagcttacttctttgttcgtt

                              9600      9610      9620      9630      9640      9650      9660      9670
                          TGTTTGGAGATCTGCTCTGTTCCGTCAGTTGTATATGC-TTATTGCAGTATACATGGAAATCATTGCAATTATTTAAG
PH207_w2_flanking(1>32227)  tgtttggagatctgctctgttccgtcagttgtatatgc-ttattgcagtatacatggaaatcattgcaattatttaag
PH207_w2(1>16227)           tgtttggagatctgctctgttccgtcagttgtatatgc-ttattgcagtatacatggaaatcattgcaattatttaag
1754_2_r.602(1>736)                   TCTGCTCTGTTCCGTCAGTTGTATATGCTTTATTGCAGTATACATGGAAATCATTGCAATTATTTAAG
WT_w2_2_r.602(1>687)                                                       CAGTATACATGGAAATCATTGCAATTATTTAAG
```

9680 9690 9700 9710 9720 9730 9740 9750

GAAGTAAACAATATATCACAGTCAAATCTAAACTCATGC-TTACAACTTTACTATGTGTTGCATGCTGAATTACCTTC

PH207_w2_flanking(1>32227) → gaagtaaacaatatatcacagtcaaatctaaactcatgc-ttacaactttactatgtgttgcatgctgaattaccttc
PH207_w2(1>16227) → gaagtaaacaatatatcacagtcaaatctaaactcatgc-ttacaactttactatgtgttgcatgctgaattaccttc
1754_2_r.602(1>736) ← GAAGTAAACAATATATCACAGTCAAATCTAAACTCATGCTTTACAACTTTACTATGTGTTGCATGCTGAATTACCTTC
WT_w2_2_r.602(1>687) ← GAAGTAAACAATATATCACAGTCAAATCTAAACTCATGC-TTACAACTTTACTATGTGTTGCATGCTGAATTACCTTC

9760 9770 9780 9790 9800 9810 9820

TTTCACTGACTTTATCCGTCTTTTGCCATCTCTAGTCAAACTTTGCTATTTATAGAATATATAGGAATATGATTAAAC

PH207_w2_flanking(1>32227) → tttcactgactttatccgtcttttgccatctctagtcaaactttgctatttatagaatatataggaatatgattaaac
PH207_w2(1>16227) → tttcactgactttatccgtcttttgccatctctagtcaaactttgctatttatagaatatataggaatatgattaaac
1754_2_r.602(1>736) ← TTTCACTGACTTTATCCGTCTTTTGCCATCTCTAGTCAAACTTTGCTATTTATAGAATATATAGGAATATGATTAAAC
WT_w2_2_r.602(1>687) ← TTTCACTGACTTTATCCGTCTTTTGCCATCTCTAGTCAAACTTTGCTATTTATAGAATATATAGGAATATGATTAAAC

9830 9840 9850 9860 9870 9880 9890 9900

ATCTACCTCCATTATCTCATCGTTTGAATCTTAAGCAAGTATCTTATTTTTCTTATGTAGGTTTGGCACAACTATAGT

PH207_w2_flanking(1>32227) → atctacctccattatctcatcgtttgaatcttaagcaagtatcttatttttcttatgtaggtttggcacaactatagt
PH207_w2(1>16227) → atctacctccattatctcatcgtttgaatcttaagcaagtatcttatttttcttatgtaggtttggcacaactatagt
1754_2_r.602(1>736) ← ATCTACCTCCATTATCTCATCGTTTGAATCTTAAGCAAGTATCTTATTTTTCTTATGTAGGTTTGGCACAACTATAGT
WT_w2_2_r.602(1>687) ← ATCTACCTCCATTATCTCATCGTTTGAATCTTAAGCAAGTATCTTATTTTTCTTATGTAGGTTTGGCACAACTATAGT

9910 9920 9930 9940 9950 9960 9970 9980

TTTGATAGTCATGTTATAGAGAACTATGGAATTAAAGTTGCTGGATTTCATGCTGATACAATGCATCTTGCACGACTT

PH207_w2_flanking(1>32227) → tttgatagtcatgttatagagaactatggaattaaagttgctggatttcatgctgatacaatgcatcttgcacgactt
PH207_w2(1>16227) → tttgatagtcatgttatagagaactatggaattaaagttgctggatttcatgctgatacaatgcatcttgcacgactt
1754_2_r.602(1>736) ← TTTGATAGTCATGTTATAGAGAACTATGGAATTAAAGTTGCTGGATTTCATGCTGATACAATGCATCTTGCACGACTT
WT_w2_2_r.602(1>687) ← TTTGATAGTCATGTTATAGAGAACTATGGAATTAAAGTTGCTGGATTTCATGCTGATACAATGCATCTTGCACGACTT

9990 10000 10010 10020 10030 10040 10050 10060

TGGGATTCTTCAAGAAGAACAGATGGAGGATATTCTCTTGAGGGACTTACAAATGATCATAGGGTCATGGGTGTTGTT

PH207_w2_flanking(1>32227) → tgggattcttcaagaagaacagatggaggatattctcttgagggacttacaaatgatcatagggtcatgggtgttgtt
PH207_w2(1>16227) → tgggattcttcaagaagaacagatggaggatattctcttgagggacttacaaatgatcatagggtcatgggtgttgtt
1754_2_r.602(1>736) ← TGGGATTCTTCAAGAAGAACAGATGGAGGATATTCTCTTGAGGGACTTACAAATGATCATAGGGTCATGGGTGTTGTT
WT_w2_2_r.602(1>687) ← TGGGATTCTTCAAGAAGAACAGATGGAGGATATTCTCTTGAGGGACTTACAAATGATCATAGGGTCATGGGTGTTGTT

10070 10080 10090 10100 10110 10120 10130 10140

CCAAAGGAATTGCAAAAGATTGGGAAGAGATCAATGAAAACCATCTTTGGTCGGAAAAAAATAAAGAAGGATGGGTCA

PH207_w2_flanking(1>32227) → ccaaaggaattgcaaaagattgggaagagatcaatgaaaaccatctttggtcggaaaaaaataaagaaggatgggtca
PH207_w2(1>16227) → ccaaaggaattgcaaaagattgggaagagatcaatgaaaaccatctttggtcggaaaaaaataaagaaggatgggtca
1754_2_r.602(1>736) ← CCAAAGGAATTGCAAAAGATTGGGAAGAGATCAATGAAAACCATCTTTGGTCGGAAAAAAATAAAGAAGGATGGGTCA
WT_w2_2_r.602(1>687) ← CCAAAGGAATTGCAAAAGATTGGGAAGAGATCAATGAAAACCATCTTTGGTCGGAAAAAAATAAAGAAGGATGGGTCA

10150 10160 10170 10180 10190 10200 10210

GAAGGGAAGATTACTGCAATTGAGCCTGTTGAAATTTTACAAAGAGAAGACAGAGAATTGTGGATCTGCTATTCTTCG

PH207_w2_flanking(1>32227) → gaagggaagattactgcaattgagcctgttgaaattttacaaagagaagacagagaattgtggatctgctattcttcg
PH207_w2(1>16227) → gaagggaagattactgcaattgagcctgttgaaattttacaaagagaagacagagaattgtggatctgctattcttcg
1754_2_r.602(1>736) ← GAAGGGAAGATTACTGCAATTGAGCCTGTTGAAATTTTACAAAGAGAAGACAGAGAATTGTGGATCTGCTATTCTTCG
WT_w2_2_r.602(1>687) ← GAAGGGAAGATTACTGCAATTGAGCCTGTTGAAATTTTACAAAGAGAAGACAGAGAATTGTGGATCTGCTATTCTTCG

10220 10230 10240 10250 10260 10270 10280 10290

TTAGATTCCATGAGTACCTTGAGGCTTTATGAGAGCTTGAAAAGCAAGCTTGAAAGGAAGCCCTGGACTTTTGACGGT

PH207_w2_flanking(1>32227) → ttagattccatgagtaccttgaggctttatgagagcttgaaaagcaagcttgaaaggaagccctggacttttgacggt
PH207_w2(1>16227) → ttagattccatgagtaccttgaggctttatgagagcttgaaaagcaagcttgaaaggaagccctggacttttgacggt
1754_2_r.602(1>736) ← TTAGATTCCATGAGTACCTTGAGGCTTTATGAGAGCTTGAAAAGCAAGCTTGAAAGGAAGCCCTGGACTTTTGACGGT
WT_w2_2_r.602(1>687) ← TTAGATTCCATGAGTACCTTGAGGCTTTATGAGAGCTTGAAAAGCAAGCTTGAAAGGAAGCCCTGGACTTTTGACGGT

10300 10310 10320 10330 10340 10350 10360 10370

TACCCCAGACCCGGAGGTTCATTGTACGATTTCTACGAGGAGTATTGGCGTCCTTTCGGTGCTATTCTTGTAAAAATG

PH207_w2_flanking(1>32227) → taccccagacccggaggttcattgtacgatttctacgaggagtattggcgtcctttcggtgctattcttgtaaaaatg
PH207_w2(1>16227) → taccccagacccggaggttcattgtacgatttctacgaggagtattggcgtcctttcggtgctattcttgtaaaaatg
1754_2_r.602(1>736) ← TACCCCAGACCCGGAGGTTCATTGTACGATTTCTACGAGGAGTA
WT_w2_2_r.602(1>687) ← TACCCCAGACCCGGAGGTTCATTGTACGATT

```
                              10380     10390     10400     10410     10420     10430     10440     10450
                             GAAACAGCTGGAATGCTTGTTGACCGTGCTTATCTTTCAGAGATTGAAAAGGTTGCTGTTGCACAGCGAGAATCAGCT
PH207_w2_flanking(1>32227) → gaaacagctggaatgcttgttgaccgtgcttatctttcagagattgaaaaggttgctgttgcacagcgagaatcagct
PH207_w2(1>16227)          → gaaacagctggaatgcttgttgaccgtgcttatctttcagagattgaaaaggttgctgttgcacagcgagaatcagct

                              10460     10470     10480     10490     10500     10510     10520     10530
                             GCAGATAAATTTCGGAAGTGGGCATCTAAGTATTGCCCTGATGCAAAATACATGAATGTTAATAGTGACACTCAGATT
PH207_w2_flanking(1>32227) → gcagataaatttcggaagtgggcatctaagtattgccctgatgcaaaatacatgaatgttaatagtgacactcagatt
PH207_w2(1>16227)          → gcagataaatttcggaagtgggcatctaagtattgccctgatgcaaaatacatgaatgttaatagtgacactcagatt

                              10540     10550     10560     10570     10580     10590     10600
                             CGACAACTTTTCTTCGGTGGTATAGAGAACATGTATAACTCTCACCTATTCTCTCTTCAAAGTTCCTTAGGGTATCCA
PH207_w2_flanking(1>32227) → cgacaacttttcttcggtggtatagagaacatgtataactctcacctattctctcttcaaagttccttagggtatcca
PH207_w2(1>16227)          → cgacaacttttcttcggtggtatagagaacatgtataactctcacctattctctcttcaaagttccttagggtatcca

                              10610     10620     10630     10640     10650     10660     10670     10680
                             GATTTTGTGTATTCTTCATCATCCATCTGTCACTTTTGATGTTTTTAAATACAAAACAAAAGGAAAAAACTTTAGTCA
PH207_w2_flanking(1>32227) → gattttgtgtattcttcatcatccatctgtcacttttgatgtttttaaatacaaaacaaaaggaaaaaactttagtca
PH207_w2(1>16227)          → gattttgtgtattcttcatcatccatctgtcacttttgatgtttttaaatacaaaacaaaaggaaaaaactttagtca

                              10690     10700     10710     10720     10730     10740     10750     10760
                             ATGTATTTTTAAGTTTGTGTCCTAGAAATTTCTTGCACGCAAATTGATATAAATTTTGATGTTTTTAATCTTTGTACA
PH207_w2_flanking(1>32227) → atgtatttttaagtttgtgtcctagaaatttcttgcacgcaaattgatataaattttgatgtttttaatctttgtaca
PH207_w2(1>16227)          → atgtatttttaagtttgtgtcctagaaatttcttgcacgcaaattgatataaattttgatgtttttaatctttgtaca

                              10770     10780     10790     10800     10810     10820     10830     10840
                             GATACAAATCTGGTGATTTTTTGCCGAAGAGTAAAGCTATTAAAGTGCCTAACAATGAAACTGCTGTTTCAGAAGGCA
PH207_w2_flanking(1>32227) → gatacaaatctggtgattttttgccgaagagtaaagctattaaagtgcctaacaatgaaactgctgtttcagaaggca
PH207_w2(1>16227)          → gatacaaatctggtgattttttgccgaagagtaaagctattaaagtgcctaacaatgaaactgctgtttcagaaggca

                              10850     10860     10870     10880     10890     10900     10910     10920
                             AGAAGGTTCCAAAGTATCGCACAATTGAACTTTTCAGCATTGCGGAAGACCTGAAAACTGATATATTTACTGCAAGTG
PH207_w2_flanking(1>32227) → agaaggttccaaagtatcgcacaattgaacttttcagcattgcggaagacctgaaaactgatatatttactgcaagtg
PH207_w2(1>16227)          → agaaggttccaaagtatcgcacaattgaacttttcagcattgcggaagacctgaaaactgatatatttactgcaagtg

                              10930     10940     10950     10960     10970     10980     10990
                             GTTGGCCTTCAGTTAGTGGGGCTGCATTGAGAAATTTGGCTGGGAAGGTTCCATCAGATCTTGTTTACTCAACAGATG
PH207_w2_flanking(1>32227) → gttggccttcagttagtggggctgcattgagaaatttggctgggaaggttccatcagatcttgtttactcaacagatg
PH207_w2(1>16227)          → gttggccttcagttagtggggctgcattgagaaatttggctgggaaggttccatcagatcttgtttactcaacagatg

                             11000     11010     11020     11030     11040     11050     11060     11070
                             ATGTAAATGATGATGAATGTGGTATTCATTCTGAAATTTCCCATTGTGATCTAGAAGACACCTCTTCTTATGGTACTG
PH207_w2_flanking(1>32227) → atgtaaatgatgatgaatgtggtattcattctgaaatttcccattgtgatctagaagacacctcttcttatggtactg
PH207_w2(1>16227)          → atgtaaatgatgatgaatgtggtattcattctgaaatttcccattgtgatctagaagacacctcttcttatggtactg

                              11080     11090     11100     11110     11120     11130     11140     11150
                             CATATGATGCATTTGGGGGAGGAAAGGAAGGAAAAGAAGCATGCCATGCCATTGCAGCTTTGTGTGAGATCTGTTCTA
PH207_w2_flanking(1>32227) → catatgatgcatttgggggaggaaaggaaggaaaagaagcatgccatgccattgcagctttgtgtgagatctgttcta
PH207_w2(1>16227)          → catatgatgcatttgggggaggaaaggaaggaaaagaagcatgccatgccattgcagctttgtgtgagatctgttcta

                              11160     11170     11180     11190     11200     11210     11220     11230
                             TTAACTCGTTAATATCCAACTTCATTCTTCCTCTACAGGTGAGCTGGCTTCACCTACTCTAATGTAGTTTGCATTGCA
PH207_w2_flanking(1>32227) → ttaactcgttaatatccaacttcattcttcctctacaggtgagctggcttcacctactctaatgtagtttgcattgca
PH207_w2(1>16227)          → ttaactcgttaatatccaacttcattcttcctctacaggtgagctggcttcacctactctaatgtagtttgcattgca
```

```
                          11240     11250     11260     11270     11280     11290     11300     11310
                        ACAGGCATTATTGTACATATGGCTATGCGAGCAATCACTTGATAGTCTTAGTTATCTATCTGTATCTTTGTCCTATTT
PH207_w2_flanking(1>32227) →  acaggcattattgtacatatggctatgcgagcaatcacttgatagtcttagttatctatctgtatctttgtcctattt
PH207_w2(1>16227)          →  acaggcattattgtacatatggctatgcgagcaatcacttgatagtcttagttatctatctgtatctttgtcctattt

                            11320     11330     11340     11350     11360     11370     11380
                        ATATTTATTATATCCTTTTGTTCATCTGGCAGGGAAATCATATATCATGTAAGGAAGGGCGGATTCACTGTTCCTTAA
PH207_w2_flanking(1>32227) →  atatttattatatccttttgttcatctggcagggaaatcatatatcatgtaaggaagggcggattcactgttccttaa
PH207_w2(1>16227)          →  atatttattatatccttttgttcatctggcagggaaatcatatatcatgtaaggaagggcggattcactgttccttaa

                      11390     11400     11410     11420     11430     11440     11450     11460
                        ATATCAACACAGAGACAGGACGTTTATCGGCAAGGGCACCAAATTTACAGGTTGGCATTTATGATTCTACATTTTCGG
PH207_w2_flanking(1>32227) →  atatcaacacagagacaggacgtttatcggcaagggcaccaaatttacaggttggcatttatgattctacattttcgg
PH207_w2(1>16227)          →  atatcaacacagagacaggacgtttatcggcaagggcaccaaatttacaggttggcatttatgattctacattttcgg

                         11470     11480     11490     11500     11510     11520     11530     11540
                        GAGTTTGCATGTGTTTTGCAGTATGAAATGACATTCTTGAGGTCAAGAATTATACAGGATTTGGGCTGTCTTTCTACA
PH207_w2_flanking(1>32227) →  gagtttgcatgtgttttgcagtatgaaatgacattcttgaggtcaagaattatacaggatttgggctgtctttctaca
PH207_w2(1>16227)          →  gagtttgcatgtgttttgcagtatgaaatgacattcttgaggtcaagaattatacaggatttgggctgtctttctaca

                           11550     11560     11570     11580     11590     11600     11610     11620
                        TATTAGTGCATTTCAGAAAAGGACCAACTACTTTGAATCTTGGGCCCTAGCTTGAAACACTGTCATACTTACTTTTTT
PH207_w2_flanking(1>32227) →  tattagtgcatttcagaaaaggaccaactactttgaatcttgggccctagcttgaaacactgtcatacttactttttt
PH207_w2(1>16227)          →  tattagtgcatttcagaaaaggaccaactactttgaatcttgggccctagcttgaaacactgtcatacttactttttt

                          11630     11640     11650     11660     11670     11680     11690     11700
                        TCTCCAACCACGCAGGAGAGCTGCACGTCATTTCATTAAGAAAAAAAAGGGGGGGGGGCTGATGGATTAGCCACCCCC
PH207_w2_flanking(1>32227) →  tctccaaccacgcaggagagctgcacgtcatttcattaagaaaaaaaaggggggggggctgatggattagccaccccc
PH207_w2(1>16227)          →  tctccaaccacgcaggagagctgcacgtcatttcattaagaaaaaaaaggggggggggctgatggattagccaccccc

                            11710     11720     11730     11740     11750     11760     11770
                        AGGGGGGAAGGGGTGGATTACAACACACACTTAAAACTATTGGACCCTAAGTGGTCCAGCCTTTAGGGCCAGGTCCTG
PH207_w2_flanking(1>32227) →  aggggggaaggggtggattacaacacacacttaaaactattggaccctaagtggtccagcctttagggccaggtcctg
PH207_w2(1>16227)          →  aggggggaaggggtggattacaacacacacttaaaactattggaccctaagtggtccagcctttagggccaggtcctg

                      11780     11790     11800     11810     11820     11830     11840     11850
                        GAGGGTCGAAGCCCCAGCCAGGCATCACATTTGACCTTCAGTAACCACTGCAGACACCACGCTTTGGATATCTGGCCT
PH207_w2_flanking(1>32227) →  gagggtcgaagccccagccaggcatcacatttgaccttcagtaaccactgcagacaccacgctttggatatctggcct
PH207_w2(1>16227)          →  gagggtcgaagccccagccaggcatcacatttgaccttcagtaaccactgcagacaccacgctttggatatctggcct

                         11860     11870     11880     11890     11900     11910     11920     11930
                        AATACCCTGAAAAACAATGTCATTTCTGTGGCTTGGGAGCTTTGGAAACACTGTCATACTTACAAAGCAATAGATGAT
PH207_w2_flanking(1>32227) →  aataccctgaaaaacaatgtcatttctgtggcttgggagctttggaaacactgtcatacttacaaagcaatagatgat
PH207_w2(1>16227)          →  aataccctgaaaaacaatgtcatttctgtggcttgggagctttggaaacactgtcatacttacaaagcaatagatgat

                           11940     11950     11960     11970     11980     11990     12000     12010
                        AGGTTCAAGCTATTGTACTCAAATAGACAAGAATCTCTTAGTCAATGTATGCAACAGTTAGATACTTAGATTGGCATG
PH207_w2_flanking(1>32227) →  aggttcaagctattgtactcaaatagacaagaatctcttagtcaatgtatgcaacagttagatacttagattggcatg
PH207_w2(1>16227)          →  aggttcaagctattgtactcaaatagacaagaatctcttagtcaatgtatgcaacagttagatacttagattggcatg

                          12020     12030     12040     12050     12060     12070     12080     12090
                        TTCTGTATACTTAATATGTGTGGACTGGATGAACTTAAATGAAGAACTGGTATTGGTATTAAGTACTCCATCCGTTCT
PH207_w2_flanking(1>32227) →  ttctgtatacttaatatgtgtggactggatgaacttaaatgaagaactggtattggtattaagtactccatccgttct
PH207_w2(1>16227)          →  ttctgtatacttaatatgtgtggactggatgaacttaaatgaagaactggtattggtattaagtactccatccgttct
WT_w2_4_r.624(1>487)       ←       TATACTTAATATGTGTGGACTGGATGAACTTAAATGAAGAACTGGTATTGGTATTAAGTACTCCATCCGTTCT
1754_4_r.624(1>485)        ←         TACTTAATATGTGTGGACTGGATGAACTTAAATGAAGAACTGGTATTGGTATTAAGTACTCCATCCGTTCT
1754_4_f.594(1>288)        →                                                   TGGTATTGGTATTAAGTACTCCATCCGTTCT
WT_w2_4_f.594(1>263)       →                                                   TGGTATTGGTATTAAGTACTCCATCCGTTCT
```

```
                                  12100     12110     12120     12130     12140     12150     12160
                                  AAAATAGTATTCGTTTTAACTCCTGATTTTTATTTCTATATCCAACTAGATGATGATGAATGTAGACACATATATAAA
PH207_w2_flanking(1>32227)  →     aaaatagtattcgttttaactcctgatttttatttctatatccaactagatgatgatgaatgtagacacatatataaa
PH207_w2(1>16227)           →     aaaatagtattcgttttaactcctgatttttatttctatatccaactagatgatgatgaatgtagacacatatataaa
WT_w2_4_r.624(1>487)        ←     AAAATAGTATTCGTTTTAACTCCTGATTTTTATTTCTATATCCAACTAGATGATGATGAATGTAGACACATATATAAA
1754_4_r.624(1>485)         ←     AAAATAGTATTCGTTTTAACTCCTGATTTTTATTTCTATATCCAACTAGATGATGATGAATGTAGACACATATATAAA
1754_4_f.594(1>288)         →     AAAATAGTATTCGTTTTAACTCCTGATTTTTATTTCTATATCCAACTAGATGATGATGAATGTAGACACATATATAAA
WT_w2_4_f.594(1>263)        →     AAAATAGTATTCGTTTTAACTCCTGATTTTTATTTCTATATCCAACTAGATGATGATGAATGTAGACACATATATAAA

                             12170     12180     12190     12200     12210     12220     12230     12240
                                  AAACATACACCAAGTATTGTATGAATCCATAAAAAAGCAAAACAAATTTTATTTTGGTACAGGGAGTATTAAAATAAG
PH207_w2_flanking(1>32227)  →     aaacatacaccaagtattgtatgaatccataaaaaagcaaaacaaattttattttggtacagggagtattaaaataag
PH207_w2(1>16227)           →     aaacatacaccaagtattgtatgaatccataaaaaagcaaaacaaattttattttggtacagggagtattaaaataag
WT_w2_4_r.624(1>487)        ←     AAACATACACCAAGTATTGTATGAATCCATAAAAAAGCAAAACAAATTTTATTTTGGTACAGGGAGTATTAAAATAAG
1754_4_r.624(1>485)         ←     AAACATACACCAAGTATTGTATGAATCCATAAAAAAGCAAAACAAATTTTATTTTGGTACAGGGAGTATTAAAATAAG
1754_4_f.594(1>288)         →     AAACATACACCAAGTATTGTATGAATCCATAAAAAAGCAAAACAAATTTTATTTTGGTACAGGGAGTATTAAAATAAG
WT_w2_4_f.594(1>263)        →     AAACATACACCAAGTATTGTATGAATCCATAAAAAAGCAAAACAAATTTTATTTTGGTACAGGGAGTATTAAAATAAG

                               12250     12260     12270     12280     12290     12300     12310     12320
                                  CTCTGTTATTTCTGCCAAAATAGGTAGCCCAGGTTGTTGCAGTATTTAATATGATAATGTTATAGATGCATTCTTAGG
PH207_w2_flanking(1>32227)  →     ctctgttatttctgccaaaataggtagcccaggttgttgcagtatttaatatgataatgttatagatgcattcttagg
PH207_w2(1>16227)           →     ctctgttatttctgccaaaataggtagcccaggttgttgcagtatttaatatgataatgttatagatgcattcttagg
WT_w2_4_r.624(1>487)        ←     CTCTGTTATTTCTGCCAAAATAGGTAGCCCAGGTTGTTGCAGTATTTAATATGATAATGTTATAGATGCATTCTTAGG
1754_4_r.624(1>485)         ←     CTCTGTTATTTCTGCCAAAATAGGTAGCCCAGGTTGTTGCAGTATTTAATATGATAATGTTATAGATGCATTCTTAGG
1754_4_f.594(1>288)         →     CTCTGTTATTTCTGCCAAAATAGGTAGCCCAGGTTGTTGCAGTATTTAATATGATAATGTTATAGATGCATTCTTAGG
WT_w2_4_f.594(1>263)        →     CTCTGTTATTTCTGCCAAAATAGGTAGCCCAGGTTGTTGCAGTATTTAATATGATAATGTTATAGATGCATTCTTA

                                 12330     12340     12350     12360     12370     12380     12390     12400
                                  CAAGCAGTATCGTAGGAGGAAAGGGGGCCAGCCGCCAGCGCAGCTAGGAGTTGGGCCCAGATGCGCAGCTAGAAGATA
PH207_w2_flanking(1>32227)  →     caagcagtatcgtaggaggaaagggggccagccgccagcgcagctaggagttgggcccagatgcgcagctagaagata
PH207_w2(1>16227)           →     caagcagtatcgtaggaggaaagggggccagccgccagcgcagctaggagttgggcccagatgcgcagctagaagata
WT_w2_4_r.624(1>487)        ←     CAAGCAGTATCGTAGGAGGAAAGGGGGCCAGCCGCCAGCGCAGCTAGGAGTTGGGCCCAGATGCGCAGCTAGAAGATA
1754_4_r.624(1>485)         ←     CAAGCAGTATCGTAGGAGGAAAGGGGGCCAGCCGCCAGCGCAGCTAGGAGTTGGGCCCAGATGCGCAGCTAGAAGATA
1754_4_f.594(1>288)         →     CAAGCAGTATCGTATGAGGAAAG

                                    12410     12420     12430     12440     12450     12460     12470     12480
                                  GGGGTCAGGAGATAGTTGTTCAGTTAAGAGATAGATTAGATCTCCTATTTTATAGTAGATGGTTGTTAGGCAGTTGGG
PH207_w2_flanking(1>32227)  →     ggggtcaggagatagttgttcagttaagagatagattagatctcctattttatagtagatggttgttaggcagttggg
PH207_w2(1>16227)           →     ggggtcaggagatagttgttcagttaagagatagattagatctcctattttatagtagatggttgttaggcagttggg
WT_w2_4_r.624(1>487)        ←     GGGGTCAGGAGATAGTTGTTCAGTTAAGAGATAGATTAGATCTCCTATTTTATAGTAGATGGTTGTTAGGCAGTTGGG
1754_4_r.624(1>485)         ←     GGGGTCAGGAGATAGTTGTTCAGTTAAGAGATAGATTAGATCTCCTATTTTATAGTAGATGGTTGTTAGGCAGTTGGG

                                  12490     12500     12510     12520     12530     12540     12550
                                  TTGTGCGGCAGTCCCAGTCTATAAGGGAATTTGCCTTTAGTTTGTATTAGGTATTGAAGGAATAANTCCCAGTCTATA
PH207_w2_flanking(1>32227)  →     ttgtgcggcagtcccagtctataagggaatttgcctttagtttgtattaggtattgaaggaataantcccagtctata
PH207_w2(1>16227)           →     ttgtgcggcagtcccagtctataagggaatttgcctttagtttgtattaggtattgaaggaataantcccagtctata
WT_w2_4_r.624(1>487)        ←     TTGTGCGGCAGTCCCAGTCTATAA
1754_4_r.624(1>485)         ←     TTGTGCGGCAGTCCCAGTCTATAA

                             12560     12570     12580     12590     12600     12610     12620     12630
                                  AGGGAATTTGCCTTTAGTTTGTATTAGGTATTGAAGGAATAAAGATCTCCATGCAGGGAGATTACCTTCGGTCGTCCT
PH207_w2_flanking(1>32227)  →     agggaatttgcctttagtttgtattaggtattgaaggaataaagatctccatgcagggagattaccttcggtcgtcct
PH207_w2(1>16227)           →     agggaatttgcctttagtttgtattaggtattgaaggaataaagatctccatgcagggagattaccttcggtcgtcct

                               12640     12650     12660     12670     12680     12690     12700     12710
                                  GGCTGACCATCACAGCCAGGCCGGCGACGGTGATCGCCCTCCCCTCTACGCCTTGCGATCTATCTCCTGTTCCTCTCA
PH207_w2_flanking(1>32227)  →     ggctgaccatcacagccaggccggcgacggtgatcgccctcccctctacgccttgcgatctatctcctgttcctctca
PH207_w2(1>16227)           →     ggctgaccatcacagccaggccggcgacggtgatcgccctcccctctacgccttgcgatctatctcctgttcctctca

                                 12720     12730     12740     12750     12760     12770     12780     12790
                                  TCCTCTTGTCTCTCCCCTGCGTACTCCCAAGAGAAGAAGCAGCAGACTACATCATTTGGTATCAGATTCAACTGGAGC
PH207_w2_flanking(1>32227)  →     tcctcttgtctctcccctgcgtactcccaagagaagaagcagcagactacatcatttggtatcagattcaactggagc
PH207_w2(1>16227)           →     tcctcttgtctctcccctgcgtactcccaagagaagaagcagcagactacatcatttggtatcagattcaactggagc
```

12800 12810 12820 12830 12840 12850 12860 12870

ATCGCCTTCCATGGTCGGCTCCGGGGGTGATCTTGCGACCATGACAGCGGCGGAGAAACTTGACTTTCTGGTCACCCA

PH207_w2_flanking(1>32227) → atcgccttccatggtcggctccgggggtgatcttgcgaccatgacagcggcggagaaacttgactttctggtcaccca
PH207_w2(1>16227) → atcgccttccatggtcggctccgggggtgatcttgcgaccatgacagcggcggagaaacttgactttctggtcaccca

12880 12890 12900 12910 12920 12930 12940

GTTCACATCAATGACCGATCTGGGGACCAGATTGTCGGCGCAATTGGAAACTCTCAATCGGCACATGGACTCCCATGA

PH207_w2_flanking(1>32227) → gttcacatcaatgaccgatctggggaccagattgtcggcgcaattggaaactctcaatcggcacatggactcccatga
PH207_w2(1>16227) → gttcacatcaatgaccgatctggggaccagattgtcggcgcaattggaaactctcaatcggcacatggactcccatga

12950 12960 12970 12980 12990 13000 13010 13020

CACGCGCCTCGCCTGCCTGGAAGTTTCACCAGGGAAACAGCCGGCCCAGCCACAGGCGACTTCTGGTTTGGGTAGCCT

PH207_w2_flanking(1>32227) → cacgcgcctcgcctgcctggaagtttcaccagggaaacagccggcccagccacaggcgacttctggtttgggtagcct
PH207_w2(1>16227) → cacgcgcctcgcctgcctggaagtttcaccagggaaacagccggcccagccacaggcgacttctggtttgggtagcct

13030 13040 13050 13060 13070 13080 13090 13100

GGCGCAGGTGGATCTGGATGACGACAACGACGAAGCAGAAACTGAAGACCTGGACGACCAGCTGGATTGGGCGCCTCC

PH207_w2_flanking(1>32227) → ggcgcaggtggatctggatgacgacaacgacgaagcagaaactgaagacctggacgaccagctggattgggcgcctcc
PH207_w2(1>16227) → ggcgcaggtggatctggatgacgacaacgacgaagcagaaactgaagacctggacgaccagctggattgggcgcctcc

13110 13120 13130 13140 13150 13160 13170 13180

AACAGCATGGGCAGGCGTCGCTCCAATCAGGGTTGCCGGGACGGCGGCGGCAGGCGGCGCTACCACAACCGTGGATGC

PH207_w2_flanking(1>32227) → aacagcatgggcaggcgtcgctccaatcagggttgccgggacggcggcggcaggcggcgctaccacaaccgtggatgc
PH207_w2(1>16227) → aacagcatgggcaggcgtcgctccaatcagggttgccgggacggcggcggcaggcggcgctaccacaaccgtggatgc

13190 13200 13210 13220 13230 13240 13250 13260

AACAACGACTACAGGTGTGACGACTCCGACCGTGGGCGGCCTCCCCGTGACCACCACGATCGTGGTGACCATCATCGT

PH207_w2_flanking(1>32227) → aacaacgactacaggtgtgacgactccgaccgtgggcggcctccccgtgaccaccacgatcgtggtgaccatcatcgt
PH207_w2(1>16227) → aacaacgactacaggtgtgacgactccgaccgtgggcggcctccccgtgaccaccacgatcgtggtgaccatcatcgt

13270 13280 13290 13300 13310 13320 13330

CCCAATCCCAAGTTTCCATCCTTCAACGGTGAGACCGATCCTCTTCCATGGCTCACCAAGTGTGCCTCATACTTTCGC

PH207_w2_flanking(1>32227) → cccaatcccaagtttccatccttcaacggtgagaccgatcctcttccatggctcaccaagtgtgcctcatactttcgc
PH207_w2(1>16227) → cccaatcccaagtttccatccttcaacggtgagaccgatcctcttccatggctcaccaagtgtgcctcatactttcgc

13340 13350 13360 13370 13380 13390 13400 13410

GGTATGCGCACCCTTGAGTTGGATTGGGTCTGGATAGCATCCCTGAATCTGGAAGGTGTCGCAGCGGAATGGTACTAC

PH207_w2_flanking(1>32227) → ggtatgcgcacccttgagttggattgggtctggatagcatccctgaatctggaaggtgtcgcagcggaatggtactac
PH207_w2(1>16227) → ggtatgcgcacccttgagttggattgggtctggatagcatccctgaatctggaaggtgtcgcagcggaatggtactac

13420 13430 13440 13450 13460 13470 13480 13490

ACGCTAGAGCGGGACCATGGCATCTTGTCCTAGCCTCGGTTTGCCGCCTTCGTCAACATGCGCTTTGGACTGCCCCTG

PH207_w2_flanking(1>32227) → acgctagagcgggaccatggcatcttgtcctagcctcggtttgccgccttcgtcaacatgcgctttggactgcccctg
PH207_w2(1>16227) → acgctagagcgggaccatggcatcttgtcctagcctcggtttgccgccttcgtcaacatgcgctttggactgcccctg
WT_638_1.638(1>1006) → CGCTAGAGCGGGACCATGGCATCTTGTCCTAGCCTCGGTTTGCCGCCTTCGTCAACATGCGCTTTGGACTGCCCCTG
1754_638_1.638(1>875) → CGCTAGAGCGGGACCATGGCATCTTGTCCTAGCCTCGGTTTGCCGCCTTCGTCAACATGCGCTTTGGACTGCCCCTG

13500 13510 13520 13530 13540 13550 13560 13570

CGCTCCAATGGCCTGGCCGAGGTCAAGGACCTTCGCCGCACTGGCTCTGTGGACGAGTACACAATGCAGTTCTCGCAG

PH207_w2_flanking(1>32227) → cgctccaatggcctggccgaggtcaaggaccttcgccgcactggctctgtggacgagtacacaatgcagttctcgcag
PH207_w2(1>16227) → cgctccaatggcctggccgaggtcaaggaccttcgccgcactggctctgtggacgagtacacaatgcagttctcgcag
WT_638_1.638(1>1006) → CGCTCCAATGGCCTGGCCGAGGTCAAGGACCTTCGCCGCACTGGCTCTGTGGACGAGTACACAATGCAGTTCTCGCAG
1754_638_1.638(1>875) → CGCTCCAATGGCCTGGCCGAGGTCAAGGACCTTCGCCGCACTGGCTCTGTGGACGAGTACACAATGCAGTTCTCGCAG

13580 13590 13600 13610 13620 13630 13640 13650

CTCCTTTGCCGTTGTGATGATCTCTCCTTGACCCAAACAAGTTAATCTGTACACAGCTGGCCTAGGCGAGGCGCTGCG

PH207_w2_flanking(1>32227) → ctcctttgccgttgtgatgatctctccttgacccaaacaagttaatctgtacacagctggcctaggcgaggcgctgcg
PH207_w2(1>16227) → ctcctttgccgttgtgatgatctctccttgacccaaacaagttaatctgtacacagctggcctaggcgaggcgctgcg
WT_638_1.638(1>1006) → CTCCTTTGCCGTTGTGATGATCTCTCCTTGACCCAAACAAGTTAATCTGTACACAGCTGGCCTAGGCGAGGCGCTGCG

```
                               13580     13590     13600     13610     13620     13630     13640     13650
                            CTCCTTTGCCGTTGTGATGATCTCTCCTTGACCCAAACAAGTTAATCTGTACACAGCTGGCCTAGGCGAGGCGCTGCG
1754_638_1.638(1>875)      → CTCCTTTGCCGTTGTGATGATCTCTCCTTGACCCAAACAAGTTAATCTGTACACAGCTGGCCTAGGCGAGGCGCTGCG

                                 13660     13670     13680     13690     13700     13710     13720
                            GACAGATGTGGAGCTCCAAGCCCCCACTAATCTTCAAACAGCCATGAGTCCGGCAAGGGCTTATGAGAGGCGCGAGAA
PH207_w2_flanking(1>32227) → gacagatgtggagctccaagcccccactaatcttcaaacagccatgagtccggcaagggcttatgagaggcgcgagaa
PH207_w2(1>16227)          → gacagatgtggagctccaagcccccactaatcttcaaacagccatgagtccggcaagggcttatgagaggcgcgagaa
WT_638_1.638(1>1006)       → GACAGATGTGGAGCTCCAAGCCCCCACTAATCTTCAAACAGCCATGAGTCCGGCAAGGGCTTATGAGAGGCGCGAGAA
1754_638_1.638(1>875)      → GACAGATGTGGAGCTCCAAGCCCCCACTAATCTTCAAACAGCCATGAGTCCGGCAAGGGCTTATGAGAGGCGCGAGAA

                          13730     13740     13750     13760     13770     13780     13790     13800
                            TGAAGCGGCAAAGGAGAGAACCTTCAAGGGAGGACGTGCTGGCCCTATACAGCAGAAACCAATGGCACCAGCACTGGC
PH207_w2_flanking(1>32227) → tgaagcggcaaaggagagaaccttcaagggaggacgtgctggccctatacagcagaaaccaatggcaccagcactggc
PH207_w2(1>16227)          → tgaagcggcaaaggagagaaccttcaagggaggacgtgctggccctatacagcagaaaccaatggcaccagcactggc
WT_638_1.638(1>1006)       → TGAAGCGGCAAAGGAGAGAACCTTCAAGGGAGGACGTGCTGGCCCTATACAGCAGAAACCAATGGCACCAGCACTGGC
1754_638_1.638(1>875)      → TGAAGCGGCAAAGGAGAGAACCTTCAAGGGAGGACGTGCTGGCCCTATACAGCAGAAACCAATGGCACCAGCACTGGC

                             13810     13820     13830     13840     13850     13860     13870     13880
                            CCAGAAGACTATACCAAACCCCAGATCACGTTTCAGAAGACTGTCGGCAGAGGAGTTGGCTGCAAAAAGGGCGTGCAG
PH207_w2_flanking(1>32227) → ccagaagactataccaaaccccagatcacgtttcagaagactgtcggcagaggagttggctgcaaaaagggcgtgcag
PH207_w2(1>16227)          → ccagaagactataccaaaccccagatcacgtttcagaagactgtcggcagaggagttggctgcaaaaagggcgtgcag
WT_638_1.638(1>1006)       → CCAGAAGACTATACCAAACCCCAGATCACGTTTCAGAAGACTGTCGGCAGAGGAGTTGGCTGCAAAAAGGGCGTGCAG
1754_638_1.638(1>875)      → CCAGAAGACTATACCAAACCCCAGATCACGTTTCAGAAGACTGTCGGCAGAGGAGTTGGCTGCAAAAAGGGCGTGCAG

                               13890     13900     13910     13920     13930     13940     13950     13960
                            CGAACGCTATAACTGCACAGAGTACACTGCTGACCATAAATGTTCAGCCAAATGGGTCTTCTTTCTGGAGTTAGACGA
PH207_w2_flanking(1>32227) → cgaacgctataactgcacagagtacactgctgaccataaatgttcagccaaatgggtcttctttctggagttagacga
PH207_w2(1>16227)          → cgaacgctataactgcacagagtacactgctgaccataaatgttcagccaaatgggtcttctttctggagttagacga
WT_638_1.638(1>1006)       → CGAACGCTATAACTGCACAGAGTACACTGCTGACCATAAATGTTCAGCCAAATGGGTCTTCTTTCTGGAGTTAGACGA
1754_638_1.638(1>875)      → CGAACGCTATAACTGCACAGAGTACACTGCTGACCATAAATGTTCAGCCAAATGGGTCTTCTTTCTGGAGTTAGACGA

                               13970     13980     13990     14000     14010     14020     14030     14040
                            TGAAAATGAAGAGGAGGATGTAGCTGCTGAATTAGGCATCTCTCTCCACGCATTGACAGGCATTGAGGTGGGAAATAC
PH207_w2_flanking(1>32227) → tgaaaatgaagaggaggatgtagctgctgaattaggcatctctctccacgcattgacaggcattgaggtgggaaatac
PH207_w2(1>16227)          → tgaaaatgaagaggaggatgtagctgctgaattaggcatctctctccacgcattgacaggcattgaggtgggaaatac
WT_638_1.638(1>1006)       → TGAAAATGAAGAGGAGGATGTAGCTGCTGAATTAGGCATCTCTCTCCACGCATTGACAGGCATTGAGGTGGGAAATAC
1754_638_1.638(1>875)      → TGAAAATGAAGAGGAGGATGTAGCTGCTGAATTAGGCATCTCTCTCCACGCATTGACAGGCATTGAGGTGGGAAATAC

                                 14050     14060     14070     14080     14090     14100     14110
                            CATGCAGCTACGAATCAATATCCATGGCACCTCCGTGATTGCGCTAGTGGATTTAGGGTCAACCCAACCTTCATCCAG
PH207_w2_flanking(1>32227) → catgcagctacgaatcaatatccatggcacctccgtgattgcgctagtggatttagggtcaacccaaccttcatccag
PH207_w2(1>16227)          → catgcagctacgaatcaatatccatggcacctccgtgattgcgctagtggatttagggtcaacccaaccttcatccag
WT_638_1.638(1>1006)       → CATGCAGCTACGAATCAATATCCATGGCACCTCCGTGATTGCGCTAGTGGATTTAGGGTCAACCCAACCTTCATCCAG
1754_638_1.638(1>875)      → CATGCAGCTACGAATCAATATCCATGGCACCTCCGTGATTGCGCTAGTGGATTTAGGGTCAACCCAACCTTCATCCAG

                          14120     14130     14140     14150     14160     14170     14180     14190
                            GAGAATGTGGTGGCTAAACTTGGGCTGCAGGTGGACTGCAGACCTGGACTATCAGTGAAGGTAGCTAGCTAATGGGGA
PH207_w2_flanking(1>32227) → gagaatgtggtggctaaacttgggctgcaggtggactgcagacctggactatcagtgaaggtagctagctaatgggga
PH207_w2(1>16227)          → gagaatgtggtggctaaacttgggctgcaggtggactgcagacctggactatcagtgaaggtagctagctaatgggga
WT_638_1.638(1>1006)       → GAGAATGTGGTGGCTAAACTTGGGCTGCAGGTGGACTGCAGACCTGGACTATCAGTGAAGGTAGCTAGCTAATGGGGA
1754_639_2.639(1>1028)     ←            GCTAAACTTGGGCTGCAGGTGGACTGCAGACCTGGACTATCAGTGAAGGTAGCTAGCTAATGGGGA
WT_639_2.639(1>1031)       ←            GCTAAACTTGGGCTGCAGGTGGACTGCAGACCTGGACTATCAGTGAAGGTAGCTAGCTAATGGG-A
1754_638_1.638(1>875)      → GAGAATGTGGTGGCTAAACTTGGGCTGCAGGTGGACTGCAGACCTGGACTATCAGTGAAGGTAGCTAGCTAATGGGGA

                             14200     14210     14220     14230     14240     14250     14260     14270
                            CAAAATACCAAGTGCAGGCGTTTGCCACAACATCCAGCTCTCCATTCATGCAACCTTTGTTCTTACTGG-TTTTGTCT
PH207_w2_flanking(1>32227) → caaaataccaagtgcaggcgtttgccacaacatccagctctccattcatgcaacctttgttcttactgg-ttttgtct
PH207_w2(1>16227)          → caaaataccaagtgcaggcgtttgccacaacatccagctctccattcatgcaacctttgttcttactgg-ttttgtct
WT_638_1.638(1>1006)       → CAAAATACCAAGTGCAGGCGTTTGCCACAACATCCAGCTCTCCATTCATGCAACCTTTGTTCTTACTGG-TTTTGTCT
1754_639_2.639(1>1028)     ← CAAAATACCAAGTGCAGGCGTTTGCCACAACATCCAGCTCTCCATTCATGCAACCTTTGTTCTTACTGG-TTTTGTCT
WT_639_2.639(1>1031)       ← CAAAATACCAAGTGCAGGC-TTTGCCACAACATCCAGCTTTCCATTCATGCAACTTTTGTTCTTACTGG-TTTTGTCT
1754_638_1.638(1>875)      → CAAAATACCAAGTGCAGGCGTTTGCCACAACATCCAGCTCTCCATTCATGCAACCTTTGTTCTTACTGGGTTTTGTCT
```

```
                         14280     14290     14300     14310     14320     14330     14340     14350
                         TA-CCCCTCGCTGGATTTGAGGTCATTTTGGGTGTTCAGTGGCTCCAGTCTTTGGGACCAATTTTGTGGAACTTCAAA
PH207_w2_flanking(1>32227) →   ta-cccctcgctggatttgaggtcattttgggtgttcagtggctccagtctttgggaccaattttgtggaacttcaaa
PH207_w2(1>16227)          →   ta-cccctcgctggatttgaggtcattttgggtgttcagtggctccagtctttgggaccaattttgtggaacttcaaa
WT_638_1.638(1>1006)       →   TA-CCCCTCGCTGGATTTGAGGTCATTTTGGGTGTTCAGTGGCTCCAGTCTTTGGGACCAATTTTGTGGAACTTCAAA
1754_639_2.639(1>1028)     ←   TACCCCCTCGCTGGATTTGAGGTCATTTTGGGTGTTCAGTGGCTCCAGTCTTTGGGACCAATTTTGTGGAACTTCAAA
WT_639_2.639(1>1031)       ←   TA-CCCCTCGCTGGATTTGAGGTCATTTTGGGTGTTCAGTGGCTCCAGTCTTTGGGACCAATTTTGTGGAACTTCAAA
1754_638_1.638(1>875)      →   TA-CCCCTCGCTGGATTTG
```

```
                            14360     14370     14380     14390     14400     14410     14420     14430
                         GACCTCACTATGAAGATTTGGCACCAGGGTCGGAATCTGTCATGGAAGGGAGTAGGTGCAGCTGGGACTCAATGTGCT
PH207_w2_flanking(1>32227) →   gacctcactatgaagatttggcaccagggtcggaatctgtcatggaagggagtaggtgcagctgggactcaatgtgct
PH207_w2(1>16227)          →   gacctcactatgaagatttggcaccagggtcggaatctgtcatggaagggagtaggtgcagctgggactcaatgtgct
WT_638_1.638(1>1006)       →   GACCTCACTATGAAGATTTGGCACCAGGGTCGGAATCTGTCATGGAAGGGAGTAGGTGCAGCTGGGACTCAAT
1754_639_2.639(1>1028)     ←   GACCTCACTATGAAGATTTGGCACCAGGGTCGGAATCTGTCATGGAAGGGAGTAGGTGCAGCTGGGACTCAATGTGCT
WT_639_2.639(1>1031)       ←   GACCTCACTATGAAGA-TTGGCACCAGGGTCGGAATCTGTCATGGAAGGGAGTAGGTGCAGCTGGGACTCAATGTGCT
```

```
                               14440     14450     14460     14470     14480     14490     14500
                         GTCGTTCACACACAGCCAGACTTAATGCAGACCCTCATCTCCTCATTTGGTGACATGTTTGCAGAACCACAGGGGCTA
PH207_w2_flanking(1>32227) →   gtcgttcacacacagccagacttaatgcagaccctcatctcctcatttggtgacatgtttgcagaaccacaggggcta
PH207_w2(1>16227)          →   gtcgttcacacacagccagacttaatgcagaccctcatctcctcatttggtgacatgtttgcagaaccacaggggcta
1754_639_2.639(1>1028)     ←   GTCGTTCACACACAGCCAGACTTAATGCAGACCCTCATCTCCTCATTTGGTGACATGTTTGCAGAACCACAGGGGCTA
WT_639_2.639(1>1031)       ←   GTCGTTCACACACAGCCAGACTTAATGCAGACCCTCATCTCCTCATTTGGTGACATGTTTGCAGAACCACAGGGGCTA
```

```
                       14510     14520     14530     14540     14550     14560     14570     14580
                         CCACCTTCTCGCTGGCAAGACCACCTTATCAGGTTGCTGCCCGGTCCAGCGCCTGTAGCAGTGCGGCCATACCGATAT
PH207_w2_flanking(1>32227) →   ccaccttctcgctggcaagaccaccttatcaggttgctgcccggtccagcgcctgtagcagtgcggccataccgatat
PH207_w2(1>16227)          →   ccaccttctcgctggcaagaccaccttatcaggttgctgcccggtccagcgcctgtagcagtgcggccataccgatat
1754_639_2.639(1>1028)     ←   CCACCTTCTCGCTGGCAAGACCACCTTATCAGGTTGCTGCCCGGTCCAGCGCCTGTAGCAGTGCGGCCATACCGATAT
WT_639_2.639(1>1031)       ←   CCACCTTCTCGCTGGCAAGACCACCTTATCAGGTTGCTGCCCGGTCCAGCGCCTGTAGCAGTGCGGCCATACCGATAT
```

```
                          14590     14600     14610     14620     14630     14640     14650     14660
                         CCCCAGCTCTTGAAGGACGAGATTGAACGACAATGCGATATGATGCTATAGCAGGGCATAACCCGGGAGTGCACATCA
PH207_w2_flanking(1>32227) →   ccccagctcttgaaggacgagattgaacgacaatgcgatatgatgctatagcagggcataacccgggagtgcacatca
PH207_w2(1>16227)          →   ccccagctcttgaaggacgagattgaacgacaatgcgatatgatgctatagcagggcataacccgggagtgcacatca
1754_639_2.639(1>1028)     ←   CCCCAGCTCTTGAAGGACGAGATTGAACGACAATGCGATATGATGCTATAGCAGGGCATAACCCGGGAGTGCACATCA
WT_639_2.639(1>1031)       ←   CCCCAGCTCTTGAAGGACGAGATTGAACGACAATGCGATATGATGCTATAGCAGGGCATAACCCGGGAGTGCACATCA
```

```
                         14670     14680     14690     14700     14710     14720     14730     14740
                         GCGTTCTCCTCTCCCGTTCTATTGGTCAAGAAGGCTGACGGCTCCTGGCGATTCTGTGTAGACTACAGGGAGCTGAAT
PH207_w2_flanking(1>32227) →   gcgttctcctctcccgttctattggtcaagaaggctgacggctcctggcgattctgtgtagactacagggagctgaat
PH207_w2(1>16227)          →   gcgttctcctctcccgttctattggtcaagaaggctgacggctcctggcgattctgtgtagactacagggagctgaat
1754_639_2.639(1>1028)     ←   GCGTTCTCCTCTCCCGTTCTATTGGTCAAGAAGGCTGACGGCTCCTGGCGATTCTGTGTAGACTACAGGGAGCTGAAT
WT_639_2.639(1>1031)       ←   GCGTTCTCCTCTCCCGTTCTATTGGTCAAGAAGGCTGACGGCTCCTGGCGATTCTGTGTAGACTACAGGGAGCTGAAT
```

```
                            14750     14760     14770     14780     14790     14800     14810     14820
                         GCGAAGACAGTGAAAGATAAGTACCCGATTCTGGTGGTGGACGAACTGCTGGATGAGCTGCAGGGTGCGAGGTTTTTC
PH207_w2_flanking(1>32227) →   gcgaagacagtgaaagataagtacccgattctggtggtggacgaactgctggatgagctgcagggtgcgaggtttttc
PH207_w2(1>16227)          →   gcgaagacagtgaaagataagtacccgattctggtggtggacgaactgctggatgagctgcagggtgcgaggtttttc
1754_639_2.639(1>1028)     ←   GCGAAGACAGTGAAAGATAAGTACCCGATTCTGGTGGTGGACGAACTGCTGGATGAGCTGCAGGGTGCGAGGTTTTTC
WT_639_2.639(1>1031)       ←   GCGAAGACAGTGAAAGATAAGTACCCGATTCTGGTGGTGGACGAACTGCTGGATGAGCTGCAGGGTGCGAGGTTTTTC
```

```
                               14830     14840     14850     14860     14870     14880     14890
                         ACCAAACTCGACCTTCGCAGTGGGTATCACCAGGTGCGGATGCATGAAGCTGATATTGAAAAGACTGCATTACGTACT
PH207_w2_flanking(1>32227) →   accaaactcgaccttcgcagtgggtatcaccaggtgcggatgcatgaagctgatattgaaaagactgcattacgtact
PH207_w2(1>16227)          →   accaaactcgaccttcgcagtgggtatcaccaggtgcggatgcatgaagctgatattgaaaagactgcattacgtact
1754_639_2.639(1>1028)     ←   ACCAAACTCGACCTTCGCAGTGGGTATCACCAGGTGCGGATGCATGAAGCTGATATTGAAAAGACTGCATTACGTACT
WT_639_2.639(1>1031)       ←   ACCAAACTCGACCTTCGCAGTGGGTATCACCAGGTGCGGATGCATGAAGCTGATATTGAAAAGACTGCATTACGTACT
```

```
                       14900     14910     14920     14930     14940     14950     14960     14970
                         CATCGGGGGCACTTCGAGTTCTTGGTGATGCCTTTTGGTCTTACAAATGCACCGTCAACATTTCAGTCCTTGATGAAT
PH207_w2_flanking(1>32227) →   catcgggggcacttcgagttcttggtgatgccttttggtcttacaaatgcaccgtcaacatttcagtccttgatgaat
PH207_w2(1>16227)          →   catcgggggcacttcgagttcttggtgatgccttttggtcttacaaatgcaccgtcaacatttcagtccttgatgaat
```

```
                                   14900     14910     14920     14930     14940     14950     14960     14970
                                   CATCGGGGGCACTTCGAGTTCTTGGTGATGCCTTTTGGTCTTACAAATGCACCGTCAACATTTCAGTCCTTGATGAAT
1754_639_2.639(1>1028)        ←    CATCGGGGGCACTTCGAGTTCTTGGTGATGCCTTTTGGTCTTACAAATGCACCGTCAACATTTCAGTCCTTGATGAAT
WT_639_2.639(1>1031)          ←    CATCGGGGGCACTTCGAGTTCTTGGTGATGCCTTTTGGTCTTACAAATGCACCGTCAACATTTCAGTCCTTGATGAAT

                                     14980     14990     15000     15010     15020     15030     15040     15050
                                   GAGATCTTGAAACCCTTCATCCGCAAGTTCGTCCTCGTCTTCTTCGATGATATTCTGATATTTAGCAGCTGCTGGTCC
PH207_w2_flanking(1>32227)    →    gagatcttgaaacccttcatccgcaagttcgtcctcgtcttcttcgatgatattctgatatttagcagctgctggtcc
PH207_w2(1>16227)             →    gagatcttgaaacccttcatccgcaagttcgtcctcgtcttcttcgatgatattctgatatttagcagctgctggtcc
1754_639_2.639(1>1028)        ←    GAGATCTTGAAACCCTTCATCCGCAAGTTCGTCCTCGTCTTCTTCGATGATATTCTGATATTTAGCAGCTGCTGGTCC
WT_639_2.639(1>1031)          ←    GAGATCTTGAAACCCTTCATCCGCAAGTTCGTCCTCGTCTTCTTCGATGATATTCTGATATTTAGCAGCTGCTGGTCC

                                       15060     15070     15080     15090     15100     15110     15120     15130
                                   ACTCATCTACAGCCTGTAAGGCAGGTGTTTCAGCTCATCAGATTCCATAAGTTTGCGCTCAAGCAGTCCAATTGCTCC
PH207_w2_flanking(1>32227)    →    actcatctacagcctgtaaggcaggtgtttcagctcatcagattccataagtttgcgctcaagcagtccaattgctcc
PH207_w2(1>16227)             →    actcatctacagcctgtaaggcaggtgtttcagctcatcagattccataagtttgcgctcaagcagtccaattgctcc
1754_639_2.639(1>1028)        ←    ACTCATCTACAGCCTGTAAGGCAGGTGTTTCAGCTCATCAGATTCCATAAGTTTGCGCTCAAGCAGTCCAATTGCTCC
WT_639_2.639(1>1031)          ←    ACTCATCTACAGCCTGTAAGGCAGGTGTTTCAGCTCATCAGATTCCATAAGTTTGCGCTCAAGCAGTCCAATTGCTCC

                                         15140     15150     15160     15170     15180     15190     15200     15210
                                   TTTGGGCAGGAATCAGTGGCATACTTAGGCCACATCATTTTTGCAGGAGTGGCTGGCAATGGATCCGACAAAGGTGGC
PH207_w2_flanking(1>32227)    →    tttgggcaggaatcagtggcatacttaggccacatcatttttgcaggagtggctggcaatggatccgacaaaggtggc
PH207_w2(1>16227)             →    tttgggcaggaatcagtggcatacttaggccacatcatttttgcaggagtggctggcaatggatccgacaaaggtggc
1754_639_2.639(1>1028)        ←    TTTGGGCAGGAATCAGTGGCATACTTA
WT_639_2.639(1>1031)          ←    TTTGGGCAGGAATCAGTGGCATACTTAGGCCACA

                                           15220     15230     15240     15250     15260     15270     15280
                                   TGTAGTAGACACCTGGCCACGACCACGGTCCCTACGAGCACTAAGAGGATTCCTAGGCCTCACAGGGTACTACCACAA
PH207_w2_flanking(1>32227)    →    tgtagtagacacctggccacgaccacggtccctacgagcactaagaggattcctaggcctcacagggtactaccacaa
PH207_w2(1>16227)             →    tgtagtagacacctggccacgaccacggtccctacgagcactaagaggattcctaggcctcacagggtactaccacaa

                                   15290     15300     15310     15320     15330     15340     15350     15360
                                   ATTTATTGCGGGATACGGGGCGGTAGCATGGCCCCTTTACAGCCCTCTTGAAGGAGGCATTTCATTGGTCCAAAGAAG
PH207_w2_flanking(1>32227)    →    atttattgcgggatacggggcggtagcatggcccctttacagccctcttgaaggaggcatttcattggtccaaagaag
PH207_w2(1>16227)             →    atttattgcgggatacggggcggtagcatggcccctttacagccctcttgaaggaggcatttcattggtccaaagaag

                                     15370     15380     15390     15400     15410     15420     15430     15440
                                   CGGACGAGGCCTTTGTGTTGTTGAAACAGGCATTGATGACTGCCCCATTGCTTCAGATGCCAGATTTTTCAAAACGGT
PH207_w2_flanking(1>32227)    →    cggacgaggcctttgtgttgttgaaacaggcattgatgactgccccattgcttcagatgccagatttttcaaaacggt
PH207_w2(1>16227)             →    cggacgaggcctttgtgttgttgaaacaggcattgatgactgccccattgcttcagatgccagatttttcaaaacggt

                                       15450     15460     15470     15480     15490     15500     15510     15520
                                   TCATAGTCGACTGTGATGCATCTGGTGCCGCAGCCGGATTCGGGGCTGTGCTTCACCAAGGAGATGGATCCATTGCTT
PH207_w2_flanking(1>32227)    →    tcatagtcgactgtgatgcatctggtgccgcagccggattcggggctgtgcttcaccaaggagatggatccattgctt
PH207_w2(1>16227)             →    tcatagtcgactgtgatgcatctggtgccgcagccggattcggggctgtgcttcaccaaggagatggatccattgctt

                                         15530     15540     15550     15560     15570     15580     15590     15600
                                   TAGTCGCCCCGTAGCACCACACCATCAGAAGTTACCCACATATGAACGTGAATTGATTGGTTTAGTCAAGAATGTTCG
PH207_w2_flanking(1>32227)    →    tagtcgccccgtagcaccacaccatcagaagttacccacatatgaacgtgaattgattggtttagtcaagaatgttcg
PH207_w2(1>16227)             →    tagtcgccccgtagcaccacaccatcagaagttacccacatatgaacgtgaattgattggtttagtcaagaatgttcg

                                           15610     15620     15630     15640     15650     15660     15670
                                   TCACTGGAGACCTTATTTGTGGGGGCGGCCATTCACAGTTCGGACAGACCATTATAGTCTCAAATTCATCCTTGATCA
PH207_w2_flanking(1>32227)    →    tcactggagaccttatttgtgggggcggccattcacagttcggacagaccattatagtctcaaattcatccttgatca
PH207_w2(1>16227)             →    tcactggagaccttatttgtgggggcggccattcacagttcggacagaccattatagtctcaaattcatccttgatca

                                   15680     15690     15700     15710     15720     15730     15740     15750
                                   GCGATTGTCCACCATTTCACAGCATGGGTCAGCAAACTCTTTGGATATGATTTGGAGGTGGAGTACCGTGTACCGTCC
PH207_w2_flanking(1>32227)    →    gcgattgtccaccatttcacagcatgggtcagcaaactctttggatatgatttggaggtggagtaccgtgtaccgtcc
PH207_w2(1>16227)             →    gcgattgtccaccatttcacagcatgggtcagcaaactctttggatatgatttggaggtggagtaccgtgtaccgtcc
```

```
                                15760     15770     15780     15790     15800     15810     15820     15830
                                TGGGAAATTGAATGGGGCAGCGGATGCACTGTCCAGACGCGAAGAGAACCAACCCTCAGTCCATGCAATTTCAGCACC
PH207_w2_flanking(1>32227)  →   tgggaaattgaatggggcagcggatgcactgtccagacgcgaagagaaccaaccctcagtccatgcaatttcagcacc
PH207_w2(1>16227)           →   tgggaaattgaatggggcagcggatgcactgtccagacgcgaagagaaccaaccctcagtccatgcaatttcagcacc

                                  15840     15850     15860     15870     15880     15890     15900     15910
                                TACCTTTGAGCTCTTTGATGTCCTCCGTGAGGAGTATGGGGGTCACCCTCAGGTGGCGTCTGTCCGGGAGATGATAAA
PH207_w2_flanking(1>32227)  →   tacctttgagctctttgatgtcctccgtgaggagtatgggggtcaccctcaggtggcgtctgtccgggagatgataaa
PH207_w2(1>16227)           →   tacctttgagctctttgatgtcctccgtgaggagtatgggggtcaccctcaggtggcgtctgtccgggagatgataaa

                                    15920     15930     15940     15950     15960     15970     15980     15990
                                GACTGGAACGGCAAAGGTGGGATGGACAGAGGCTGATGATCTGCTACTTTTTAACAAAAAAAGTATTTCTACCGGATT
PH207_w2_flanking(1>32227)  →   gactggaacggcaaaggtgggatggacagaggctgatgatctgctactttttaacaaaaaaagtatttctaccggatt
PH207_w2(1>16227)           →   gactggaacggcaaaggtgggatggacagaggctgatgatctgctactttttaacaaaaaaagtatttctaccggatt

                                      16000     16010     16020     16030     16040     16050     16060
                                CCTCGTCTGTCTGGCCTTTATTGTCGGCAGACGCCCATGCAGCTGGGCATGAAGGGGTTGAGAAGACTGTCCACAGAC
PH207_w2_flanking(1>32227)  →   cctcgtctgtctggcctttattgtcggcagacgcccatgcagctgggcatgaaggggttgagaagactgtccacagac
PH207_w2(1>16227)           →   cctcgtctgtctggcctttattgtcggcagacgcccatgcagctgggcatgaaggggttgagaagactgtccacagac

                               16070     16080     16090     16100     16110     16120     16130     16140
                                TCCGTGCAGAATTTTACAACCCACATTTGTTGAAAAAGGTCCGCAAATATGTTCGGGGTTGCGCAGTTTGTCAGCGCA
PH207_w2_flanking(1>32227)  →   tccgtgcagaattttacaacccacatttgttgaaaaaggtccgcaaatatgttcggggttgcgcagtttgtcagcgca
PH207_w2(1>16227)           →   tccgtgcagaattttacaacccacatttgttgaaaaaggtccgcaaatatgttcggggttgcgcagtttgtcagcgca

                                16150     16160     16170     16180     16190     16200     16210     16220
                                ATAAGTCTGAACACCTTCATCCGGCAGGGCTGCTGCAGCCTGTGACAGTTCTTTCGGAGATATGGAGTGATACCACAA
PH207_w2_flanking(1>32227)  →   ataagtctgaacaccttcatccggcagggctgctgcagcctgtgacagttctttcggagatatggagtgataccacaa
PH207_w2(1>16227)           →   ataagtctgaacaccttcatccggcagggctgctgcagcctgtgacagttctttcggagatatggagtgataccacaa

                                  16230     16240     16250     16260     16270     16280     16290     16300
                                TGGATTTCATTGAGGGATTTACTAAGGTGGGCGGCAAGTCAGTCATTTTGATAGTGGTGGACCGTTTCTCGAAATTTG
PH207_w2_flanking(1>32227)  →   tggatttcattgagggatttactaaggtgggcggcaagtcagtcattttgatagtggtggaccgtttctcgaaatttg
PH207_w2(1>16227)           →   tggatttcattgagggatttactaaggtgggcggcaagtcagtcattttgatagtggtggaccgtttctcgaaatttg

                                    16310     16320     16330     16340     16350     16360     16370     16380
                                CCCACTTCATTCCCTTGAGCCATCCATACTCTGCCTCTTCAGTGGCCAGGGCCTTCTTTGATAATATAGTAAGATTAC
PH207_w2_flanking(1>32227)  →   cccacttcattcccttgagccatccatactctgcctcttcagtggccagggccttctttgataatatagtaagattac
PH207_w2(1>16227)           →   cccacttcattcccttgagccatccatactctgcctcttcagtggccagggccttctttgataatatagtaagattac

                                      16390     16400     16410     16420     16430     16440     16450
                                ATGGGTTCCCGTGCTCTATTGTGAATGATAGAGGCACCGTTTCACAAGTTCGTCCTGGAAGGAGTTGTTTAACCTAGC
PH207_w2_flanking(1>32227)  →   atgggttcccgtgctctattgtgaatgatagaggcaccgtttcacaagttcgtcctggaaggagttgtttaacctagc
PH207_w2(1>16227)           →   atgggttcccgtgctctattgtgaatgatagaggcaccgtttcacaagttcgtcctggaaggagttgtttaacctagc

                               16460     16470     16480     16490     16500     16510     16520     16530
                                TGGGGTTAACCACCTCTGACTGCCTCTTCGTTCTGGCAGTCAGAGGTGGTTAACATGGTAATCCTAATGTATTTGCGC
PH207_w2_flanking(1>32227)  →   tggggttaaccacctctgactgcctcttcgttctggcagtcagaggtggttaacatggtaatcctaatgtatttgcgc
PH207_w2(1>16227)           →   tggggttaaccacctctgactgcctcttcgttctggcagtcagaggtggttaacatggtaatcctaatgtatttgcgc

                                16540     16550     16560     16570     16580     16590     16600     16610
                                GTTGCGGTGCTTAGCTGGAGATCGATATCGCACTTGGCTACAATGGTTACCGTGGGCAAAGTATTGTTACAATACTTC
PH207_w2_flanking(1>32227)  →   gttgcggtgcttagctggagatcgatatcgcacttggctacaatggttaccgtgggcaaagtattgttacaatacttc
PH207_w2(1>16227)           →   gttgcggtgcttagctggagatcgatatcgcacttggctacaatggttaccgtgggcaaagtattgttacaatacttc
```

16620 16630 16640 16650 16660 16670 16680 16690

TTATCACATGACACTCAAATGCTCCCCATTTCGGGTGGTATATGGACGGGAACCTCCGTCCTTGCAGTCTTATGCACC

PH207_w2_flanking(1>32227) ttatcacatgacactcaaatgctccccatttcgggtggtatatggacgggaacctccgtccttgcagtcttatgcacc
PH207_w2(1>16227) ttatcacatgacactcaaatgctccccatttcgggtggtatatggacgggaacctccgtccttgcagtcttatgcacc

16700 16710 16720 16730 16740 16750 16760 16770

TGGGAGTGCACGGTTAGCAGCAGTTGACAAACAGATGAAAGACATGGATGACTTCTTGGCTGAAATCAAAGACAGATT

PH207_w2_flanking(1>32227) tgggagtgcacggttagcagcagttgacaaacagatgaaagacatggatgacttcttggctgaaatcaaagacagatt
PH207_w2(1>16227) tgggagtgcacggttagcagcagttgacaaacagatgaaagacatggatgacttcttggctgaaatcaaagacagatt

16780 16790 16800 16810 16820 16830 16840

GATACAGTCACATATTACTATGAAACATCAGCAAGATAAGGGCAGAAGGGAGGTTGAGTTTCAGGTTGGACAGTGGGT

PH207_w2_flanking(1>32227) gatacagtcacatattactatgaaacatcagcaagataagggcagaagggaggttgagtttcaggttggacagtgggt
PH207_w2(1>16227) gatacagtcacatattactatgaaacatcagcaagataagggcagaagggaggttgagtttcaggttggacagtgggt

16850 16860 16870 16880 16890 16900 16910 16920

CTGGGTCCGGTTACAGCAGCGTACAGCGGTGGGAGTGACTTCTCCATCTCATTCAAAGTTGGGACCAAGATTCTATGG

PH207_w2_flanking(1>32227) ctgggtccggttacagcagcgtacagcggtgggagtgacttctccatctcattcaaagttgggaccaagattctatgg
PH207_w2(1>16227) ctgggtccggttacagcagcgtacagcggtgggagtgacttctccatctcattcaaagttgggaccaagattctatgg

16930 16940 16950 16960 16970 16980 16990 17000

CCCCTACAAGATTTTGAAGACAGTAGGTCCAGTTTCTTATCAATTGGAACTACCAGCTCCTAAAGAAGTATGAAGGAA

PH207_w2_flanking(1>32227) cccctacaagattttgaagacagtaggtccagtttcttatcaattggaactaccagctcctaaagaagtatgaaggaa
PH207_w2(1>16227) cccctacaagattttgaagacagtaggtccagtttcttatcaattggaactaccagctcctaaagaagtatgaaggaa

17010 17020 17030 17040 17050 17060 17070 17080

CACCTTCAGAGACGGTGGTGCCTCTGCCTGAACTGCTGCATGGCAGAGTGGTCCCTACACCGGCCACAGTGACTAAAG

PH207_w2_flanking(1>32227) caccttcagagacggtggtgcctctgcctgaactgctgcatggcagagtggtccctacaccggccacagtgactaaag
PH207_w2(1>16227) caccttcagagacggtggtgcctctgcctgaactgctgcatggcagagtggtccctacaccggccacagtgactaaag

17090 17100 17110 17120 17130 17140 17150 17160

CTCGGCTAAATAGAGGCGTCTGGGAAGTGCTTGTGTAATGGATGGGTCAGCCCTCCTCGGAAGCAACCTGGGAACCAG

PH207_w2_flanking(1>32227) ctcggctaaatagaggcgtctgggaagtgcttgtgtaatggatgggtcagccctcctcggaagcaacctgggaaccag
PH207_w2(1>16227) ctcggctaaatagaggcgtctgggaagtgcttgtgtaatggatgggtcagccctcctcggaagcaacctgggaaccag

17170 17180 17190 17200 17210 17220 17230

TGGAAGAATTCAAGAGCCGCTTTCTGAAAGTACAGATCTTGGACGAGCTGTTTGTTGGGGAGGAGGGAAATGTTATAG

PH207_w2_flanking(1>32227) tggaagaattcaagagccgctttctgaaagtacagatcttggacgagctgtttgttggggaggagggaaatgttatag
PH207_w2(1>16227) tggaagaattcaagagccgctttctgaaagtacagatcttggacgagctgtttgttggggaggagggaaatgttatag

17240 17250 17260 17270 17280 17290 17300 17310

ATGCATTCTTAGGCAAGCAGTATCATAGGGGGAAAGGGGGCCAGCATAGCTAGGAGTTGGGCCCAGATGCACAGCTGG

PH207_w2_flanking(1>32227) atgcattcttaggcaagcagtatcatagggggaaagggggccagcatagctaggagttgggcccagatgcacagctgg
PH207_w2(1>16227) atgcattcttaggcaagcagtatcatagggggaaagggggccagcatagctaggagttgggcccagatgcacagctgg

17320 17330 17340 17350 17360 17370 17380 17390

AAGATAGGGGTCAGGAGATATTTTTTTGAAGGGACAGGCTGTAGGGAAAACCCCTACAGCACGATTTTATTAAATAAT

PH207_w2_flanking(1>32227) aagataggggtcaggagatatttttttgaagggacaggctgtagggaaaacccctacagcacgattttattaaataat
PH207_w2(1>16227) aagataggggtcaggagatatttttttgaagggacaggctgtagggaaaacccctacagcacgattttattaaataat

17400 17410 17420 17430 17440 17450 17460 17470

GAGAAGAAGGGAACAAGAAATGGACCAAGAAAAGAAAACACAAAAAACCGATTACCGAGAGTTTAGCCACAAAGACAC

PH207_w2_flanking(1>32227) gagaagaagggaacaagaaatggaccaagaaaagaaaacacaaaaaaccgattaccgagagtttagccacaaagacac
PH207_w2(1>16227) gagaagaagggaacaagaaatggaccaagaaaagaaaacacaaaaaaccgattaccgagagtttagccacaaagacac

```
        17480     17490     17500     17510     17520     17530     17540     17550
                                CCTAAGCCTCGGAACGCTATTAAACCTAAGCATTTGCAACTTCAGCATGTCATTGAAGCTGCCCTTCCAAGAAAGGAA
PH207_w2_flanking(1>32227)  →   cctaagcctcggaacgctattaaacctaagcatttgcaacttcagcatgtcattgaagctgcccttccaagaaaggaa
PH207_w2(1>16227)           →   cctaagcctcggaacgctattaaacctaagcatttgcaacttcagcatgtcattgaagctgcccttccaagaaaggaa

        17560     17570     17580     17590     17600     17610     17620
                                AGAAGGGGCCATGTTCCTGAAGATGAAGTCATTCATCTGCTTCCAAATGCACCATACTACCACTGCCAGAATATCCAT
PH207_w2_flanking(1>32227)  →   agaaggggccatgttcctgaagatgaagtcattcatctgcttccaaatgcaccatactaccactgccagaatatccat
PH207_w2(1>16227)           →   agaaggggccatgttcctgaagatgaagtcattcatctgcttccaaatgcaccatactaccactgccagaatatccat

        17630     17640     17650     17660     17670     17680     17690     17700
                                GAAGAAGTTGTGCCCAAAGGCACCCCTTGCAGCTTCGATAAAAAGGGCGTACCCAGTGCCGTAGCCTTCCTGCACTGT
PH207_w2_flanking(1>32227)  →   gaagaagttgtgcccaaaggcaccccttgcagcttcgataaaaagggcgtacccagtgccgtagccttcctgcactgt
PH207_w2(1>16227)           →   gaagaagttgtgcccaaaggcaccccttgcagcttcgataaaaagggcgtacccagtgccgtagccttcctgcactgt

        17710     17720     17730     17740     17750     17760     17770     17780
                                ACGGGGTCTGGGGAAGGGTATCTTTAAGCGCCAAGCCTTACCCGCATAATATGCAGAGGCTGGGGCTCGAACCCGGGA
PH207_w2_flanking(1>32227)  →   acggggtctggggaagggtatctttaagcgccaagccttacccgcataatatgcagaggctggggctcgaacccggga
PH207_w2(1>16227)           →   acggggtctggggaagggtatctttaagcgccaagccttacccgcataatatgcagaggctggggctcgaacccggga

        17790     17800     17810     17820     17830     17840     17850     17860
                                CATTCCGGGTACAGACGGTAGGCTCTACCGCTGCACTAGGCCCACCCTTCAAGGTTATGGCGCCAATGAATTCCAACG
PH207_w2_flanking(1>32227)  →   cattccgggtacagacggtaggctctaccgctgcactaggcccacccttcaaggttatggcgccaatgaattccaacg
PH207_w2(1>16227)           →   cattccgggtacagacggtaggctctaccgctgcactaggcccacccttcaaggttatggcgccaatgaattccaacg

        17870     17880     17890     17900     17910     17920     17930     17940
                                TAATTCCATCATTCTATACTGAAGGGACAACGAAAAAGGTGATATGTTGTTTCTTCATAGCCTTGATCACAGAGAACA
PH207_w2_flanking(1>32227)  →   taattccatcattctatactgaagggacaacgaaaaaggtgatatgttgtttcttcatagccttgatcacagagaaca
PH207_w2(1>16227)           →   taattccatcattctatactgaagggacaacgaaaaaggtgatatgttgtttcttcatagccttgatcacagagaaca

        17950     17960     17970     17980     17990     18000     18010
                                CAGTTGAGATCACTGCCCTCATTCATAAAGCCTTTTCTTCTTAAGATGCTTTCCGAGTTGATTCTGTCCCAGAAAACC
PH207_w2_flanking(1>32227)  →   cagttgagatcactgccctcattcataaagccttttcttcttaagatgctttccgagttgattctgtcccagaaaacc
PH207_w2(1>16227)           →   cagttgagatcactgccctcattcataaagccttttcttcttaagatgctttccgagttgattctgtcccagaaaacc

        18020     18030     18040     18050     18060     18070     18080     18090
                                AACCAAACGAAAACTTATGCTTTCTTACAGATCTTGGACCGCCAGATCCAGATGAAAGGAGCTGGGGGTTGGATGGAT
PH207_w2_flanking(1>32227)  →   aaccaaacgaaaacttatgctttcttacagatcttggaccgccagatccagatgaaaggagctgggggttggatggat
PH207_w2(1>16227)           →   aaccaaacgaaaacttatgctttcttacagatcttggaccgccagatccagatgaaaggagctgggggttggatggat

        18100     18110     18120     18130     18140     18150     18160     18170
                                CTGAAAGTTAGCTTGTAAAACTTTTTTTGAGTTGGATGGAGCTGGGAGATATTTTTGTTGTTCAGTTAAGAGATAGAT
PH207_w2_flanking(1>32227)  →   ctgaaagttagcttgtaaaactttttttgagttggatggagctgggagatatttttgttgttcagttaagagatagat
PH207_w2(1>16227)           →   ctgaaagttagcttgtaaaactttttttgagttggatggagctgggagatatttttgttgttcagttaagagatagat

        18180     18190     18200     18210     18220     18230     18240     18250
                                TAGATATCATATTTTATAGTAGATGGTTGTTAGGCAGTTGGGTTGTGCGGCAGTCCCAGTCTATCAGGCCCCGTTTGT
PH207_w2_flanking(1>32227)  →   tagatatcatattttatagtagatggttgttaggcagttgggttgtgcggcagtcccagtctatcaggccccgtttgt
PH207_w2(1>16227)           →   tagatatcatattttatagtagatggttgttaggcagttgggttgtgcggcagtcccagtctatcaggccccgtttgt

        18260     18270     18280     18290     18300     18310     18320     18330
                                TTGGTTAGAATTGAATTCTATTTTAATAATTATAATTTAGACAAAACTAATTAAGTTCATATATTTATATATGTAATA
PH207_w2_flanking(1>32227)  →   ttggttagaattgaattctattttaataattataatttagacaaaactaattaagttcatatatttatatatgtaata
PH207_w2(1>16227)           →   ttggttagaattgaattctattttaataattataatttagacaaaactaattaagttcatatatttatatatgtaata
```

```
18340 18350 18360 18370 18380 18390 18400
TATTTGTATATTATCTTAAATTATATGAGAGAGATAGTTATATACTACATTCATGTTATAGCGAAGCAAGTAGAAGAG
PH207_w2_flanking(1>32227) → tatttgtatattatcttaaattatatgagagagatagttatatactacattcatgttatagcgaagcaagtagaagag
PH207_w2(1>16227) → tatttgtatattatcttaaattatatgagagagatagttatatactacattcatgttatagcgaagcaagtagaagag

18410 18420 18430 18440 18450 18460 18470 18480
TATGCTATAAGTTGTACATCAGAAAAATAGCATGTAAATCTATAGAATCAATTTCTATCTCTCACCCCATGAATTTGA
PH207_w2_flanking(1>32227) → tatgctataagttgtacatcagaaaaatagcatgtaaatctatagaatcaatttctatctctcaccccatgaatttga
PH207_w2(1>16227) → tatgctataagttgtacatcagaaaaatagcatgtaaatctatagaatcaatttctatctctcaccccatgaatttga

18490 18500 18510 18520 18530 18540 18550 18560
GATAGGCTTATATGATAACTTTGGAAAGTGATGGAATGTCATATTCTAAAAAAATAGCCTATTCCATTAATAAGATTC
PH207_w2_flanking(1>32227) → gataggcttatatgataactttggaaagtgatggaatgtcatattctaaaaaaatagcctattccattaataagattc
PH207_w2(1>16227) → gataggcttatatgataactttggaaagtgatggaatgtcatattctaaaaaaatagcctattccattaataagattc

18570 18580 18590 18600 18610 18620 18630 18640
CAATTCCTCAAAATGAAAGGAAACAAACGAGGCCTCAGGGAATTTGCCTTTAGTTTGTATTAGGCATTGAAGGAATAA
PH207_w2_flanking(1>32227) → caattcctcaaaatgaaaggaaacaaacgaggcctcagggaatttgcctttagtttgtattaggcattgaaggaataa
PH207_w2(1>16227) → caattcctcaaaatgaaaggaaacaaacgaggcctcagggaatttgcctttagtttgtattaggcattgaaggaataa

18650 18660 18670 18680 18690 18700 18710 18720
AGATCTCCACACAGGGAGATTACCTTCCGTCGTCCTGGCTGACACATCCAGGCCAGCGACGGTGATCGCCCTCCCCCC
PH207_w2_flanking(1>32227) → agatctccacacagggagattaccttccgtcgtcctggctgacacatccaggccagcgacggtgatcgccctcccccc
PH207_w2(1>16227) → agatctccacacagggagattaccttccgtcgtcctggctgacacatccaggccagcgacggtgatcgccctcccccc
WT_617_1.617(1>1053) → CCAGCGACGGTGATCGCCCTCCCCCC
1754_5_f.617(1>402) → GGCTGACACATCCAGGCCAGCGACGGTGATCGCCCTCCCCCC
WT_w2_5_f.617(1>236) → GCTGACACATCCAGGCCAGCGACGGTGATCGCCCTCCCCCC

18730 18740 18750 18760 18770 18780 18790
TATGCCTTGCGATCTATCTCCTGTTCCTCTCATCCTCTTGTCTCTCCCCTGCGTACTCCCAAGAGAAGCAGCAGCAGA
PH207_w2_flanking(1>32227) → tatgccttgcgatctatctcctgttcctctcatcctcttgtctctcccctgcgtactcccaagagaagcagcagcaga
PH207_w2(1>16227) → tatgccttgcgatctatctcctgttcctctcatcctcttgtctctcccctgcgtactcccaagagaagcagcagcaga
WT_617_1.617(1>1053) → TATGCCTTGCGATCTATCTCCTGTTCCTCTCATCCTCTTGTCTCTCCCCTGCGTACTCCCAAGAGAAGCAGCAGCAGA
1754_5_f.617(1>402) → TATGCCTTGCGATCTATCTCCTGTTCCTCTCATCCTCTTGTCTCTCCCCTGCGTACTCCCAAGAGAAGCAGCAGCAGA
WT_w2_5_f.617(1>236) → TATGCCTTGCGATCTATCTCCTGTTCCTCTCATCCTCTTGTCTCTCCCCTGCGTACTCCCAAGAGAAGCAGCAGCAGA
1754_617_1.617(1>414) → CTTGTCTCTCCCCTGCGTACTCCCAAGAGAAGCAGCAGCAGA

18800 18810 18820 18830 18840 18850 18860 18870
CTACATCAGATAACCAAAATATGAGAAATGTTGTCAAGTGTTGCAAATTGTGAGCTGGTTCCTTTAAATAGTGTGGTA
PH207_w2_flanking(1>32227) → ctacatcagataaccaaaatatgagaaatgttgtcaagtgttgcaaattgtgagctggttcctttaaatagtgtggta
PH207_w2(1>16227) → ctacatcagataaccaaaatatgagaaatgttgtcaagtgttgcaaattgtgagctggttcctttaaatagtgtggta
WT_617_1.617(1>1053) → CTACATCAGATAACCAAAATATGAGAAATGTTGTCAAGTGTTGCAAATTGTGAGCTGGTTCCTTTAAATAGTGTGGTA
1754_5_f.617(1>402) → CTACATCAGATAACCAAAATATGAGAAATGTTGTCAAGTGTTGCAAATTGTGAGCTGGTTCCTTTAAATAGTGTGGTA
WT_w2_5_f.617(1>236) → CTACATCAGATAACCAAAATATGAGAAATGTTGTCAAGTGTTGCAAATTGTGAGCTGGTTCCTTTAAATAGTGTGGTA
1754_617_1.617(1>414) → CTACATCAGATAACCAAAATATGAGAAATGTTGTCAAGTGTTGCAAATTGTGAGCTGGTTCCTTTAAATAGTGTGGTA

18880 18890 18900 18910 18920 18930 18940 18950
CTGTGATACCAACATCAAATAATTTGTTGAACCTGTGATGTAAATTACTCCATGACAGAACCAACCTGCACTTGAAAA
PH207_w2_flanking(1>32227) → ctgtgataccaacatcaaataatttgttgaacctgtgatgtaaattactccatgacagaaccaacctgcacttgaaaa
PH207_w2(1>16227) → ctgtgataccaacatcaaataatttgttgaacctgtgatgtaaattactccatgacagaaccaacctgcacttgaaaa
WT_617_1.617(1>1053) → CTGTGATACCAACATCAAATAATTTGTTGAACCTGTGATGTAAATTACTCCATGACAGAACCAACCTGCACTTGAAAA
1754_5_f.617(1>402) → CTGTGATACCAACATCAAATAATTTGTTGAACCTGTGATGTAAATTACTCCATGACAGAACCAACCTGCACTTGAAAA
WT_w2_5_f.617(1>236) → CTGTGATACCAACATCAAATAATTTGTTGAACCTGTGAT
1754_617_1.617(1>414) → CTGTGATACCAACATCAAATAATTTGTTGAACCTGTGATGTAAATTACTCCATGACAGAACCAACCTGCACTTGAAAA

18960 18970 18980 18990 19000 19010 19020 19030
GGATAGGTATAAAATCCGCCAAGCTTTTGTTGCAGCTCCAGGGAACACTCTTATTGTTGCTGATTATGGTCAGGTATG
PH207_w2_flanking(1>32227) → ggataggtataaaatccgccaagcttttgttgcagctccagggaacactcttattgttgctgattatggtcaggtatg
PH207_w2(1>16227) → ggataggtataaaatccgccaagcttttgttgcagctccagggaacactcttattgttgctgattatggtcaggtatg
WT_617_1.617(1>1053) → GGATAGGTATAAAATCCGCCAAGCTTTTGTTGCAGCTCCAGGGAACACTCTTATTGTTGCTGATTATGGTCAGGTATG
1754_5_f.617(1>402) → GGATAGGTATAAAATCCGCCAAGCTTTTGTTGCAGCTCCAGGGAACACTCTTATTGTTGCTGATTATGGTCAGGTATG
1754_617_1.617(1>414) → GGATAGGTATAAAATCCGCCAAGCTTTTGTTGCAGCTCCAGGGAACACTCTTATTGTTGCTGATTATGGTCAGGTATG
```

```
          19040     19050     19060     19070     19080     19090     19100     19110
          CTTGTGCACAGTAGAGTTTTTGAATGTTCATATGCTGATGATACTAGTTCCAATTAAAAAGACAAACTTGTTCTGCGT
PH207_w2_flanking(1>32227) →  cttgtgcacagtagagtttttgaatgttcatatgctgatgatactagttccaattaaaaagacaaacttgttctgcgt
PH207_w2(1>16227) →           cttgtgcacagtagagtttttgaatgttcatatgctgatgatactagttccaattaaaaagacaaacttgttctgcgt
WT_617_1.617(1>1053) →        CTTGTGCACAGTAGAGTTTTTGAATGTTCATATGCTGATGATACTAGTTCCAATTAAAAAGACAAACTTGTTCTGCGT
WT_604_2.604(1>1059) ←                                                        CTAGTTCCAATTAAAAAGACAAACTTGTTCTGCGT
1754_5_f.617(1>402) →         CTTGTGCACAGTAGAGTTTTTGAATGTTCATATGCTGATGATACTAGT
1754_617_1.617(1>414) →       CTTGTGCACAGTAGAGTTTTTGAATGTTCATATGCTGATGATACTAGTTCCAATTAAAAAGACAAACTTGTTCTGCGT

            19120     19130     19140     19150     19160     19170     19180
          GTAGCTGGAACTCAGGATCTTGGCGCATCTTGCTGATTGCAAAAGCATGTTAGATGCTTTTAAAGCTGGCGGTGACTT
PH207_w2_flanking(1>32227) →  gtagctggaactcaggatcttggcgcatcttgctgattgcaaaagcatgttagatgcttttaaagctggcggtgactt
PH207_w2(1>16227) →           gtagctggaactcaggatcttggcgcatcttgctgattgcaaaagcatgttagatgcttttaaagctggcggtgactt
WT_617_1.617(1>1053) →        GTAGCTGGAACTCAGGATCTTGGCGCATCTTGCTGATTGCAAAAGCATGTTAGATGCTTTTAAAGCTGGCGGTGACTT
WT_604_2.604(1>1059) ←        GTAGCTGGAACTCAGGATCTTGGCGCATCTTGCTGATTGCAAAAGCATGTTAGATGCTTTTAAAGCTGGCGGTGACTT
1754_617_1.617(1>414) →       GTAGCTGGAACTCAGGATCTTGGCGCATCTTGCTGATTGCAAAAGCATGTTAGATGCTTT

       19190     19200     19210     19220     19230     19240     19250     19260
          CCATTCCAGGACAGCCATGAACATGTATCAGCATATTCGTGAGGCTGTTGAAGAAGAGAGAGTAATTCTTGAGTGGCA
PH207_w2_flanking(1>32227) →  ccattccaggacagccatgaacatgtatcagcatattcgtgaggctgttgaagaagagagagtaattcttgagtggca
PH207_w2(1>16227) →           ccattccaggacagccatgaacatgtatcagcatattcgtgaggctgttgaagaagagagagtaattcttgagtggca
WT_617_1.617(1>1053) →        CCATTCCAGGACAGCCATGAACATGTATCAGCATATTCGTGAGGCTGTTGAAGAAGAGAGAGTAATTCTTGAGTGGCA
WT_604_2.604(1>1059) ←        CCATTCCAGGACAGCCATGAACATGTATCAGCATATTCGTGAGGCTGTTGAAGAAGAGAGAGTAATTCTTGAGTGGCA

        19270     19280     19290     19300     19310     19320     19330     19340
          TCCTCAACCCGGTCAAGAGAAACCTCCTGTGCCGTTGTTGAAGGTATGCTAGCTTTAATGGTGTTTCTGAGGATTATG
PH207_w2_flanking(1>32227) →  tcctcaacccggtcaagagaaacctcctgtgccgttgttgaaggtatgctagctttaatggtgtttctgaggattatg
PH207_w2(1>16227) →           tcctcaacccggtcaagagaaacctcctgtgccgttgttgaaggtatgctagctttaatggtgtttctgaggattatg
WT_617_1.617(1>1053) →        TCCTCAACCCGGTCAAGAGAAACCTCCTGTGCCGTTGTTGAAGGTATGCTAGCTTTAATGGTGTTTCTGAGGATTATG
WT_604_2.604(1>1059) ←        TCCTCAACCCGGTCAAGAGAAACCTCCTGTGCCGTTGTTGAAGGTATGCTAGCTTTAATGGTGTTTCTGAGGATTATG

         19350     19360     19370     19380     19390     19400     19410     19420
          TTGATTCCTTCTAAATGTTAATATTATGTTCAGTGCTACTAGATTATTAACTTTTGTACTGCACTTCCAACCACATAC
PH207_w2_flanking(1>32227) →  ttgattccttctaaatgttaatattatgttcagtgctactagattattaacttttgtactgcacttccaaccacatac
PH207_w2(1>16227) →           ttgattccttctaaatgttaatattatgttcagtgctactagattattaacttttgtactgcacttccaaccacatac
WT_617_1.617(1>1053) →        TTGATTCCTTCTAAATGTTAATATTATGTTCAGTGCTACTAGATTATTAACTTTTGTACTGCACTTCCAACCACATAC
WT_604_2.604(1>1059) ←        TTGATTCCTTCTAAATGTTAATATTATGTTCAGTGCTACTAGATTATTAACTTTTGTACTGCACTTCCAACCACATAC

          19430     19440     19450     19460     19470     19480     19490     19500
          AGTTTTTTTCCCTTATTTCTTTTATCTTCCATGTCGTCTGGTCCAGAACTTGTACCGTGGAGTTAATTATGGATATGG
PH207_w2_flanking(1>32227) →  agtttttttcccttatttcttttatcttccatgtcgtctggtccagaacttgtaccgtggagttaattatggatatgg
PH207_w2(1>16227) →           agtttttttcccttatttcttttatcttccatgtcgtctggtccagaacttgtaccgtggagttaattatggatatgg
WT_617_1.617(1>1053) →        AGTTTTTTTCCCTTATTTCTTTTATCTTCCATGTCGTCTGGTCCAGAACTTGTACCGTGGAGTTAATTATGGATATGG
WT_604_2.604(1>1059) ←        AGTTTTTTTCCCTTATTTCTTTTATCTTCCATGTCGTCTGGTCCAGAACTTGTACCGTGGAGTTAATTATGGATATGG

            19510     19520     19530     19540     19550     19560     19570
          ATCTAGGAAATATTATTAGCATGAAAATATACATGCGTGTATGGAATAAAATGGACTCTTATGCTTTTCTTGTGACGT
PH207_w2_flanking(1>32227) →  atctaggaaatattattagcatgaaaatatacatgcgtgtatggaataaaatggactcttatgcttttcttgtgacgt
PH207_w2(1>16227) →           atctaggaaatattattagcatgaaaatatacatgcgtgtatggaataaaatggactcttatgcttttcttgtgacgt
WT_617_1.617(1>1053) →        ATCTAGGAAATATTATTAGCATGAAAATATACATGCGTGTATGGAATAAAATGGACTCTTATGCTTTTCTTGTGACGT
WT_604_2.604(1>1059) ←        ATCTAGGAAATATTATTAGCATGAAAATATACATGCGTGTATGGAATAAAATGGACTCTTATGCTTTTCTTGTGACGT

       19580     19590     19600     19610     19620     19630     19640     19650
          AGGATGCTTTTGGTGCTGAGAGGAGGAAAGCTAGATGCTAAATTTCTCCATTGCATATGGGAAAACGGCCCATGGGC
PH207_w2_flanking(1>32227) →  aggatgcttttggtgctgagaggaggaaagctagatgctaaatttctccattgcatatgggaaaacggcccatgggc
PH207_w2(1>16227) →           aggatgcttttggtgctgagaggaggaaagctagatgctaaatttctccattgcatatgggaaaacggcccatgggc
WT_617_1.617(1>1053) →        AGGATGCTTTTGGTGCTGAGAGGAGGAAAGCTAGATGCTAAATTTCTCCATTGCATATGGGAAAACGGCCCATGGGC
WT_604_2.604(1>1059) ←        AGGATGCTTTTGGTGCTGAGAGGAGGAAAGCTAGATGCTAAATTTCTCCATTGCATATGGGAAAACGGCCCATGGGC
1754_604_2.604(1>439) ←                                                             CATATGGGAAAACGGCCCATGGGC
```

```
                                      19660     19670     19680     19690     19700     19710     19720     19730
                                      TTGCTCGTGATTGGAAGGTACAAACTTGTCAAACAAACATAGCTATAAGTTGATAAGGAGCAATCAACTTTATTGTTC
PH207_w2_flanking(1>32227)      →     ttgctcgtgattggaaggtacaaacttgtcaaacaaacatagctataagttgataaggagcaatcaactttattgttc
PH207_w2(1>16227)               →     ttgctcgtgattggaaggtacaaacttgtcaaacaaacatagctataagttgataaggagcaatcaactttattgttc
WT_617_1.617(1>1053)            →     TTGCTCGTGATTGGAAGGTACAAACTTGTCAAACAAACTTAGCTATAAGTTGATAAGGAGCAATCAACTTTATTGTTC
WT_604_2.604(1>1059)            ←     TTGCTCGTGATTGGAAGGTACAAACTTGTCAAACAAACATAGCTATAAGTTGATAAGGAGCAATCAACTTTATTGTTC
1754_604_2.604(1>439)           ←     TTGCTCGTGATTGGAAGGTACAAACTTGTCAAACAAACATAGCTATAAGTTGATAAGGAGCAATCAACTTTATTGTTC
```

```
                                        19740     19750     19760     19770     19780     19790     19800     19810
                                      ATTACACTTAAATCTGAGCTTTGGCCTTTAGATTACTGGGTTTGTGACAGTCAGCTAGAAACCTGAAAACAACACACT
PH207_w2_flanking(1>32227)      →     attacacttaaatctgagctttggcctttagattactgggtttgtgacagtcagctagaaacctgaaaacaacacact
PH207_w2(1>16227)               →     attacacttaaatctgagctttggcctttagattactgggtttgtgacagtcagctagaaacctgaaaacaacacact
WT_617_1.617(1>1053)            →     ATTACACTTAAAT
WT_604_2.604(1>1059)            ←     ATTACACTTAAATCTGAGCTTTGGCCTTTAGATTACTGGGTTTGTGACAGTCAGCTAGAAACCTGAAAACAACACACT
1754_604_2.604(1>439)           ←     ATTACACTTAAATCTGAGCTTTGGCCTTTAGATTACTGGGTTTGTGACAGTCAGCTAGAAACCTGAAAACAACACACT
1754_5_r.604(1>366)             ←                                               TGTGACAGTCAGCTAGAAACCTGAAAACAACACACT
```

```
                                          19820     19830     19840     19850     19860     19870     19880     19890
                                      TGCTGAGTAGGTATGTTTACAAGAAATATAGCCTTCATTGCTTCTTGTAACCCTTCCTCTCTTGTCAGGTTTCTGTTA
PH207_w2_flanking(1>32227)      →     tgctgagtaggtatgtttacaagaaatatagccttcattgcttcttgtaacccttcctctcttgtcaggtttctgtta
PH207_w2(1>16227)               →     tgctgagtaggtatgtttacaagaaatatagccttcattgcttcttgtaacccttcctctcttgtcaggtttctgtta
WT_604_2.604(1>1059)            ←     TGCTGAGTAGGTATGTTTACAAGAAATATAGCCTTCATTGCTTCTTGTAACCCTTCCTCTCTTGTCAGGTTTCTGTTA
1754_604_2.604(1>439)           ←     TGCTGAGTAGGTATGTTTACAAGAAATATAGCCTTCATTGCTTCTTGTAACCCTTCCTCTCTTGTCAGGTTTCTGTTA
1754_5_r.604(1>366)             ←     TGCTGAGTAGGTATGTTTACAAGAAATATAGCCTTCATTGCTTCTTGTAACCCTTCCTCTCTTGTCAGGTTTCTGTTA
WT_w2_5_r.604(1>253)            ←                                                                    GGTTTCTGTTA
```

```
                                            19900     19910     19920     19930     19940     19950     19960
                                      AGGAAGCAAAAGATACACTGAAACTCTGGTATAGCGATAGAAAGGAAGTTCTGGCTTGGCAAATGAAGCAGAAACAGC
PH207_w2_flanking(1>32227)      →     aggaagcaaaagatacactgaaactctggtatagcgatagaaaggaagttctggcttggcaaatgaagcagaaacagc
PH207_w2(1>16227)               →     aggaagcaaaagatacactgaaactctggtatagcgatagaaaggaagttctggcttggcaaatgaagcagaaacagc
WT_604_2.604(1>1059)            ←     AGGAAGCAAAAGATACACTGAAACTCTGGTATAGCGATAGAAAGGAAGTTCTGGCTTGGCAAATGAAGCAGAAACAGC
1754_604_2.604(1>439)           ←     AGGAAGCAAAAGATACACTGAAACTCTGGTATAGCGATAGAAAGGAAGTTCTGGCTTGGCAAATGAAGCAGAAACAGC
1754_5_r.604(1>366)             ←     AGGAAGCAAAAGATACACTGAAACTCTGGTATAGCGATAGAAAGGAAGTTCTGGCTTGGCAAATGAAGCAGAAACAGC
WT_w2_5_r.604(1>253)            ←     AGGAAGCAAAAGATACACTGAAACTCTGGTATAGCGATAGAAAGGAAGTTCTGGCTTGGCAAATGAAGCAGAAACAGC
```

```
                                    19970     19980     19990     20000     20010     20020     20030     20040
                                      TAGCACATGAGAAGTGTGAAGTTTATACTTTGCTTGGGCGATCACGCCGTTTTCCAAATATGGCTCATGCAACTTCTG
PH207_w2_flanking(1>32227)      →     tagcacatgagaagtgtgaagtttatactttgcttgggcgatcacgccgttttccaaatatggctcatgcaacttctg
PH207_w2(1>16227)               →     tagcacatgagaagtgtgaagtttatactttgcttgggcgatcacgccgttttccaaatatggctcatgcaacttctg
WT_604_2.604(1>1059)            ←     TAGCACATGAGAAGTGTGAAGTTTATACTTTGCTTGGGCGATCACGCCGTTTTCCAAATATGGCTCATGCAACTTCTG
1754_604_2.604(1>439)           ←     TAGCACATGAGAAGTGTGAAGTTTATACTTTGCTTGGGCGATCACGCCGTTTTCCAAATATGGCTCATGCAACTTCTG
1754_5_r.604(1>366)             ←     TAGCACATGAGAAGTGTGAAGTTTATACTTTGCTTGGGCGATCACGCCGTTTTCCAAATATGGCTCATGCAACTTCTG
WT_w2_5_r.604(1>253)            ←     TAGCACATGAGAAGTGTGAAGTTTATACTTTGCTTGGGCGATCACGCCGTTTTCCAAATATGGCTCATGCAACTTCTG
```

```
                                      20050     20060     20070     20080     20090     20100     20110     20120
                                      GCCAAAGGGGTCACATTGAGCGTGCTGCTATCAATGCTCCTGTACAGGTGATTACTGTTTATTGCAACTC-GTTGTTG
PH207_w2_flanking(1>32227)      →     gccaaaggggtcacattgagcgtgctgctatcaatgctcctgtacaggtgattactgtttattgcaactc-gttgttg
PH207_w2(1>16227)               →     gccaaaggggtcacattgagcgtgctgctatcaatgctcctgtacaggtgattactgtttattgcaactc-gttgttg
WT_604_2.604(1>1059)            ←     GCCAAAGGGGTCACATTGAGCGTGCTGCTATCAATGCTCCTGTACAGGTGATTACTGTTTATTGCAACTC-GTTGTTG
1754_604_2.604(1>439)           ←     GCCAAAGGTGTCACATTGAGC-TG-TG
1754_5_r.604(1>366)             ←     GCCAAAGGGGTCACATTGAGCGTGCTGCTATCAATGCTCCTGTACAGGTGATTACTGTTTATTGCAACTC-GTTGTTG
WT_w2_5_r.604(1>253)            ←     GCCAAAGGGGTCACATTGAGCGTGCTGCTATCAATGCTCCTGTACAGGTGATTACTGTTTATTGCAACTCTGTTGTTG
```

```
                                        20130     20140     20150     20160     20170     20180     20190     20200
                                      AAATCAATTTTGTGCTGAAATTATTTTCTTTGAACACCACAGGGCAGTGCAGCTGATGTTGCTATGTGTGCAATGCTT
PH207_w2_flanking(1>32227)      →     aaatcaattttgtgctgaaattattttctttgaacaccacagggcagtgcagctgatgttgctatgtgtgcaatgctt
PH207_w2(1>16227)               →     aaatcaattttgtgctgaaattattttctttgaacaccacagggcagtgcagctgatgttgctatgtgtgcaatgctt
WT_604_2.604(1>1059)            ←     AAATCAATTTT
1754_5_r.604(1>366)             ←     AAATCAATTTTGTGCTGAA
WT_w2_5_r.604(1>253)            ←     AAATCAAT
```

```
                                          20210     20220     20230     20240     20250     20260     20270     20280
                                      GAGATAGACAGGAATACTCGTCTTaAGCAGCTTGGTTGGACGCTACTCTTGCAGGTACTTTATGCATAAGTTTAGGCC
PH207_w2_flanking(1>32227)      →     gagatagacaggaatactcgtcttaagcagcttggttggacgctactcttgcaggtactttatgcataagtttaggcc
PH207_w2(1>16227)               →     gagatagacaggaatactcgtcttaagcagcttggttggacgctactcttgcaggtactttatgcataagtttaggcc
```

```
                   20210     20220     20230     20240     20250     20260     20270     20280
                   GAGATAGACAGGAATACTCGTCTTaAGCAGCTTGGTTGGACGCTACTCTTGCAGGTACTTTATGCATAAGTTTAGGCC
1754_6_f.595(1>325)      →                TCGTCTT-AGCAGCTTGGTTGGACGCTACTCTTGCAGGTACTTTATGCATAAGTTTAGGCC
WT_w2_6_f.595(1>275)     →                TCGTCTT-AGCAGCTTGGTTGGACGCTACTCTTGCAGGTACTTTATGCATAAGTTTAGGCC

                   20290     20300     20310     20320     20330     20340     20350
                   TGTTCACTGCTTAAATGTTGGCGTAGTTTGTTTTTCTCTTTAAAACTGTGAAGGGCACGACTTTTGTAACTTGTGTAT
PH207_w2_flanking(1>32227) →  tgttcactgcttaaatgttggcgtagtttgtttttctctttaaaactgtgaagggcacgacttttgtaacttgtgtat
PH207_w2(1>16227)          →  tgttcactgcttaaatgttggcgtagtttgtttttctctttaaaactgtgaagggcacgacttttgtaacttgtgtat
1754_6_f.595(1>325)        →  TGTTCACTGCTTAAATGTTGGCGTAGTTTGTTTTTCTCTTTAAAACTGTAAAGGGCACGACTTTTGTAACTTGTGTAT
WT_w2_6_f.595(1>275)       →  TGTTCACTGCTTAAATGTTGGCGTAGTTTGTTTTTCTCTTTAAAACTGTGAAGGGCACGACTTTTGTAACTTGTGTAT

                   20360     20370     20380     20390     20400     20410     20420     20430
                   TTAACGACTTCTGTAACTTGTGTATTTAACGACTTCTGTAACTTGTGTATTTGACATCTGGTATTCGGCATTCTGAGT
PH207_w2_flanking(1>32227) →  ttaacgacttctgtaacttgtgtatttaacgacttctgtaacttgtgtatttgacatctggtattcggcattctgagt
PH207_w2(1>16227)          →  ttaacgacttctgtaacttgtgtatttaacgacttctgtaacttgtgtatttgacatctggtattcggcattctgagt
1754_6_f.595(1>325)        →  TTAACGACTTCTGTAACTTGTGTATTTAACGACTTCTGTAACTTGTGTATTTGACATCTGGTATTCGGCATTCAGAGT
WT_w2_6_f.595(1>275)       →  TTAACGACTTCTGTAACTTGTGTATTTAACGACTTCTGTAACTTGTGTATTTGACATCTGGTATTCGGCATTCTGAGT

                   20440     20450     20460     20470     20480     20490     20500     20510
                   TAAAATGGTCACATTTGGTGTTTGCATAGTTTGCAGCCTTTTTTTAATTTATAAATGCAACATAACTTTAGACCTTCA
PH207_w2_flanking(1>32227) →  taaaatggtcacatttggtgtttgcatagtttgcagcctttttttaatttataaatgcaacataactttagaccttca
PH207_w2(1>16227)          →  taaaatggtcacatttggtgtttgcatagtttgcagcctttttttaatttataaatgcaacataactttagaccttca
1754_6_f.595(1>325)        →  TAAAATGGTCACATTTGGTGTTTGCATAGTTTGCAGCCTTTTTTTAATTTATAAATGCAACATAACTTTAGACCTTCA
WT_w2_6_f.595(1>275)       →  TAAAATGGTCACATTTGGTGTTTGCATAGTTTGCAGCCTTTTTTTAATTTATAAATGCA

                   20520     20530     20540     20550     20560     20570     20580     20590
                   GAACTGACAGCTCTATCTTTCAGGTGCATGATGAAGTGATACTGGAAGGGCCTTCAGAGTCTGCGGAGCTGGCCAAGT
PH207_w2_flanking(1>32227) →  gaactgacagctctatctttcaggtgcatgatgaagtgatactggaagggccttcagagtctgcggagctggccaagt
PH207_w2(1>16227)          →  gaactgacagctctatctttcaggtgcatgatgaagtgatactggaagggccttcagagtctgcggagctggccaagt
1754_6_f.595(1>325)        →  GAACTGACAGCTCTATCTTTCAGGTGCATGA

                   20600     20610     20620     20630     20640     20650     20660     20670
                   CCATAGTTGTTGAGTGCATGTCTAAGCCCTTCTATGGCACCAATATCCTGAAGGTCGACCTTGCTGTTGATGCCAAGT
PH207_w2_flanking(1>32227) →  ccatagttgttgagtgcatgtctaagcccttctatggcaccaatatcctgaaggtcgaccttgctgttgatgccaagt
PH207_w2(1>16227)          →  ccatagttgttgagtgcatgtctaagcccttctatggcaccaatatcctgaaggtcgaccttgctgttgatgccaagt

                   20680     20690     20700     20710     20720     20730     20740
                   GTGCACAGAATTGGTATGCGGCCAAATAGACCTGTTAAGTGTCCCAGAAGGGCGTCAATTCCTTCGGTGAAACTCGGT
PH207_w2_flanking(1>32227) →  gtgcacagaattggtatgcggccaaatagacctgttaagtgtcccagaagggcgtcaattccttcggtgaaactcggt
PH207_w2(1>16227)          →  gtgcacagaattggtatgcggccaaatagacctgttaagtgtcccagaagggcgtcaattccttcggtgaaactcggt

                   20750     20760     20770     20780     20790     20800     20810     20820
                   GGATTTTGAACTGGTATCTGCTGGCTGCTGCTAGTAGCCTAGTGAGCTCATCTCCACGTGGATGCCATGCACTGATTA
PH207_w2_flanking(1>32227) →  ggattttgaactggtatctgctggctgctgctagtagcctagtgagctcatctccacgtggatgccatgcactgatta
PH207_w2(1>16227)          →  ggattttgaactggtatctgctggctgctgctagtagcctagtgagctcatctccacgtggatgccatgcactgatta

                   20830     20840     20850     20860     20870     20880     20890     20900
                   TTTCGCTGTTGATCTGGACAGTGAGGGGTCAGCACTGAGCAGCGAAGGCACTGTACACTATTCCGTATTCCCTATAGG
PH207_w2_flanking(1>32227) →  tttcgctgttgatctggacagtgaggggtcagcactgagcagcgaaggcactgtacactattccgtattccctatagg
PH207_w2(1>16227)          →  tttcgctgttgatctggacagtgaggggtcagcactgagcagcgaaggcactgtacactattccgtattccctatagg

                   20910     20920     20930     20940     20950     20960     20970     20980
                   TCGGGTAGCTTCACCTTCCACACTCCCTGTTAGGATCTCTCGATCATTCATGCCTGATGTAGTTGGTCGTCCTGGATA
PH207_w2_flanking(1>32227) →  tcgggtagcttcaccttccacactccctgttaggatctctcgatcattcatgcctgatgtagttggtcgtcctggata
PH207_w2(1>16227)          →  tcgggtagcttcaccttccacactccctgttaggatctctcgatcattcatgcctgatgtagttggtcgtcctggata
```

```
                                  20990     21000     21010     21020     21030     21040     21050     21060
                                  GCAGTGAAGACTAAGCATGCCGTGTTCATAAGATTTATTTAGCATGCTGATGTGTTGAGTTGCTGACCAGTTCTGCTG
PH207_w2_flanking(1>32227)   →    gcagtgaagactaagcatgccgtgttcataagatttatttagcatgctgatgtgttgagttgctgaccagttctgctg
PH207_w2(1>16227)            →    gcagtgaagactaagcatgccgtgttcataagatttatttagcatgctgatgtgttgagttgctgaccagttctgctg

                                  21070     21080     21090     21100     21110     21120     21130
                                  ACGTCTAACACTGTTGCACGCACTGCAACAATGTTTTGATCACAAGAGAATGTAGTTGATTGTGAGCGTTCTTAATCT
PH207_w2_flanking(1>32227)   →    acgtctaacactgttgcacgcactgcaacaatgttttgatcacaagagaatgtagttgattgtgagcgttcttaatct
PH207_w2(1>16227)            →    acgtctaacactgttgcacgcactgcaacaatgttttgatcacaagagaatgtagttgattgtgagcgttcttaatct

                                  21140     21150     21160     21170     21180     21190     21200     21210
                                  CCTTATTTCTGCTGTTGAACTGTGCGTCTTGTTAATTGTATCTTCTCTAAGCATTGTCCGCACGGCAGGGTGCCAGGG
PH207_w2_flanking(1>32227)   →    ccttatttctgctgttgaactgtgcgtcttgttaattgtatcttctctaagcattgtccgcacggcagggtgccaggg
PH207_w2(1>16227)            →    ccttatttctgctgttgaactgtgcgtcttgttaattgtatcttctctaagcattgtccgcacggcagggtgccaggg

                                  21220     21230     21240     21250     21260     21270     21280     21290
                                  TTGGGCAAATGTTTCAGTtcgtcataaacttaagcgagctcctgatctcaccagctttagcacccattagcaacacaa
PH207_w2_flanking(1>32227)   →    ttgggcaaatgtttcagttcgtcataaacttaagcgagctcctgatctcaccagctttagcacccattagcaacacaa
PH207_w2(1>16227)            →    ttgggcaaatgtttcagt

                                  21300     21310     21320     21330     21340     21350     21360     21370
                                  gtggtgctaaagtttagtacgtgggatccaaacaccccctaactaatactgtatctaatagtgatactaggatcacat
PH207_w2_flanking(1>32227)   →    gtggtgctaaagtttagtacgtgggatccaaacaccccctaactaatactgtatctaatagtgatactaggatcacat

                                  21380     21390     21400     21410     21420     21430     21440     21450
                                  cataatgtgataaagatgaaacaatgtcggcgtagcaataaactgaccagcgaagaatattgctcccgttcagaacgt
PH207_w2_flanking(1>32227)   →    cataatgtgataaagatgaaacaatgtcggcgtagcaataaactgaccagcgaagaatattgctcccgttcagaacgt

                                  21460     21470     21480     21490     21500     21510     21520
                                  gaagcagcagataacagcaaaaccagaacaaaaccatacgccttttgttgtttatgctcaaatcatgttaaaacaata
PH207_w2_flanking(1>32227)   →    gaagcagcagataacagcaaaaccagaacaaaaccatacgccttttgttgtttatgctcaaatcatgttaaaacaata

                                  21530     21540     21550     21560     21570     21580     21590     21600
                                  ttccctgtcaatctctaaatccgatgaaacaacacacattgcagaggtccgaacagagagatgaatcaatcaccctct
PH207_w2_flanking(1>32227)   →    ttccctgtcaatctctaaatccgatgaaacaacacacattgcagaggtccgaacagagagatgaatcaatcaccctct

                                  21610     21620     21630     21640     21650     21660     21670     21680
                                  gactgcgagctgctgcaaatggtggacgaatcaccacccaagctacccttgcggtggtcaggaggatacagttcgagt
PH207_w2_flanking(1>32227)   →    gactgcgagctgctgcaaatggtggacgaatcaccacccaagctacccttgcggtggtcaggaggatacagttcgagt

                                  21690     21700     21710     21720     21730     21740     21750     21760
                                  tcaggcacctgGGTGCTGTTTGGTTCAAAAAATGTAACGCAAATGGTAATGGTAATGGTTTCGGCTCGATTACTGACG
PH207_w2_flanking(1>32227)   →    tcaggcacctgggtgctgtttggttcaaaaaatgtaacgcaaatggtaatggtaatggtttcggctcgattactgacg
1754_6_r.605(1>256)          ←               GGTGCTGTTTGGTTCAAAAAATGTAACGCAAATGGTAATGGTAATGGTTTCGGCTCGATTACTGACG

                                  21770     21780     21790     21800     21810     21820     21830     21840
                                  GTAACAAATTTGAATAGGCTGGTATCAAATTATAGTGTGGTATCTGATTACGGTTGGACTAAAACAAACATGATTTAA
PH207_w2_flanking(1>32227)   →    gtaacaaatttgaataggctggtatcaaattatagtgtggtatctgattacggttggactaaaacaaacatgatttaa
1754_6_r.605(1>256)          ←    GTAACAAATTTGAATAGGCTGGTATCAAATTATAGTGTGGTATCTGATTACGGTTGGACTAAAACAAACATGATTTAA
WT_w2_6_r.605(1>129)         ←                                                                AAAACAAACATGATTTAA

                                  21850     21860     21870     21880     21890     21900     21910
                                  CGTTATCTGTTACCCATTACGTTACAAATATGTGAACCAAACGGCACCCTGGTGGCTATACCTCAGTATGTAGGGGTT
PH207_w2_flanking(1>32227)   →    cgttatctgttacccattacgttacaaatatgtgaaccaaacggcaccctggtggctatacctcagtatgtaggggtt
1754_6_r.605(1>256)          ←    CGTTATCTGTTACCCATTACGTTACAAATATGTGAACCAAACGGCACCCTGGTGGCTATACCTCAGTATGTAGGGGTT
WT_w2_6_r.605(1>129)         ←    CGTTATCTGTTACCCATTACGTTACAAATATGTGAACCAAACGGCACCCTGGTGGCTATACCTCAGTATGTAGGGGTT
```

```
                              21920     21930     21940     21950     21960     21970     21980     21990
                              GTTTGGTTCACCACGTGGTCTCTGTCCTACGGTtgcggacgctaagtcatcgtcgttctttctctcactacacctatg
PH207_w2_flanking(1>32227)  → gtttggttcaccacgtggtctctgtcctacggttgcggacgctaagtcatcgtcgttctttctctcactacacctatg
1754_6_r.605(1>256)         ← GTTTGGTTCACCACGTGGTCTCTGTCCTACGGT
WT_w2_6_r.605(1>129)        ← GTTTGGTTCACCACGTGGTCTCTGTCCTACGGT

                              22000     22010     22020     22030     22040     22050     22060     22070
                              gtgctagagtatctacgtcgtaacaagtttcatctcagtatatatggatatcatagagacattactgctattgttgta
PH207_w2_flanking(1>32227)  → gtgctagagtatctacgtcgtaacaagtttcatctcagtatatatggatatcatagagacattactgctattgttgta

                              22080     22090     22100     22110     22120     22130     22140     22150
                              tcgattgatcaacaacgaacgtgctgtcgtgcgaggacgactgctttcattacacgtatccatttcactacaacaata
PH207_w2_flanking(1>32227)  → tcgattgatcaacaacgaacgtgctgtcgtgcgaggacgactgctttcattacacgtatccatttcactacaacaata

                              22160     22170     22180     22190     22200     22210     22220     22230
                              gaggagacattcatgcacaatttttttatttggctggtctgtgtatatagacaatacatgttataggttggatcttgt
PH207_w2_flanking(1>32227)  → gaggagacattcatgcacaatttttttatttggctggtctgtgtatatagacaatacatgttataggttggatcttgt

                              22240     22250     22260     22270     22280     22290     22300
                              atcatggaatgagacggaagatgtagttgtccatacaatcacacaacttatttacccttatcgtctgaaatcttcatt
PH207_w2_flanking(1>32227)  → atcatggaatgagacggaagatgtagttgtccatacaatcacacaacttatttacccttatcgtctgaaatcttcatt

                              22310     22320     22330     22340     22350     22360     22370     22380
                              ggagcgttgaatcagttcactttctcttctaaccactcctacaacttatccctatcgactggtatcaggttaacatcg
PH207_w2_flanking(1>32227)  → ggagcgttgaatcagttcactttctcttctaaccactcctacaacttatccctatcgactggtatcaggttaacatcg

                              22390     22400     22410     22420     22430     22440     22450     22460
                              tatacctttcaaattgagaaaaggtaataaatctgagttggatatactattatcgttcgatctgacgtaaatatcaat
PH207_w2_flanking(1>32227)  → tatacctttcaaattgagaaaaggtaataaatctgagttggatatactattatcgttcgatctgacgtaaatatcaat

                              22470     22480     22490     22500     22510     22520     22530     22540
                              acgattgatggtgaaaacagagatatttaaaatatcttagaattagcttgatttgatcaacctgatgagatgttcata
PH207_w2_flanking(1>32227)  → acgattgatggtgaaaacagagatatttaaaatatcttagaattagcttgatttgatcaacctgatgagatgttcata

                              22550     22560     22570     22580     22590     22600     22610     22620
                              gctatttcattgatatgatcgatgaaccaataggttgttcggaatgtctcagactcccaggactcctgtttgcataca
PH207_w2_flanking(1>32227)  → gctatttcattgatatgatcgatgaaccaataggttgttcggaatgtctcagactcccaggactcctgtttgcataca

                              22630     22640     22650     22660     22670     22680     22690
                              acacatagattgcttaaataatctcttttcatattacacgatagtatctgtttctccgtctaatttggttgtacaggg
PH207_w2_flanking(1>32227)  → acacatagattgcttaaataatctcttttcatattacacgatagtatctgtttctccgtctaatttggttgtacaggg

                              22700     22710     22720     22730     22740     22750     22760     22770
                              aatattatgaatggtataacccaaatatatatgtgatgcctgttatttttatgtaattcttttactagctaatggtag
PH207_w2_flanking(1>32227)  → aatattatgaatggtataacccaaatatatatgtgatgcctgttatttttatgtaattcttttactagctaatggtag

                              22780     22790     22800     22810     22820     22830     22840     22850
                              catagtttttgatattatcataaaggacccaagcatgaaggcacgaaggacgtaaacttggtgatggagatcactgtt
PH207_w2_flanking(1>32227)  → catagtttttgatattatcataaaggacccaagcatgaaggcacgaaggacgtaaacttggtgatggagatcactgtt

                              22860     22870     22880     22890     22900     22910     22920     22930
                              gagtttgtgatccaaattctgaagaaggcggccaagttggcgagcgaagcggagNNNNNNNNNNNNNNNNNNNNNNNN
PH207_w2_flanking(1>32227)  → gagtttgtgatccaaattctgaagaaggcggccaagttggcgagcgaagcggagnnnnnnnnnnnnnnnnnnnnnnnn
```

22940 22950 22960 22970 22980 22990 23000 23010
NNNNNNNNNNNNNNNNNNNNNNNNNNNNNNNNNNNNNNNNNNNNNNNNNNNNNNNNNNNNNNNNNNNNNNNNNNNNNN
PH207_w2_flanking(1>32227) → nnnnnnnnnnnnnnnnnnnnnnnnnnnnnnnnnnnnnnnnnnnnnnnnnnnnnnnnnnnnnnnnnnnnnnnnnnnnnn

23020 23030 23040 23050 23060 23070 23080
NNNNNNNNNNNNNNNNNNNNNNNNNNNNNNNNNNNNNNNNNNNNNNNNNNNNNNNNNNNNNNNNNNNNNNNNNNNNNN
PH207_w2_flanking(1>32227) → nnnnnnnnnnnnnnnnnnnnnnnnnnnnnnnnnnnnnnnnnnnnnnnnnnnnnnnnnnnnnnnnnnnnnnnnnnnnnnn

23090 23100 23110 23120 23130 23140 23150 23160
NNNNNNNNNNNNNNNNNNNNNNNNNNNNNNNNNNNNNNNNNNNNNNNNNNNNNNNNNNNNNNNNNNNNNNNNNNNNNN
PH207_w2_flanking(1>32227) → nnnnnnnnnnnnnnnnnnnnnnnnnnnnnnnnnnnnnnnnnnnnnnnnnnnnnnnnnnnnnnnnnnnnnnnnnnnnnn

23170 23180 23190 23200 23210 23220 23230 23240
NNNNNNNNNNNNNNNNNNNNNNNNNNNNNNNNNNNNNNNNNNNNNNNNNNNNNNNNNNNNNNNNNNNNNNNNNNNNNN
PH207_w2_flanking(1>32227) → nnnnnnnnnnnnnnnnnnnnnnnnnnnnnnnnnnnnnnnnnnnnnnnnnnnnnnnnnnnnnnnnnnnnnnnnnnnnnn

23250 23260 23270 23280 23290 23300 23310 23320
NNNNNNNNNNNNNNNNNNNNNNNNNNNNNNNNNNNNNNNNNNNNNNNNNNNNNNNNNNNNNNNNNNNNNNNNNNNNNN
PH207_w2_flanking(1>32227) → nnnnnnnnnnnnnnnnnnnnnnnnnnnnnnnnnnnnnnnnnnnnnnnnnnnnnnnnnnnnnnnnnnnnnnnnnnnnnn

23330 23340 23350 23360 23370 23380 23390 23400
NNNNNNNNNNNNNNNNNNNNNNNNNNNNNNNNNNNNNNNNNNNNNNNNNNNNNNNNNNNNNNNNNNNNNNNNNNNNNN
PH207_w2_flanking(1>32227) → nnnnnnnnnnnnnnnnnnnnnnnnnnnnnnnnnnnnnnnnnnnnnnnnnnnnnnnnnnnnnnnnnnnnnnnnnnnnnn

23410 23420 23430 23440 23450 23460 23470
NNNNNNNNtccacgttaaatccgtgtctactctgtgattgatcctatttgtgtcgcatttatttataacaatcaccat
PH207_w2_flanking(1>32227) → nnnnnnnntccacgttaaatccgtgtctactctgtgattgatcctatttgtgtcgcatttatttataacaatcaccat

23480 23490 23500 23510 23520 23530 23540 23550
gatatatggccatactatcacatatgtactatgattagttttatttttgtgtaatactattcatccaaacattaaata
PH207_w2_flanking(1>32227) → gatatatggccatactatcacatatgtactatgattagttttatttttgtgtaatactattcatccaaacattaaata

23560 23570 23580 23590 23600 23610 23620 23630
tgatataatagcttctaaaaaatgaagagttcactttgatacccttctaacctattgcgcctataatgattctaacca
PH207_w2_flanking(1>32227) → tgatataatagcttctaaaaaatgaagagttcactttgatacccttctaacctattgcgcctataatgattctaacca

23640 23650 23660 23670 23680 23690 23700 23710
tcattttggtagggctgccaaagcaaagtgtcattggttatctcctaataggatggatatcaaacatctacatatata
PH207_w2_flanking(1>32227) → tcattttggtagggctgccaaagcaaagtgtcattggttatctcctaataggatggatatcaaacatctacatatata

23720 23730 23740 23750 23760 23770 23780 23790
atgttgattttcttaacgaggatatgcaatcggatctggggattaagtatgatgttgggagccacaacattggttacc
PH207_w2_flanking(1>32227) → atgttgattttcttaacgaggatatgcaatcggatctggggattaagtatgatgttgggagccacaacattggttacc

23800 23810 23820 23830 23840 23850 23860
atccaacaaataaaagtcatatataataagattagcaagcattgcttatctagatggacaagtgtgacgaactgcaag
PH207_w2_flanking(1>32227) → atccaacaaataaaagtcatatataataagattagcaagcattgcttatctagatggacaagtgtgacgaactgcaag

23870 23880 23890 23900 23910 23920 23930 23940
aaggctgcaggctacttctctacaaccgctgcatgcaggacccccacccccacacacacacaaagaagaaaaagaaaa
PH207_w2_flanking(1>32227) → aaggctgcaggctacttctctacaaccgctgcatgcaggacccccacccccacacacacacaaagaagaaaaagaaaa

23950 23960 23970 23980 23990 24000 24010 24020

ggaggcaccatgaagatctgagtcgcataattgaggcatcgtcactgtgaagtgtggtaccataaatctgaacatgca

PH207_w2_flanking(1>32227) → ggaggcaccatgaagatctgagtcgcataattgaggcatcgtcactgtgaagtgtggtaccataaatctgaacatgca

24030 24040 24050 24060 24070 24080 24090 24100

tgtccttcatgcatcaacgcaagcgtaagcccttgcgtgctgcagcttaattaacgtctcaaacatcagaaacatcta

PH207_w2_flanking(1>32227) → tgtccttcatgcatcaacgcaagcgtaagcccttgcgtgctgcagcttaattaacgtctcaaacatcagaaacatcta

24110 24120 24130 24140 24150 24160 24170 24180

ctttgtcttatttgatgtccgcatcggagaagcctacatgaattacatcaggcattcaggcttcagagccatggatta

PH207_w2_flanking(1>32227) → ctttgtcttatttgatgtccgcatcggagaagcctacatgaattacatcaggcattcaggcttcagagccatggatta

24190 24200 24210 24220 24230 24240 24250

tttaaaggtgcatctacgacggccgcagatctctgatgggcaaaatatttatgtaggcgcagaggctatgagcgagcc

PH207_w2_flanking(1>32227) → tttaaaggtgcatctacgacggccgcagatctctgatgggcaaaatatttatgtaggcgcagaggctatgagcgagcc

24260 24270 24280 24290 24300 24310 24320 24330

aaggtagacaaccatgcgcggcgagtctactcctagtgatgtacgcctccattgccaccgctcccttctctcgcgtgg

PH207_w2_flanking(1>32227) → aaggtagacaaccatgcgcggcgagtctactcctagtgatgtacgcctccattgccaccgctcccttctctcgcgtgg

24340 24350 24360 24370 24380 24390 24400 24410

tccggcggccgcggccgcggcctcctcaccgcgaccgcccatacgcgggtgatcacctcatgcatgtagaaaccctgc

PH207_w2_flanking(1>32227) → tccggcggccgcggccgcggcctcctcaccgcgaccgcccatacgcgggtgatcacctcatgcatgtagaaaccctgc

24420 24430 24440 24450 24460 24470 24480 24490

ggcggctgcatgcacgcattttcatttcacttgagtcagtcagtgaacaactgaacatccatcggccggttattacca

PH207_w2_flanking(1>32227) → ggcggctgcatgcacgcattttcatttcacttgagtcagtcagtgaacaactgaacatccatcggccggttattacca

24500 24510 24520 24530 24540 24550 24560 24570

atcggcaggctagcttttggcgaaatcaagcggcgatcaaccacttacgttagccttcgccatgtcgacgccggcgtc

PH207_w2_flanking(1>32227) → atcggcaggctagcttttggcgaaatcaagcggcgatcaaccacttacgttagccttcgccatgtcgacgccggcgtc

24580 24590 24600 24610 24620 24630 24640

ttcttcatcctgggacgagctcgccgccgccaccgtggcggcttcagctgcagttgcagcaacggcagccgccggcgg

PH207_w2_flanking(1>32227) → ttcttcatcctgggacgagctcgccgccgccaccgtggcggcttcagctgcagttgcagcaacggcagccgccggcgg

24650 24660 24670 24680 24690 24700 24710 24720

ctgctgatcgccgccgcagccgtgcaggcccaggaagccggcgcaggcgcgcaacaacgagcacgggtgcccgaaggg

PH207_w2_flanking(1>32227) → ctgctgatcgccgccgcagccgtgcaggcccaggaagccggcgcaggcgcgcaacaacgagcacgggtgcccgaaggg

24730 24740 24750 24760 24770 24780 24790 24800

tggcgccgccgaggcttctccagagcccacgtcgtcctcgggcagcaggcccgccgattcgtccgctttatacccgtg

PH207_w2_flanking(1>32227) → tggcgccgccgaggcttctccagagcccacgtcgtcctcgggcagcaggcccgccgattcgtccgctttatacccgtg

24810 24820 24830 24840 24850 24860 24870 24880

cccgtcctcgcgctggtccgtggattcctcagacgtgtcctctccctccgccgacgccgtctcctcgcgctgctccgc

PH207_w2_flanking(1>32227) → cccgtcctcgcgctggtccgtggattcctcagacgtgtcctctccctccgccgacgccgtctcctcgcgctgctccgc

24890 24900 24910 24920 24930 24940 24950 24960

cgccaaagccgaatcttgagacttgttctcctccttttccccgtgctcatccattgcctctgttgccgtgtccgctcc

PH207_w2_flanking(1>32227) → cgccaaagccgaatcttgagacttgttctcctccttttccccgtgctcatccattgcctctgttgccgtgtccgctcc

```
                                  24970    24980    24990    25000    25010    25020    25030
                                  gctctagacaccgggagcttctggacgtcggagctttgttgagatttgtatacgatcagttctctaccaacgacatgt
PH207_w2_flanking(1>32227)    →   gctctagacaccgggagcttctggacgtcggagctttgttgagatttgtatacgatcagttctctaccaacgacatgt

                                  25040    25050    25060    25070    25080    25090    25100    25110
                                  cgagacagactccaactagagatattctcattagagatagagatggcagatttgcagttcaaacgcaaaatctctaca
PH207_w2_flanking(1>32227)    →   cgagacagactccaactagagatattctcattagagatagagatggcagatttgcagttcaaacgcaaaatctctaca

                                  25120    25130    25140    25150    25160    25170    25180    25190
                                  gcactggagagggcggtatgcttttaatctctatatctctatatCTaATCTCTGGCTTTTGTCCTCTCAACATGTGG
PH207_w2_flanking(1>32227)    →   gcactggagagggcggtatgcttttaatctctatatctctatatctaatctctggcttttgtcctctcaacatgtgg
1754_8_f.598(1>491)           →                                               CT-ATCTCTGGCTTTTGTCCTCTCAACATGTGG

                                  25200    25210    25220    25230    25240    25250    25260    25270
                                  GTTTTTTTTTGTTTATTCAGTGCTAGTTTGCAAAAATTAGACTGTTTGGGAGGGCTTCGTTTCAAAAGTTTTTCAGCT
PH207_w2_flanking(1>32227)    →   gtttttttttgtttattcagtgctagtttgcaaaaattagactgtttgggagggcttcgtttcaaaagtttttcagct
1754_8_f.598(1>491)           →   GTTTTTTTTTGTTTATTCAGTGCTAGTTTGCAAAAATTAGACTGTTTGGGAGGGCTTCGTTTCAAAAGTTTTTCAGCT

                                  25280    25290    25300    25310    25320    25330    25340    25350
                                  TCACCATAAAACAGATTCAACAGATTGATAAGATATGATTTAATAGTGTTTCATTTGCATATAGACTCTAGCTTATGT
PH207_w2_flanking(1>32227)    →   tcaccataaaacagattcaacagattgataagatatgatttaatagtgtttcatttgcatatagactctagcttatgt
1754_8_f.598(1>491)           →   TCACCATAAAACAGATTCAACAGATTGATAAGATATGATTTAATAGTGTTTCATTTGCATATAGACTCTAGCTTATGT

                                  25360    25370    25380    25390    25400    25410    25420
                                  AAAATAATTAATTTGTTCGACTTTTCTTAATTATCTATGATAATTAGCGGGTGAAGAAAGAGACATGAATCAGTACGT
PH207_w2_flanking(1>32227)    →   aaaataattaatttgttcgacttttcttaattatctatgataattagcgggtgaagaaagagacatgaatcagtacgt
1754_8_f.598(1>491)           →   AAAATAATTAATTTGTTCGACTTTTCTTAATTATCTATGATAATTAGCGGGTGAAGAAAGAGACATGAATCAGTACGT

                                  25430    25440    25450    25460    25470    25480    25490    25500
                                  CATATAACCAATGATATGGTGATTAATTTTTGTGCAACTTCATGAGGAGGTAATGACATGGTGATTAATTTTTGTGCA
PH207_w2_flanking(1>32227)    →   catataaccaatgatatggtgattaatttttgtgcaacttcatgaggaggtaatgacatggtgattaatttttgtgca
1754_8_f.598(1>491)           →   CATATAACCAATGATATGGTGATTAATTTTTGTGCAACTTCATGAGGAGGTAATGACATGGTGATTAATTTTTGTGCA

                                  25510    25520    25530    25540    25550    25560    25570    25580
                                  ACTTCATGAGGAGGTATGTTTTTAAGCAGCCTTTGATAAGTTTGGAAAATTTTAATGAAATTGACCTCTAATTTCAGT
PH207_w2_flanking(1>32227)    →   acttcatgaggaggtatgtttttaagcagcctttgataagtttggaaaattttaatgaaattgacctctaatttcagt
1754_8_f.598(1>491)           →   ACTTCATGAGGAGGTATGTTTTTAAGCAGCCTTTGATAAGTTTGGAAAATTTTAATGAAATTGACCTCTAATTTCAGT

                                  25590    25600    25610    25620    25630    25640    25650    25660
                                  TTTTTAAAAACTAGAGCTCGTGGAGCTAGACCTGTTTGGATAGAAGGAGCTGAAATAGATCTGAAATTGttaaagtaa
PH207_w2_flanking(1>32227)    →   tttttaaaaactagagctcgtggagctagacctgtttggatagaaggagctgaaatagatctgaaattgttaaagtaa
1754_8_f.598(1>491)           →   TTTTTAAAAACTAGAGCTCGTGGAGCTAGACCTGTTTGGATAGAAGGAGCTGAAATAGATCTGAAATTG

                                  25670    25680    25690    25700    25710    25720    25730    25740
                                  agctctgccaaataggatcttaaatcccctccagaattctcggggattgagctaatttcccactcaatttttggaatc
PH207_w2_flanking(1>32227)    →   agctctgccaaataggatcttaaatcccctccagaattctcggggattgagctaatttcccactcaatttttggaatc

                                  25750    25760    25770    25780    25790    25800    25810
                                  cctaagggtatttaaatttcccactctatattTTTTAAAGGTTTTCAACAAGATAGTAATGTTTCATTAAATTAAATT
PH207_w2_flanking(1>32227)    →   cctaagggtatttaaatttcccactctatattttttaaaggttttcaacaagatagtaatgtttcattaaattaaatt
1754_8_r.608(1>180)           ←                                   TTTTAAAGGTTTTCAACAAGATAGTAATGTTTCATTAAATTAAATT

                                  25820    25830    25840    25850    25860    25870    25880    25890
                                  TTCCAAAGCAGCTGAAAAATATTTTCGGGAACATCTAACATAAGAAACAAACGTAGAAAATTAAAATTTAGATTAGTC
PH207_w2_flanking(1>32227)    →   ttccaaagcagctgaaaaatattttcgggaacatctaacataagaaacaaacgtagaaaattaaaatttagattagtc
1754_8_r.608(1>180)           ←   TTCCAAAGCAGCTGAAAAATATTTTCGGGAACATCTAACATAAGAAACAAACGTAGAAAATTAAAATTTAGATTAGTC
```

```
                                   25900     25910     25920     25930     25940     25950     25960     25970
                                   TACCACCATAATAAATAAGTAAACTATTTGCTCCAATAAATGCATATAAATATAGTctttcttctgtggggaattggg
PH207_w2_flanking(1>32227)   →     taccaccataataaataagtaaactatttgctccaataaatgcatataaatatagtctttcttctgtggggaattggg
1754_8_r.608(1>180)          ←     TACCACCATAATAAATAAGTAAACTATTTGCTCCAATAAATGCATATAAATATAGT

                                     25980     25990     26000     26010     26020     26030     26040     26050
                                   acacattgctgttattgacaacacgctgccatggctctaattcacagacacagttaacgatcacagtgagcaggggaa
PH207_w2_flanking(1>32227)   →     acacattgctgttattgacaacacgctgccatggctctaattcacagacacagttaacgatcacagtgagcaggggaa

                                       26060     26070     26080     26090     26100     26110     26120     26130
                                   GGGCTCACTGAGTGAAAGGATAAAAAAATGGAATGAGATTGGAGACGCTATATATATAGATAATGGTGGTAGAAAAAT
PH207_w2_flanking(1>32227)   →     gggctcactgagtgaaaggataaaaaaatggaatgagattggagacgctatatatatagataatggtggtagaaaaat
1754_599_1.599(1>1052)       →     GGGCTCACTGAGTGAAAGGATAAAAAAATGGAATGAGATTGGAGACGCTATATATATAGATAATGGTGGTAGAAAAAT
WT_599_1.599(1>1024)         →     GGGCTCACTGAGTGAAAGGATAAAAAAATGGAATGAGATTGGAGACGCTATATATATAGATAATGGTGGTAGAAAAAT

                                         26140     26150     26160     26170     26180     26190     26200
                                   TGCTTCCTGGCCTCAGTTTTGTGTCTTCTCCACCTTGGCCTTGATCTTCTGCTCGATGATCGCCTTCTCCATGTCGAG
PH207_w2_flanking(1>32227)   →     tgcttcctggcctcagttttgtgtcttctccaccttggccttgatcttctgctcgatgatcgccttctccatgtcgag
1754_599_1.599(1>1052)       →     TGCTTCCTGGCCTCAGTTTTGTGTCTTCTCCACCTTGGCCTTGATCTTCTGCTCGATGATCGCCTTCTCCATGTCGAG
WT_599_1.599(1>1024)         →     TGCTTCCTGGCCTCAGTTTTGTGTCTTCTCCACCTTGGCCTTGATCTTCTGCTCGATGATCGCCTTCTCCATGTCGAG

                                 26210     26220     26230     26240     26250     26260     26270     26280
                                   GCTGAACATCCGGTTGAGGGCGTGCATGTTCTCGAGCGTCTCGTCAAGGGTGCGGCTGTCGCTGCCGTCGCACCTCAG
PH207_w2_flanking(1>32227)   →     gctgaacatccggttgagggcgtgcatgttctcgagcgtctcgtcaagggtgcggctgtcgctgccgtcgcacctcag
1754_599_1.599(1>1052)       →     GCTGAACATCCGGTTGAGGGCGTGCATGTTCTCGAGCGTCTCGTCAAGGGTGCGGCTGTCGCTGCCGTCGCACCTCAG
WT_599_1.599(1>1024)         →     GCTGAACATCCGGTTGAGGGCGTGCATGTTCTCGAGCGTCTCGTCAAGGGTGCGGCTGTCGCTGCCGTCGCACCTCAG

                                   26290     26300     26310     26320     26330     26340     26350     26360
                                   GTGCTGCAGCGGGCCATGGAGGAGCTTGTTAACGATGCCGGTGCTCAGCTCCTCGATGGCTCTCCTCATCTTCTTGGT
PH207_w2_flanking(1>32227)   →     gtgctgcagcgggccatggaggagcttgttaacgatgccggtgctcagctcctcgatggctctcctcatcttcttggt
1754_599_1.599(1>1052)       →     GTGCTGCAGCGGGCCATGGAGGAGCTTGTTAACGATGCCGGTGCTCAGCTCCTCGATGGCTCTCCTCATCTTCTTGGT
WT_599_1.599(1>1024)         →     GTGCTGCAGCGGGCCATGGAGGAGCTTGTTAACGATGCCGGTGCTCAGCTCCTCGATGGCTCTCCTCATCTTCTTGGT
1754_609_2.609(1>1049)       ←        CTGCAGCGGGCCATGGAGGAGCTTGTTAACGATGCCGGTGCTCAGCTCCTCGATGGCTCTCCTCATCTTCTTGGT
WT_609_2.609(1>1049)         ←        CTGCAGCGGGCCATGGAGGAGCTTGTTAACGATGCCGGTGCTCAGCTCCTCGATGGCTCTCCTCATCTTCTTGGT

                                     26370     26380     26390     26400     26410     26420     26430     26440
                                   GAGGGCGTCCTCACCGACTTTCTGCAGGCACTTCTCGAGCTCCGAGGCCCTGATCCTGTCCGCGTACGACCTCAGCTT
PH207_w2_flanking(1>32227)   →     gagggcgtcctcaccgactttctgcaggcacttctcgagctccgaggccctgatcctgtccgcgtacgacctcagctt
1754_599_1.599(1>1052)       →     GAGGGCGTCCTCACCGACTTTCTGCAGGCACTTCTCGAGCTCCGAGGCCCTGATCCTGTCCGCGTACGACCTCAGCTT
WT_599_1.599(1>1024)         →     GAGGGCGTCCTCACCGACTTTCTGCAGGCACTTCTCGAGCTCCGAGGCCCTGATCCTGTCCGCGTACGACCTCAGCTT
1754_609_2.609(1>1049)       ←     GAGGGCGTCCTCACCGACTTTCTGCAGGCACTTCTCGAGCTCCGAGGCCCTGATCCTGTCCGCGTACGACCTCAGCTT
WT_609_2.609(1>1049)         ←     GAGGGCGTCCTCACCGACTTTCTGCAGGCACTTCTCGAGCTCCGAGGCCCTGATCCTGTCCGCGTACGACCTCAGCTT

                                       26450     26460     26470     26480     26490     26500     26510     26520
                                   CTTGATGGTCGGAACGGTCTCCAGCGAGTCCCTCCATGCCTCGAACCGTCTCAGTTCTTCTGTGATGATTGTCTGCGC
PH207_w2_flanking(1>32227)   →     cttgatggtcggaacggtctccagcgagtccctccatgcctcgaaccgtctcagttcttctgtgatgattgtctgcgc
1754_599_1.599(1>1052)       →     CTTGATGGTCGGAACGGTCTCCAGCGAGTCCCTCCATGCCTCGAACCGTCTCAGTTCTTCTGTGATGATTGTCTGCGC
WT_599_1.599(1>1024)         →     CTTGATGGTCGGAACGGTCTCCAGCGAGTCCCTCCATGCCTCGAACCGTCTCAGTTCTTCTGTGATGATTGTCTGCGC
1754_609_2.609(1>1049)       ←     CTTGATGGTCGGAACGGTCTCCAGCGAGTCCCTCCATGCCTCGAACCGTCTCAGTTCTTCTGTGATGATTGTCTGCGC
WT_609_2.609(1>1049)         ←     CTTGATGGTCGGAACGGTCTCCAGCGAGTCCCTCCATGCCTCGAACCGTCTCAGTTCTTCTGTGATGATTGTCTGCGC

                                         26530     26540     26550     26560     26570     26580     26590
                                   CTCCATTGCTTTCCTGAGCCGGTCCTCCTTGTTGGCTTCCACCACCTCTTTCAAGTCGTCGACATTGTACACTCGTGC
PH207_w2_flanking(1>32227)   →     ctccattgctttcctgagccggtcctccttgttggcttccaccacctctttcaagtcgtcgacattgtacactcgtgc
1754_599_1.599(1>1052)       →     CTCCATTGCTTTCCTGAGCCGGTCCTCCTTGTTGGCTTCCACCACCTCTTTCAAGTCGTCGACATTGTACACTCGTGC
WT_599_1.599(1>1024)         →     CTCCATTGCTTTCCTGAGCCGGTCCTCCTTGTTGGCTTCCACCACCTCTTTCAAGTCGTCGACATTGTACACTCGTGC
1754_609_2.609(1>1049)       ←     CTCCATTGCTTTCCTGAGCCGGTCCTCCTTGTTGGCTTCCACCACCTCTTTCAAGTCGTCGACATTGTACACTCGTGC
WT_609_2.609(1>1049)         ←     CTCCATTGCTTTCCTGAGCCGGTCCTCCTTGTTGGCTTCCACCACCTCTTTCAAGTCGTCGACATTGTACACTCGTGC
```

```
                              26600     26610     26620     26630     26640     26650     26660     26670
                              AGCGCCAACTTCAGACACACATGCGCTGACATTCCTGGGGACAGATATGTCGACAAACAGGCGAACACCTCCCATAGT
PH207_w2_flanking(1>32227)  → agcgccaacttcagacacacatgcgctgacattcctggggacagatatgtcgacaaacaggcgaacacctcccatagt
1754_599_1.599(1>1052)      → AGCGCCAACTTCAGACACACATGCGCTGACATTCCTGGGGACAGATATGTCGACAAACAGGCGAACACCTCCCATAGT
WT_599_1.599(1>1024)        → AGCGCCAACTTCAGACACACATGCGCTGACATTCCTGGGGACAGATATGTCGACAAACAGGCGAACACCTCCCATAGT
1754_609_2.609(1>1049)      ← AGCGCCAACTTCAGACACACATGCGCTGACATTCCTGGGGACAGATATGTCGACAAACAGGCGAACACCTCCCATAGT
WT_609_2.609(1>1049)        ← AGCGCCAACTTCAGACACACATGCGCTGACATTCCTGGGGACAGATATGTCGACAAACAGGCGAACACCTCCCATAGT

                              26680     26690     26700     26710     26720     26730     26740     26750
                              ATCAGAGACAGGGGGGAGTGCCTCTGCGTGTTCTTTTGCGAACAATGAAGTTTCAGATGCGGTGCTGGTGAACACGAC
PH207_w2_flanking(1>32227)  → atcagagacaggggggagtgcctctgcgtgttcttttgcgaacaatgaagtttcagatgcggtgctggtgaacacgac
1754_599_1.599(1>1052)      → ATCAGAGACAGGGGGGAGTGCCTCTGCGTGTTCTTTTGCGAACAATGAAGTTTCAGATGCGGTGCTGGTGAACACGAC
WT_599_1.599(1>1024)        → ATCAGAGACAGGGGGGAGTGCCTCTGCGTGTTCTTTTGCGAACAATGAAGTTTCAGATGCGGTGCTGGTGAACACGAC
1754_609_2.609(1>1049)      ← ATCAGAGACAGGGGGGAGTGCCTCTGCGTGTTCTTTTGCGAACAATGAAGTTTCAGATGCGGTGCTGGTGAACACGAC
WT_609_2.609(1>1049)        ← ATCAGAGACAGGGGGGAGTGCCTCTGCGTGTTCTTTTGCGAACAATGAAGTTTCAGATGCGGTGCTGGTGAACACGAC

                              26760     26770     26780     26790     26800     26810     26820     26830
                              ATCAGCTTCAGCAGCAGCTTGATACATGTCTGAGAGAGGCCTGTACACGATCTCTATATCTTTCATCTCCTCACGAAT
PH207_w2_flanking(1>32227)  → atcagcttcagcagcagcttgatacatgtctgagagaggcctgtacacgatctctatatctttcatctcctcacgaat
1754_599_1.599(1>1052)      → ATCAGCTTCAGCAGCAGCTTGATACATGTCTGAGAGAGGCCTGTACACGATCTCTATATCTTTCATCTCCTCACGAAT
WT_599_1.599(1>1024)        → ATCAGCTTCAGCAGCAGCTTGATACATGTCTGAGAGAGGCCTGTACACGATCTCTATATCTTTCATCTCCTCACGAAT
1754_609_2.609(1>1049)      ← ATCAGCTTCAGCAGCAGCTTGATACATGTCTGAGAGAGGCCTGTACACGATCTCTATATCTTTCATCTCCTCACGAAT
WT_609_2.609(1>1049)        ← ATCAGCTTCAGCAGCAGCTTGATACATGTCTGAGAGAGGCCTGTACACGATCTCTATATCTTTCATCTCCTCACGAAT

                              26840     26850     26860     26870     26880     26890     26900     26910
                              AGCATCCACCCTTTCCACCGAGCGGTTCACCACAACAACCTTCTTGCATCCTTTGGCAACCAGATGTTTGATCACTAG
PH207_w2_flanking(1>32227)  → agcatccaccctttccaccgagcggttcaccacaacaaccttcttgcatcctttggcaaccagatgtttgatcactag
1754_599_1.599(1>1052)      → AGCATCCACCCTTTCCACCGAGCGGTTCACCACAACAACCTTCTTGCATCCTTTGGCAACCAGATGTTTGATCACTAG
WT_599_1.599(1>1024)        → AGCATCCACCCTTTCCACCGAGCGGTTCACCACAACAACCTTCTTGCATCCTTTGGCAACCAGATGTTTGATCACTAG
1754_609_2.609(1>1049)      ← AGCATCCACCCTTTCCACCGAGCGGTTCACCACAACAACCTTCTTGCATCCTTTGGCAACCAGATGTTTGATCACTAG
WT_609_2.609(1>1049)        ← AGCATCCACCCTTTCCACCGAGCGGTTCACCACAACAACCTTCTTGCATCCTTTGGCAACCAGATGTTTGATCACTAG

                              26920     26930     26940     26950     26960     26970     26980
                              CTTTCCCATTTTCCCAGCACCAATCAGAAGCATCCTAGCTGACAGTGCTTCAGACTTCGGAAGCTTCATCAGGGCCAG
PH207_w2_flanking(1>32227)  → ctttcccattttcccagcaccaatcagaagcatcctagctgacagtgcttcagacttcggaagcttcatcagggccag
1754_599_1.599(1>1052)      → CTTTCCCATTTTCCCAGCACCAATCAGAAGCATCCTAGCTGACAGTGCTTCAGACTTCGGAAGCTTCATCAGGGCCAG
WT_599_1.599(1>1024)        → CTTTCCCATTTTCCCAGCACCAATCAGAAGCATCCTAGCTGACAGTGCTTCAGACTTCGGAAGCTTCATCAGGGCCAG
1754_609_2.609(1>1049)      ← CTTTCCCATTTTCCCAGCACCAATCAGAAGCATCCTAGCTGACAGTGCTTCAGACTTCGGAAGCTTCATCAGGGCCAG
WT_609_2.609(1>1049)        ← CTTTCCCATTTTCCCAGCACCAATCAGAAGCATCCTAGCTGACAGTGCTTCAGACTTCGGAAGCTTCATCAGGGCCAG

                              26990     27000     27010     27020     27030     27040     27050     27060
                              TTCAACCGCCGCTGAACTGACAGAAACAGCACCAGAT-G-ATATGTTGGTCTCGCTGCGGACACGCTTTCCAGCAGTG
PH207_w2_flanking(1>32227)  → ttcaaccgccgctgaactgacagaaacagcaccagat-g-atatgttggtctcgctgcggacacgctttccagcagtg
1754_599_1.599(1>1052)      → TTCAACCGCCGCTGAACTGACAGAAACAGCACCAGAT-GGATATGTTGGTCTCGCTGCGGACACGCTTTCCAGCAGTG
WT_599_1.599(1>1024)        → TTCAACCGCCGCTGAACTGACAGAAACAGCACCAGATTG-ATATGTTGGTCTCGCTGCGGACACGCTTTCCAGCAGTG
1754_609_2.609(1>1049)      ← TTCAACCGCCGCTGAACTGACAGAAACAGCACCAGAT-G-ATATGTTGGTCTCGCTGCGGACACGCTTTCCAGCAGTG
WT_609_2.609(1>1049)        ← TTCAACCGCCGCTGAACTGACAGAAACAGCACCAGAT-G-ATATGTTGGTCTCGCTGCGGACACGCTTTCCAGCAGTG

                              27070     27080     27090     27100     27110     27120     27130     27140
                              ATTGCATCCTTGAACATCCTATCAATGTTCTTTCCCAAGCCTCCGCTGTTCTGTCCACTCCTCACAACTTGTTTAACC
PH207_w2_flanking(1>32227)  → attgcatccttgaacatcctatcaatgttctttcccaagcctccgctgttctgtccactcctcacaacttgtttaacc
1754_599_1.599(1>1052)      → ATTGCATCCTTGAACATCCAATCAATGTTCTTTCCCAAG
WT_599_1.599(1>1024)        → ATTGCATCCTT
1754_609_2.609(1>1049)      ← ATTGCATCCTTGAACATCCTATCAATGTTCTTTCCCAAGCCTCCGCTGTTCTGTCCACTCCTCACAACTTGTTTAACC
WT_609_2.609(1>1049)        ← ATTGCATCCTTGAACATCCTATCAATGTTCTTTCCCAAGCCTCCGCTGTTCTGTCCACTCCTCACAACTTGTTTAACC

                              27150     27160     27170     27180     27190     27200     27210     27220
                              TGAGCAAGGATTTGTCCTTCACCGAGAACCAAAGAATCAGGCCAGCTGACACCTCAAACAGATGGCGTGTGGCATCA
PH207_w2_flanking(1>32227)  → tgagcaaggatttgtccttcaccgagaaccaaagaatcaggccagctgacacctcaaacagatggcgtgtggcatca
1754_609_2.609(1>1049)      ← TGAGCAAGGATTTGTCCTTCACCGAGAACCAAAGAATCAGGCCAGCTGACACCTCAAACAGATGGCGTGTGGCATCA
WT_609_2.609(1>1049)        ← TGAGCAAGGATTTGTCCTTCACCGAGAACCAAAGAATCAGGCCAGCTGACACCTCAAACAGATGGCGTGTGGCATCA
WT_w2_9_r.609(1>180)        ←                        GAGAACCAAAGAATCAGGCCAGCTGACACCTCAAACAGATGGCGTGTGGCATCA
```

```
                           27230     27240     27250     27260     27270     27280     27290     27300
                           CTGCTTCGCAAGATGAACAGGTGCTCCCTAAGCTCGGAAGCGGGAATACCACTTTTCTGCAAAATCACAAAAAATACA
PH207_w2_flanking(1>32227) ctgcttcgcaagatgaacaggtgctccctaagctcggaagcgggaataccacttttctgcaaaatcacaaaaaataca
1754_609_2.609(1>1049)     CTGCTTCGCAAGATGAACAGGTGCTCCCTAAGCTCGGAAGCGGGAATACCACTTTTCTGCAAAATCACAAAAAATACA
WT_609_2.609(1>1049)       CTGCTTCGCAAGATGAACAGGTGCTCCCTAAGCTCGGAAGCGGGAATACCACTTTTCTGCAAAATCACAAAAAATACA
WT_w2_9_r.609(1>180)       CTGCTTTGCAAGATGAACAGGTGCTCCCTAAGCTTGGAAGCGGGAATACCACTTTTCTGCAAAATCACAAAAAATACA
```

```
                              27310     27320     27330     27340     27350     27360     27370
                           CATATTAGGGAACCTTTCCTAAACTGCGTCTT-GAGCAACCAAAAAGCtgggcaaaaagactaccacagactgaacag
PH207_w2_flanking(1>32227) catattagggaacctttcctaaactgcgtctttgagcaaccaaaaagctgggcaaaaagactaccacagactgaacag
1754_609_2.609(1>1049)     CATATTAGGGAACCTTTCCTAAACTGCGTCTT-GAGCAACC
WT_609_2.609(1>1049)       CATATTAGGGAACCTTTCCTAAACTGCGTCTT-GAGCAACC
WT_w2_9_r.609(1>180)       CATATTAGGGAACCTTTCCTAAACTGCGTCTT-GAGCAACCAAAAAGC
```

```
                         27380     27390     27400     27410     27420     27430     27440     27450
                           agaaggttgatCAAACAGTATTCACCTTCGACATCCAGTCCACTACTTCTCT-GATACCACGGTTCCAT-GATAGCGC
PH207_w2_flanking(1>32227) agaaggttgatcaaacagtattcaccttcgacatccagtccactacttctct-gataccacggttccat-gatagcgc
WT_610_2.610(1>891)                   CAAACAGTATTCACCTTCGACATCCAGTCCACTACTTCTCT-GATACCACGGTTCCAT-GATAGCGC
WT_w2_10_f.600(1>287)                                                   CTTCTCT-GATACCACGGTTCCAT-GATAGCGC
1754_10_f.600(1>326)                                                    CTTCTCTAGATACCACGGTTCCATAGATAGCGC
1754_600_1.600(1>898)                                                        CT-GATACCACGGTTCCAT-GATAGCGC
1754_610_2.610(1>847)                                                            ATACCACGGTTCCAT-GATAGCGC
WT_600_1.600(1>881)                                                                     CGGTTCCAT-GATAGCGC
```

```
                            27460     27470     27480     27490     27500     27510     27520     27530
                           CACCACATAAATTTCCATTCTATTACAGGTACTAAGAACAGCAGCCTCTT-CAATATGGTTCAGGCTAGTAAGTTCTT
PH207_w2_flanking(1>32227) caccacataaatttccattctattacaggtactaagaacagcagcctctt-caatatggttcaggctagtaagttctt
WT_610_2.610(1>891)        CACCACATAAATTTCCATTCTATTACAGGTACTAAGAACAGCAGCCTCTT-CAATATGGTTCAGGCTAGTAAGTTCTT
WT_w2_10_f.600(1>287)      CACCACATAAATTTCCATTCTATTACAGGTACTAAGAACAGCAGCCTCTT-CAATATGGTTCAGGCTAGTAAGTTCTT
1754_10_f.600(1>326)       CACCACATAAATTTCCATTCTATTACAGGTACTAAGAACAGCAGCCTCTCGCAATATGGTTCAGGCTAGTAAGTTCTT
1754_600_1.600(1>898)      CACCACATAAATTTCCATTCTATTACAGGTACTAAGAACAGCAGCCTCTT-CAATATGGTTCAGGCTAGTAAGTTCTT
1754_610_2.610(1>847)      CACCACATAAATTTCCATTCTATTACAGGTACTAAGAACAGCAGCCTCTT-CAATATGGTTCAGGCTAGTAAGTTCTT
WT_600_1.600(1>881)        CACCACATAAATTTCCATTCTATTACAGGTACTAAGAACAGCAGCCTCTT-CAATATGGTTCAGGCTAGTAAGTTCTT
```

```
                             27540     27550     27560     27570     27580     27590     27600     27610
                           GAATAGCTCGGGGCCACAGTTCCTCTGCAACAGCAAGTTTTTCACGCATCTCCACTGGTGCTGTGTGTACACTGAGGC
PH207_w2_flanking(1>32227) gaatagctcggggccacagttcctctgcaacagcaagtttttcacgcatctccactggtgctgtgtgtacactgaggc
WT_610_2.610(1>891)        GAATAGCTCGGGGCCACAGTTCCTCTGCAACAGCAAGTTTTTCACGCATCTCCACTGGTGCTGTGTGTACACTGAGGC
WT_w2_10_f.600(1>287)      GAATAGCTCGGGGCCACAGTTCCTCTGCAACAGCAAGTTTTTCACGCATCTCCACTGGTGCTGTGTGTACACTGAGGC
1754_10_f.600(1>326)       GAATAGCTCGGGGCCACAGTTCCTCTGCAACAGCAAGTTTTTCACGCATCTCCACTGGTGCTGTGTGTACACTGAGGC
1754_600_1.600(1>898)      GAATAGCTCGGGGCCACAGTTCCTCTGCAACAGCAAGTTTTTCACGCATCTCCACTGGTGCTGTGTGTACACTGAGGC
1754_610_2.610(1>847)      GAATAGCTCGGGGCCACAGTTCCTCTGCAACAGCAAGTTTTTCACGCATCTCCACTGGTGCTGTGTGTACACTGAGGC
WT_600_1.600(1>881)        GAATAGCTCGGGGCCACAGTTCCTCTGCAACAGCAAGTTTTTCACGCATCTCCACTGGTGCTGTGTGTACACTGAGGC
```

```
                           27620     27630     27640     27650     27660     27670     27680     27690
                           CTATCACAGCTATGGTACTCCTTTCCTTCATGTACCCTGGTAGAGAATATAATAGTCAAAACATTGTCAAACATGCAC
PH207_w2_flanking(1>32227) ctatcacagctatggtactcctttccttcatgtaccctggtagagaatataatagtcaaaacattgtcaaacatgcac
WT_610_2.610(1>891)        CTATCACAGCTATGGTACTCCTTTCCTTCATGTACCCTGGTAGAGAATATAATAGTCAAAACATTGTCAAACATGCAC
WT_w2_10_f.600(1>287)      CTATCACAGCTATGGTACTCCTTTCCTTCATGTACCCTGGTAGAGAATATAATAGTCAAAACATTGTCAAACATGCAC
1754_10_f.600(1>326)       CTATCACAGCTATGGTACTCCTTTCCTTCATGTACCCTGGTAGAGAATATAATAGTCAAAACATTGTCAAACATGCAC
1754_600_1.600(1>898)      CTATCACAGCTATGGTACTCCTTTCCTTCATGTACCCTGGTAGAGAATATAATAGTCAAAACATTGTCAAACATGCAC
1754_610_2.610(1>847)      CTATCACAGCTATGGTACTCCTTTCCTTCATGTACCCTGGTAGAGAATATAATAGTCAAAACATTGTCAAACATGCAC
WT_600_1.600(1>881)        CTATCACAGCTATGGTACTCCTTTCCTTCATGTACCCTGGTAGAGAATATAATAGTCAAAACATTGTCAAACATGCAC
```

```
                              27700     27710     27720     27730     27740     27750     27760
                           ACTTGCACAGAGACAATTTTGTAAGAAACTGGATACAATTCGATTGGATAAGTTTATCAAGAAAATTCTAAGGGGAG
PH207_w2_flanking(1>32227) acttgcacagagacaattttgtaagaaactggatacaattcgattggataagtttatcaagaaaattctaaggggag
WT_610_2.610(1>891)        ACTTGCACAGAGACAATTTTGTAAGAAACTGGATACAATTCGATTGGATAAGTTTATCAAGAAAATTCTAAGGGGAG
WT_w2_10_f.600(1>287)      ACTTGCACAGAGACAATTTTGTA
1754_10_f.600(1>326)       ACTTGCACAGAGACAATTTTGTAAGAAACTGGATACAATTCAATTGGATAATTTTATCA
1754_600_1.600(1>898)      ACTTGCACAGAGACAATTTTGTAAGAAACTGGATACAATTCGATTGGATAAGTTTATCAAGAAAATTCTAAGGGGAG
1754_610_2.610(1>847)      ACTTGCACAGAGACAATTTTGTAAGAAACTGGATACAATTCGATTGGATAAGTTTATCAAGAAAATTCTAAGGGGAG
WT_600_1.600(1>881)        ACTTGCACAGAGACAATTTTGTAAGAAACTGGATACAATTCGATTGGATAAGTTTATCAAGAAAATTCTAAGGGGAG
```

```
                               27770     27780     27790     27800     27810     27820     27830     27840
                               ATACACTAGATGAGTATGACTGAACAAGATATTCCCAAAAAAGAATCGTGCAAAATGTTTGGCCAATAGCCGATGCCA
PH207_w2_flanking(1>32227)  →  atacactagatgagtatgactgaacaagatattcccaaaaaagaatcgtgcaaaatgtttggccaatagccgatgcca
WT_610_2.610(1>891)         ←  ATACACTAGATGAGTATGACTGAACAAGATATTCCCAAAAAAGAATCGTGCAAAATGTTTGGCCAATAGCCGATGCCA
1754_600_1.600(1>898)       →  ATACACTAGATGAGTATGACTGAACAAGATATTCCCAAAAAAGAATCGTGCAAAATGTTTGGCCAATAGCCGATGCCA
1754_610_2.610(1>847)       ←  ATACACTAGATGAGTATGACTGAACAAGATATTCCCAAAAAAGAATCGTGCAAAATGTTTGGCCAATAGCCGATGCCA
WT_600_1.600(1>881)         →  ATACACTAGATGAGTATGACTGAACAAGATATTCCCAAAAAAGAATCGTGCAAAATGTTTGGCCAATAGCCGATGCCA

                               27850     27860     27870     27880     27890     27900     27910     27920
                               AAGCGAACAATATGATTTATCCTTTGTCAAATTGTACATGAATATAATTATTTCGCATCTTTGTCAAATTAGATTTAT
PH207_w2_flanking(1>32227)  →  aagcgaacaatatgatttatcctttgtcaaattgtacatgaatataattatttcgcatctttgtcaaattagatttat
WT_610_2.610(1>891)         ←  AAGCGAACAATATGATTTATCCTTTGTCAAATTGTACATGAATATAATTATTTCGCATCTTTGTCAAATTAGATTTAT
1754_600_1.600(1>898)       →  AAGCGAACAATATGATTTATCCTTTGTCAAATTGTACATGAATATAATTATTTCGCATCTTTGTCAAATTAGATTTAT
1754_610_2.610(1>847)       ←  AAGCGAACAATATGATTTATCCTTTGTCAAATTGTACATGAATATAATTATTTCGCATCTTTGTCAAATTAGATTTAT
WT_600_1.600(1>881)         →  AAGCGAACAATATGATTTATCCTTTGTCAAATTGTACATGAATATAATTATTTCGCATCTTTGTCAAATTAGATTTAT

                               27930     27940     27950     27960     27970     27980     27990     28000
                               CCTTCCTAAAGGACTAACGGTTCTGTATGCATACACTATCATTTGGCTTCTTACAAAATGTTGTTTTGGTGTATATAG
PH207_w2_flanking(1>32227)  →  ccttcctaaaggactaacggttctgtatgcatacactatcatttggcttcttacaaaatgttgttttggtgtatatag
WT_610_2.610(1>891)         ←  CCTTCCTAAAGGACTAACGGTTCTGTATGCATACACTATCATTTGGCTTCTTACAAAATGTTGTTTTGGTGTATATAG
1754_600_1.600(1>898)       →  CCTTCCTAAAGGACTAACGGTTCTGTATGCATACACTATCATTTGGCTTCTTACAAAATGTTGTTTTGGTGTATATAG
1754_610_2.610(1>847)       ←  CCTTCCTAAAGGACTAACGGTTCTGTATGCATACACTATCATTTGGCTTCTTACAAAATGTTGTTTTGGTGTATATAG
WT_600_1.600(1>881)         →  CCTTCCTAAAGGACTAACGGTTCTGTATGCATACACTATCATTTGGCTTCTTACAAAATGTTGTTTTGGTGTATATAG

                               28010     28020     28030     28040     28050     28060     28070     28080
                               ATAAATACAACCGTTTAATTTTAAACCAACGAACATACACATACAAACAAAAGCTCCACAAGTCTCTGAAACTTATAT
PH207_w2_flanking(1>32227)  →  ataaatacaaccgtttaattttaaaccaacgaacatacacatacaaacaaaagctccacaagtctctgaaacttatat
WT_610_2.610(1>891)         ←  ATAAATACAACCGTTTAATTTTAAACCAACGAACATACACATACAAACAAAAGCTCCACAAGTCTCTGAAACTTATAT
1754_600_1.600(1>898)       →  ATAAATACAACCGTTTAATTTTAAACCAACGAACATACACATACAAACAAAAGCTCCACAAGTCTCTGAAACTTATAT
1754_610_2.610(1>847)       ←  ATAAATACAACCGTTTAATTTTAAACCAACGAACATACACATACAAACAAAAGCTCCACAAGTCTCTGAAACTTATAT
WT_600_1.600(1>881)         →  ATAAATACAACCGTTTAATTTTAAACCAACGAACATACACATACAAACAAAAGCTCCACAAGTCTCTGAAACTTATAT

                               28090     28100     28110     28120     28130     28140     28150
                               CTCCACAACCACAGCCAATTAAACACTTCAACAAATCCCCAAAATGTAACTTGCTACTCCCTATCGTATGCCCCACTC
PH207_w2_flanking(1>32227)  →  ctccacaaccacagccaattaaacacttcaacaaatccccaaaatgtaacttgctactccctatcgtatgccccactc
WT_610_2.610(1>891)         ←  CTCCACAACCACAGCCAATTAAACACTTCAACAAATCCCCAAAATGTAACTTGCTACTCCCTATCGTATGCCCCACTC
1754_600_1.600(1>898)       →  CTCCACAACCACAGCCAATTAAACACTTCAACAAATCCCCAAAATGTAACTTGCTACTCCCTATCGTATGCCCCACTC
1754_610_2.610(1>847)       ←  CTCCACAACCACAGCCAATTAAACACTTCAACAAATCCCCAAAATGTAACTTGCTACTCCCTATCGTATGCCCCACTC
WT_600_1.600(1>881)         →  CTCCACAACCACAGCCAATTAAACACTTCAACAAATCCCCAAAATGTAACTTGCTACTCCCTATCGTATGCCCCACTC

                               28160     28170     28180     28190     28200     28210     28220     28230
                               AGTTCAGAAAACTAATCAATGTTTTAATTGCGGAACCAAGAAGTTTCCTCATCCCCACGACCAATAATAATTCTTCTC
PH207_w2_flanking(1>32227)  →  agttcagaaaactaatcaatgttttaattgcggaaccaagaagtttcctcatccccacgaccaataataattcttctc
WT_610_2.610(1>891)         ←  AGTTCAGAAAACTAATCAATGTTTTAATTGCGGAACCAAGAAGTTTCCTCATCCCCACGACCAATAATAATTCTTCTC
1754_600_1.600(1>898)       →  AGTTCAGAAAACTAATCAATGTTTTAATTGCGGAACCAAGAAGTTTCCTCATCCCCACGACCAATAATAATTCTTCTC
1754_610_2.610(1>847)       ←  AGTTCAGAAAACTAATCAATGTTTTAATTGCGGAACCAAGAAGTTTCCTCATCCCCACGACCAATAATAATTCTTCTC
WT_600_1.600(1>881)         →  AGTTCAGAAAACTAATCAATGTTTTAATTGCGGAACCAAGAAGTTTCCTCATCCCCACGACCAATAATAATTCTTCTC
WT_w2_10_r.610(1>119)       ←          AAACTAATCAATGTTTTAATTGCGGAACCAAGAAGTTTCCTCATCCCCACGACCAATAATAATTCTTCTC

                               28240     28250     28260     28270     28280     28290     28300     28310
                               TGATGCCATGTCACCTGTTGGTGTCCTCCTCCACATACTAATCTTACTCAGGAACCAACCAATTAGTCTGTCCAAATT
PH207_w2_flanking(1>32227)  →  tgatgccatgtcacctgttggtgtcctcctccacatactaatcttactcaggaaccaaccaattagtctgtccaaatt
WT_610_2.610(1>891)         ←  TGATGCCATGTCACCTGTTGGTGTCCTCCTCCACATACTAATCTTAC
1754_600_1.600(1>898)       →  TGATGCCATGTCACCTGTTGGTGTCCTCCTCCACATACTAATCTTACTCAGGAACCAACCAATTAGTCTGTCCAAATT
1754_610_2.610(1>847)       ←  TGATGCCATGTCACCTGTTGGTGTCCTCCTCCACATACTAATCT-A
WT_600_1.600(1>881)         →  TGATGCCATGTCACCTGTTGGTGTCCTCCTCCACATACTAATCTTACTCAGGAACCAACCAATTAGTCTGTCCAAATT
WT_w2_10_r.610(1>119)       ←  TGATGCCATGTCACCTGTTGGTGTCCTCCTCCACATACTAATCTTACTC

                               28320     28330     28340     28350     28360     28370     28380     28390
                               TTAAGTGAATAAGCAataacatgaaagttaaaacgcttggtttaaggcaaaatttcaacagggcaatgcaaagttcac
PH207_w2_flanking(1>32227)  →  ttaagtgaataagcaataacatgaaagttaaaacgcttggtttaaggcaaaatttcaacagggcaatgcaaagttcac
1754_600_1.600(1>898)       →  TTAAGTGAATAAGCA
WT_600_1.600(1>881)         →  TTAAGTG
```

```
                                   28400     28410     28420     28430     28440     28450     28460     28470
                                 acctattccttactcttccgcaaaccacccacatgttttggcccgtaccaaaacaaaactaatcccaactgcagcagt
PH207_w2_flanking(1>32227)  →    acctattccttactcttccgcaaaccacccacatgttttggcccgtaccaaaacaaaactaatcccaactgcagcagt

                                     28480     28490     28500     28510     28520     28530     28540
                                 gacctcgacaaaaggttcctcaaatcgcgcacacaccagaccggcaaagcacaatcccccgcgtcccagggttacagc
PH207_w2_flanking(1>32227)  →    gacctcgacaaaaggttcctcaaatcgcgcacacaccagaccggcaaagcacaatcccccgcgtcccagggttacagc

                               28550     28560     28570     28580     28590     28600     28610     28620
                                 ttacagggaggcaacaacggcggcggatttccagaggcgaggggcaagggactcacggtcggcggatatcttgaactg
PH207_w2_flanking(1>32227)  →    ttacagggaggcaacaacggcggcggatttccagaggcgaggggcaagggactcacggtcggcggatatcttgaactg

                                 28630     28640     28650     28660     28670     28680     28690     28700
                                 ctcgagggcggcgacgctggcagcctttgccacggcctgcgcctgggcctccacgccggcggcgtcgcaccgcaccac
PH207_w2_flanking(1>32227)  →    ctcgagggcggcgacgctggcagcctttgccacggcctgcgcctgggcctccacgccggcggcgtcgcaccgcaccac

                                   28710     28720     28730     28740     28750     28760     28770     28780
                                 cccggagcgccgcctgccgccgccggccaccctctggcagagggccgacgacgacccccgcggcttggcggtggtggc
PH207_w2_flanking(1>32227)  →    cccggagcgccgcctgccgccgccggccaccctctggcagagggccgacgacgacccccgcggcttggcggtggtggc

                                   28790     28800     28810     28820     28830     28840     28850     28860
                                 ggctgctgctgcggcggtggtcgctgacgtcgtggtcgccattatctagatagattagggaagacagaaagctaccgc
PH207_w2_flanking(1>32227)  →    ggctgctgctgcggcggtggtcgctgacgtcgtggtcgccattatctagatagattagggaagacagaaagctaccgc

                                     28870     28880     28890     28900     28910     28920     28930
                                 ctttcgtgagggaacggaaccaatgcgacgagaatcgcagagataaccagagagggaaaggagacggaggcggggcga
PH207_w2_flanking(1>32227)  →    ctttcgtgagggaacggaaccaatgcgacgagaatcgcagagataaccagagagggaaaggagacggaggcggggcga

                               28940     28950     28960     28970     28980     28990     29000     29010
                                 ggagcagaaactctcgtgcgcttgcctctctacctctcttcccgctcagttcttggtgcgagggtcctcggaggtagg
PH207_w2_flanking(1>32227)  →    ggagcagaaactctcgtgcgcttgcctctctacctctcttcccgctcagttcttggtgcgagggtcctcggaggtagg

                                 29020     29030     29040     29050     29060     29070     29080     29090
                                 taggtgcgggatttcaccgtcgcattagcgaaatcggaggccttggcaacttcccaagctcctctttattgatgcgta
PH207_w2_flanking(1>32227)  →    taggtgcgggatttcaccgtcgcattagcgaaatcggaggccttggcaacttcccaagctcctctttattgatgcgta

                                   29100     29110     29120     29130     29140     29150     29160     29170
                                 cccatttttatgacgcgtgttgggcgtggtggcggtggactcggtggcatcggggaggagaaaggagatgagccgga
PH207_w2_flanking(1>32227)  →    cccatttttatgacgcgtgttgggcgtggtggcggtggactcggtggcatcggggaggagaaaggagatgagccgga

                                   29180     29190     29200     29210     29220     29230     29240     29250
                                 ggatgagagcgtagcggagtgtgatgtgctctggcctgtgattggctgcgcccgcagcgttctatccgcaacccttat
PH207_w2_flanking(1>32227)  →    ggatgagagcgtagcggagtgtgatgtgctctggcctgtgattggctgcgcccgcagcgttctatccgcaacccttat

                                     29260     29270     29280     29290     29300     29310     29320
                                 ctccctcgtcactgacaccagatgcagcagcttcgcttctgtatggcatggcctgcgacgggacgtggggactggtac
PH207_w2_flanking(1>32227)  →    ctccctcgtcactgacaccagatgcagcagcttcgcttctgtatggcatggcctgcgacgggacgtggggactggtac

                               29330     29340     29350     29360     29370     29380     29390     29400
                                 tccgtacatgagcgcccgtggctacctcacgcgcgggcgctgggcgcagccgtctcctgcgcctgatggcgaagcgtg
PH207_w2_flanking(1>32227)  →    tccgtacatgagcgcccgtggctacctcacgcgcgggcgctgggcgcagccgtctcctgcgcctgatggcgaagcgtg
```

	29410 29420 29430 29440 29450 29460 29470 29480
	tcgacgtggtgaccacactctcctcgcagtggcgctggcgcaggcacagattttctggactaaaggggttcagtattt
PH207_w2_flanking(1>32227) →	tcgacgtggtgaccacactctcctcgcagtggcgctggcgcaggcacagattttctggactaaaggggttcagtattt

	29490 29500 29510 29520 29530 29540 29550 29560
	caggtcttcaggcgtgatggatgcagtgttcaccattttatttttactgtagcgaatttcattttttaccaaactttg
PH207_w2_flanking(1>32227) →	caggtcttcaggcgtgatggatgcagtgttcaccattttatttttactgtagcgaatttcattttttaccaaactttg

	29570 29580 29590 29600 29610 29620 29630 29640
	acttttgtatttttgaatttggaaccgaaatctatcaaaaatcggaccgaaactcgtatctcggccagcagcgaaaac
PH207_w2_flanking(1>32227) →	acttttgtatttttgaatttggaaccgaaatctatcaaaaatcggaccgaaactcgtatctcggccagcagcgaaaac

	29650 29660 29670 29680 29690 29700 29710
	aggaaatttcaccgaatttcggtgatttcccggcgagaaagtaaactatggatgaatgaccgtctaaacatgctaaga
PH207_w2_flanking(1>32227) →	aggaaatttcaccgaatttcggtgatttcccggcgagaaagtaaactatggatgaatgaccgtctaaacatgctaaga

	29720 29730 29740 29750 29760 29770 29780 29790
	ctattttcagctattctagctcattctctatcatatttttattttaaactttactatataaattagactatctacagt
PH207_w2_flanking(1>32227) →	ctattttcagctattctagctcattctctatcatatttttattttaaactttactatataaattagactatctacagt

	29800 29810 29820 29830 29840 29850 29860 29870
	gcaaaataatatttaacacgtctatatacacgatctattgtatgatgtgtcaaggactcaaggacgtgattggctaca
PH207_w2_flanking(1>32227) →	gcaaaataatatttaacacgtctatatacacgatctattgtatgatgtgtcaaggactcaaggacgtgattggctaca

	29880 29890 29900 29910 29920 29930 29940 29950
	tgaatttagcccagccagacttttggcatccagatttagttagtgccggaaccgtttaatttattataatctcgaat
PH207_w2_flanking(1>32227) →	tgaatttagcccagccagacttttggcatccagatttagttagtgccggaaccgtttaatttattataatctcgaat

	29960 29970 29980 29990 30000 30010 30020 30030
	ttatttggatgcctaatgatatcaaagattaagaccccgtttggtttggggtgactaaagtttagtcacttttaatct
PH207_w2_flanking(1>32227) →	ttatttggatgcctaatgatatcaaagattaagaccccgtttggtttggggtgactaaagtttagtcacttttaatct

	30040 30050 30060 30070 30080 30090 30100
	ctaaaaagcaggggtcaccaaagagtaactaaaagtgagtaaagtaatattttttttacgttatttactctctctact
PH207_w2_flanking(1>32227) →	ctaaaaagcaggggtcaccaaagagtaactaaaagtgagtaaagtaatattttttttacgttatttactctctctact

	30110 30120 30130 30140 30150 30160 30170 30180
	ttcttcttatagcaaacaccactaattagtagggtaatacagtcattattcacaacaattaatgttttttagtccggt
PH207_w2_flanking(1>32227) →	ttcttcttatagcaaacaccactaattagtagggtaatacagtcattattcacaacaattaatgttttttagtccggt

	30190 30200 30210 30220 30230 30240 30250 30260
	ttaatcactggaaccaaacgagatactttagtgactaaactttagtcagtctagtgactaaaggaaccaaacatggtc
PH207_w2_flanking(1>32227) →	ttaatcactggaaccaaacgagatactttagtgactaaactttagtcagtctagtgactaaaggaaccaaacatggtc

	30270 30280 30290 30300 30310 30320 30330 30340
	taatttaaatttagtagtctgtggaacacaccacaaaagagaactcggaaatccacgtttctaacaggataataaata
PH207_w2_flanking(1>32227) →	taatttaaatttagtagtctgtggaacacaccacaaaagagaactcggaaatccacgtttctaacaggataataaata

	30350 30360 30370 30380 30390 30400 30410 30420
	aataggtaaacgaagttttcaacattgataaacaatttatacatcacataattataaaatacttacatagcaagtctg
PH207_w2_flanking(1>32227) →	aataggtaaacgaagttttcaacattgataaacaatttatacatcacataattataaaatacttacatagcaagtctg

30430 30440 30450 30460 30470 30480 30490

agcaaataatgaaagataaattatttcacataaaaccaacaaaaactaaacagctttgaacactaaagtaggggcaga

PH207_w2_flanking(1>32227) → agcaaataatgaaagataaattatttcacataaaaccaacaaaaactaaacagctttgaacactaaagtaggggcaga

30500 30510 30520 30530 30540 30550 30560 30570

gatgtaaagaataagattttatgatcgcaatggtaaaggaagtcttgttgaataacaattagctctggcatctaccta

PH207_w2_flanking(1>32227) → gatgtaaagaataagattttatgatcgcaatggtaaaggaagtcttgttgaataacaattagctctggcatctaccta

30580 30590 30600 30610 30620 30630 30640 30650

gctacaatataaggaaataaaaattagagcacacaatatacacaacaagacttatccgactagatggcgctagtctcc

PH207_w2_flanking(1>32227) → gctacaatataaggaaataaaaattagagcacacaatatacacaacaagacttatccgactagatggcgctagtctcc

30660 30670 30680 30690 30700 30710 30720 30730

caactctcaaggatgtgctaaggctatttggctacctatttagttttgtttttgcagagaaatgtgctctagtttgta

PH207_w2_flanking(1>32227) → caactctcaaggatgtgctaaggctatttggctacctatttagttttgtttttgcagagaaatgtgctctagtttgta

30740 30750 30760 30770 30780 30790 30800 30810

aggcatgatgacttttaactcattatatttagaagcttaataaggtgataccgtactttatggtattcttttatgaac

PH207_w2_flanking(1>32227) → aggcatgatgacttttaactcattatatttagaagcttaataaggtgataccgtactttatggtattcttttatgaac

30820 30830 30840 30850 30860 30870 30880

acacttatataagttgatcgttggttacagtttgtgagttatgggaattctataagagcatatccaacaacgcattaa

PH207_w2_flanking(1>32227) → acacttatataagttgatcgttggttacagtttgtgagttatgggaattctataagagcatatccaacaacgcattaa

30890 30900 30910 30920 30930 30940 30950 30960

agtggtgcctcaaattgaaatatagggttttacgcaaaaaaaaatcctactccaagtatctcatttataaaatttggt

PH207_w2_flanking(1>32227) → agtggtgcctcaaattgaaatatagggttttacgcaaaaaaaaatcctactccaagtatctcatttataaaatttggt

30970 30980 30990 31000 31010 31020 31030 31040

caaaaaactatagggcatcctttcaagtggctcaagtatattatatcgtaatagactgcccctataatctagatttgt

PH207_w2_flanking(1>32227) → caaaaaactatagggcatcctttcaagtggctcaagtatattatatcgtaatagactgcccctataatctagatttgt

31050 31060 31070 31080 31090 31100 31110 31120

ggttttactgttggagcagaatattttattggtgtcataattctacaaaatatacttatttttaaactatagggcatt

PH207_w2_flanking(1>32227) → ggttttactgttggagcagaatattttattggtgtcataattctacaaaatatacttatttttaaactatagggcatt

31130 31140 31150 31160 31170 31180 31190 31200

tttatagatcacacactcacgctgttagagatgctctaagagactcatgaaaaaagaaatataagtatgacttgtaag

PH207_w2_flanking(1>32227) → tttatagatcacacactcacgctgttagagatgctctaagagactcatgaaaaaagaaatataagtatgacttgtaag

31210 31220 31230 31240 31250 31260 31270

ctccagcaagaaggtagccttttgggcgtcatgtatgagtcagactcttaagtgaaaccagttcatgcaaaactacga

PH207_w2_flanking(1>32227) → ctccagcaagaaggtagccttttgggcgtcatgtatgagtcagactcttaagtgaaaccagttcatgcaaaactacga

31280 31290 31300 31310 31320 31330 31340 31350

atttaaggcgtagccctgttgagtagttgtaagccaacgtagatgttcaacttaacatactgcatttctcataatttg

PH207_w2_flanking(1>32227) → atttaaggcgtagccctgttgagtagttgtaagccaacgtagatgttcaacttaacatactgcatttctcataatttg

31360 31370 31380 31390 31400 31410 31420 31430

aattatacagtacctacgaagccccgaataaatactggaacagaggattccaggtctttggagtttcccatcagctgc

PH207_w2_flanking(1>32227) → aattatacagtacctacgaagccccgaataaatactggaacagaggattccaggtctttggagtttcccatcagctgc

31440 31450 31460 31470 31480 31490 31500 31510

gtgcctcaagcattctgttctgtagaagctgttacacagtgcggcaaagggataatacgaaagagcggagttgggtcg

PH207_w2_flanking(1>32227) → gtgcctcaagcattctgttctgtagaagctgttacacagtgcggcaaagggataatacgaaagagcggagttgggtcg

31520 31530 31540 31550 31560 31570 31580 31590

tgctgggctgtgggcccatgtaagttgggtctagttaggcccatgttattcatattaaaacattgcgccaaaaaaagc

PH207_w2_flanking(1>32227) → tgctgggctgtgggcccatgtaagttgggtctagttaggcccatgttattcatattaaaacattgcgccaaaaaaagc

31600 31610 31620 31630 31640 31650 31660

acaatacatctgatctcgttgttcggatgggcaagagtttgacatgtatatatagtacatgctcttttccttattctt

PH207_w2_flanking(1>32227) → acaatacatctgatctcgttgttcggatgggcaagagtttgacatgtatatatagtacatgctcttttccttattctt

31670 31680 31690 31700 31710 31720 31730 31740

cttcttcttcttaaaaaaaagaaaaagaaaaaagaaaattaaaataagatctagtggcggtgcaggcaacccgcggtc

PH207_w2_flanking(1>32227) → cttcttcttcttaaaaaaaagaaaaagaaaaaagaaaattaaaataagatctagtggcggtgcaggcaacccgcggtc

31750 31760 31770 31780 31790 31800 31810 31820

aagagagtaatcatgctgccacgggatgggcgcggcgcgatcgctcgatcggaagcgtgacatgcgtgcgagctagct

PH207_w2_flanking(1>32227) → aagagagtaatcatgctgccacgggatgggcgcggcgcgatcgctcgatcggaagcgtgacatgcgtgcgagctagct

31830 31840 31850 31860 31870 31880 31890 31900

aggctctgaaatccgaaaggcgaggcaggatgagtacgctctgctctgtcatggccccgcgtccagcaaaccccgacg

PH207_w2_flanking(1>32227) → aggctctgaaatccgaaaggcgaggcaggatgagtacgctctgctctgtcatggccccgcgtccagcaaaccccgacg

31910 31920 31930 31940 31950 31960 31970 31980

tgtttggtgagttgccatggccatggccatggcgtgtataaatggcgcgcccggcgatcggctccggcaccagcactc

PH207_w2_flanking(1>32227) → tgtttggtgagttgccatggccatggccatggcgtgtataaatggcgcgcccggcgatcggctccggcaccagcactc

31990 32000 32010 32020 32030 32040 32050

gcactcgccgagccaagcaatacacagtagtgagcaatcgtaggcagcgcagcaggatacatagcagttagcaccaca

PH207_w2_flanking(1>32227) → gcactcgccgagccaagcaatacacagtagtgagcaatcgtaggcagcgcagcaggatacatagcagttagcaccaca

32060 32070 32080 32090 32100 32110 32120 32130

gcgagcagctgcccgtacgcacgtacgcttgatggcgatggcgatggcgagcttcgtcgctcagctcaaggacatgtt

PH207_w2_flanking(1>32227) → gcgagcagctgcccgtacgcacgtacgcttgatggcgatggcgatggcgagcttcgtcgctcagctcaaggacatgtt

32140 32150 32160 32170 32180 32190 32200 32210

cctcggcctcgtcgaccgcgtcaccggctgccgcggctgtggcggagacaagcaggacgtgccggaggcaaccaagat

PH207_w2_flanking(1>32227) → cctcggcctcgtcgaccgcgtcaccggctgccgcggctgtggcggagacaagcaggacgtgccggaggcaaccaagat

32220 32230

agcaagcctccaggtccttcctgct

PH207_w2_flanking(1>32227) → agcaagcctccaggtccttcctgct
