# Supplementary material for: Combining next‐generation sequencing and progeny testing for rapid identification of induced recessive and dominant mutations in maize M2 individuals
Source: Plant J. 2019 Jul 12;100(4):851–62. doi: 10.1111/tpj.14431 (PMC6899793; doi:10.1111/tpj.14431)
Supplement: Supplementary file 4 — Figure S4. Allele frequencies of the segregating SNPs each in aggregated plots and individual plots. [file TPJ-100-851-s004.pdf]

Figure S4: Allele frequencies of the SNPs segregating in the *DWARF* and *PALE GREEN* families, presented in aggregated plots and individual plots depicting the zygosity of each SNP position for each sequenced individual, annotated as mut (homozygous mutant), as het (heterozygous mutant/WT), and as WT (homozygous WT). Blue lines indicate start and end point of each chromosome (B73_AGPv3) and the filtered SNPs from Table 4 are plotted in red and their position is marked with a red line in the frequency plots. SNP frequencies in each population and the state of zygosity of every SNP in the individuals for (**A**) the 7 homozygous *dwarf* individuals, (**B**) the 9 *DWARF* individuals (7 heterozygotes, 2 homozygotes: MaHe25_DWARF & MaHe33_DWARF), (**C**) the 8 heterozygous *pale green* individuals, (**D**) and in the 8 homozygous *PALE GREEN* individuals. The occurrence of two alternative haplotypes which are each composed of strings of syntenic mutant and wt alleles of neighboring loci in each of the two mutant families is most probably the result of the performed pollen EMS mutagenesis: Mature maize pollen is in the tricellular G1 stage (Mogensen *et al.*, 1995; Friedman, 1999) and the EMS-induced base (Guanine) modifications result in manifested mutations during the following replication, which takes place after fertilization and leads to two alternative double stranded DNA molecules in the chromatids of the paternal chromosomes. Their mitotic separation lead to chimeric M_1 plants and their propagation to the selfed progeny result in segregation of the aforementioned alternative haplotypes among the individuals of the corresponding M_2 families. Thereby patterns of neighboring homozygous mut and WT SNPs alternating in close vicinity are explained. The rare occurrence of heterozygous SNPs within (otherwise) homozygous chromosomal regions can be attributed to zygosity miscalling by SAMtools erroneously identifying SNPs as heterozygous instead of homozygous which is potentially enhanced in positions of low read coverage.

(A)

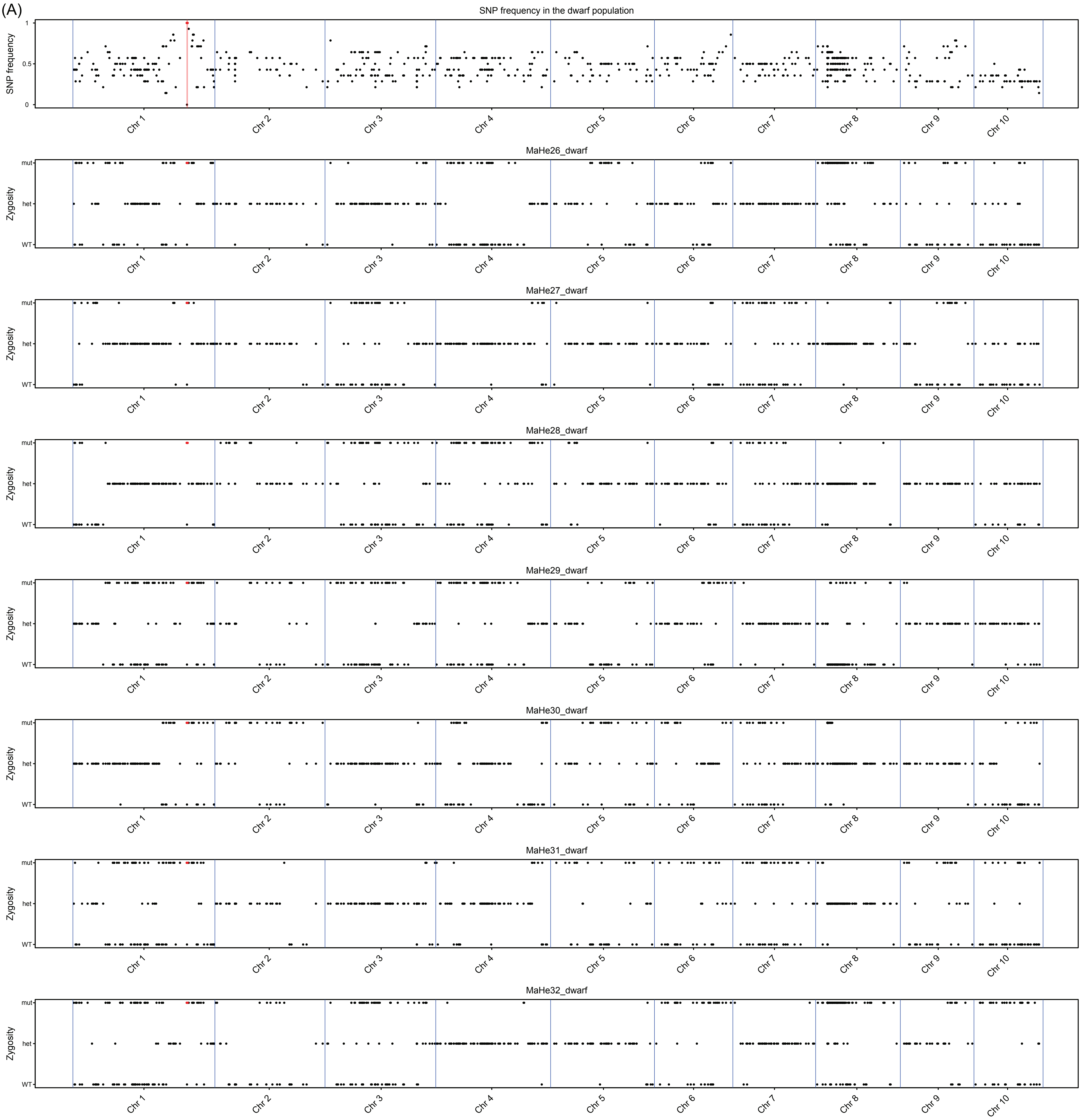
SNP frequency in the dwarf population
SNP frequency
MaHe26_dwarf
MaHe27_dwarf
MaHe28_dwarf
MaHe29_dwarf
MaHe30_dwarf
MaHe31_dwarf
MaHe32_dwarf
Zygosity
mut
het
WT
Chr 1
Chr 2
Chr 3
Chr 4
Chr 5
Chr 6
Chr 7
Chr 8
Chr 9
Chr 10

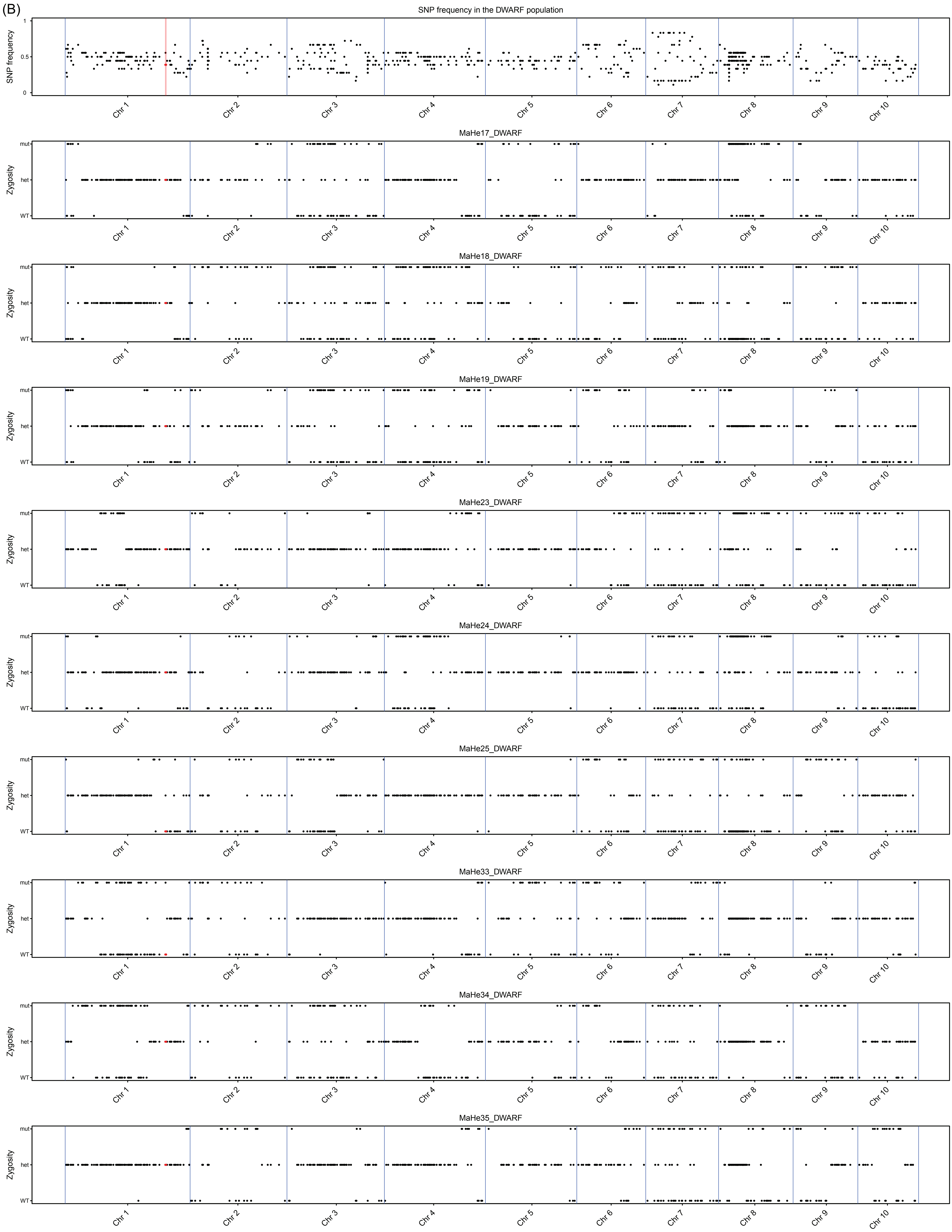

(B)
SNP frequency in the DWARF population
SNP frequency
1
0.5
0
Chr 1
Chr 2
Chr 3
Chr 4
Chr 5
Chr 6
Chr 7
Chr 8
Chr 9
Chr 10
MaHe17_DWARF
MaHe18_DWARF
MaHe19_DWARF
MaHe23_DWARF
MaHe24_DWARF
MaHe25_DWARF
MaHe33_DWARF
MaHe34_DWARF
MaHe35_DWARF
Zygosity
mut
het
WT

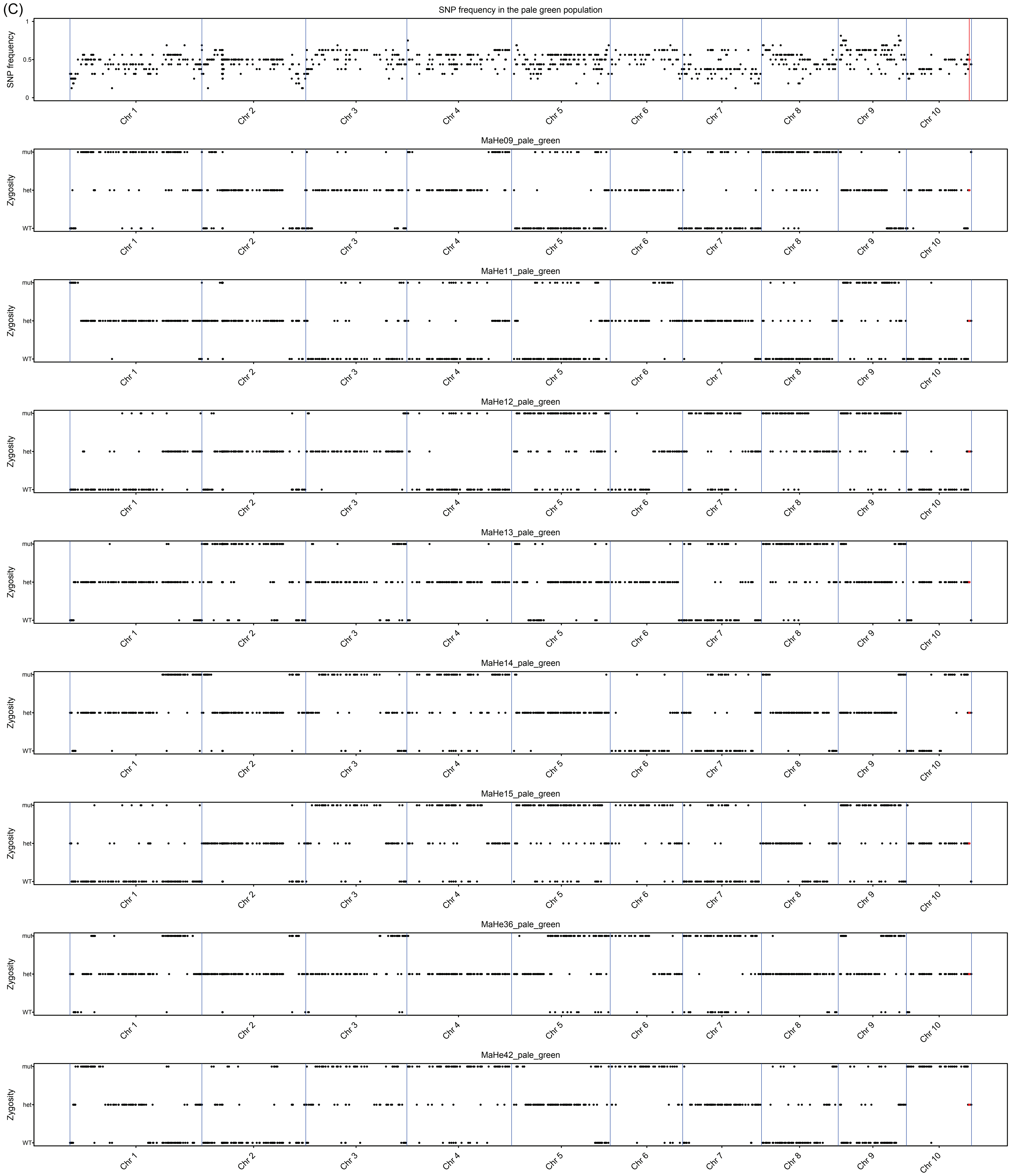

(C)
SNP frequency in the pale green population
SNP frequency
1
0.5
0
Chr 1
Chr 2
Chr 3
Chr 4
Chr 5
Chr 6
Chr 7
Chr 8
Chr 9
Chr 10
MaHe09_pale_green
MaHe11_pale_green
MaHe12_pale_green
MaHe13_pale_green
MaHe14_pale_green
MaHe15_pale_green
MaHe36_pale_green
MaHe42_pale_green
Zygosity
mut
het
WT

(D)

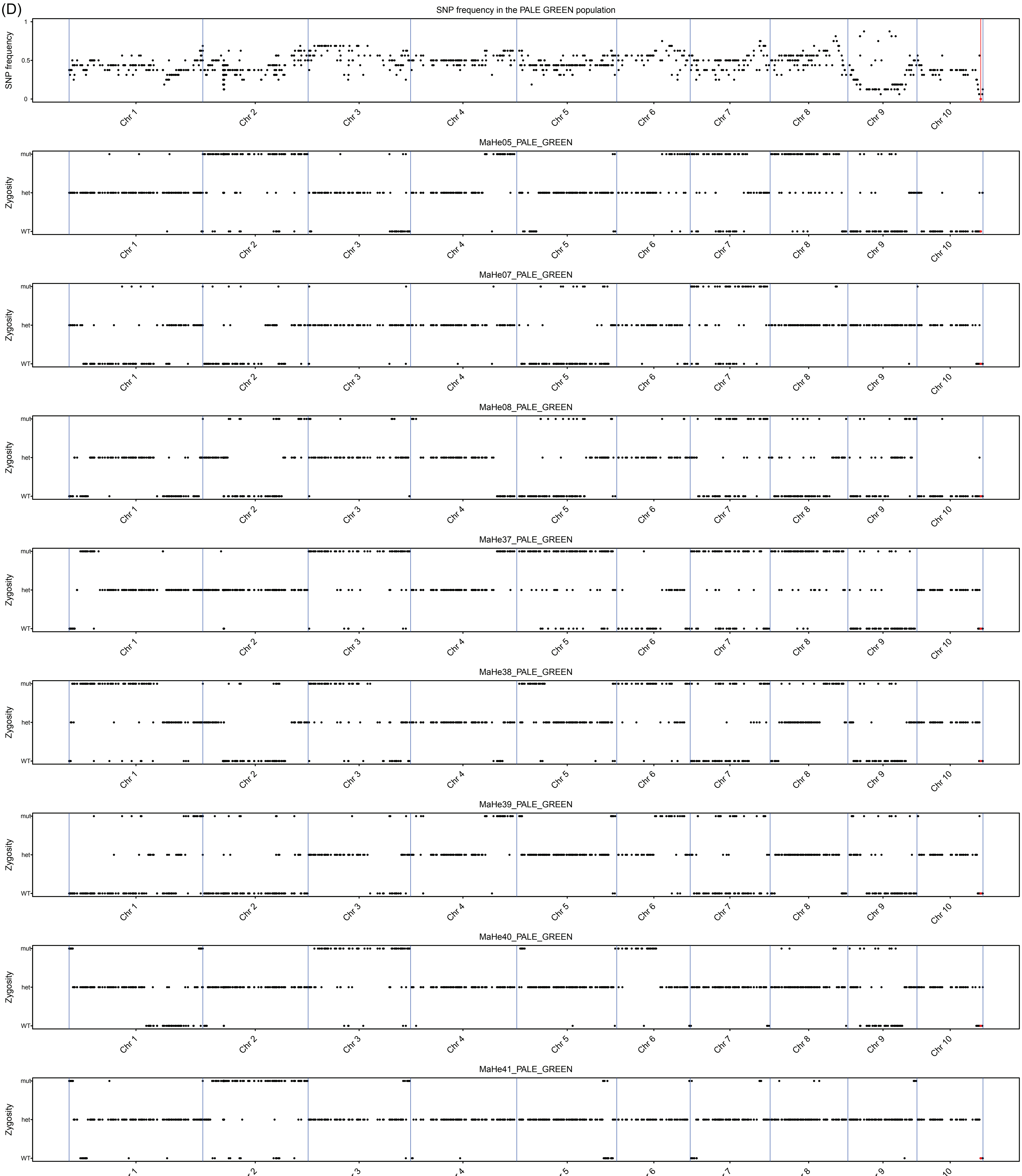
SNP frequency in the PALE GREEN population
SNP frequency
1
0.5
0
Chr 1
Chr 2
Chr 3
Chr 4
Chr 5
Chr 6
Chr 7
Chr 8
Chr 9
Chr 10
MaHe05_PALE_GREEN
MaHe07_PALE_GREEN
MaHe08_PALE_GREEN
MaHe37_PALE_GREEN
MaHe38_PALE_GREEN
MaHe39_PALE_GREEN
MaHe40_PALE_GREEN
MaHe41_PALE_GREEN
Zygosity
mut
het
WT
