# Supplementary material for: Combining next‐generation sequencing and progeny testing for rapid identification of induced recessive and dominant mutations in maize M2 individuals
Source: Plant J. 2019 Jul 12;100(4):851–62. doi: 10.1111/tpj.14431 (PMC6899793; doi:10.1111/tpj.14431)
Supplement: Supplementary file 5 — Figure S5. Dot plots of the 10 MB and 500 kB regions surrounding the an1 (Chr.1) and w2 (Chr.10) gene. [file TPJ-100-851-s005.pdf]

Figure S5: DotPlots with the Gepard software (Krumsiek *et al.*, 2007) of the 10 MB and 500 kB regions surrounding the *an1* (Chr.1) & *w2* (Chr.10) gene loci between the PH207 sequence and the B73_AGPv3 sequence. (**A**) B73_AGPv3 sequence (Chr.10:139030633-149038185; *w2* ± 5 MB) against PH207 (Chr.10:135707976-145724202; *w2* ± 5 MB), (**B**) B73_AGPv3 sequence (Chr.1:236277428-246285679; *an1* ± 5 MB) against PH207 (Chr.1:239834855-249839358; *an1* ± 5 MB), (**C**) B73_AGPv3 sequence (Chr.10:143780633-144288185; *w2* ± 250 kB) against PH207 (Chr.10:140457976-140974202; *w2* ± 250 kB), (**D**) B73_AGPv3 sequence (Chr.1:241027428-241535679; *an1* ± 250 kB) against PH207 (Chr.1:244584855-245089358; *an1* ± 250 kB)

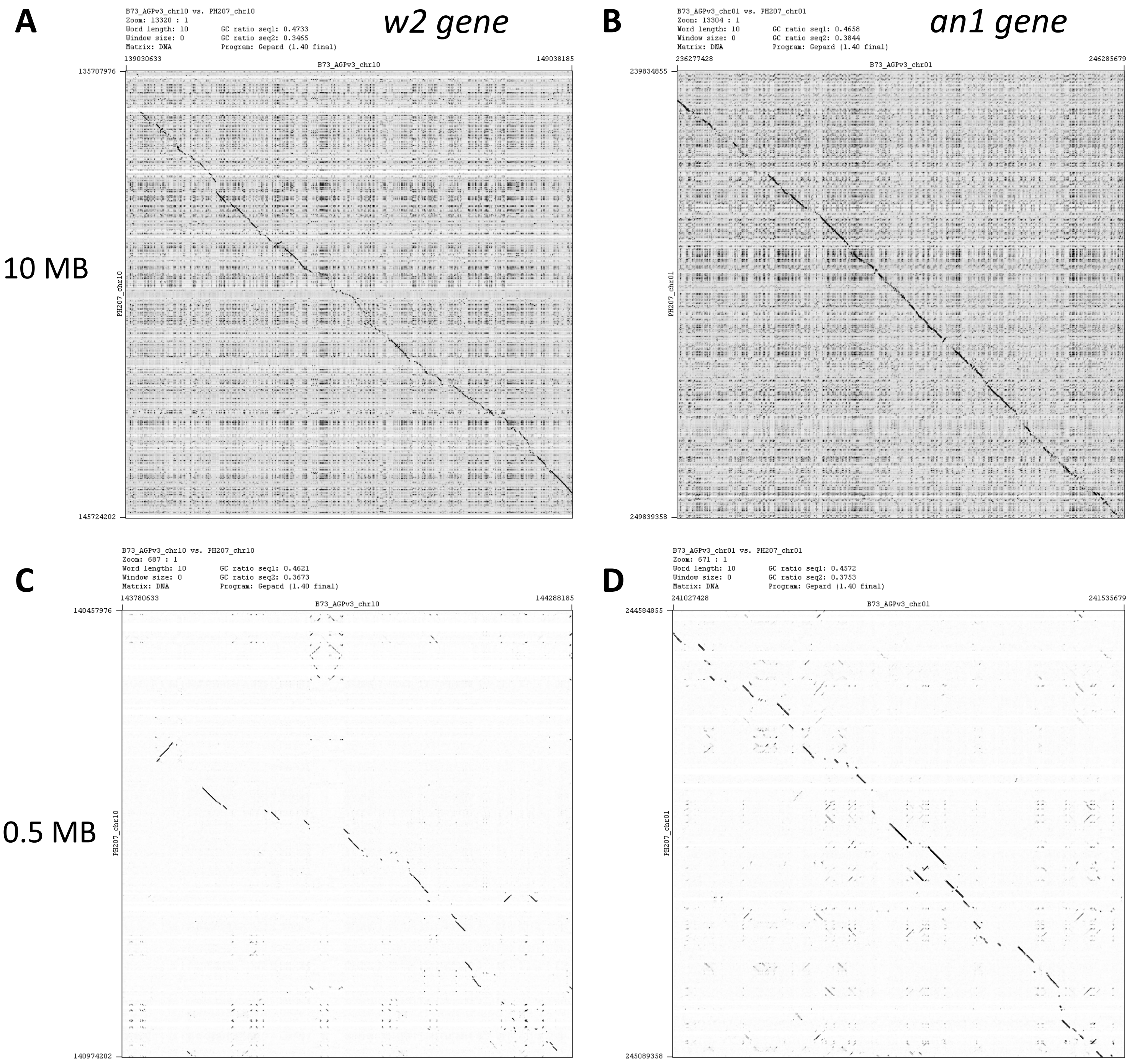
A
B73_AGPv3_chr10 vs. PH207_chr10
Zoom: 13320 : 1
Word length: 10
GC ratio seq1: 0.4733
Window size: 0
GC ratio seq2: 0.3465
Matrix: DNA
Program: Gepard (1.40 final)
w2 gene
139030633
B73_AGPv3_chr10
149038185
135707976
PH207_chr10
145724202
10 MB
B
B73_AGPv3_chr01 vs. PH207_chr01
Zoom: 13304 : 1
Word length: 10
GC ratio seq1: 0.4658
Window size: 0
GC ratio seq2: 0.3844
Matrix: DNA
Program: Gepard (1.40 final)
an1 gene
236277428
B73_AGPv3_chr01
246285679
239834855
PH207_chr01
249839358
C
B73_AGPv3_chr10 vs. PH207_chr10
Zoom: 687 : 1
Word length: 10
GC ratio seq1: 0.4621
Window size: 0
GC ratio seq2: 0.3673
Matrix: DNA
Program: Gepard (1.40 final)
143780633
B73_AGPv3_chr10
144288185
140457976
PH207_chr10
140974202
0.5 MB
D
B73_AGPv3_chr01 vs. PH207_chr01
Zoom: 671 : 1
Word length: 10
GC ratio seq1: 0.4572
Window size: 0
GC ratio seq2: 0.3753
Matrix: DNA
Program: Gepard (1.40 final)
241027428
B73_AGPv3_chr01
241535679
244584855
PH207_chr01
245089358
