# Supplementary material for: Combining next‐generation sequencing and progeny testing for rapid identification of induced recessive and dominant mutations in maize M2 individuals
Source: Plant J. 2019 Jul 12;100(4):851–62. doi: 10.1111/tpj.14431 (PMC6899793; doi:10.1111/tpj.14431)
Supplement: Supplementary file 6 — Table S1. Primer sequences used for the re‐sequencing of the an1 and w2 gene loci in the mutant populations. [file TPJ-100-851-s006.docx]

**Table S1**: Primer sequences used for the re-sequencing of the *an1* & *w2* gene loci in the mutant populations

| **Population** | **Primer #** | **Sequence** |
| --- | --- | --- |
| dwarf | 558 | GTGGTCGGCATTGAGGAACGGGG |
| dwarf | 573 | GGCCTAAGGAGTATAGCTTGAATG |
| dwarf | 576 | GCTGGAGATAGCATAAGCCTCC |
| dwarf | 578 | GAGCAGTGCATGGACTATGTGAAC |
| dwarf | 579 | GTATCATGGTGAGTACTGTACC |
| dwarf | 580 | CTCTATGGTACATGCAAATGGAATAC |
| dwarf | 582 | CATTCAAGCTATACTCCTTAGGCC |
| dwarf | 583 | GGCTGCTGCTAATCATTTCCTTC |
| dwarf | 586 | TGACCACACACATACAGAGATACC |
| dwarf | 588 | GGTACAGTACTCACCATGATAC |
| dwarf | 589 | GTATTCCATTTGCATGTACCATAGAG |
| dwarf | 590 | CTGTGGAGCTGTTCGCGTCCTCCAC |
| dwarf | 612 | GCAGCAGCCTGACAACGTCTCCAG |
| dwarf | 613 | GCTCGTGTATGGTTACCGTTTC |
| dwarf | 620 | GAAACGGTAACCATACACGAGC |
| dwarf | 621 | CTCAAGGTCTTGTGGTTTCCCTGG |
| dwarf | 634 | GGAATGTTTCGTGCACTGTCTCTATG |
| dwarf | 635 | CCAGAATAAAGCACAATCAGTCCAC |
| pale green | 592 | GTTGCTGTGTCAGCACTGACCAG |
| pale green | 594 | CATGTTCTGTATACTTAATATGTG |
| pale green | 595 | TGCAGCTGATGTTGCTATGTGTGC |
| pale green | 598 | CAGCACTGGAGAGGGCGGTATGC |
| pale green | 599 | CACGCTGCCATGGCTCTAATTCAC |
| pale green | 600 | CTGAACAGAGAAGGTTGATCAAAC |
| pale green | 601 | CTGGTCAGTGCTGACACAGCAAC |
| pale green | 602 | CACGGTCAACAAGCATTCCAGCTG |
| pale green | 604 | GCACACATAGCAACATCAGCTGCA |
| pale green | 605 | GATACTCTAGCACCATAGGTGTAG |
| pale green | 608 | GTGAATTAGAGCCATGGCAGCGTG |
| pale green | 609 | GTTTGATCAACCTTCTCTGTTCAG |
| pale green | 610 | CTTTCATGTTATTGCTTATTCAC |
| pale green | 615 | CATGATGTCGCCGTTCACTGGCGC |
| pale green | 617 | GCATTGAAGGAATAAAGATCTCC |
| pale green | 624 | GGAGATCTTTATTCCTTCAATGC |
| pale green | 638 | GATTGGGTCTGGATAGCATCCCTG |
| pale green | 639 | CGTGGCCAGGTGTCTACTACAGC |
