# Supplementary material for: Combining next‐generation sequencing and progeny testing for rapid identification of induced recessive and dominant mutations in maize M2 individuals
Source: Plant J. 2019 Jul 12;100(4):851–62. doi: 10.1111/tpj.14431 (PMC6899793; doi:10.1111/tpj.14431)
Supplement: Supplementary file 7 [file TPJ-100-851-s007.docx]

**SUPPORTING INFORMATION**

**Table S1**: Primer sequences used for the re-sequencing of the *an1* & *w2* gene loci in the mutant populations

**Figure S1**: Phenotype of young dwarf and PH207 ears. (**A**) PH207 ear (**B**) dwarf ear (**C**) Magnification of dwarf ear to show anthers next to the kernels

**Figure S2**: Alignment and strategy view of the re-sequenced *an1* gene in the *dwarf* (1744) and *DWARF* (WT) mutants, B73 (AGPv4; Chr1:244856531-244869541) and PH207 (Chr1:244828512-244841534)

**Figure S3**: Alignment and strategy view of the re-sequenced *w2* gene in the *pale* *green* (1754) and *PALE* *GREEN* (WT) mutants, PH207_w2_flanking & PH207_ws (Chr10:140702976-140735202 & 140707976-140724202)

**Figure S4:** Allele frequencies of the segregating SNPs each in aggregated plots and individual plots depicting the zygosity of each SNP position for each sequenced individual, annotated as mut (homozygous mutant), as het (heterozygous mutant/WT), and as WT (homozygous WT). Blue lines indicate start and end point of each chromosome (B73_AGPv3) and the filtered SNPs from Table 4 are plotted in red and their position is marked with a red line in the frequency plots. SNP frequencies in each population and the state of zygosity of every SNP in the individuals for (**A**) the 7 homozygous *dwarf* individuals, (**B**) the 9 *DWARF* individuals (7 heterozygotes, 2 homozygotes: MaHe25_DWARF & MaHe33_DWARF), (**C**) the 8 heterozygote *pale* *green* individuals, (**D**) and in the 8 homozygous *PALE* *GREEN* individuals. The occurrence of two alternative haplotypes which are each composed of strings of syntenic mutant and wt alleles of neighboring loci in each of the two mutant families is most probably the result of the performed pollen EMS mutagenesis: Mature maize pollen is in the tricellular G1 stage (Mogensen *et al.*, 1995; Friedman, 1999) and the EMS-induced base (Guanine) modifications result in manifested mutations during the following replication, which takes place after fertilization and leads to two alternative double stranded DNA molecules in the chromatids of the paternal chromosomes. Their mitotic separation lead to chimeric M_1_ plants and their propagation to the selfed progeny result in segregation of the aforementioned alternative haplotypes among the individuals of the corresponding M_2_ families. Thereby patterns of neighboring homozygous mut and WT SNPs alternating in close vicinity are explained. The rare occurrence of heterozygous SNPs within (otherwise) homozygous chromosomal regions can be attributed to zygosity miscalling by SAMtools erroneously identifying SNPs as heterozygous instead of homozygous which is potentially enhanced in positions of low read coverage.

**Figure S5:** DotPlots with the Gepard software (Krumsiek *et al.*, 2007) of the 10 MB and 500 kB regions surrounding the *an1* (Chr.1) & *w2* (Chr.10) gene loci between the PH207 sequence and the B73_AGPv3 sequence. (**A**) B73_AGPv3 sequence (Chr.10:139030633-149038185; *w2* ± 5 MB) against PH207 (Chr.10:135707976-145724202; *w2* ± 5 MB), (**B**) B73_AGPv3 sequence (Chr.1:236277428-246285679; *an1* ± 5 MB) against PH207 (Chr.1:239834855-249839358; *an1* ± 5 MB), (**C**) B73_AGPv3 sequence (Chr.10:143780633-144288185; *w2* ± 250 kB) against PH207 (Chr.10:140457976-140974202; *w2* ± 250 kB), (**D**) B73_AGPv3 sequence (Chr.1:241027428-241535679; *an1* ± 250 kB) against PH207 (Chr.1:244584855-245089358; *an1* ± 250 kB)
